# Expanding the inhibitor space of the WWP1 and WWP2 HECT E3 ligases

**DOI:** 10.1080/14756366.2024.2394895

**Published:** 2024-09-02

**Authors:** Ashley P. Dudey, Jake M. Rigby, Gregory R. Hughes, G. Richard Stephenson, Thomas E. Storr, Andrew Chantry, Andrew M. Hemmings

**Affiliations:** a School of Biological Sciences, University of East Anglia, Norwich, UK; b School of Chemistry, Pharmacy & Pharmacology, University of East Anglia, Norwich, UK; c International Research Center for Food and Health, College of Food Science and Technology, Shanghai Ocean University, Shanghai, China

**Keywords:** ubiquitin ligase inhibitors, drug discovery, SAR, WWP1, WWP2

## Abstract

The HECT E3 ubiquitin ligases 1 (WWP1) and 2 (WWP2) are responsible for the ubiquitin-mediated degradation of key tumour suppressor proteins and are dysregulated in various cancers and diseases. Here we expand their limited inhibitor space by identification of NSC-217913 displaying a WWP1 IC_50_ of 158.3 µM (95% CI = 128.7, 195.1 µM). A structure-activity relationship by synthesis approach aided by molecular docking led to compound **11** which displayed increased potency with an IC_50_ of 32.7 µM (95% CI = 24.6, 44.3 µM) for WWP1 and 269.2 µM (95% CI = 209.4, 347.9 µM) for WWP2. Molecular docking yielded active site-bound poses suggesting that the heterocyclic imidazo[4,5-*b*]pyrazine scaffold undertakes a π-stacking interaction with the phenolic group of tyrosine, and the ethyl ester enables strong ion-dipole interactions. Given the therapeutic potential of WWP1 and WWP2, we propose that compound 11 may provide a basis for future lead compound development.

## Graphical Abstract

**Figure:**
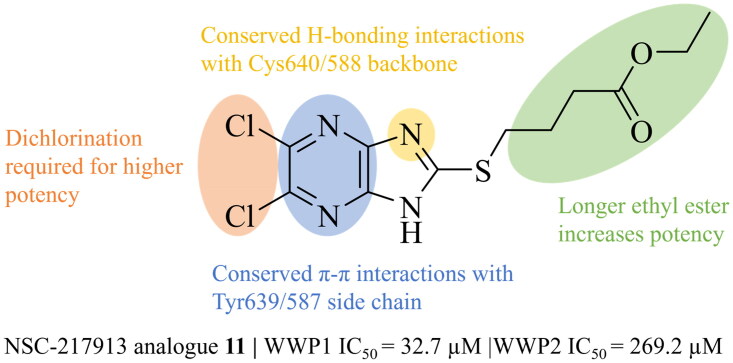

## Introduction

The ubiquitin (Ub) system functions as a catalytic cascade of three core enzymes, Ub-activating (E1), Ub-conjugating (E2) and Ub-ligating (E3), tasked with loading Ub onto targeted proteins yielding a complex array of mono or poly Ub chains^1^. Dysregulation of the ubiquitination system has been widely associated with various diseases, namely cancer, fuelling efforts to identify new therapeutic opportunities^2^. The therapeutic potential of the Ub system has already been demonstrated by the FDA-approved drug Velcade (Bortezomib) used in the treatment of multiple myeloma. This targets the 26S proteasome used downstream during canonical K48-linked ubiquitination^3^^,^^4^. However, as a vital enzyme for proteolytic function, proteasome inhibition has resulted in numerous side-effects^5^. E3 ligases provide an alternative and attractive target for future drug discovery being known for determining the substrate specificity of the Ub system and as such offers the potential of targeting specific malignant pathways^6^.

There are over 600 E3 ligases categorised into three subtypes based on their mechanism of Ub transfer: Really Interesting New Gene (RING), Homologous to E6AP Carboxyl Terminus (HECT) and the more recently characterised RING Between RING (RBR)^7–9^. The HECT-E3 ligases may offer the best druggability as they participate directly in the Ub transfer, unlike the RING-type, and with their catalytic function relying on a single active-site cysteine. Various HECT-E3 ligases have also been highlighted to play fundamental roles in tumour initiation and progression, with the NEDD4 family gaining the most attention given their substantial malignant involvement^10^. Two members of the NEDD4 family, WW domain-containing E3 ligase 1 (WWP1) and 2 (WWP2), are of particular interest due to their targeting of the tumour suppressor protein phosphatase and tensin homolog (PTEN) as well as various other tumour suppressors and transcription factors^11^^,^^12^. To no surprise, both WWP1 and WWP2 dysregulation has been directly linked to cancer, as well as other neurological, inflammatory, and even infectious diseases including COVID-19^13–15^.

There are only a limited number of small-molecule inhibitors identified to target HECT E3 ligases, all with low µM potency^16^. Currently only two target WWP1, the commercially available HECT ligase inhibitor Heclin with an IC_50_ of 6.9 μM and indole-3-carbinol (I3C) and derivatives indicated through cell proliferation studies to bind and inhibit ^11^^,^^17^. Only a handful of potential WWP2 inhibitors have been highlighted in a screen of the National Cancer Institute (NCI) Diversity Set V compound library reported by us previously, of which NSC-288387 with an IC_50_ of 2.3 µM^18^.

Here we expand our screening strategy using differential scanning fluorimetry (DSF) and an *in vitro* enzyme-linked immunosorbent (ELISA)-based autoubiquitination assay to screen both WWP1 and WWP2 against the ‘next in series’ NCI Diversity Set VI compound library with the aim to increase the inhibitor space for these therapeutic targets. We evaluate their structure-activity relationship (SAR) by synthesis aided by molecular docking using X-ray crystal structures of WWP1 and WWP2 solved as part of this study and improved over those previously reported^19^^,^^20^.

## Materials and methods

### Materials

All reagents were purchased from ThermoFisher, Sigma Aldrich and Melford, unless otherwise stated. The diversity set VI compound library was obtained from the National Cancer Institute (NCI, USA). All plasmids were either purchased from Addgene or kindly gifted (see Supporting Information (SI) Table S10). Full details of the protein purification procedures and compound synthesis strategies are described in the SI. The truncated WWP1 and WWP2 constructs used in this study, WWP1- 2,3-linker-WW3-WW4-HECT (WWP1-L34H), WWP1-WW2-2,3-linker-WW3-WW4-HECT (WWP1-2L34H), WWP2-WW2-2,3-linker-HECT (WWP2-LH), full-length WWP2 (WWP2-FL) are further defined in SI Figure S8.

### Differential scanning fluorimetry

A 96 well-plate (MicroAmp Optical) was loaded with 18 µL of 1 µM WWP1-L34H, 3.8 µM WWP1-2L34H and 2.5 µM WWP2-LH, in their respective buffers (SI Table S12) containing 5 × SYPRO orange dye. A 2 µL aliquot of the compound was added to a final concentration ranging from 10 − 100 µM containing 0.1% DMSO before the plate was sealed (MicroAmp Clear Adhesive Film). Both non-protein and DMSO controls were also generated. The plates were briefly centrifuged before the assay was run using an ABI 7500 RT-PCR following the melt curve using ROX^™^ (575 nm) as the ‘pre-set’ fluorescence dye. A standard thermal profile of 25 − 70 °C, rising at 0.5 °C per minute was used. The mid-point melting temperature (Tm) was calculated from either the first derivative or Boltzmann fit to the fluorescence curve using Protein Thermal Shift Software v1.4 (ThermoFisher). Results were further processed, and graphs were produced using Excel.

### ELISA autoubiquitination assay

Cell lysate containing His-tagged WWP1-L34H or GST-tagged WWP2-FL proteins were incubated on either 96-well Clear Pierce glutathione ^21^ or nickel-coated plates for 1 h in PBS. Reaction mixtures of either 3 ng/well GST-Uba1 and 15 ng/well UbcH7 or 10 ng/well His-Uba1 and 150 ng/well His-UbcH7 were incubated together in 25 mM Tris pH 8.0, 100 mM NaCl, 4 mM MgCl_2_ containing 60 ng/well FLAG-ubiquitin and 1.25 mM ATP for 40 min. A prior 1% BSA plate blocking step is required for nickel-coated plates. After plate washing, 2 μL of the compound was added at the desired concentration (0.1 – 1% DMSO) followed by 18 μL of the reaction mixture. This was then incubated for 2 h with 0% and 100% controls before 100 μL of anti-FLAG M2-Peroxidase HRP (1: 10,000 PBST) was added to each well and incubated for 1 h. Finally, 100 μL of 1 × TMB substrate solution (Invitrogen) was added to each well and incubated for up to 10 min until sufficient blue colour change was observed. To stop the reaction, 100 μL of 1 M HCl was added. GST-tagged Uba1 counter-assay followed a similar protocol first incubating cell lysate for 1 h before adding 20 µL of reaction mixture containing 60 ng/well FLAG-ubiquitin, 1.25 mM ATP, and 1 mM compound at 1% DMSO. His-tagged UbcH7 counter-assays preincubated 3 ng/well GST-Uba1 and 200 ng/well UbcH7 with the reaction mixture components (60 ng/well FLAG-ubiquitin, 1.25 mM ATP, and 1 mM compound at 1% DMSO) for 1 h before incubating onto nickel-coated plates for a further 1 h. All other steps were followed. The plates were washed three times with PBST (and 15 mM Imidazole) between each step. Quantification was measured by absorbance read at 450 nm, standardised to the 0% and 100% activity controls. A visual representation of the assay steps is shown in SI Figure S13. All assay optimisations have been previously reported by Watt and colleagues^18^. IC_50_ non-linear regression curves were calculated in GraphPad v10.2 (Prism).

### X-ray crystallography

WWP1-2L34H crystallisation followed a previous report^20^, with protein at 4 mg/mL crystallised in a 96-well MRC 2 sitting drop plates (SWISSCI) at 16 °C. 1 µL drops were plated at 0.4 − 0.6 µL protein in 50 mM Tris pH 8.0, 500 mM NaCl, 1 mM EDTA and 1 mM DTT and mixed with 0.4 − 0.6 µL of reservoir solution. The reservoir contained 100 µL of 100 mM Tris.HCl pH 6.5, 17.5% reagent alcohol (90% ethanol, 5% methanol, 5% isopropanol). Plates were sealed using ClearVue^TM^ sheets (Molecular Dimensions). Clear, flat-rod crystal clusters formed after 2 weeks which were cryo-protected by brief immersion in 100 mM Tris.HCl pH 6.5, 17.5% reagent alcohol plus 30% glycerol and stored in liquid nitrogen. WWP2-LH crystallisation followed the literature protocol^19^ with protein concentrated to 1.5 mg/mL crystallised in a 15-well EasyXtal hanging drop plate (QIAGEN) at 16 °C. DG X-Seal crystal supports (QIAGEN) were loaded with 2 – 4 µL drops of a 1:1 volume ratio of protein to reservoir solution (100 mM MMT pH 6.0, 25% PEG1500), before securing for vapour diffusion against 500 µL of reservoir solution. Crystals of square plate habit formed after 3 – 7 days which were cryo-protected in mother liquor containing 20% glycerol and stored in liquid nitrogen.

Data collection was performed at the Diamond Light Source (Didcot, UK) using the I24 and I04 beamlines. Image data was auto-processed in the AutoProc pipeline^22^ and downloaded for subsequent molecular replacement using PDB ID: 6J1X and PDB ID: 5TJ8 for WWP1–2L34H and WWP2-LH, respectively. Refinement was processed in the CCP4 software suit v8.0^23^ and COOT v0.9.8^24^. The final refined structure of WWP1-2L34H had an Rfree of 0.269 at 3.00 Å resolution, while that of WWP2-LH had an Rfree of 0.275 at 2.05 Å resolution. Data collection and structure refinement statistics can be found in Table S8 of the SI. Molecular images were generated using PyMOL v2.5^25^.

### Molecular docking

Molecular docking was performed using Flare v8.0 (Cresset) which uses the Lead Finder program for docking calculation^26^^,^^27^. For protein and ligand preparation the refined structures of WWP1-2L34H and WWP2-LH were imported into Cresset Flare. Protein preparation was carried out using the default settings at pH 7.4. The molecular structures of the ligands identified in this project were energy minimised with charges at pH 7 prior to docking in Flare^26^. The grid box for WWP1-2L34H was defined according to the proposed binding site cavity close to the active site cysteine (Cys890), via picking of amino acids Tyr639, Cys640, Ser679, Ser698, Ile699, Thr851, Gly852, Thr853, Thr889 and Cys890. The grid box for WWP2-LH was similarly defined surrounding the active site cysteine, Cys838, via picking of key amino acids Tyr587, Cys588, Thr627, Ser646, Ile-647, Thr-799, Gly-800, Thr-801, Thr-837 and Cys-838. The docking score results are listed in SI Table S9, ligand poses discussed are visualised in Figure 4 and in SI Figures S6 and S7.

## Results and discussion

### High-throughput screen of NCI diversity set VI

**Figure 1.**
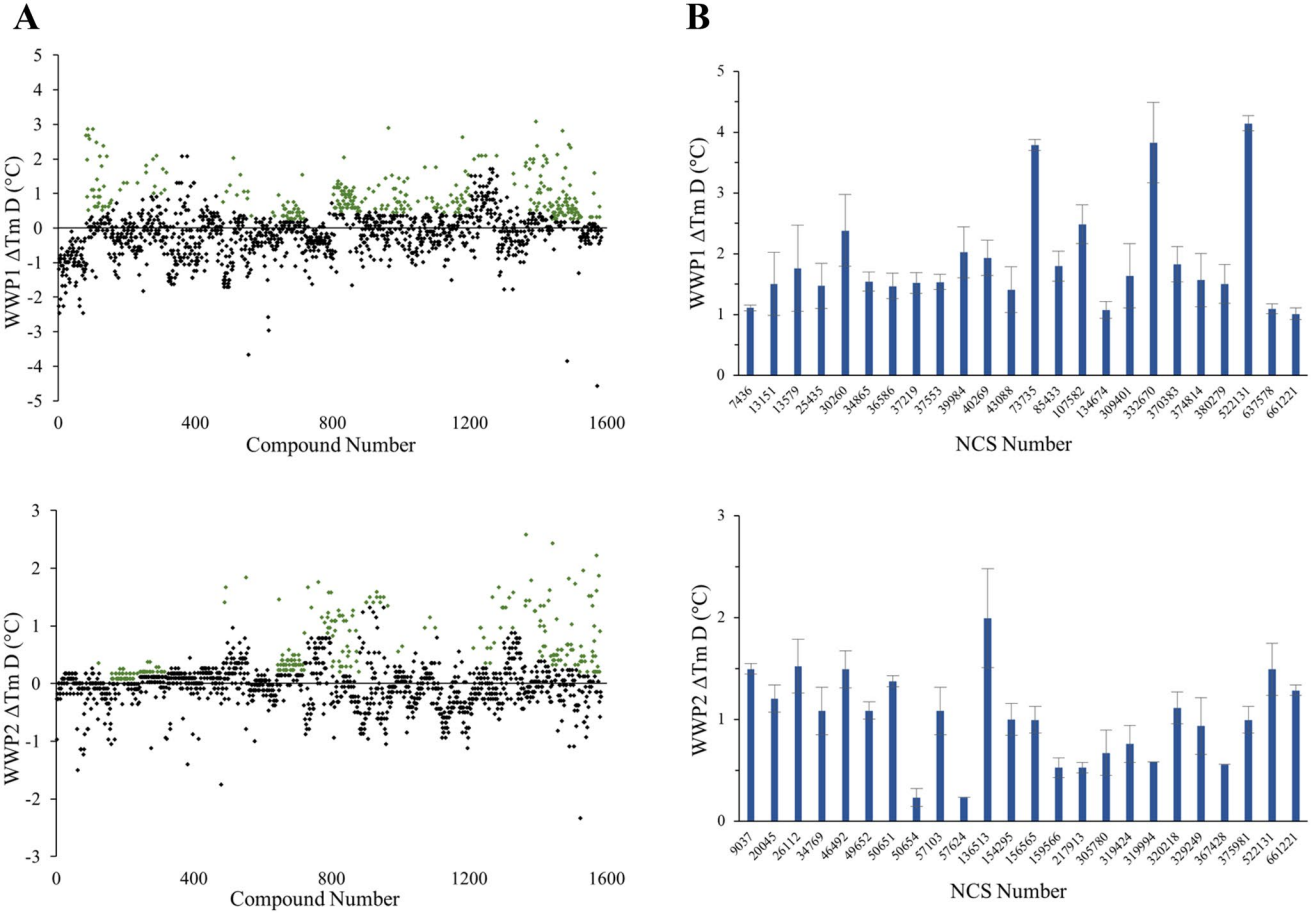
High throughput DSF screen of the NCI Diversity Set VI compound library against WWP1 and WWP2. Assays were carried out using 10 µM compound, containing 0.1% DMSO and normalised by ΔT_m_ calculated from controls. Positive hits (green) displayed had an average ΔT_m_ and associated errors above a threshold of three times the standard deviation of their respective controls (A) Single-shot DSF screen of WWP2-LH (top) and WWP1-L34H (bottom) against NCI Diversity Set VI compounds. (B) Triplicate DSF screen of 24 confirmed hit compounds for WWP1-L34H and WWP2-LH.

Our first step to discover small molecule inhibitors of WWP1 and WWP2 was to screen the NCI Diversity Set VI compound library consisting of 1584 NSC compounds using high-throughput DSF to identify interactors. Given the dynamic nature of these particular E3 ligases, we used the constructs WWP1-L34H and WWP2-LH as we predicted that their thermal profiles would be more stable^19^^,^^20^. Initial single-shot screens were run at 10 µM compound concentration (0.1% DMSO) with midpoint melting temperature (T_m_) determined by the first derivative of the melting curve. A total of 209 and 264 preliminary hits were initially identified as having a Tm above a threshold of three times the standard deviation of the respective WWP1 and WWP2 controls (Figure 1a). These initial positives were then assayed in triplicate, with 24 NSC compounds confirmed to maintain an average ΔTm beyond the threshold for both targets (Figure 1b). PAINS filtering identified four compounds for WWP1 and six compounds for WWP2 as problematic and so were removed as that may display as false positives (SI Table S1 & S2)^28^. Thus, in total, 20 and 18 NSC compounds were identified to interact with WWP1 and WWP2, respectively. Subject to further characterisation, these compounds may provide opportunities for future development in targeted protein degradation (TPD) given the unique capabilities of E3 ligases used in the novel proteolysis targeting chimaera (PROTAC) drug strategy^29^.

**Table 1.** Summary of IC_50_ values from NSC-217913 analogues screened using a WWP1 and WWP2 ELISA autoubiquitination assay.

Compound	Structure	WWP1 IC_50_ (µM)	WWP2 IC_50_ (µM)
**6**/217913	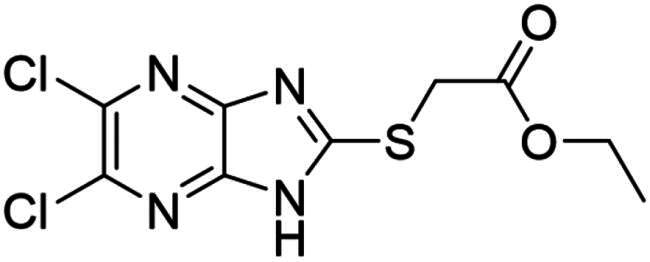	158.3 (128.7, 195.1)^b^	Unstable^a^
**7**	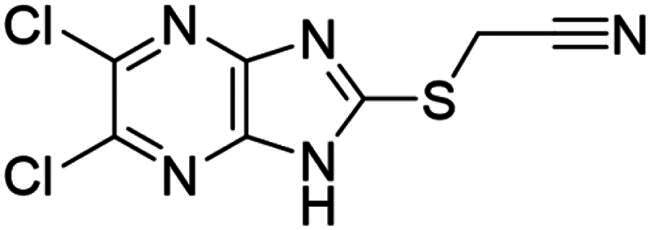	97.29 (77.2, 128.1)^b^	856.3 (786.4, 924.9)^b^
**9**	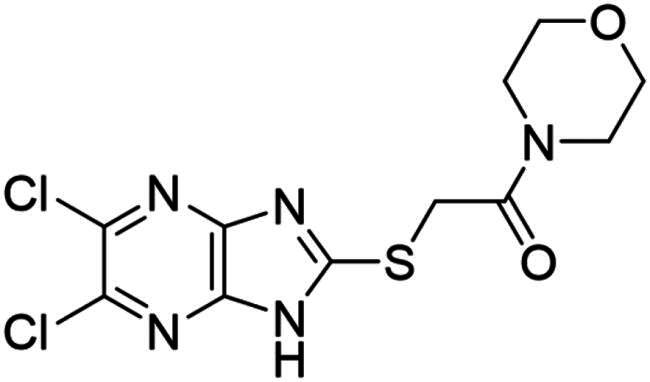	–	621.8 (586.2, 658.9)^b^
**11**	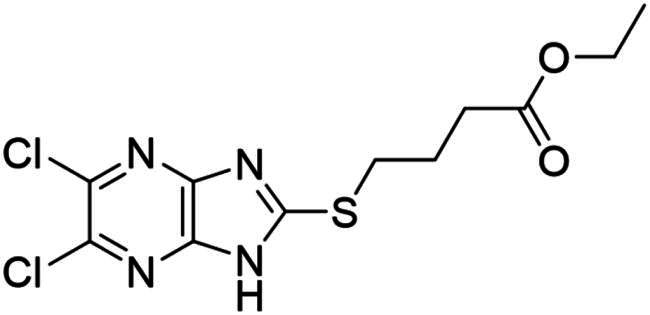	32.74 (24.6, 44.3)^b^	269.2 (209.4, 347.9)^b^
**13**	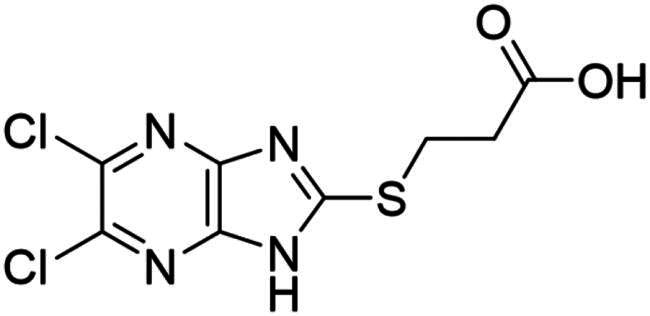	–	603.3 (496.3, 726.5)^b^
**15**	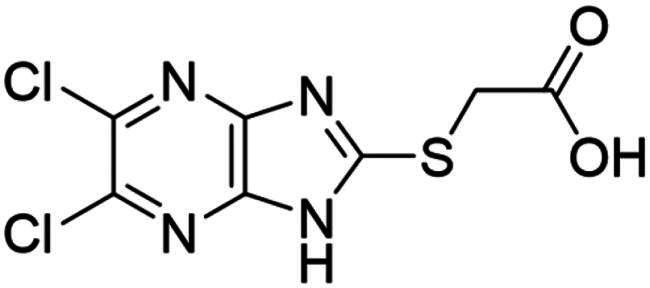	375.5 (256.6, 551.5)^b^	666.4 (575.3, 766.4)^b^

^[a]^Regression curve could not be accurately modelled. ^[b]^The 95% CI range.

### Discovery of small molecule inhibitor NSC-217913

**Figure 2.**
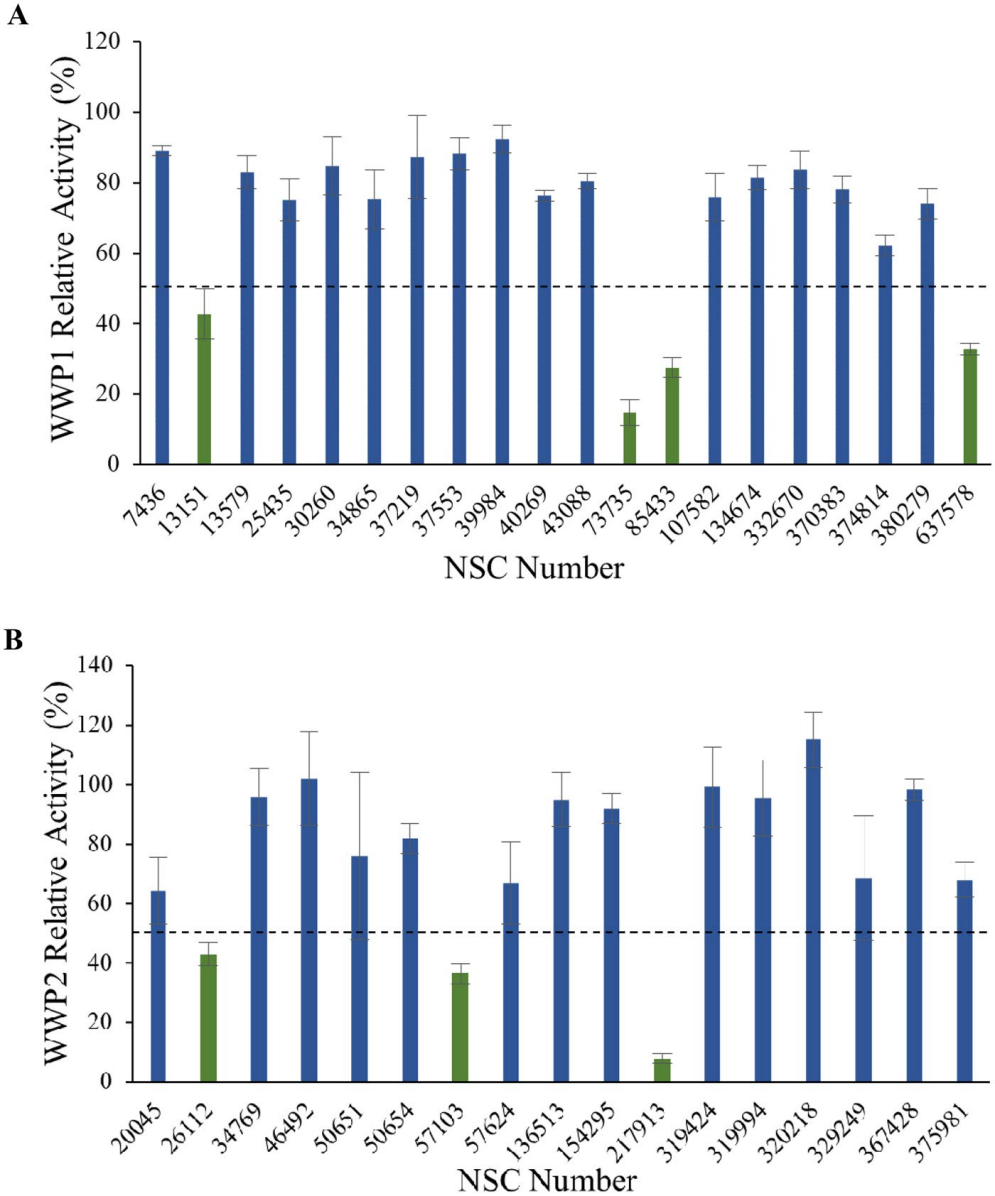
ELISA autoubiquitination assay screen of PAINS-filtered DSF hit compounds against WWP1 (A) and WWP2 (B). Enzyme inhibition was measured at a compound concentration of 500 µM (1% DMSO), with a hit threshold (hits shown in green) of less than 50% relative activity, normalised to their respective 0% and 100% WWP1-L34H and WWP2-FL controls.

The DSF hit compounds were initially screened using a single-shot WWP1 and WWP2 ELISA autoubiquitination assay at 500 µM compound (1% DMSO), measuring inhibition against their relative autoubiquitination activity following protocols previously described^18^. Although full-length constructs would have been preferred, in the case of WWP1, the truncated WWP1-L34H construct was required to overcome autoinhibitory interactions that had previously been shown to result in low activity^20^. Four NSC compounds for WWP1 (13151, 73735, 85433 & 637578) and three NSC compounds for WWP2 (26112, 57103 & 217913) were initially selected having a threshold of less than 50% relative activity when compared to 0% and 100% controls (Figure 2).

These preliminary hit compounds were further screened in dose-dependent ELISA autoubiquitination assays from 500 µM − 5 nM, with three NSC compounds for WWP1 (13151, 73735 & 85433) and only two NSC compounds for WWP2 (57103 & 217913) showing a dose-response (SI Table S4). The most potent NSC compounds (73735, 85433 & 217913), having an IC50 below 100 µM, were selected for cross-reactivity analysis against the other enzyme target, all displaying more inhibition towards WWP1 over WWP2 (SI Table S5). To ensure the intended target was being inhibited and not the downstream Uba1 (E1) and UbcH7 (E2) enzymes used in the catalytic cascade of the assay, a counter autoubiquitination screen was utilised. Upon counter-activity analysis, only with NSC-217913 did UbcH7 retain more than 90% activity and so this was selected as the sole validated hit compound from our inhibition screen (SI Table S6).

**Figure 3.**
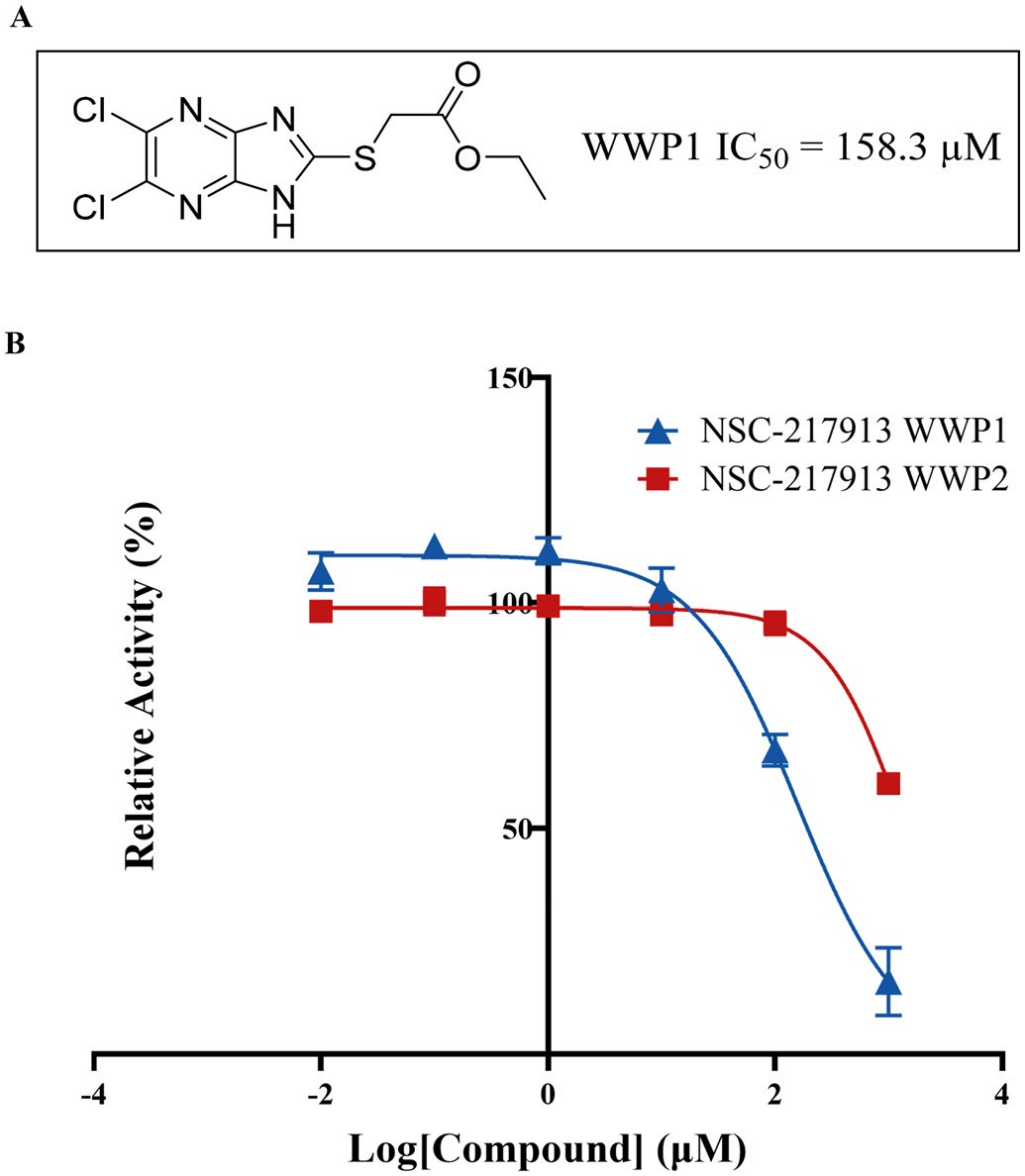
Synthesised NSC-217913 against WWP1 and WWP2. (A) Chemical structure of NSC-217913 and associated WWP1 IC_50_ (B) Dose-dependent ELISA autoubiquitination assay of synthesised NSC-217913. Compound inhibition was measured on a log scale from 1000 – 10 nM (1% DMSO), normalised to 0% and 100% WWP1-L34H (blue triangle) and WWP2-FL (red square) controls. IC_50_ values were calculated from non-linear regression curves fitted in GraphPad.

When used directly from the NCI library, NSC-217913 had IC_50_ of 33.3 µM (95% CI = 29.1, 38.5 µM) and 69.8 µM (95% CI = 59.1, 82.9 µM) against WWP1 and WWP2, respectively. However, once re-synthesised the potency was reduced displaying an IC_50_ of 158.3 µM (95% CI = 128.7, 195.1 µM) against WWP1, with WWP2 inhibition too weak to determine accurately (Figure 3). The NCI sample may have provided a higher potency due to the presence of impurities as indicated by HRMS and ^1^H NMR analysis (Figures S14–S17). Given the low number of small molecule inhibitors against WWP1 and WWP2 present in the literature, we continued working with NSC-217913 aiming to validate it as a novel scaffold by exploring an initial SAR.

### Synthesis and activity of NSC-217913 and analogue library

**Scheme 1.**
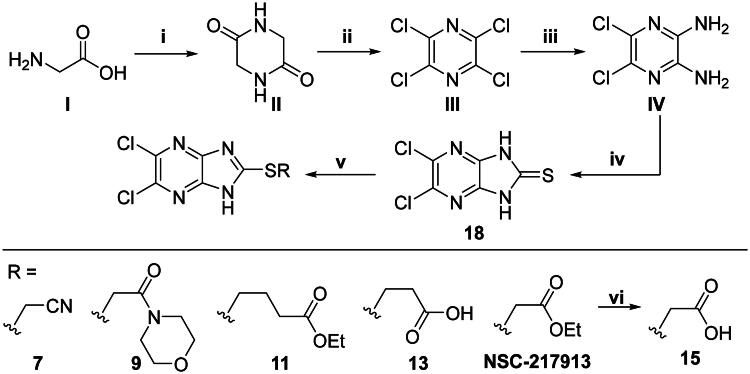
Synthetic route to access **6**, **7**, **9**, **11**, **13** and **15**. i: Ethylene glycol (2.04 M), 175 °C, 18 h, 45%. ii: PCl_5_ (7.0 eq), POCl_3_ (0.44 M), 90 °C – 200 °C, 18 h, 32%. iii: NH_4_OH (25% aq., 0.27 M), 120 °C, 2 d., 31%. iv: Thiocarbonyldiimidazole (2.0 eq), dioxane (0.2 M), 120 °C, 36 h, 34%. v: Br-R (1.05 eq), NaOH (1.5 eq), EtOH (0.067 M), RT, 18 h, Ar or N_2_, 24 – 76%. vi: NaOH (0.15 M, methanol), CH_2_Cl_2_: MeOH (14:11 v/v, 0.063 M), RT, 18 h, 69%.

We sought to design a library which separately varied the thioether side chain and heterocyclic core to elucidate initial SARs between NSC-217913 and enzymes WWP1/2. The thioether side chains were targeted for ease of synthesis and which either varied the present ester functionality, introduced further polar functionalities, or were varied to hold less functionality. Heterocyclic variations were similarly chosen to change either the position or number of nitrogen atoms and C-Cl bonds for SAR development. Commercial derivatives were also tested to investigate heterocyclic cores further removed from the imidazo[*4,5-b*]pyrazine system. To this end we were able to generate a series of 27 analogues (SI Figure S1) in sufficient purity, including NSC-217913 as compound **6**, for biological analysis. The synthetic route to the most active compounds identified in this study is described here (see SI for full synthetic methods). A route to access the common 5,6-dichloroimidazo[4,5-*b*]pyrazine-2(1,3*H*)-thione heterocycle (Scheme 1, **18**) is known, but a slightly modified route is described here^30^. Compound **18** was prepared by firstly producing 2,5-diketopiperazine **II** by heating glycine **I** in ethylene glycol overnight^31^. After recrystallisation, reaction with PCl_5_ in POCl_3_ at 90 °C provided tetrachloropyrazine **III** in 32% yield^32^. Amination over two days at 120 °C provided 2,3-diamino-5,6-dichloropyrazine **IV**, along with its regioisomer, 2,6-diamino-3,5-dichloropyrazine. Reacting **IV** with thiocarbonyldiimidazole over 36 h in dioxane allows access to thione **18** in 34% yield. From here, adapted *S*-alkylation conditions with either commercial or prepared alkyl bromides (see SI for details) provided compounds **7**, **9**, **11**, **13** and NSC-217913 (**6**) in 24 − 76%^30^. Simple alkaline hydrolysis conditions at room temperature allowed the hydrolysis of NSC-217913 and provided **15** in 69%^33^.

The resulting synthesised analogues were screened using the single-shot ELISA autoubiquitination assay at 1 mM compound concentration (1% DMSO) before IC_50_ values were calculated for hit compounds (SI Figure 2–4). Preliminary hits showing signs of dose-dependency were also counter-screened, leading to compounds **18** and **25** being omitted due to significant Uba1 interactions (SI Table S7). The results from the IC_50_ ELISA autoubiquitination assays are shown in Table 1.

**Figure 4.**
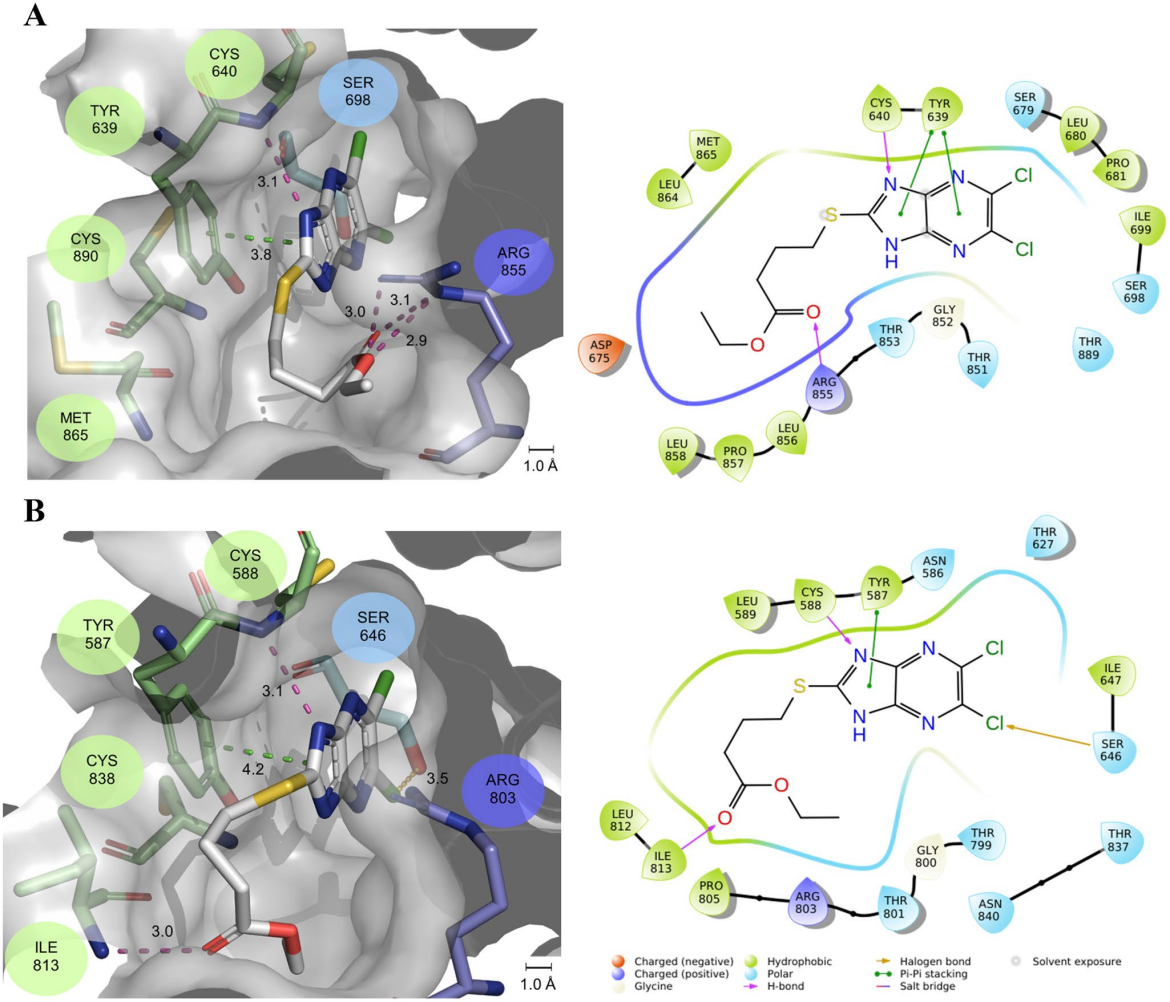
2D and 3D ligand poses of compound **11** docked to (A) WWP1-2L34H and (B) WWP2-LH. Key interacting residues are represented as sticks and coloured associated with their hydrophilic (blue), hydrophobic (green) or positively (purple) and negative (orange) charged characteristics, with the binding pocket surface (grey) also shown. Interactions including π-π stacking (green), hydrogen bonding (pink), halogen bonding (orange) and electrostatic (red) are given in angstroms (Å). 2D and 3D images were created using the Maestro Schrodinger Suite and PYMOL, respectively.

Compound **11** provided the best observed activity for WWP1 IC_50_ of 32.74 µM (95% CI = 24.6, 44.3 µM) and WWP2 IC_50_ of 269.2 µM (95% CI = 209.4, 347.9 µM), an improvement over NSC-217913. This improvement was also observed from the nitrile derivative **7** providing improved activity for WWP1 IC_50_ of 97.29 µM (95% CI = 77.7, 128.1 µM) but only limited inhibition against WWP2. The NSC-217913 hydrolysed variant **15**, was shown to provide worse activity against WWP1 than NSC-217913 but was identified as inhibitory against WWP2, although only weakly. Interestingly, compounds **9** and **13** demonstrate improved activity against WWP2 however failed to reach the activity threshold during the WWP1 single-shot assay. Generally, the activity against WWP1 is higher than against WWP2 and the inclusion of polar functionalities on the thioether chain is seemingly required for activity. A tentative trend between NSC-217913 and **11** shows improved inhibitory activity when increasing the ethyl ester chain length. When comparing to the heterocyclic variants **1** – **5** and **19** – **27** also synthesised in this project, it seems that the common dichlorinated heterocycle present in Table 1 is required for higher activity against WWP2. Compound **5**, containing the 5-chloroimidazo[4,5-*b*]pyrazine core, **4** containing 6,8-dichloroimidazo[1,2-*a*]pyrazine core, **2** with the less chlorinated 6-chloroimidazo[1,2-*a*]pyrazine core and **26** with the 5-bromothiazolo[4,5-]pyrazine system also provide some decrease in activity during the single-shot assays against WWP2. Compared to these, the non-halogenated heterocycles effectively did not. However, against WWP1 the picture is less clear with compounds **20**, **21** and **23** also reducing relative activity in the single-shot autoubiquitination.

Several other compounds (**8**, **10** & **14**) bearing the 5,6-dichloroimidazo[4,5-*b*]pyrazine heterocycle provided a rather small decrease in relative activity of both WWP1 and WWP2 in the single-shot assay. The thioether chains on **8** and **10** were rather undecorated, and whilst **14** was larger and more functionalised, it bears a bulky O*^t^*Bu group which may have obstructed binding. Compared to these, **12** and **16**, which contain ethyl acetamidoacetate or acetamido-ethanoic acid thioether chains respectively, provided better single-shot data against both WWP1 and WWP2. This along with the data obtained from the IC_50_ ELISA autoubiquitination assays points towards more hydrophilic interactions being preferred on linear thioether groups.

Further work surrounding the ethyl butyrate side chain, along with further investigation of the imidazo[4,5-*b*]pyrazine core and potential functionalisation of the chlorine atoms should lead to more interesting and potentially selective inhibitors of WWP1 and WWP2.

### Molecular docking of NSC-217913 analogues

Our initial objective was to determine X-ray crystal structures of enzyme-bound inhibitors by crystal soaking or co-crystallisation. However, these studies proved inconclusive due to a loss of resolution of diffraction from the resulting crystals exposed to DMSO. However, we were able to solve and refine structures for the apo-proteins (SI Figure S5) which were improvements on previously published data. The available crystal structures for WWP1 (PDB ID: 6J1X^20^) and WWP2 (PDB ID: 5TJ8^19^) have missing domains and/or high R_free_ values. The WWP1-2L34H crystal structure was solved at a resolution of 3.17 Å in the previously unreported space group C2 with two molecules in the asymmetric unit. Interestingly, this is the most complete structure of WWP1 solved to date revealing a structured WW2 domain. The WWP2-LH crystal structure was solved at a resolution of 2.05 Å in the same space group as that reported in the literature. This structure is higher in resolution and has a lower Rfree than PDB:5TJ8. Unfortunately, however, the WW2 domain was still disordered and therefore missing from the structure.

Being unable to solve structures of their complexes with WWP1/2, molecular docking was undertaken against the hit analogues (NSC-217913/**6**, **7**, **9**, **11**, **13** and **15**) to gain a better understanding of the SAR of NSC-217913. The grid boxes to perform the docking simulations were generated in cavities on the HECT domain surrounding the active site cysteines 890 (WWP1) and 838 (WWP2) given the ability of NSC-217913 to interfere with ubiquitination. This location has also been previously suggested as a binding site for the WWP2 small-molecule inhibitor NSC-288387^18^. The ligand poses corresponding to the best docking scores for compound **11** are presented in Figure 4 for WWP1-2L34H and WWP2-LH. The docking simulation provided a rank score of **11** against WWP1-2L34H of −8.221 (3^rd^ in rank order) and WWP2-LH of −8.893 (1^st^ in rank order). Additionally, the estimated binding free energies of **11** are −8.641 kcal/mol and −9.876 kcal/mol for WWP1-2L34H and WWP2-LH, respectively (see SI Table S9, supporting information for comparison against the other compounds modelled). The estimated free energy of binding predicts compound **11** as the second best against WWP1-2L34H and best within the compounds under investigation against WWP2-LH.

The ligand poses of compound **11** in both WWP1-2L34H and WWP2-LH are somewhat similar due to the similarities of the proposed binding site between these two enzymes (Figure 4). These poses show the imidazo[4,5-*b*]pyrazine moiety engaging in π-π interactions with the phenolic side chain of Tyr639^WWP1-2L34H^ and Tyr587^WWP2-LH^ alongside the Cys640^WWP1-2L34H^ and Cys588^WWP2-LH^ backbone amides participating in hydrogen bonding interactions with the imidazole nitrogen. However, the ethyl butyrate thioether chain interactions differ significantly between the two enzymes, most likely associated with the residue mutation at M865/I813. When docked against WWP1-2L34H, the side chain of **11** orients itself so as to interact with the guanidium ion of Arg855, orientating compound **11** in closer contact with Tyr639, allowing π-π interactions with both rings of the imidazo[4,5-*b*]pyrazine core (Figure 4, A). Against WWP2-LH, the butyrate chain has multiple hydrophobic contacts with the aromatic ring of Tyr587 and the alkyl side chain of Ile813, with the backbone amide from Ile813 also undergoing hydrogen bonding with the carbonyl oxygen of the ester. Lastly, the side chain of Ser646, located at the rear of the cavity, forms a halogen bond with one of the heterocyclic chlorides (Figure 4, B). As the heterocycle is oriented very similarly in the predicted complexes with both WWP1-2L34H and WWP2-LH, it may be that the increased π-π interactions and ion-dipole interactions between the ester and guanidinium functionalities of **11** docked to WWP1-2L34H (Figure 4, A) provide the additional strength of binding observed in the IC_50_ values. Against WWP2-LH, the ester is participating in weaker hydrogen bonding interactions against Ile813. Despite this, compound **11** demonstrated an improved free energy of binding against WWP2-LH. Such differences may have been due to the limitations of the modelling strategy having an inflexible receptor site, thus preventing the orientation of Ser698 in WWP1-2L34H from forming an equivalent halogen bond with the heterocyclic chloride. Comparing the other hit compounds, the heterocyclic portion of all molecules (SI Figure S6 and S7) are also in very similar orientations, and all pick up at least the same π-π interactions with the tyrosine phenolic side chains and hydrogen bonding interactions with the Cys residues. However, depending on the thioether identity, interactions with either the arginine residue (R855/R803) (right of the compound in the image), methionine (M865) or isoleucine (I813) (left of the compound in the image) occur (Figures S6 and S7). Interestingly, all compounds modelled against WWP2-LH also participate in halogen bonding between Ser646 and the same heterocyclic chloride.

## Conclusions

In summary, our screening efforts have resulted in the identification of a novel heterocyclic scaffold discovered from NSC-217913, capable of inhibiting both WWP1 and WWP2 into the low to mid µM range through various functionalities. Molecular docking to active site models based on improved X-ray crystal structures identified a possible binding site close to the active site cysteine residues. These predictions are supported by our SAR findings with compound **11** showing the most significant inhibition attributed to a conserved π- π interaction and increased length of the ethyl ester enabling strong ion-dipole interactions. Given the therapeutic potential of WWP1 and WWP2, this heterocyclic imidazo[4,5-*b*]pyrazine scaffold inhibitor scaffold may offer future development into a lead compound. Such work should focus on the ethyl butyrate side chain and the dichloro-substituted imidazo[4,5-*b*]pyrazine core with the aim of increasing both potency and selectivity between WWP1 and WWP2.

## Supplementary Material

Supplemental Material

## Data Availability

The crystal structures of WWP1 and WWP2 are openly available in the RCSB Protein Data Bank with accession codes 9EQK (https://doi.org/10.2210/pdb9EQK/pdb) and 9EQH (https://doi.org/10.2210/pdb9EQH/pdb), respectively. The authors confirm that further data supporting the findings of this study are available within the article and its supplementary materials.
