# Supplementary material for: Expanding the inhibitor space of the WWP1 and WWP2 HECT E3 ligases
Source: J Enzyme Inhib Med Chem. 2024 Sep 2;39(1):2394895. doi: 10.1080/14756366.2024.2394895 (PMC11373361; doi:10.1080/14756366.2024.2394895)
Supplement: Supplemental Material [file IENZ_A_2394895_SM2518.pdf]

Supporting information

Expanding the Inhibitor Space of the WWP1 and WWP2 HECT E3 Ligases

Ashley P. Dudey[a], Jake M. Rigby[b], Gregory R. Hughes[a], G. Richard Stephenson[b], Thomas E. Storr[b], Andrew Chantry[a], and Andrew M. Hemmings[a,b,c]*

[a]School of Biological Sciences, University of East Anglia, Norwich, U.K; [b]School of Chemistry, Pharmacy and Pharmacology, University of East Anglia, Norwich, U.K; [c]International Research Center for Food and Health, College of Food Science and Technology, Shanghai Ocean University, Shanghai, China.

E-mail: a.hemmings@uea.ac.uk

Contents

Supporting Information for Results and Discussion **6**
High-throughput Screen of NCI Diversity Set VI 6
Discovery of Small Molecule Inhibitor NSC-217913 8
Synthesis and Activity of NSC-217913 and Analogue Library 10
Molecular Docking of NSC-217913 Analogues 14
Biological Experimental Section **21**
Truncated Construct Arrangement 21
DNA Techniques 21
Protein Purification Techniques 22
Protein Expression 22
Protein Purification 23
SDS-PAGE Analysis 26
ELISA Autoubiquitination Assay 31
Chemical Experimental Section **32**
^{1}H NMR and HRMS data for purity analysis of NCI NSC217913 sample 34
General procedure 1: Ester hydrolysis 36
General procedure 2: Thione formation 37
General procedure 3: S-alkylation of heterocyclic thiones 38

Heterocyclic Synthesis 39
Strategy 1: 2-amino-4-chloropyrazine, 2-amino-3-chloropyrazine & 2-amino-3,5-dichloropyrazine 39
Strategy 1_1: 2,3-diamino-5-choropyrazine 45
Strategy 1_1_: 15-chloroimidazo[4,5-b]pyrazine-2(1,3H)-thione 47
Strategy 1_1_2: 2,3-diaminopyrazine 50
Strategy 1_1_2_1: Imidazo[4,5-b]pyrazine-2(1,3H)-thione 52
Strategy 2: 4,5-diamino-6-chloropyrimidine 54
Strategy 2_1: 6-Chloroimidazo[4,5-d]pyrimidine-8(7,9H)-thione 56
Strategy 3: Imidazo[4,5-b]pyridine-2(1,3H)-thione 58
Strategy 4: Imidazo[4,5-d]pyrimidine-8(7,9H)-thione 60
Strategy 5: Imidazo[4,5-c]pyridine-2(1,3H)-thione 62
Strategy 6: 2,6-diketopiperazine 64
Strategy 6_1: Tetrachloropyrazine 66
Strategy 6_1_1: 2,3-diamino-5,6-dichloropyrazine 69
Strategy 6_1_1_1: 5,6-dichloroimidazo[4,5-b]pyrazine-2(1,3H)-thione 74
Functionality Synthesis 77
N-(bromoacetyl)morpholine for **compound 9** 77
1-bromopentan-2-one for **compound 10** 79
N-(bromoacetyl)glycine ethyl ester for **compound 12** 81
(+)-(2S)-methyl 2-(2-bromoacetylamino)-3-(tert-butoxy)propanoate for **compound 14** ... 83
Methyl (3-hydroxy)-2(S)-({5,6-dichloro-1H-imidazo[4,5-b]pyrazine-2-yl}sulfanyl)propanioate for **compound 14** 85
Compound Synthesis 87
Compound 1 – Ethyl imidazo[1,2-a]pyrazine-2-acetate 87
Compound 2 – Ethyl 6-chloroimidazo[1,2-a]pyrazine-2-acetate 89
Compound 3 – Ethyl 2-aceto-dihydroimidazo[1,2-a]pyrazin-8(7H)-one 92
Compound 4 – Ethyl 6,8-Dichloroimidazo[1,2-a]pyrazine-2-acetate 95
Compound 5 – Ethyl 2-({5-chloro-1H-imidazo[4,5-b]pyrazine-2-yl}sulfanyl)acetate 98
Compound 6/NSC-217913 – Ethyl 2-({5,6-dichloro-1H-imidazo[4,5-b]pyrazine-2-yl}sulfanyl)-acetate 101
Compound 7 – 2-({5,6-Dichloro-1H-imidazo[4,5-b]pyrazine-2-yl}sulfanyl)acetonitrile104
Compound 8 – 2-({5,6-dichloro-1H-imidazo[4,5-b]pyrazine-2-yl}sulfanyl)pentane 107
Compound 9 – 2-({5,6-dichloro-1H-imidazo[4,5-b]pyrazine-2-yl}sulfanyl)acetylmorpholine 110
Compound 10 – 2-({5,6-dichloro-1H-imidazo[4,5-b]pyrazine-2-yl}sulfanyl)pentan-2-one 113
Compound 11 – Ethyl 2-({5,6-dichloro-1H-imidazo[4,5-b]pyrazine-2-yl}sulfanyl)butyrate 116

Compound 12 – Ethyl 2-({5,6-dichloro-1H-imidazo[4,5-b]pyrazine-2-yl}sulfanyl)acetamidoacetate 119

Compound 13 – 3-({5,6-Dichloro-1H-imidazo[4,5-b]pyrazine-2-yl}sulfanyl)propionic acid 122

Compound 14 – Methyl 3-(tert-butoxy)-2(S)-[2-({5,6-dichloro-1H-imidazo[4,5-b]pyrazine-2-yl}sulfanyl)acetamido]propionate 125

Compound 15 – ({5,6-dichloro-1H-imidazo[4,5-b]pyrazine-2-yl}sulfanyl)acetic acid .. 128

Compound 16 – 2-({5,6-dichloro-1H-imidazo[4,5-b]pyrazine-2-yl}sulfanyl)acetamidoacetic acid 131

Compound 17 – 3-hydroxy-2(S)-[({5,6-dichloro-1H-imidazo[4,5-b]pyrazine-2-yl}sulfanyl)acetamido]propanoic acid 134

Compound 18 – 5,6-dichloroimidazo[4,5-b]pyrazine-2(1,3H)-thione 137

Compound 19 – Ethyl 2-({1H-imidazo[4,5-b]pyridine}sulfan-2-yl)acetate 140

Compound 20 – Ethyl 2-({1H-imidazo[4,5-b]pyrazine}sulfanyl-2-yl)acetate 143

Compound 21 – Ethyl 2-({9H-imidazo[4,5-d]pyrimidine}sulfan-8-yl)acetate 146

Compound 22 – Ethyl 2-({6-chloro-9H-imidazo[4,5-d]pyrimidine}sulfan-8-yl)acetate 149

Compound 23 – Ethyl 2-[({imidazo[4,5-c]pyridine}sulfan-2-yl-5N-(ethyl-2-acetate))]acetate 152

Compound 24 – Ethyl imidazo-(1H)2-sulfanyl-1-yl acetate 155

Compound 25 – 4-phenylpyrimidine-2-thiol 157

Compound 26 – 5-bromothiazolo[4,5-b]pyrazine-2-amine 158

Compound 27 – 3-(imidazo[1,2-a]pyrimidin-2-yl)propionic acid 159

References 160

List of Tables

Table S1. Summary of DSF hit compound PAINS filtering against WWP1.

Table S2. Summary of DSF hit compound PAINS filtering against WWP2.

Table S3. Aggregate risk rankings and associated descriptions from Aggregate Advisor[2].

Table S4. Dose-dependency auto-ubiquitination screen of hit NCI Div VI compounds against their respective WWP1 and WWP2 targets.

Table S5. Cross-reactivity auto-ubiquitination screen of hit NCI Div VI compounds against both WWP1 and WWP2.

Table S6. Counter auto-ubiquitination screen of hit NCI Div VI compounds against UbcH7.

Table S7. Counter auto-ubiquitination screen of NSC-217913 analogues against Uba1 and UbcH7.

Table S8. Data collection and refinement statistics for WWP1-2L34H and WWP2-LH.

Table S9. Scoring functions and estimated binding free energies of compounds discussed in the text against WWP1-2L34H and WWP2-LH, calculations performed in Cresset Flare.

Table S10. Plasmids, constructs, and their origins.

Table S11. *E. coli* cell-line and protein expression conditions.

Table S12. Proteins and their respective buffers.

List of Figures

Figure S1. Synthesised structures of compounds under investigation with associated numbering.

Figure S2. Single-shot Autoubiquitination screen of 27 NSC-217913 analogues against WWP1-L34H (top) and WWP2-FL (bottom).

Figure S3. Dose-dependent auto-ubiquitination assay of single-shot hit NSC-217913 analogues against WWP1-L34H.

Figure S4. Dose-dependent auto-ubiquitination assay of single-shot hit NSC-217913 analogues against WWP2-FL.

Figure S5. Crystal structure of WWP1-2L34H (left) and WWP2-LH (right). The HECT domain containing the N lobe (grey) and C lobe (orange) with the 2,3-linker (pink) binding across the hinge region are shown in both structures.

Figure S6. 3D and 2D ligand poses of compounds 6, 7, 9, 11, 13 and 15 docked to WWP1-2L34H.

Figure S7. 3D and 2D ligand poses of compounds 6, 7, 9, 11, 13 and 15 docked to WWP2-LH.

Figure S8. Schematic diagram of the WWP1 and WWP2 construct architecture.

Figure S9. SDS-PAGE analysis of WWP1-L34H purification.

Figure S10. SDS-PAGE analysis of WWP1-2L34H purification.

Figure S11. SDS-PAGE analysis of WWP2-LH purification.

Figure S12. SDS-PAGE analysis of Uba1 and UbcH7 purifications.

Figure S13. Schematic diagram of the WWP2 ELISA autoubiquitination assay.

Figure S14. UPLC chromatogram of NCI sample (top) vs. blank (bottom).

Figure S15. Mass spectrum for the impurity 2.

Figure S16. Proposed structures of impurity 2.

Figure S17. ^{1}H NMR spectrum of NCI-sample of NSC-217913 in DMSO-d_6.

Supporting Information for Results and Discussion

High-throughput Screen of NCI Diversity Set VI

Table S1. Summary of DSF hit compound PAINS filtering against WWP1.

NSC Number	PAINS Filter[(a)]	Other PAINS	Aggregate Risk[(c)]	Omitted
7436	-	-	Very Low	N
13151	-	-	Medium	N
13579	-	-	High	N
25435	-	-	Medium	N
30260	-	-	High	N
34865	-	-	Very Low	N
36586	-	Aggregate	Very High	Y
37219	-	-	Medium	N
37553	-	-	High	N
39984	-	-	High	N
40269	-	-	Low	N
43088	-	-	High	N
73735	-	-	High	N
85433	-	-	High	N
107582	-	-	High	N
134674	-	-	Low	N
309401	Anil_no_alk(40)	-	Very Low	Y
332670	-	-	High	N
370383	-	-	Very Low	N
374814	-	-	High	N
380279	-	-	Very Low	N
522131	-	Radical Quencher[(b)]	High	Y
637578	-	-	Medium	N
661221	Quinone(370)	-	Medium	Y

[(a)] ZINC15 Filter[1]. [(b)] Ranking based on descriptions from Aggregate Advisor (Table S3)[2]. [(c)] Auto-ubiquitination assay-specific PAINS[3]

Table S2. Summary of DSF hit compound PAINS filtering against WWP2.

NSC Number	PAINS Filter[(a)]	Other PAINS	Aggregate Risk[(b)]	Omitted
9037	Catechol_A(92)	-	High	Y
20045	-	-	High	N
26112	-	-	Medium	N
34769	-	-	Very Low	N
46492	-	-	High	N
49652	-	Reactive	High	Y
50651	-	-	High	N
50654	-	-	High	N
57103	-	-	High	N
57624	-	-	High	N
136513	-	-	High	N
154295	-	-	Very Low	N
156565	Quinone(370), Anthranil_one_A(38)	-	Very Low	Y
159566	Quinone(370)	-	Low	Y
217913	-	-	Very Low	N
305780	-	Impurity	High	Y
319424	-	-	High	N
319994	-	-	High	N
320218	-	-	High	N
329249	-	-	High	N
367428	-	-	Very Low	N
375981	-	-	High	N
522131	-	-	High	N
661221	Quinone(370)	-	Medium	Y

[(a)] ZINC15 Filter[1]. [(b)] Ranking based on descriptions from Aggregate Advisor (Table S6)[2].

Table S3. Aggregate risk rankings and associated descriptions from Aggregate Advisor[2].

Risk	Descriptions from Aggregate Advisor
Very Low	This molecule does not look like one that has been previously observed to aggregate.
Low	This molecule is not similar to a known aggregator, but with a high logP, is a candidate for aggregation.
Medium	This molecule is somewhat similar to a molecule that has been previously observed to aggregate, AND, with a high logP, is suspicious.
High	This molecule is very similar to a molecule that has been previously observed to aggregate.
Very High	This molecule has previously been observed to aggregate.

Discovery of Small Molecule Inhibitor NSC-217913

Table S4. Dose-dependency auto-ubiquitination screen of hit NCI Div VI compounds against their respective WWP1 and WWP2 targets.

NSC Number	Structure	WWP1 IC50 (μM)
13151[a]	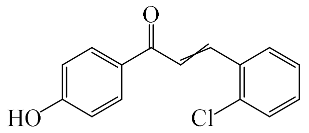	396.9
73735	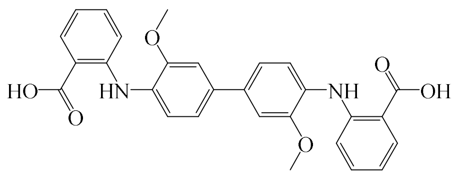	19.21
85433	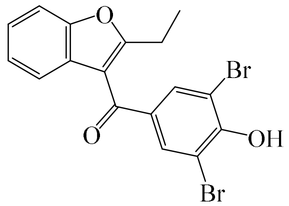	92.41
637578	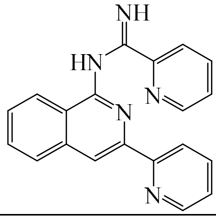	N/A

NSC Number	Structure	WWP2 IC50 (μM)
26112[a]	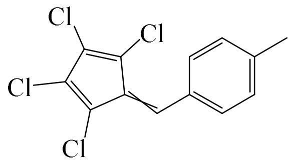	N/A
57103	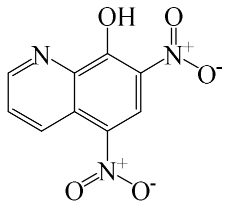	513.0
217913	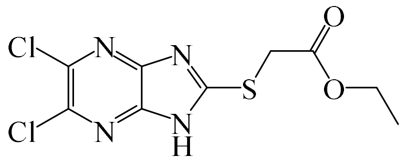	69.8

[a]mixture of stereoisomers

Table S5. Cross-reactivity auto-ubiquitination screen of hit NCI Div VI compounds against both WWP1 and WWP2.

NSC Number	Structure	WWP1 IC50 (μM)	WWP2 IC50 (μM)
73735	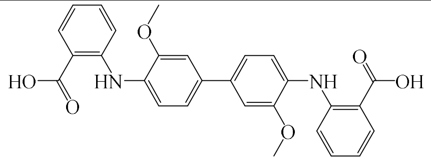	19.2	49.1
85433	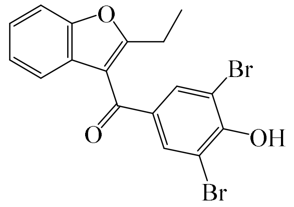	92.4	256.4
217913	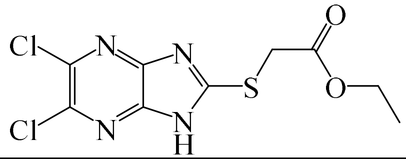	33.3	69.8

Table S6. Counter auto-ubiquitination screen of hit NCI Div VI compounds against UbcH7.

NSC Number	Structure	UbcH7 Activity (%)
73735[a]	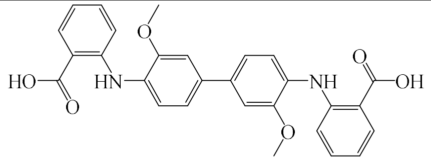	62.5 ± 2.4
85433	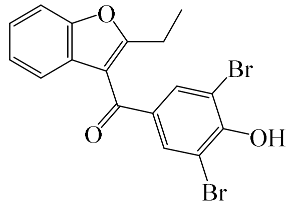	7.5 ± 0.6
217913[a]	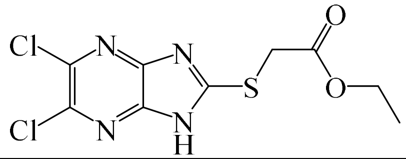	92.2 ± 1.1

[a]synthesised

Synthesis and Activity of NSC-217913 and Analogue Library

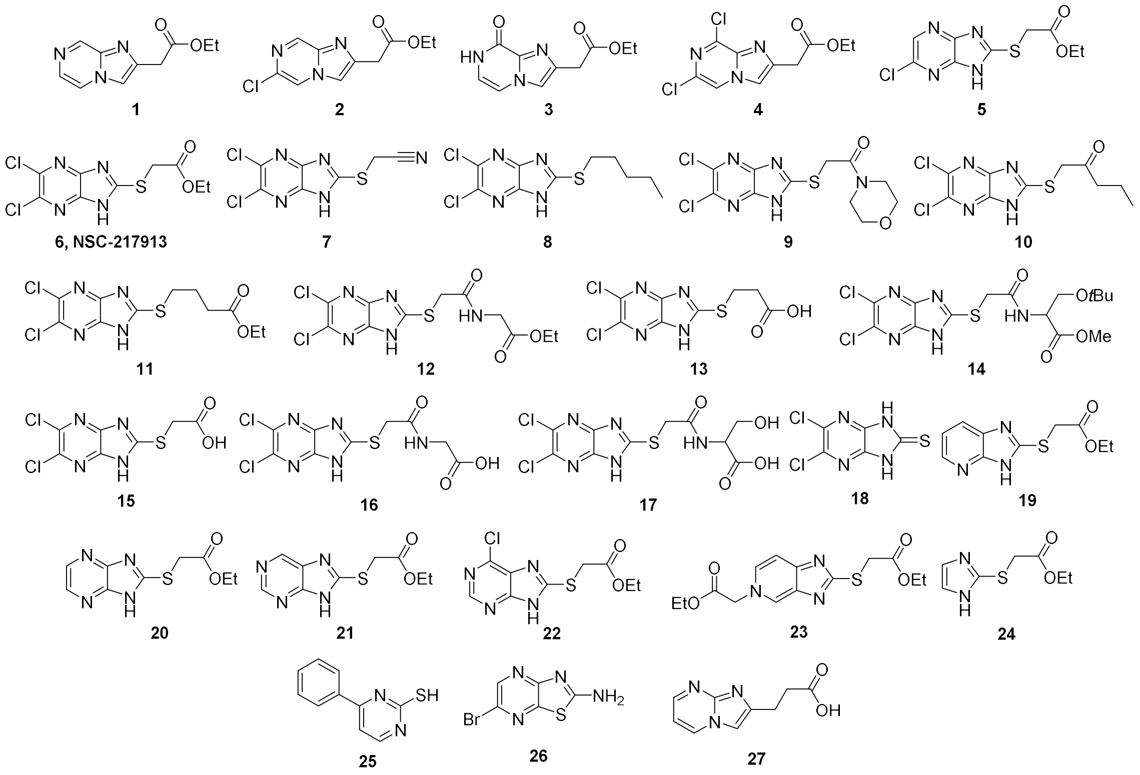

Figure S1. Synthesised structures of compounds under investigation with associated numbering. Images created in Chemdraw.

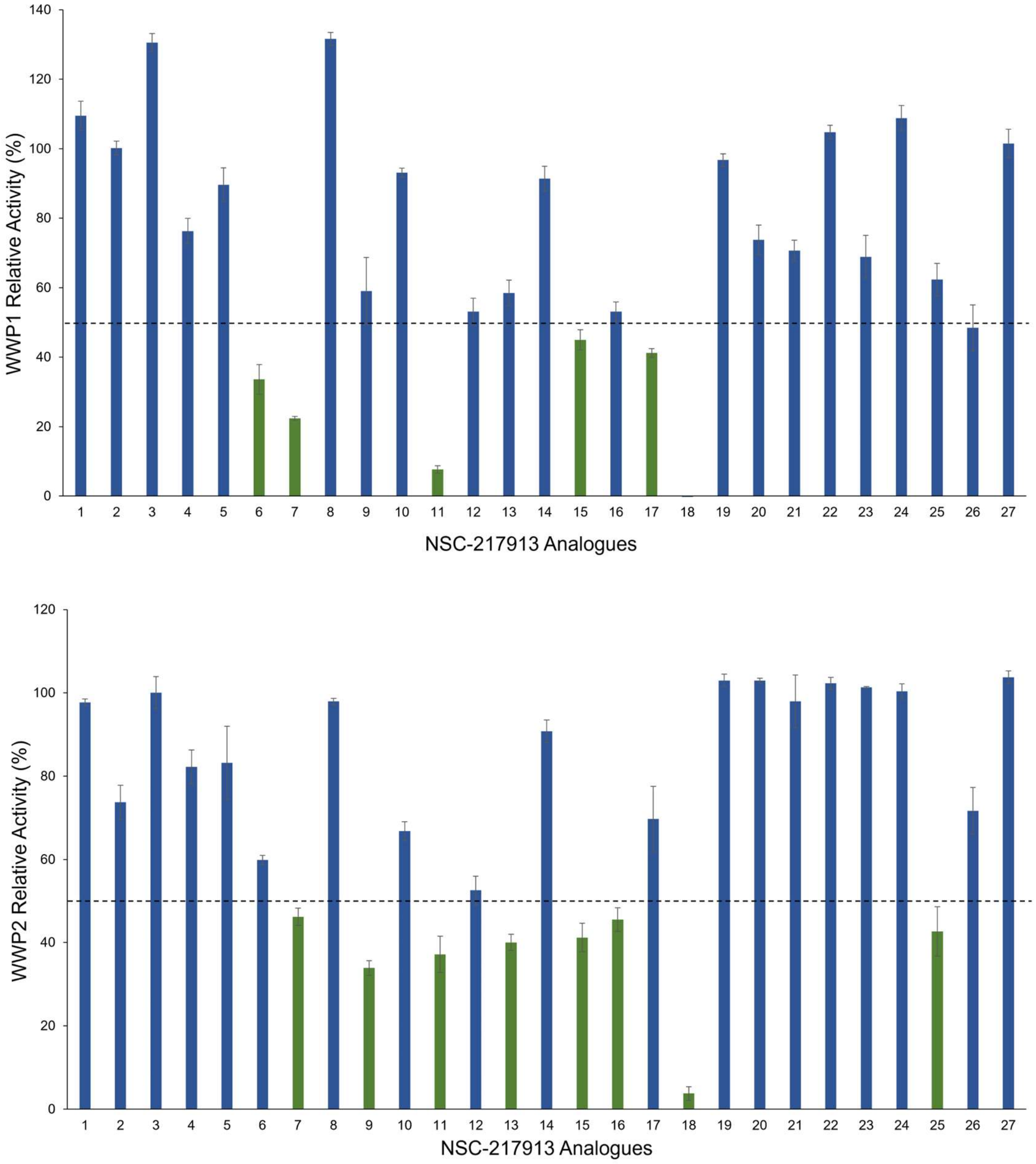

Figure S2. Single-shot Autoubiquitination screen of 27 NSC-217913 analogues against WWP1-L34H (top) and WWP2-FL (bottom). Compound inhibition was measured at 1 mM (1 % DMSO), with a hit threshold (green) of less than 50 % relative activity (dashed line), normalised to their respective 0 % and 100 % WWP1-L34H and WWP2-FL controls.

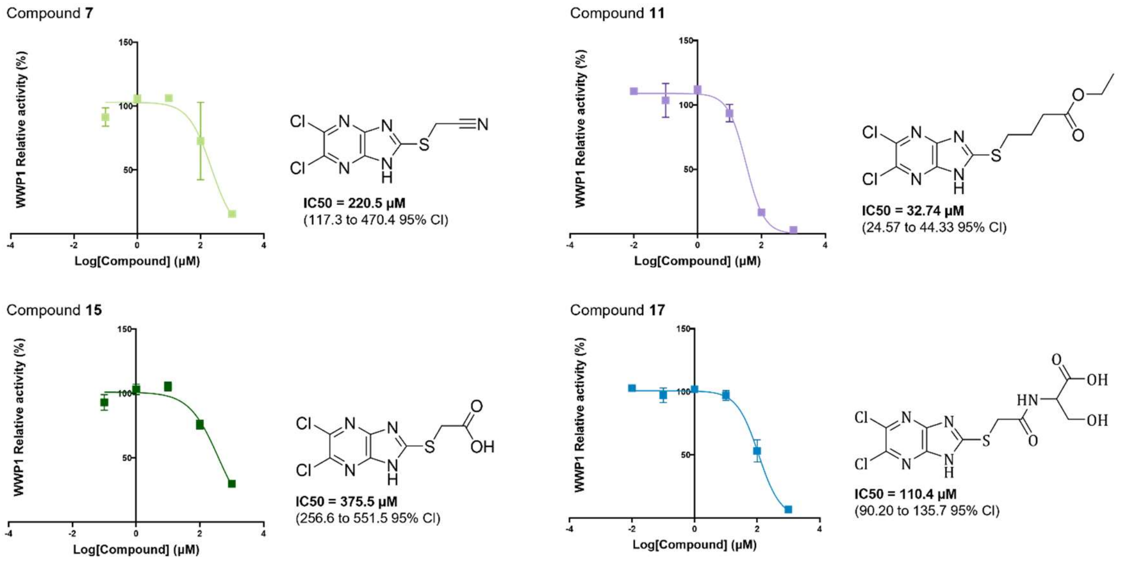

Figure S3. Dose-dependent auto-ubiquitination assay of single-shot hit NSC-217913 analogues against WWP1-L34H. Compound inhibition was measured on a log scale from 1000 – 10 nM (1 % DMSO), normalised to 0 % and 100 % controls. IC50 values were calculated from non-linear regression curves fitted in GraphPad software. The 95 % confidence interval is given in brackets.

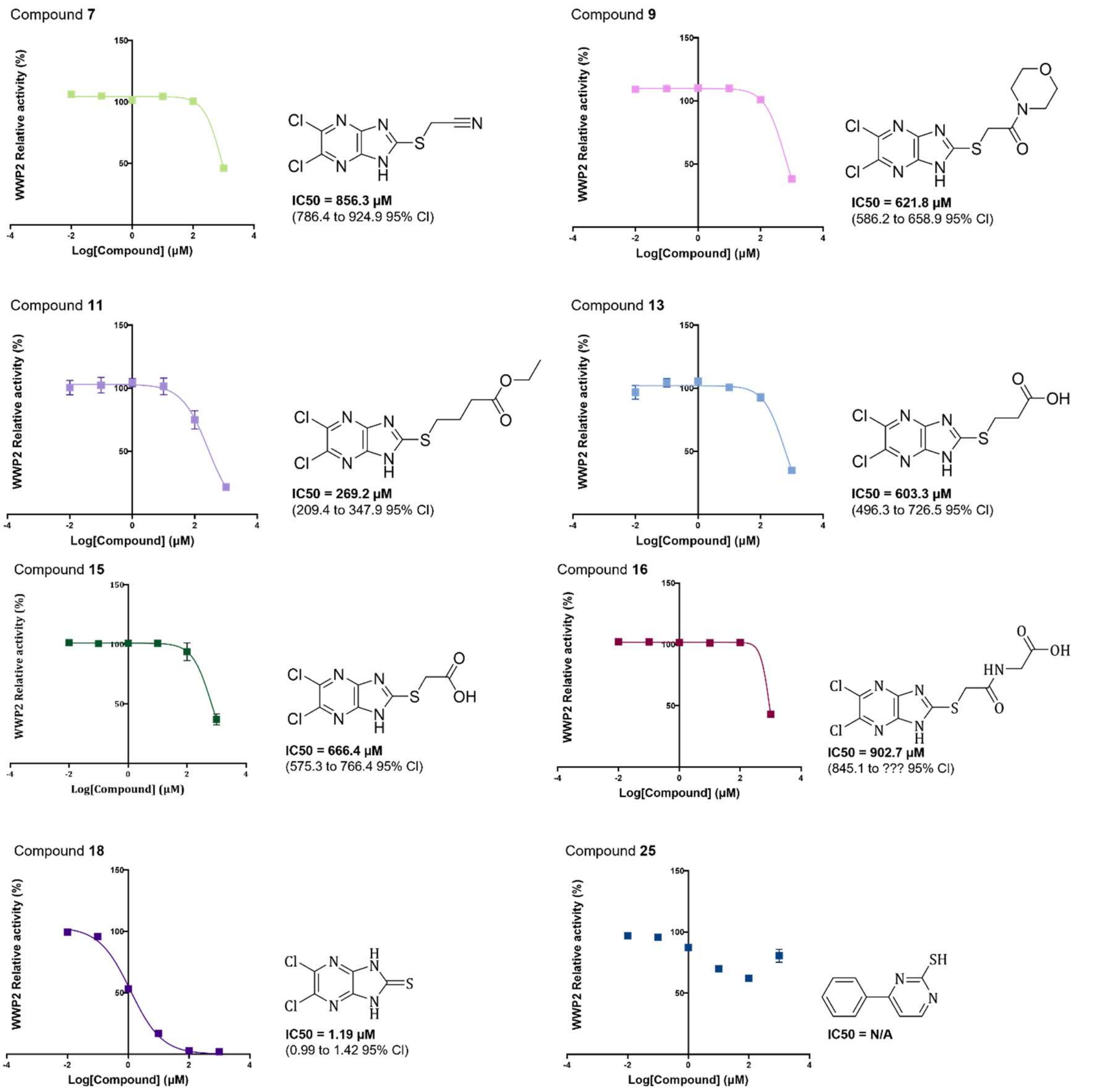

Figure S4. Dose-dependent auto-ubiquitination assay of single-shot hit NSC-217913 analogues against WWP2-FL. Compound inhibition was measured on a log scale from 1000 – 10 nM (1 % DMSO), normalised to 0 % and 100 % controls. IC50 values were calculated from non-linear regression curves fitted in GraphPad software. The 95 % confidence interval is given in brackets.

Table S7. Counter auto-ubiquitination screen of NSC-217913 analogues against Uba1 and UbcH7.

Compound	Uba1 Activity (%)	UbcH7 Activity (%)
**7**	-	94.2 ± 0.8
**11**	-	88.8 ± 0.2
**15**	-	93.0 ± 1.0
**17**	-	88.8 ± 0.7
**18**	22.1 ± 4.1	-
**25**	59.2 ± 7.4	-

Molecular Docking of NSC-217913 Analogues

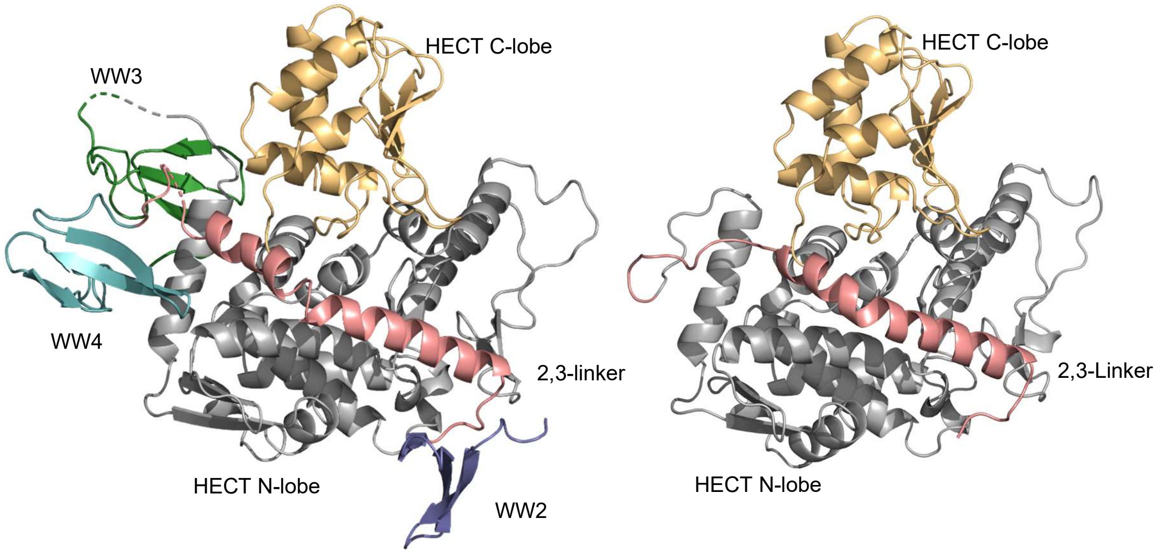

Figure S5. Crystal structure of WWP1-2L34H (left) and WWP2-LH (right). The HECT domain containing the N lobe (grey) and C lobe (orange) with the 2,3-linker (pink) binding across the hinge region are shown in both structures. WWP1-2L34H also contains the WW2 (purple) domain missing from the WWP2-LH structure, as well as the WW3 (blue) and WW4 (green) domains. WWP1-2L34H structure shown is chain B, having two molecules in the asymmetric unit, chain A WW3 and WW4 are more disordered and are conformationally different.

Table S8. Data collection and refinement statistics for WWP1-2L34H and WWP2-LH.

	**WWP1-2L34H**	**WWP2-LH**
**Wavelength/ Å**	0.9763	0.9795
**Resolution range**	84.49 - 3.0 (3.11 - 3.00)	45.15 - 2.06 (2.134 - 2.06)
**Space group**	C 1 2 1	P 21 21 21
**Unit cell**	227.0 59.7 108.3 90 99.4 90	44.03 90.31 111.25 90 90 90
**Total reflections**	201784 (19744)	329940 (30499)
**Unique reflections**	29068 (2805)	28211 (2767)
**Multiplicity**	6.9 (7.0)	11.7 (11.0)
**Completeness (%)**	99.66 (97.87)	99.91 (99.96)
**Mean I/sigma(I)**	5.36 (0.74)	6.18 (0.98)
**Wilson B-factor**	66.38	36.38
**R-merge**	0.317 (1.895)	0.3231 (3.378)
**R-meas**	0.3427 (2.047)	0.3386 (3.543)
**R-pim**	0.1292 (0.7681)	0.09963 (1.059)
**CC1/2**	0.981 (0.469)	0.992 (0.38)
**CC***	0.995 (0.799)	0.998 (0.742)
**Reflections used in refinement**	29029 (2800)	28206 (2766)
**Reflections used for R-free**	1492 (138)	1390 (130)
**R-work**	0.2038 (0.3358)	0.2165 (0.3183)
**R-free**	0.2692 (0.3974)	0.2655 (0.3489)
**CC(work)**	0.956 (0.677)	0.936 (0.699)
**CC(free)**	0.931 (0.517)	0.912 (0.640)
**Number of non-hydrogen atoms**	8686	3642
**macromolecules**	8667	3524
**ligands**	3	29
**solvent**	16	105
**Protein residues**	1056	422
**RMS(bonds)**	0.011	0.015
**RMS(angles)**	1.31	1.36
**Ramachandran favored (%)**	88.37	91.43
**Ramachandran allowed (%)**	9.71	7.86
**Ramachandran outliers (%)**	1.92	0.71
**Rotamer outliers (%)**	3.91	2.65
**Clashscore**	12.22	10.37
**Average B-factor**	78.36	53.78
**macromolecules**	78.40	54.00
**ligands**	56.04	62.83
**solvent**	63.89	45.31
**Number of TLS groups**	11	1

Table S9. Scoring functions and estimated binding free energies of compounds discussed in the text against WWP1-2L34H and WWP2-LH, calculations performed in Cresset Flare.

**Compound**	**Structure**	**WWP1-2L34H**		**WWP2-LH**	
		Docking score	Binding energy (kcal/mol)	Docking Score	Binding energy (kcal/mol)
**6**	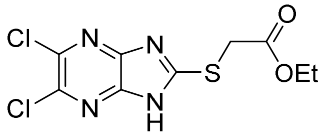	-8.259	-8.545	-8.686	-9.188
**7**	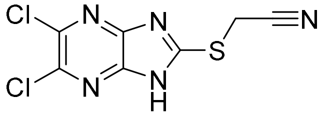	-7.732	-8.012	-8.147	-8.267
**9**	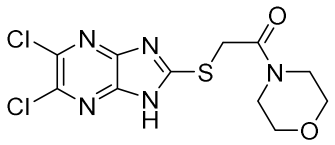	-8.801	-9.146	-8.876	-9.708
**11**	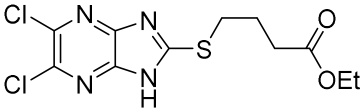	-8.221	-8.641	-8.893	-9.876
**13**	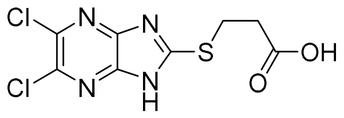	-7.504	-8.11	-7.943	-8.877
**15**	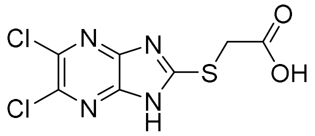	-7.566	-8.176	-7.654	-8.394

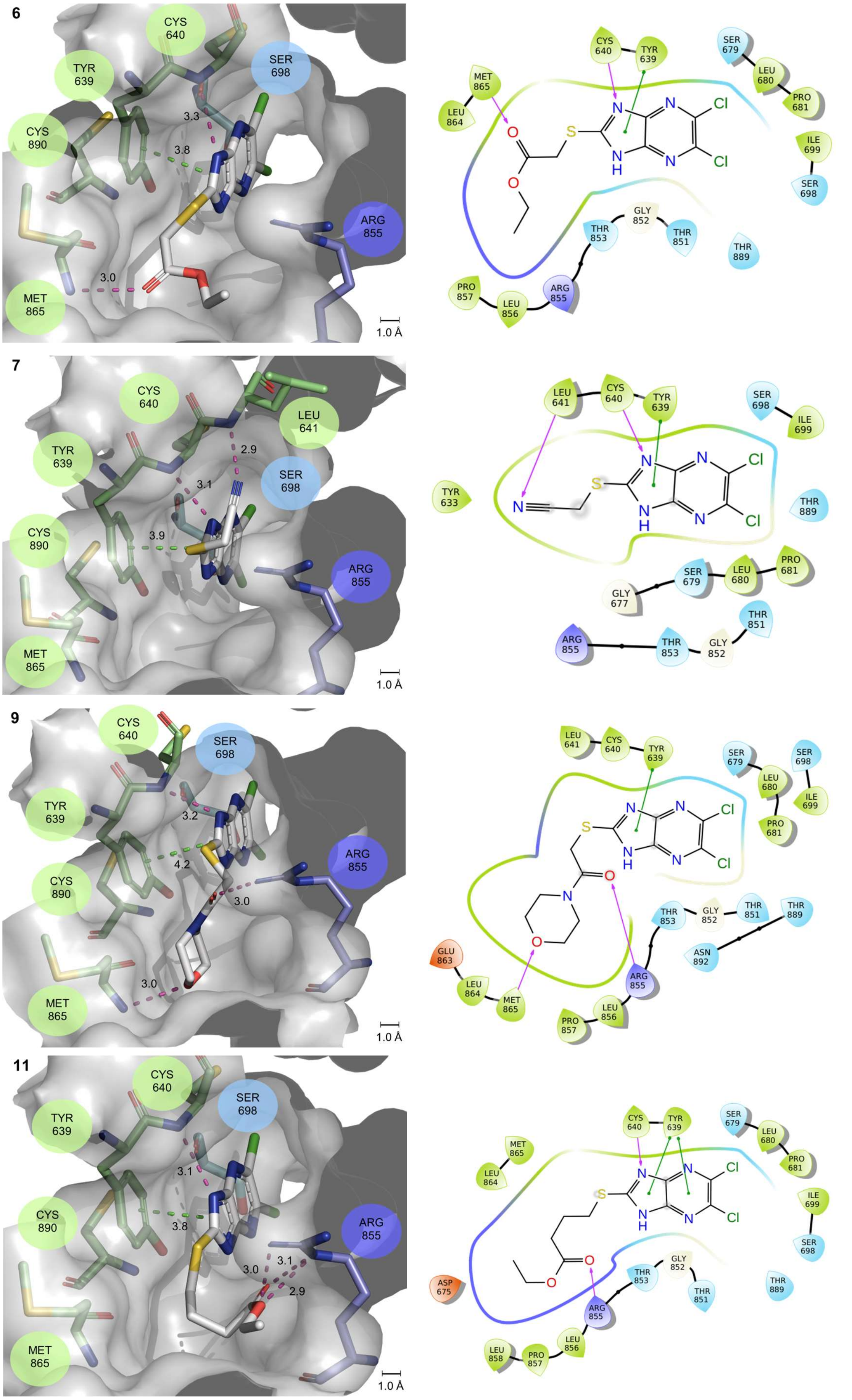

6
CYS 640
TYR 639
SER 698
CYS 890
3.3
3.8
ARG 855
3.0
MET 865
1.0 Å
CYS 640
TYR 639
SER 679
LEU 680
PRO 681
MET 865
LEU 864
ILE 699
SER 698
GLY 852
THR 853
THR 851
THR 889
PRO 857
LEU 856
ARG 855
7
CYS 640
LEU 641
TYR 639
2.9
SER 698
3.1
CYS 890
3.9
ARG 855
MET 865
1.0 Å
LEU 641
CYS 640
TYR 639
SER 698
ILE 699
TYR 633
THR 889
PRO 681
SER 679
LEU 680
GLY 677
THR 851
ARG 855
THR 853
GLY 852
9
CYS 640
SER 698
TYR 639
3.2
ARG 855
CYS 890
4.2
3.0
3.0
MET 865
1.0 Å
LEU 641
CYS 640
TYR 639
SER 679
SER 698
LEU 680
ILE 699
PRO 681
THR 853
GLY 852
THR 851
THR 889
ASN 892
GLU 863
LEU 864
MET 865
ARG 855
LEU 856
PRO 857
11
CYS 640
TYR 639
SER 698
3.1
CYS 890
3.8
ARG 855
3.0
3.1
2.9
MET 865
1.0 Å
CYS 640
TYR 639
SER 679
LEU 680
MET 865
LEU 864
PRO 681
ILE 699
SER 698
ASP 675
THR 853
GLY 852
THR 851
THR 889
ARG 855
LEU 858
PRO 857
LEU 856

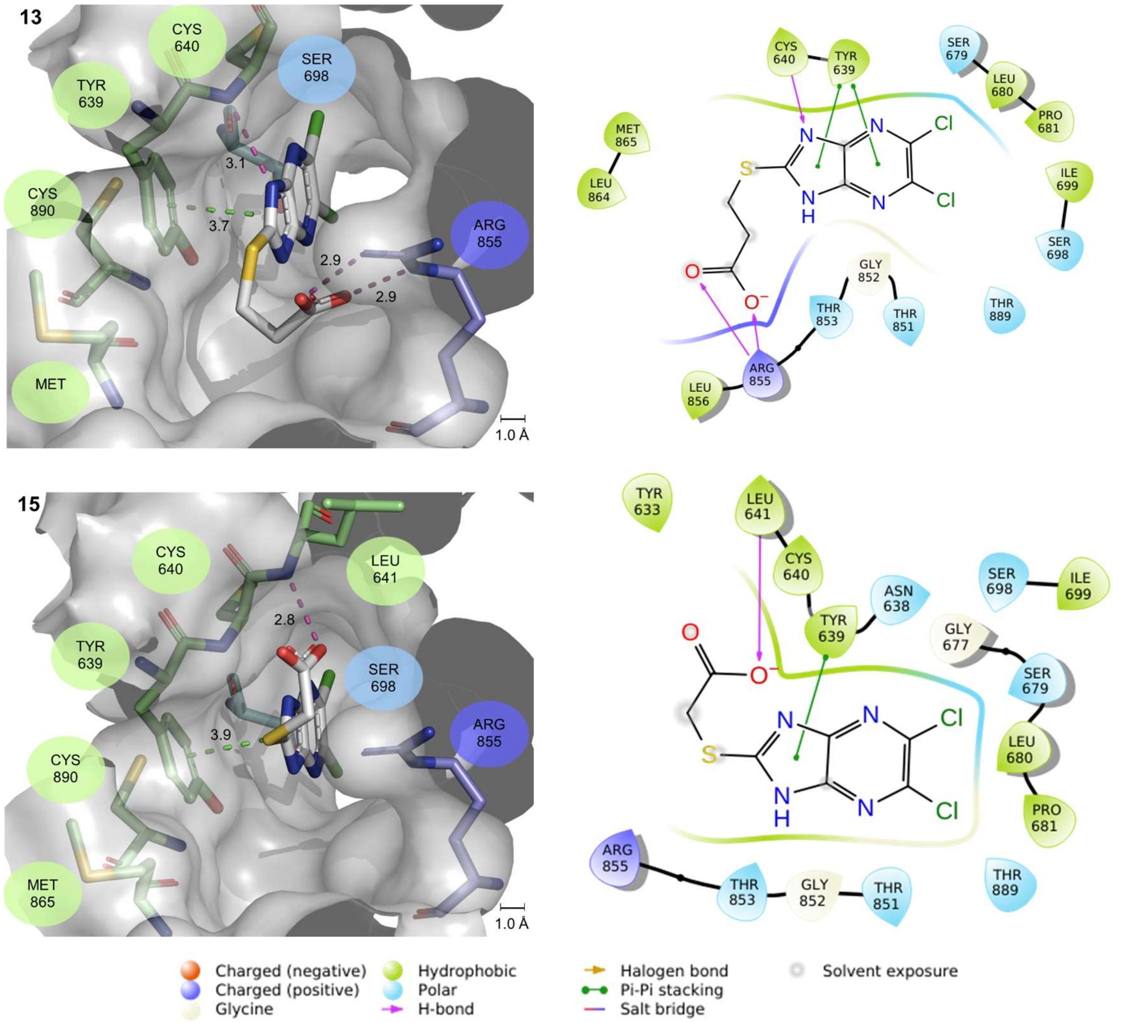

Figure S6. 3D and 2D ligand poses of compounds 6, 7, 9, 11, 13 and 15 docked to WWP1-2L34H. Key interacting residues are represented as sticks and coloured associated with their hydrophilic (blue), hydrophobic (green) or positively (purple) and negative (orange) charged characteristics, with the binding pocket surface (grey) also shown. Interactions including π-π stacking (green), hydrogen bonding (pink), halogen bonding (orange) and electrostatic (red) are given in angstroms (Å). 2D and 3D images were created using the Maestro Schrodinger Suite and PYMOL, respectively.

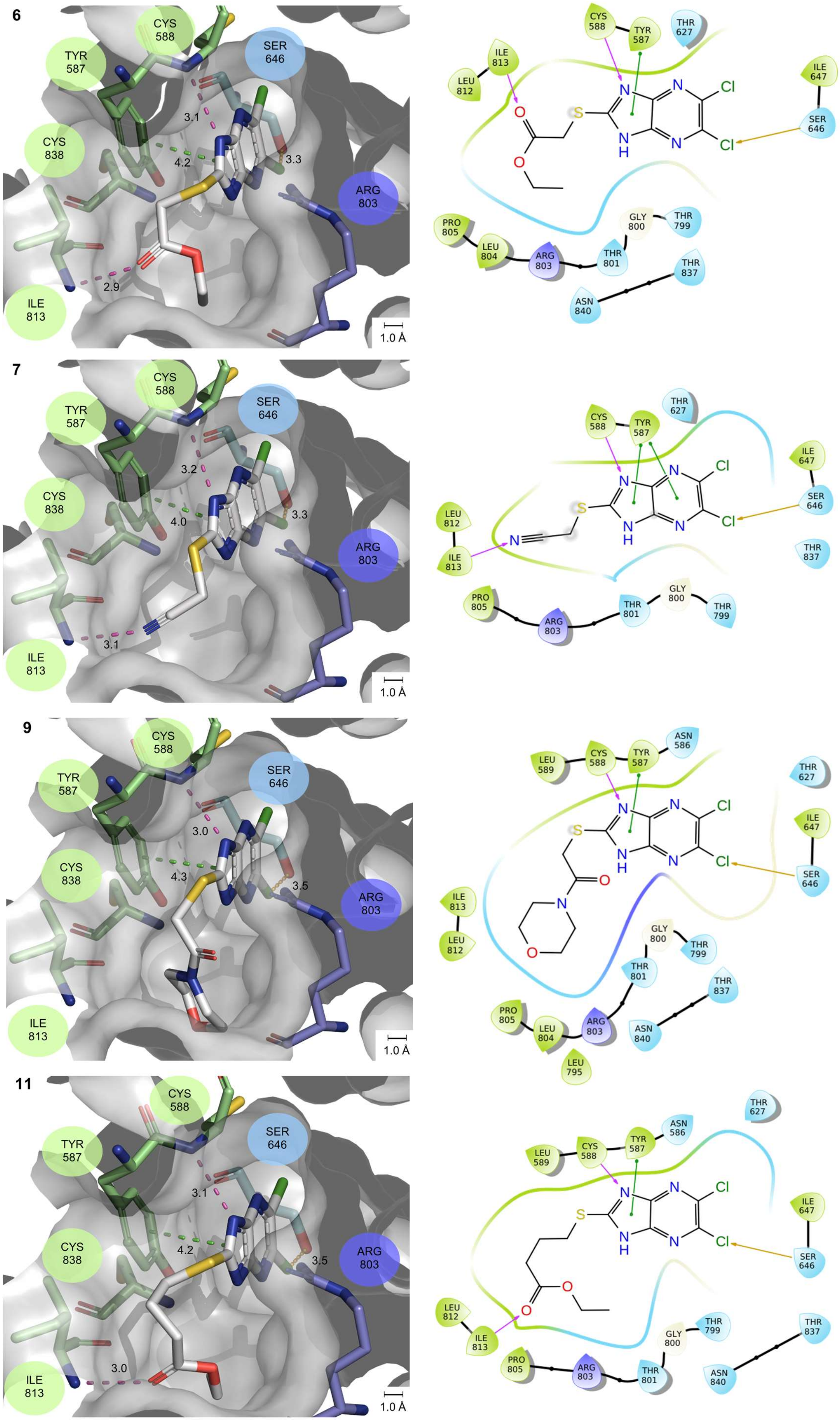
6
CYS 588
TYR 587
SER 646
CYS 838
ARG 803
ILE 813
3.1
4.2
3.3
2.9
1.0 Å
7
3.2
4.0
3.3
3.1
9
3.0
4.3
3.5
11
3.1
4.2
3.5
3.0
LEU 812
ILE 647
THR 627
PRO 805
LEU 804
GLY 800
THR 799
THR 801
THR 837
ASN 840
ASN 586
LEU 589
LEU 795
N
NH
S
Cl
O

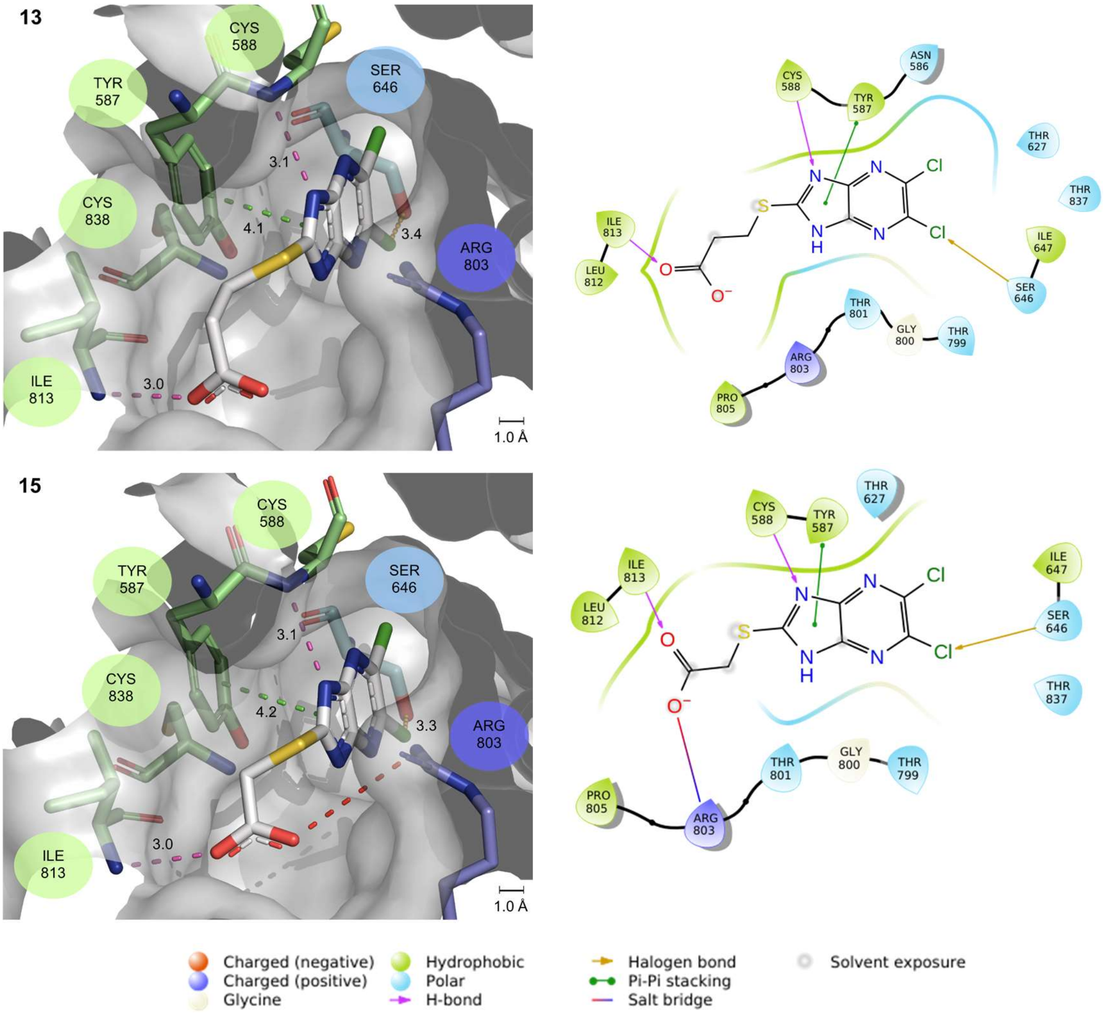

Figure S7. 3D and 2D ligand poses of compounds 6, 7, 9, 11, 13 and 15 docked to WWP2-LH. Key interacting residues are represented as sticks and coloured associated with their hydrophilic (blue), hydrophobic (green) or positively (purple) and negative (orange) charged characteristics, with the binding pocket surface (grey) also shown. Interactions including π-π stacking (green), hydrogen bonding (pink), halogen bonding (orange) and electrostatic (red) are given in angstroms (Å). 2D and 3D images were created using the Maestro Schrodinger Suite and PYMOL, respectively.

Biological Experimental Section

Truncated Construct Arrangement

The various truncated forms of both WWP1 and WWP2 as used throughout this study are illustrated below in Figure S8.

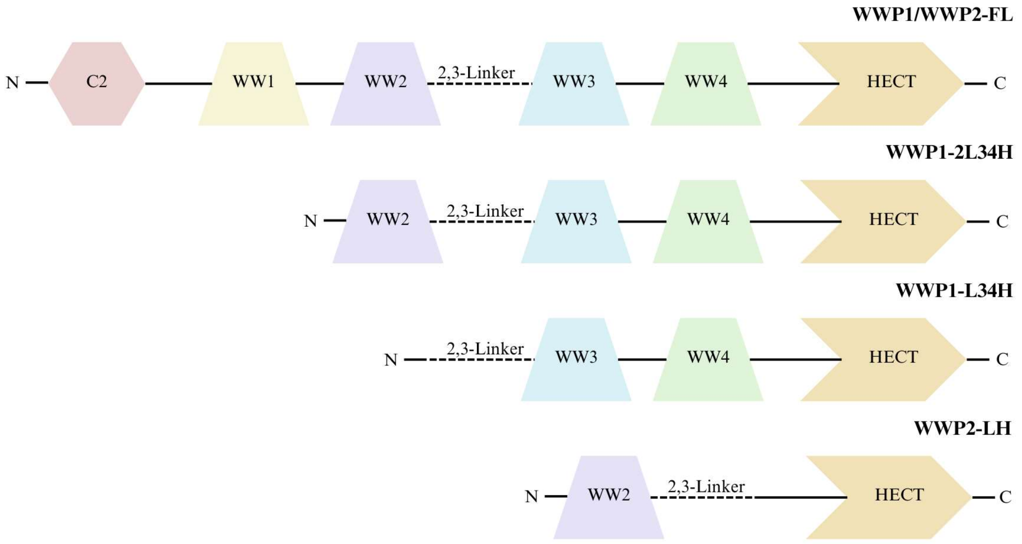

Figure S8. Schematic diagram of the WWP1 and WWP2 construct architecture. Full-length WWP2 (WWP2-FL), WWP1-WW2-2,3-linker-WW3-WW4-HECT (WWP1-2L34H), WWP1- 2,3-linker-WW3-WW4-HECT (WWP1-L34H) and WWP2-WW2-2,3-linker-HECT (WWP2-LH) are all shown, containing various arrangements of the Ca^{2+} binding (C2) (pink), four Tryptophan-Tryptophan (yellow, purple, blue and green), and active site HECT (orange) domain alongside the 2,3-linker (dashed line) region vital for autoinhibition.

DNA Techniques

Plasmids (Table S10) were transformed using standard heat shock or electroporation, incubating for 12-18 hours at 37 °C on LB agar plates containing respective antibiotics (Table S11).

Table S10. Plasmids, constructs, and their origins.

Plasmid	Construct	Origin
**pET3a**-hUba1^{His}	Wildtype	Addgene plasmid #63571[4]
**pGEX4T-1**-hUba1$^{GST\text{-}F2}$	Wildtype	Gifted by Dr Arthur L Haas's Lab
**pET3a**-UbcH7^{His}	Wildtype	Gifted by Professor Martin Scheffner
**pGEX4T-1**-UbcH7a$^{GST\text{-}F2}$	Wildtype	Gifted by Dr Arthur L Haas's Lab
**pET32a**-WWP1-2L34H$^{His\text{-}Trx\text{-}3C}$	WW2-2,3-linker-WW3-WW4-HECT	Gifted by Dr Wenyu Wen
**pET32a**-WWP1-L34H$^{His\text{-}Trx\text{-}3C}$	2,3-linker-WW3-WW4-HECT	Gifted by Dr Wenyu Wen
**pGEX2T**-WWP2-FL$^{GST\text{-}F2}$	Wildtype	Dr Andrew Chantry's Lab
**pGEX6p-2**-WWP2-LH$^{GST\text{-}3C}$	WW2-2,3-linker-HECT	Gifted by Professor Philip Cole

Protein Purification Techniques

All protocols were performed on ice or at 5 °C unless stated otherwise.

Protein Expression

Transformed E. coli cells were inoculated and incubated overnight at 37 °C, 180 rpm in LB containing appropriate antibiotics. The desired recombinant proteins were expressed, inducing with IPTG at OD^{600} 0.6 – 1.0 before incubating at protein-specific conditions (Table S11). Cells were pelleted by centrifugation (Beckman Coulter J20, JLA 8.1000 rotor) at 4,000 g, 4 °C for 30 minutes and stored at -20 °C.

Table S11. *E. coli* cell-line and protein expression conditions.

Recombinant Protein	*E. coli* cell-line	[IPTG] (mM)	Temp (°C)	Antibiotics[(a)]
**hUba1**^{His}	BL21-Star (DE3)	1.0	25	Amp
**hUba1**$^{GST\text{-}F2}$	BL21 (DE3)	1.0	25	Amp
**UbcH7**^{His}	BL21-CodonPlus RP	1.0	25	Amp, Cam
**UbcH7a**$^{GST\text{-}F2}$	BL21 (DE3)	0.4	20	Amp

**WWP1-2L34H$^{His\text{-}Trx\text{-}3C}$**	BL21-CodonPlus RP	0.5	16	Amp, Cam
**WWP1-L34H$^{His\text{-}Trx\text{-}3C}$**	BL21-CodonPlus RP	0.5	16	Amp, Cam
**WWP2-FL$^{GST\text{-}3C}$**	BL21-CodonPlus RP	0.75	30	Amp, Cam
**WWP2-LH$^{GST\text{-}3C}$**	BL21-CodonPlus RP	0.5	16	Amp, Cam

(a) 50 ug/mL Amp (Ampicillin or Carbenicillin) and Kan (Kanamycin), 34 ug/mL Cam (Chloramphenicol).

Protein Purification

The protein-specific buffers for each purification step is shown in Table S12.

General protocols: Cells were lysed by either using a 4710 series ultrasonic homogenizer CP50 (Cole-Parmer) at 50 % amp for 10 seconds on, 10 seconds off for a total of 6 minutes or french pressed at 16,000 psi using a pre-cooled pressure cell (Thermo French Press). Affinity columns (Cytiva Life Sciences) were installed onto a bench-top peristaltic pump (Parnachia Biotech) at 20 °C or AKTA pure 2 system (Cytiva Life Sciences) at 5 °C with supplier protocols followed. Sample concentrating was achieved using 5 or 10 kDa MW cut off Vivaspin protein concentrators (GE Healthcare), centrifuged (Beckman Coulter J-15R, JS-4.750 rotor) at 4,000 g for 10 to 20 minutes per spin with mixing. All samples were snap-frozen and stored at -80 °C unless otherwise stated.

His-tagged Uba1 and UbcH7: Pelleted cells from 1 L culture were resuspended in 35 mL buffer before being lysed, clarified, and purified through a 5 mL HisTrap™ FF column via straight elution. These were dialysed at 10 kDa cut-off in a 5 L reservoir overnight before concentrating and stored.

GST-tagged Uba1: Pelleted cells from 1 L culture were resuspended in 35 mL buffer before being lysed, clarified, and purified through a 1 mL GSTrap™ HP column via

straight elution. The sample was then dialysed at 3 kDa cut-off in a 2 L reservoir overnight before concentrating and stored.

GST-tagged UbcH7: Pelleted cells from 2 L culture were resuspended in 35 mL buffer before being lysed, clarified, and loaded onto a 1 mL GSTrap™ HP column. On-column cleavage was achieved by incubating with 20 units per mL of thrombin overnight. Untagged UbcH7 was collected by washing the column with 7 CV of high salt PBS (500 mM NaCl) before passing through a 1 mL HiTrap Benzamidine column (Cytiva). The sample was then concentrated and stored.

His-tagged WWP1-L34H: Pelleted cells from 8 L culture were resuspended in 70 mL buffer before being lysed, clarified, and purified through a 5 mL HisTrap™ FF column, washing for 20 CV before finally eluting stepwise at 2 CV steps. The sample was then dialysed at 10 kDa cut-off in a 5 L reservoir overnight, filtering any precipitation before spin concentrating (10 °C) to 2 mL for gel filtration on a SEC 75 pg. PreScission protease was added at 2 units per mg to the eluted fractions and incubated overnight before removing by reverse GSTrap™. Buffer exchange was performed using the HiPrep™ 26/10 Desalting column to remove EDTA, followed by a reverse IMAC. The sample was concentrated to 2 mL and a second gel filtration was carried out on the SEC 75 pg. Eluted fractions were pooled, concentrated and stored.

His-tagged WWP1-2L34H: Pelleted cells from 8 L culture were resuspended in 70 mL buffer before being lysed, clarified, and purified through a 5 mL HisTrap™ FF column, washing for 20 CV before finally gradient eluting over 10 CV. The sample was then dialysed at 10 kDa cut-off in a 5 L reservoir overnight and filtered to remove any precipitation. PreScission protease was added at 1.5 units per mg and incubated during

another round of dialysis. The protease was then removed by reverse IMAC, before the sample was concentrated to 2 mL and loaded onto SEC 200 pg. Eluted fractions were pooled and concentrated for either immediate use in crystallography or stored.

GST-tagged WWP2-LH: Pelleted cells from 4 L culture were resuspended in 35 mL buffer before being lysed, clarified, and purified through a 5 mL GSTrap™ FF column on bench top, collecting the straight elution on ice. PreScission protease was added at 1 unit per mg, and incubated during dialysed at 10 kDa cut-off in a 5 L reservoir overnight, before being refreshed for a further 4 hrs. A reverse GSTrap was used, removing protease, uncleaved WWP2-LH and free GST. The sample was concentrated to 2 mL for gel filtration on a SEC 75 pg. Eluted fractions were pooled and again passed through the reverse GSTrap before adding 5 % glycerol. Samples were concentrated at 20 °C for either immediate use in crystallography or stored.

Table S12. Proteins and their respective buffers.

Recombinant Protein	Buffer	Contents
**hUba1**^{His}	Resuspension & Wash	20 mM Na_2PO_4 pH 7.4, 500 mM NaCl, 20 mM Imidazole containing 1 x Roche tablet
	Elution	20 mM Na_2PO_4 pH 7.4, 500 mM NaCl, 500 mM Imidazole
	Dialysis	50 mM Tris.HCl pH 7.4, 1 mM DTT
**hUba1**$^{GST\text{-}F2}$	Resuspension & Wash	10 mM Na_2HPO_4 pH 7.4, 2 mM KH_2PO_4, 2.7 mM KCl, 137 mM NaCl containing 1 x Roche tablet

	Elution	10 mM Na_2HPO_4 pH 7.4, 2 mM KH_2PO_4, 2.7 mM KCl, 137 mM NaCl, 50 mM Reduced Glutathione
	Dialysis	10 mM Na_2HPO_4 pH 7.4, 2 mM KH_2PO_4, 2.7 mM KCl, 137 mM NaCl
**UbcH7**^{His}	Resuspension & Wash	20 mM Na_2PO_4 pH 7.4, 500 mM NaCl, 20 mM Imidazole containing 1 x Roche tablet
	Elution	20 mM Na_2PO_4 pH 7.4, 500 mM NaCl, 500 mM Imidazole
	Dialysis	50 mM HEPES pH 7.4, 150 mM NaCl, 1 mM DTT
**UbcH7a**$^{GST\text{-}F2}$	Resuspension & Wash	10 mM Na_2HPO_4 pH 7.4, 2 mM KH_2PO_4, 2.7 mM KCl, 137 mM NaCl containing 1 x Roche tablet
	Elution	10 mM Na_2HPO_4 pH 7.4, 2 mM KH_2PO_4, 2.7 mM KCl, 500 mM NaCl
**WWP1-L34H**$^{His\text{-}Trx\text{-}3C}$	Resuspension	50 mM Tris pH 8.0, 500 mM NaCl, 10 mM imidazole containing 0.1 mM PMSF
	Wash	50 mM Tris pH 8.0, 500 mM NaCl, 30 mM imidazole
	Elution	50 mM Tris pH 8.0, 500 mM NaCl, 250 mM imidazole
	Dialysis & SEC	50 mM Tris pH 8.0, 100 mM NaCl, 1 mM DTT, 1 mM EDTA
	Buffer Exchange	50 mM Tris pH 8.0, 500 mM NaCl, 1 mM DTT
	Second SEC	50 mM Tris pH 8.0, 500 mM NaCl, 1 mM DTT, 1 mM EDTA
**WWP1-2L34H**$^{His\text{-}Trx\text{-}3C}$	Resuspension & Wash	100 mM Tris pH 8.0, 250 mM NaCl, 10 mM imidazole containing 0.1 mM PMSF
	Elution	100 mM Tris pH 8.0, 250 mM NaCl, 250 mM imidazole
	Dialysis	100 mM Tris pH 8.0, 100 mM NaCl, 1 mM DTT. 1 mM EDTA, 25 mM Maltose
	SEC	50 mM Tris pH 8.0, 500 mM NaCl, 1 mM DTT, 1 mM EDTA
**WWP2-LH**$^{GST\text{-}3C}$	Resuspension	25 mM Tris.HCl pH 8.0, 250 mM NaCl containing 0.1 mM PMSF and 1 x Roche tablet.
	Wash	25 mM Tris.HCl pH 8.0, 250 mM NaCl, 0.1 % Triton X-100
	Elution	25 mM Tris.HCl pH 8.0, 250 mM NaCl, 50 mM reduced glutathione.
	Dialysis	25 mM Tris.HCl pH 8.0, 250 mM NaCl, 5 mM DTT
	SEC	25 mM Tris.HCl pH 7.5, 150 mM NaCl, 5 mM DTT or 3 mM TCEP

SDS-PAGE Analysis

Samples were collected at relevant purification stages and diluted to the appropriate concentration for SDS-PAGE analysis using Bolt^{Tm} pre-cast 4 – 12 % BIS-Tris Plus acrylamide gels. Manufacture protocols followed by combining sample, 1 × Bolt^{Tm} LDS Buffer and 1 × Bolt^{Tm} Reducing Agent before heating in a thermal cycler at 75 °C for 10 minutes and running gels in the Invitrogen Mini Gel Tank using 1 × Bolt^{Tm} MES

SDS Buffer at 165 V for 30 minutes. InstantBlue (Expedeon) was used to stain gels overnight before de-staining and storing in H_2O.

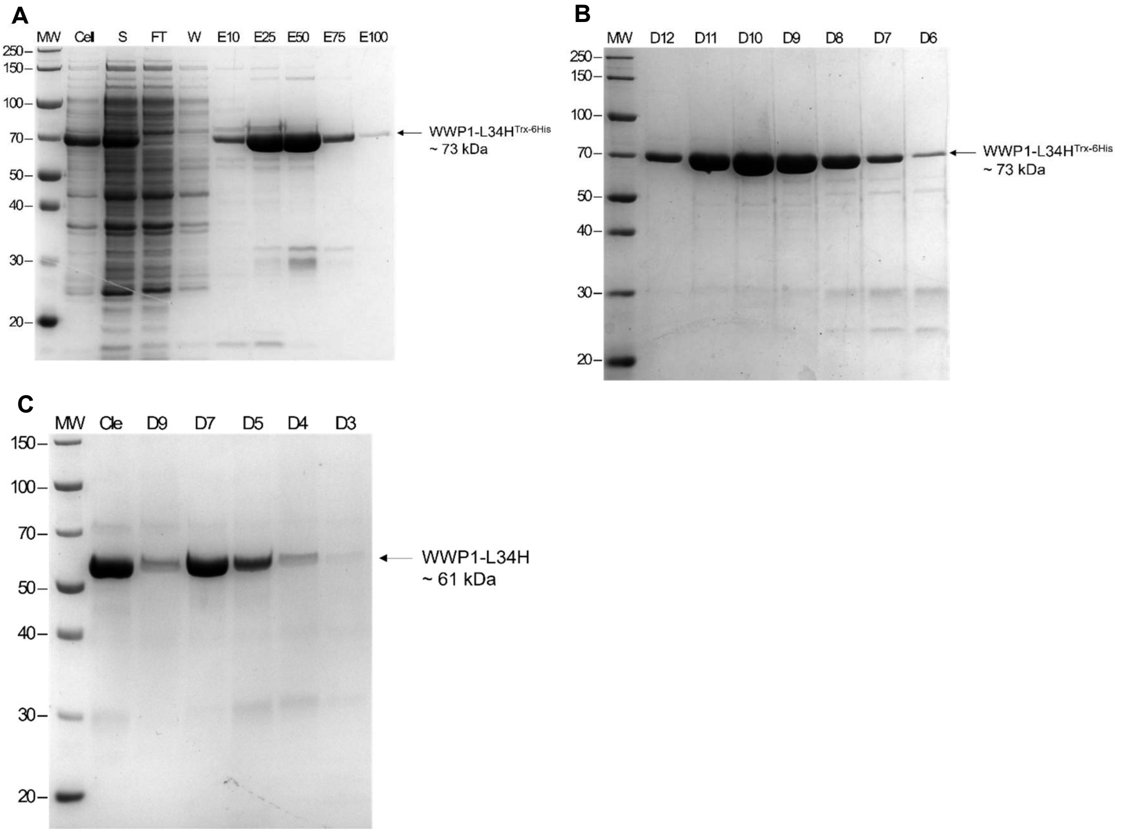

Figure S9. SDS-PAGE analysis of WWP1-L34H purification. (A) Stepwise IMAC isolation. Intense band at ~73 kDa indicating Trx-His tagged WWP1-L34H. Lane labels: Ladder (MW), whole cell (Cell), soluble lysate (S), column flow-through (FT), column wash (W), 10% elution (E10), 25% elution (E25), 50% elution (E50), 75% elution (E75), 100% elution (E100). All fractions were pooled. (B) First SEC purification. Trx-His tagged WWP1-L34H identified as shown by the band at ~73 kDa. Lanes labelled as column fraction positions excluding ladder (MW). (C) Cleavage and second SEC purification. Intense band at ~61 kDa as untagged WWP1-L34H. Lanes labelled as fraction positions other than ladder (MW) and cleavage sample (Cle). Fractions D9 – D4 were pooled. Image enhanced using Image Lab (BioRad).

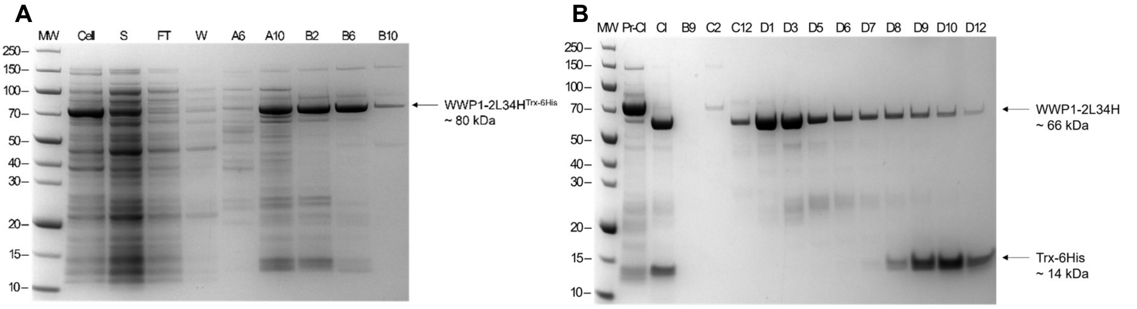

Figure S10. SDS-PAGE analysis of WWP1-2L34H purification. (A) Gradient IMAC isolation. Intense bands at ~80 kDa indicating Trx-His tagged WWP1-2L34H. Lane labels: Ladder (MW), whole cell (Cell), soluble lysate (S), column flow-through (FT), column wash (W), elution fractions positions (A6 – B10). Fractions A10 – B10 were pooled. (B) SEC purification. Untagged WWP1-2L34H identified by intense bands at ~66 kDa. Lanes labelled as fraction positions other than ladder (MW), pre-cleavage (Pr-Cl) and cleavage sample (Cl). Fractions C12 – D6 were pooled. Image enhanced using Image Lab (BioRad).

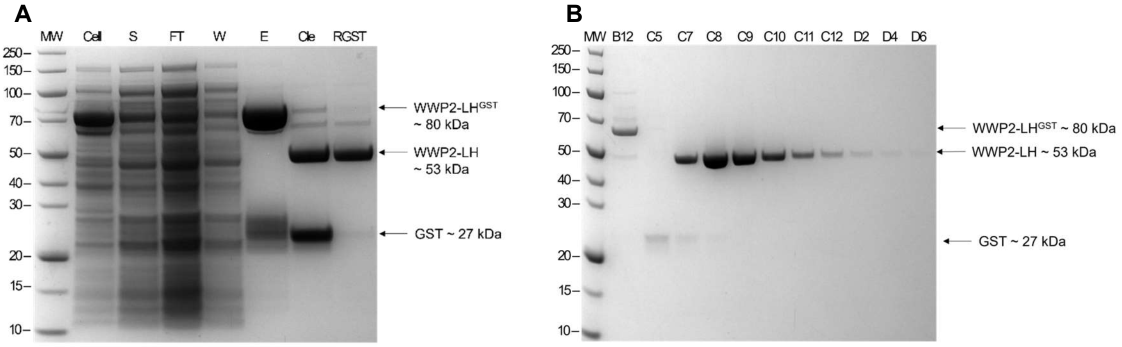

Figure S11. SDS-PAGE analysis of WWP2-LH purification. (A) Straight GSTrap isolation and cleavage. Isolation and cleavage of GST-tagged WWP2-LH are shown by an intense band at ~80 kDa and ~53 kDa respectively, with free GST shown at ~27 kDa band. Lane labels: Ladder (MW), whole cell (Cell), soluble lysate (S), column flow-through (FT), column wash (W), column elution (E), cleavage sample (Cle) and reverse GSTrap flow-through (RGST). (B) SEC purification. Untagged WWP2-LH identified by intense bands at ~53 kDa. Lanes labelled as fraction positions, with fractions C7 – D6 pooled. Image enhanced using Image Lab (BioRad).

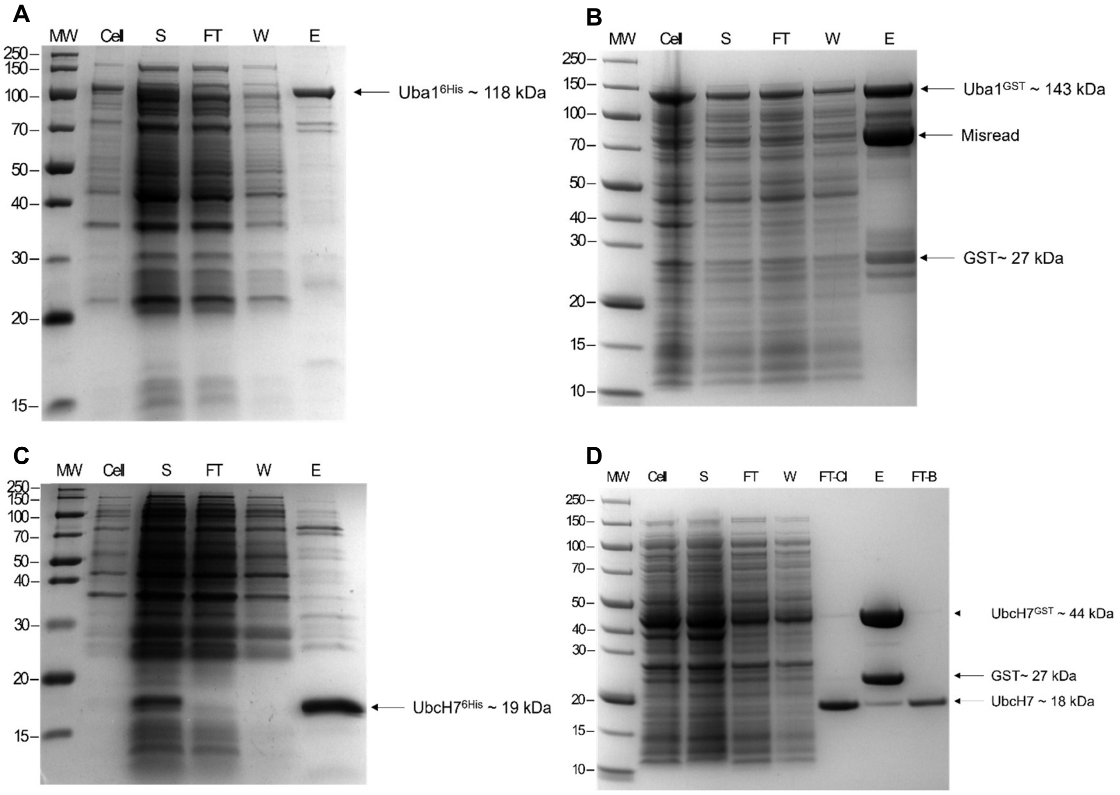

Figure S12. SDS-PAGE analysis of Uba1 and UbcH7 purifications. Lane labels: Ladder (MW), whole cell (Cell), soluble lysate (S), column flow-through (FT), column wash (W), column elution (E), on-column cleavage flow-through (FT-Cl) and Benzamidine column flow-through (FT-B). (A) Straight IMAC isolation of Uba1. Intense band at ~118 kDa for His-tagged Uba1. (B) Straight GST affinity isolation of Uba1. Intense bands at ~ 143 kDa for GST-tagged Uba1, as well as possible misread at ~80 kDa. (C) Straight IMAC isolation of UbcH7. Intense band at ~19 kDa for His-tagged UbcH7. (D) Straight GST affinity isolation of UbcH7 and cleavage. Intense bands at ~44 kDa for GST-tagged UbcH7, ~ 27 kDa for free GST and 18 kDa for untagged UbcH7. Image enhanced using Image Lab (BioRad).

ELISA Autoubiquitination Assay

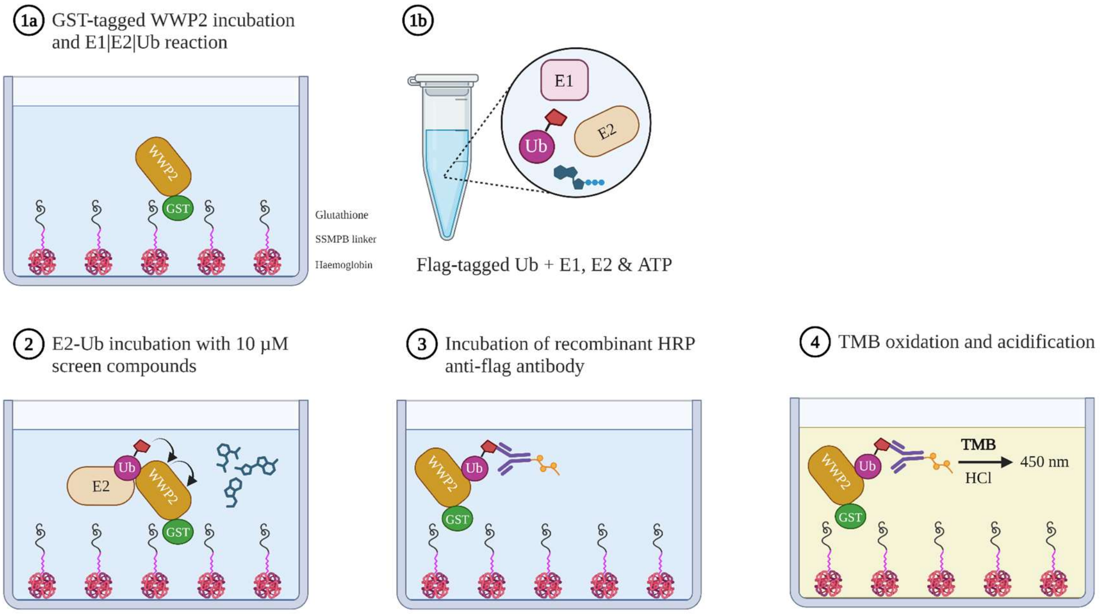

Figure S13. Schematic diagram of the WWP2 ELISA autoubiquitination assay. (1a) Immobilisation of GST-Tagged WWP2 on glutathione coated plates. The GST-tagged (green) WWP2-FL (orange) is bound to glutathione (black), further linked via an SSMPB linker (pink) bound to haemoglobin (red). (1b) Reaction mixture of His-E1, His-E2 and flag-tagged Ub. Flag-tagged (red) Ub (dark pink) is first charged to Uba1/E1 (light pink) using ATP (blue) and then transferred to UbcH7/E2 (light brown). (2) Ub charged E2 incubation with immobilised WWP2. UbcH7/E2 transfers flag-tagged Ub to WWP2 before WWP2 auto-ubiquitination occurs in the absence or presence of compounds (blue). (3) Incubation of recombinant HRP anti-flag antibody with auto-ubiquitinated WWP2. Anti-flag antibody (purple) containing recombinant HRP (orange) associates with the flag-tagged Ub. (4) HRP-mediated oxidation of TMB. Single-electron oxidation of TMB by HRP in the presence of HO creates first a blue colour change, before acidification results in a yellow colour change (450 nm) stopping the reaction.

Chemical Experimental Section

Anhydrous THF was freshly distilled from sodium and benzophenone. Anhydrous dichloromethane was freshly distilled from calcium hydride powder. Anhydrous dioxane (1,4-dioxane) was prepared by storing over activated 4 Å molecular sieves for at least 24 h. All other solvents and reagents were purchased and used as supplied. Thin-layer chromatography was performed on Merck silica gel 60 F254 plates and visualised by UV absorption, purchased from VWR International. Flash column chromatography was carried out using Silica Gel 60 purchased from Material Harvest. 'Concentrated' refers to the removal of volatile organic solvents *via* distillation using a rotary evaporator. 'Dried' refers to pouring onto or adding anhydrous $MgSO_4$ or Na_2SO_4 to (as specified), followed by filtration. Water refers to deionised water. Compounds **25**, **26** and **27** were purchased from Fluorochem, their ^{1}H and ^{13}C NMR were taken for purity analysis, and are included here.

Unless specified, all reagents and starting materials were purchased from commercial sources (Sigma-Aldrich (Merck Life Sciences), Fluorochem (Doug Discovery), Fischer Scientific, Alfa Aesar) and used as received. NMR spectra were recorded on 400 or 500 MHz Bruker NMR spectrometer using the deuterated solvent stated in the reported data. ^{1}H, ^{13}C, NMR samples were prepared by dissolving a sample in 0.4 mL – 0.7 mL deuterated solvent. All deuterated solvents were purchased from Cambridge Isotopes and used as received, solvents were stored under 4 Å molecular sieves after opening. All spectra were referenced to the residual solvent peaks of the solvent used.[2] NMR spectra chemical shifts (δ) are reported in ppm and coupling constants (J) are reported in hertz (Hz). Abbreviations for NMR splitting are s (singlet), d (doublet), t (triplet), q (quartet), p (pentet) and m (multiplet). Infrared spectra were

recorded using a Perkin Elmer Spectrum Two LITA. High-resolution mass spectrometry was performed at the University of East Anglia using a UPLC-HRMS (ACQUITY H-Class PLUS UPLC and Waters SYNAPT XS High Resolution Mass Spectrometer) setup with electrospray ionisation using ca. 1 μgmL^{-1} solution in acetonitrile or methanol. Melting points (not corrected) were recorded on a Büchi Melting Point B-545 using capillary melting point tubes made in-house.

^{1}H NMR and HRMS data for purity analysis of NCI NSC217913 sample

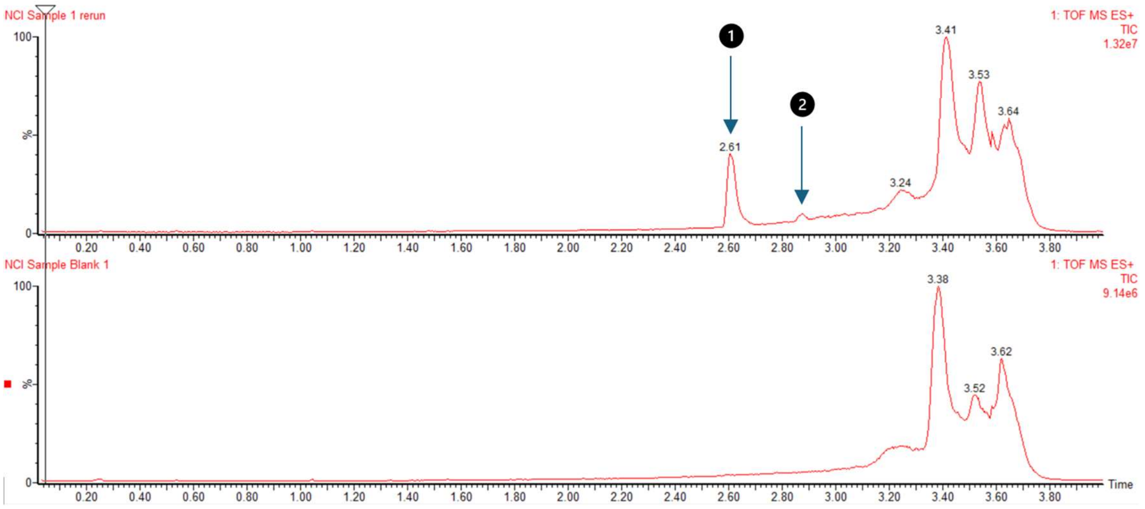

Figure S14. UPLC chromatogram trace of NCI sample (top) *vs*. blank (bottom). Peaks 1 and 2 represent NSC-217913 and an identified impurity, respectively.

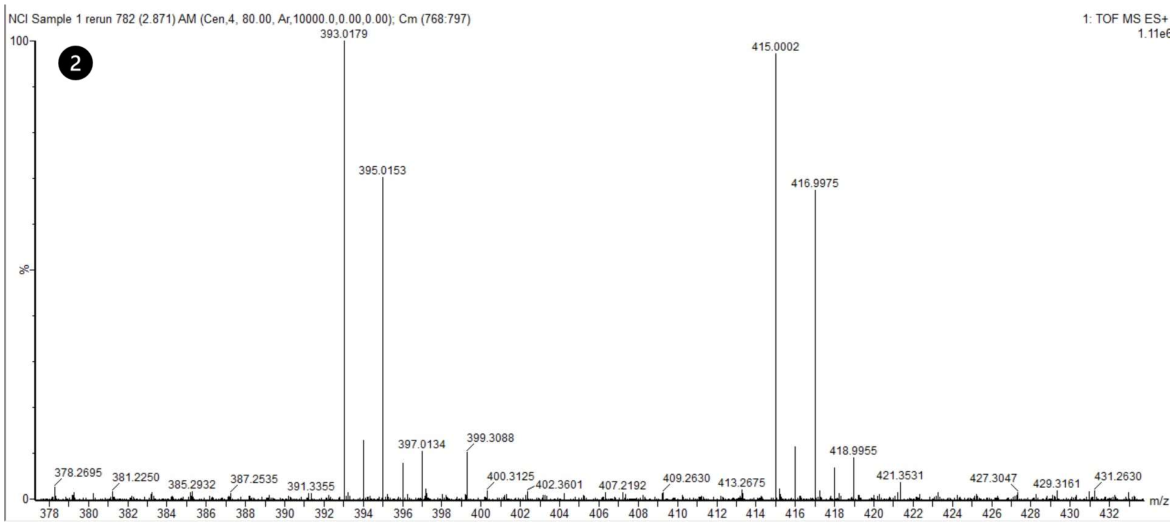

Figure S15. M+H and M+Na *m/z* signals for the identified impurity 2.

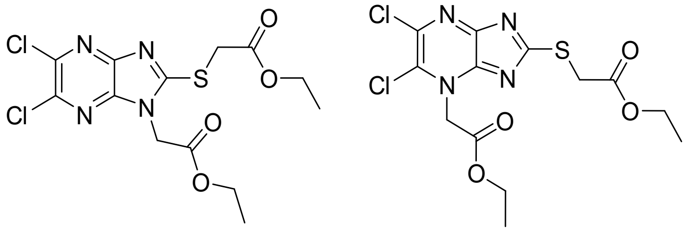

Figure S16. Proposed structures of impurity 2 based on HRMS data.

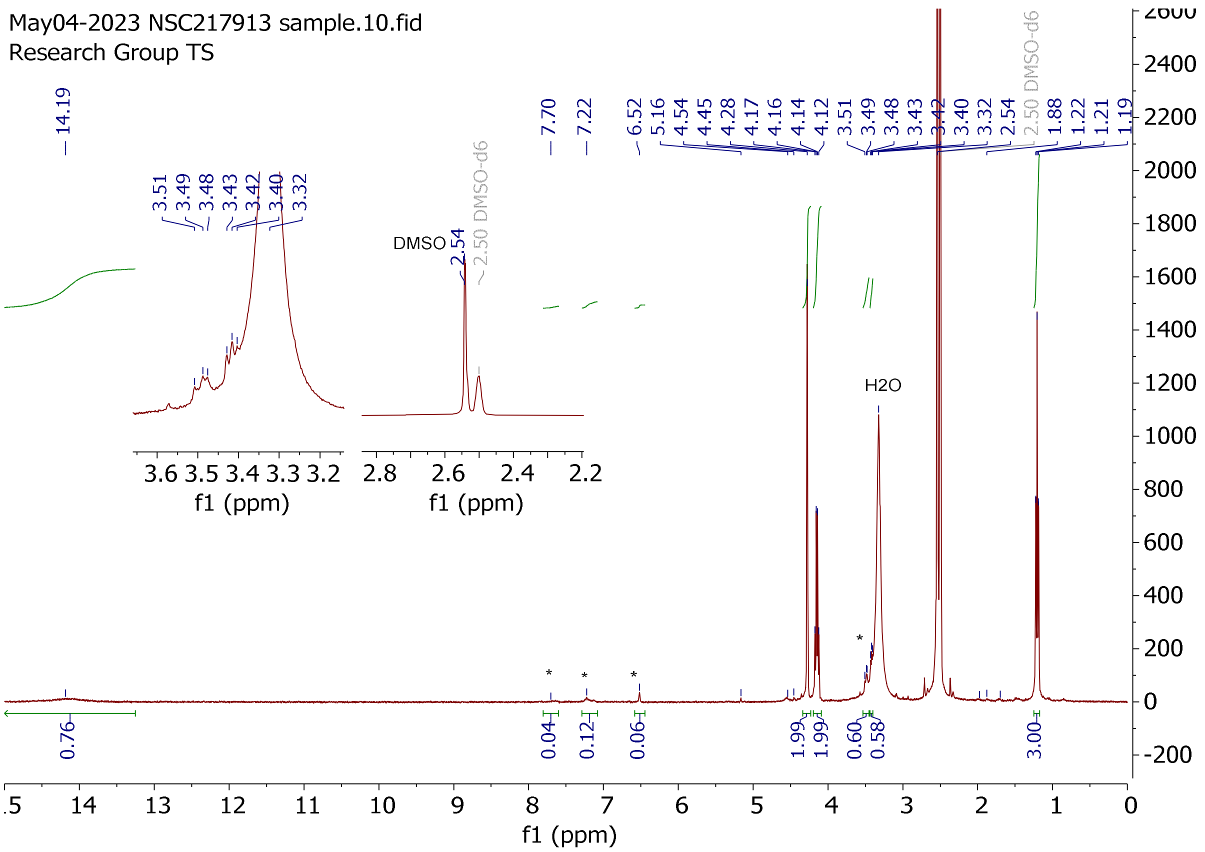

Figure S17. ^{1}H NMR spectrum for NCI sample of NSC-217913 in DMSO-d_6. Expansion 2.2 – 2.8 ppm shows the DMSO remaining from sample preparation. Expansion 3.1 – 3.65 ppm shows some significant impurities shouldering the water signal.

General procedure 1: Ester hydrolysis

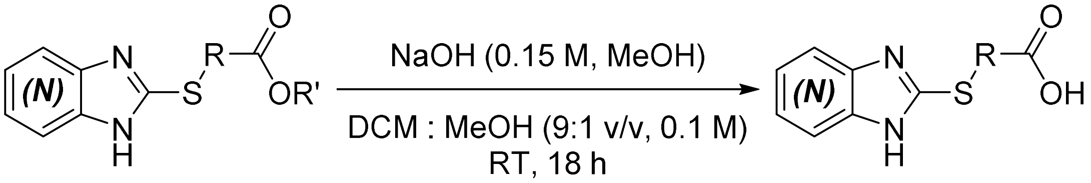

A stock solution of NaOH (0.25 M) (NaOH (50 mg) in methanol (5 mL)) and DCM : MeOH (9:1 v/v, 10 mL) were prepared. To an 8 mL vial <u>starter compound</u> and a stirrer bar were added. DCM : MeOH (9:1 v/v) solution was added to reach a concentration of 0.1 M. Methanolic NaOH solution was added to reach a NaOH concentration of 0.15 M and a final reagent concentration of 0.063 M. The mixture was left to stir at RT for 18 h. Afterwards, TLC analysis (7:3 Pet E: EtOAc) indicated complete consumption of starting material, solvents were removed under reduced pressure and the residue dissolved in water (5 mL). Extracted with Et_2O (2x 10 mL) and the aqueous layer acidified (2 M HCl, 5 mL) and re-extracted with Et_2O (3x 10 mL), these extracts were collected and dried ($MgSO_4$) and solvent removed under reduced pressure.

Adapted from literature procedure[5].

General procedure 2: Thione formation

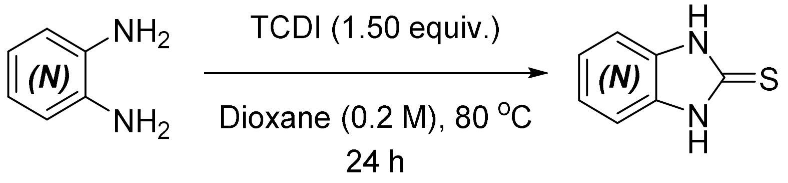

To a 50 mL RBF was added the diamino-heterocycle, a stirrer bar and TCDI (1.0 equiv.). Dioxane (0.2 M) was added, and the mixture heated to 80 °C for 18 h. Afterwards, TLC indicated incomplete reaction and additional TCDI (0.408 g, 0.5 equiv.) was added, and the mixture further heated at 80 °C for 6 h. After TLC indicated complete reaction, allowed to cool to RT and dioxane removed under reduced pressure. The residue was taken up in EtOAc and HCl (1.0 M) (10 mL each). The layers separated and the aqueous layer extracted with EtOAc (3x 20 mL). The organic layers were collected and dried ($MgSO_4$) and solvent removed to provide crude material that is further purified on an individual basis.

General procedure 3: S-alkylation of heterocyclic thiones

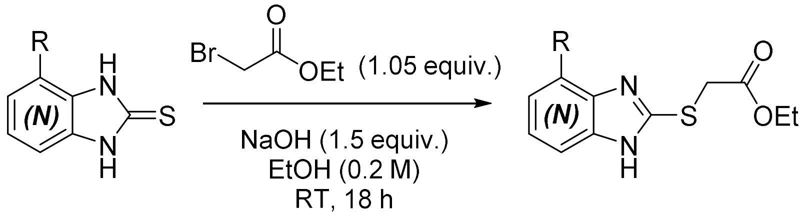

To an 8 mL vial was added imidazo-2-thione derivative and the respective amount of sodium hydroxide (1.5 equiv.). EtOH (0.2 M) was added *via* syringe with stirring. Ethyl bromoacetate (1.05 equiv.) was added *via* microsyringe. The vial was sealed with a septum and an exit needle. Left to stir for 18 h at RT. Afterwards, the ethanol was removed *in vacuo.* and the residue was taken up in sat. soln. NH_4Cl and EtOAc (*ca.* 5 mL each). Transferred to a separatory funnel and the layers separated. The aqueous layer was extracted with EtOAc (2x 10 mL), and organic layers collected and dried ($MgSO_4$). Solvent removed under reduced pressure to provide a residue, further purified on an individual basis.

Adapted from literature procedure[5].

Heterocyclic Synthesis

Strategy 1: 2-amino-4-chloropyrazine, 2-amino-3-chloropyrazine & 2-amino-3,5-dichloropyrazine

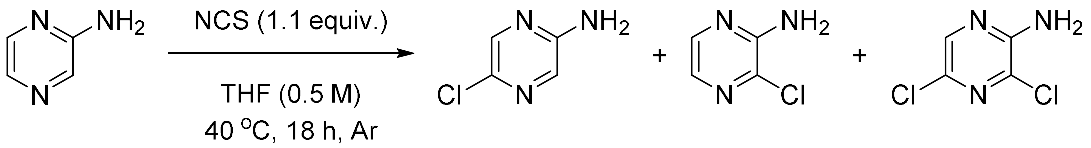

To an oven-dried 50 mL RBF was added 2-aminopyrazine (1.0 g, 10.51 mmol) and a stirrer bar. This was purged with argon and anhydrous THF (21 mL, 0.5 M) was added *via* syringe. To the stirred solution NCS (1.544 g, 1.1 equiv.) was then added in one portion with stirring until the majority dissolved. The reaction was then heated to 40 °C for 18 h. The reaction was allowed to cool to RT and filtered through a pad of Celite®, washing with EtOAc. The filtrate was then washed with $NaHCO_3$ sat. soln. (3x 30 mL), dried (Na_2SO_4) and dry loaded onto silica gel. Purified by column chromatography (9:1 -> 8:2 -> 7:3 Hexane : EtOAc) to yield:

2-amino-5-chloropyrazine as a yellow powder (518 mg, 3.99 mmol, 38%).

^{1}H NMR (400 MHz, C*D*Cl$_3$) δ 8.01 (d, *J* = 1.5 Hz, 1H), 7.78 (d, *J* = 1.5 Hz, 1H), 4.58 (br s, 2H).

^{13}C NMR (101 MHz, C*D*Cl$_3$) δ 153.2, 141.4, 137.8, 131.0

Data in-line with literature values[6].

NMR data:

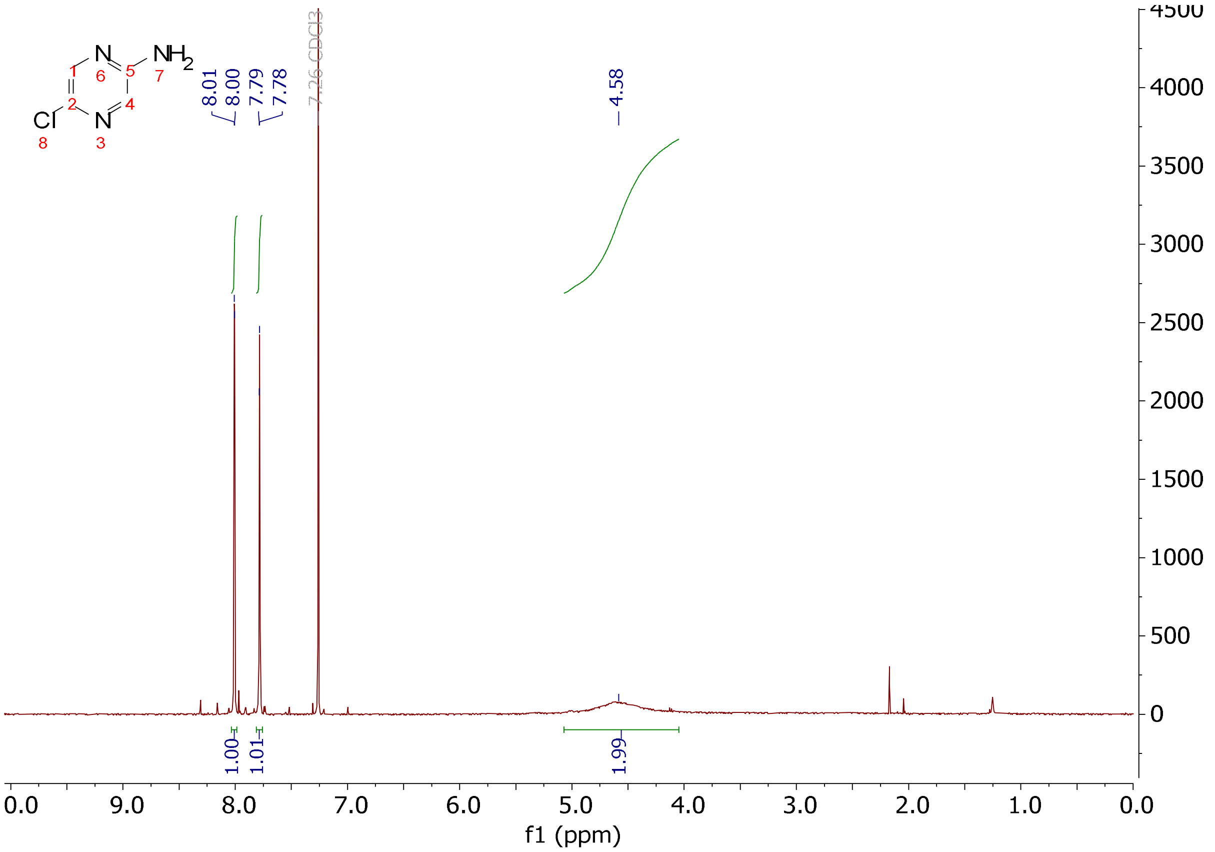

NH2
Cl
N
8.01
8.00
7.79
7.78
7.26 CDCl3
4.58
1.00
1.01
1.99
f1 (ppm)

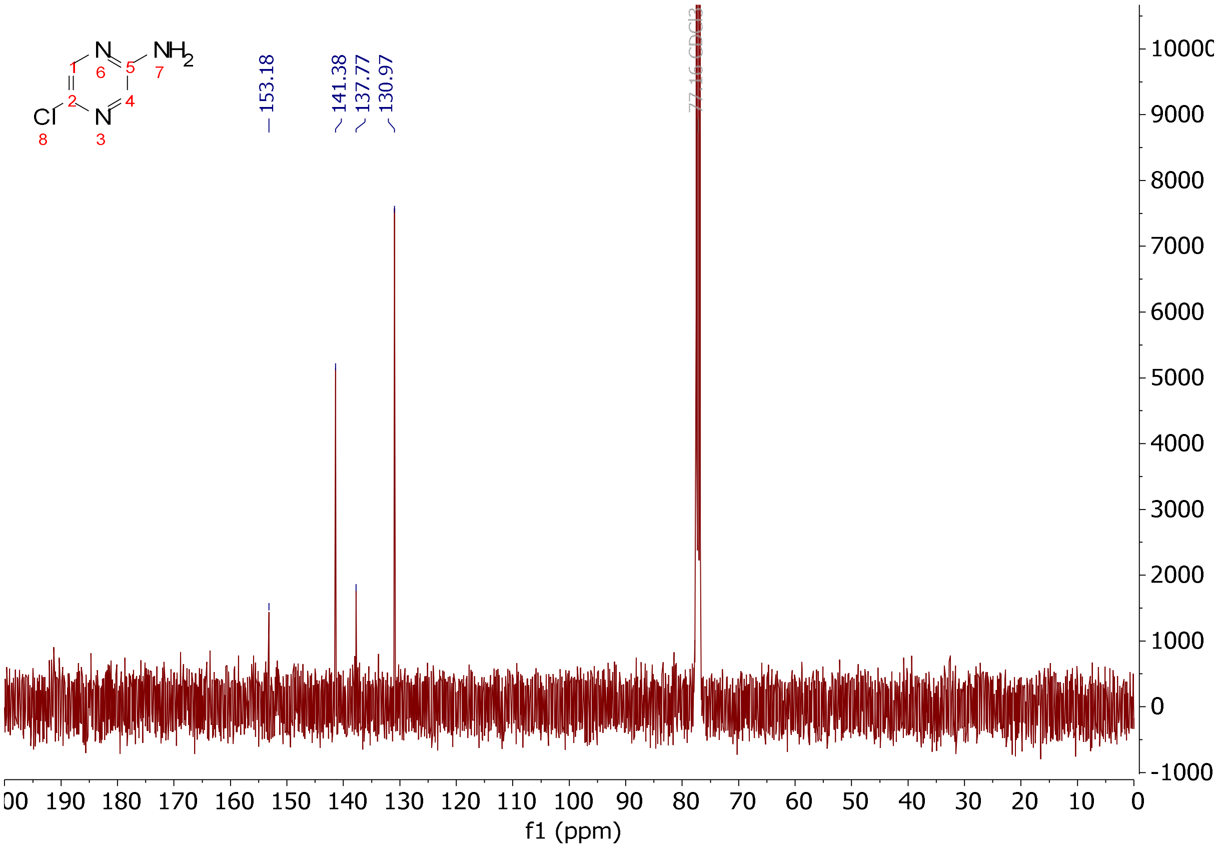

NH2
Cl
N
153.18
141.38
137.77
130.97
77.16 CDCl3
f1 (ppm)

2-amino-3-chloropyrazine as a light-yellow powder (148 mg, 1.14 mmol, 11%).

^{1}H NMR (400 MHz, C*D*Cl$_3$) δ 7.93 (d, J = 2.7 Hz, 1H), 7.72 (d, J = 2.7 Hz, 1H), 5.09 (br s, 2H).

^{13}C NMR (101 MHz, C*D*Cl$_3$) δ 151.7, 140.6, 134.4, 133.1.

Data in-line with literature values[7].

NMR data:

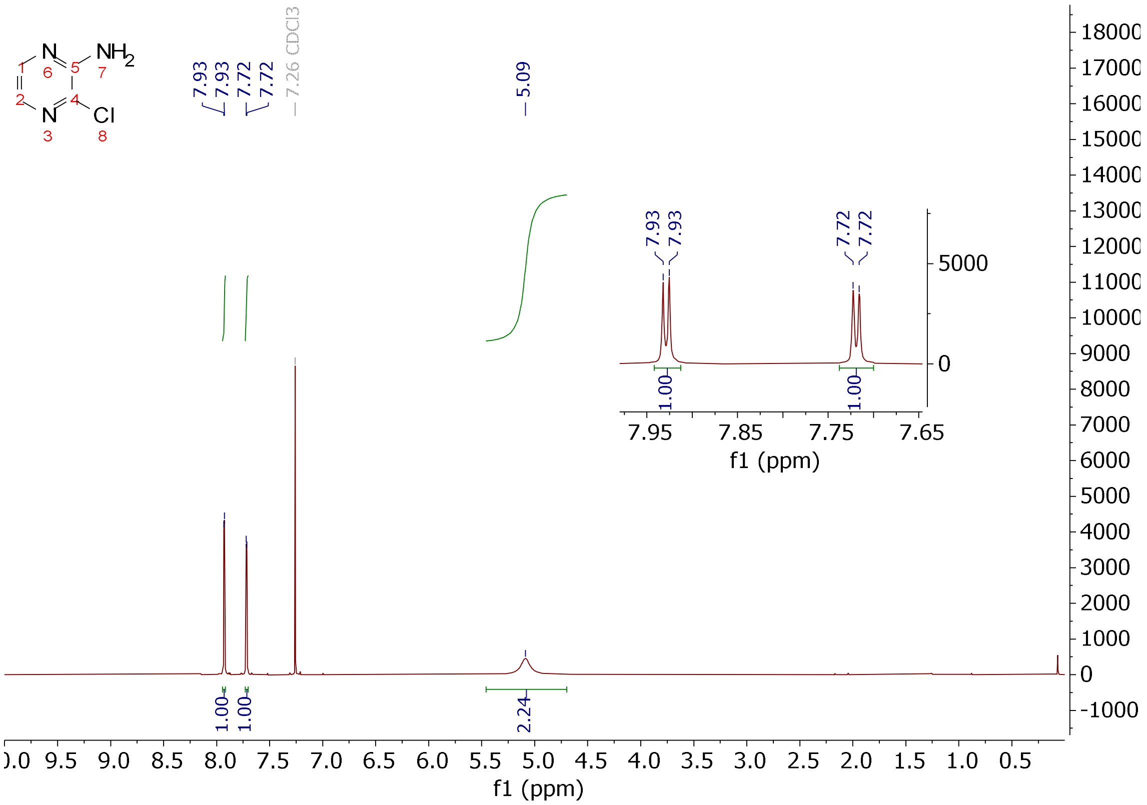

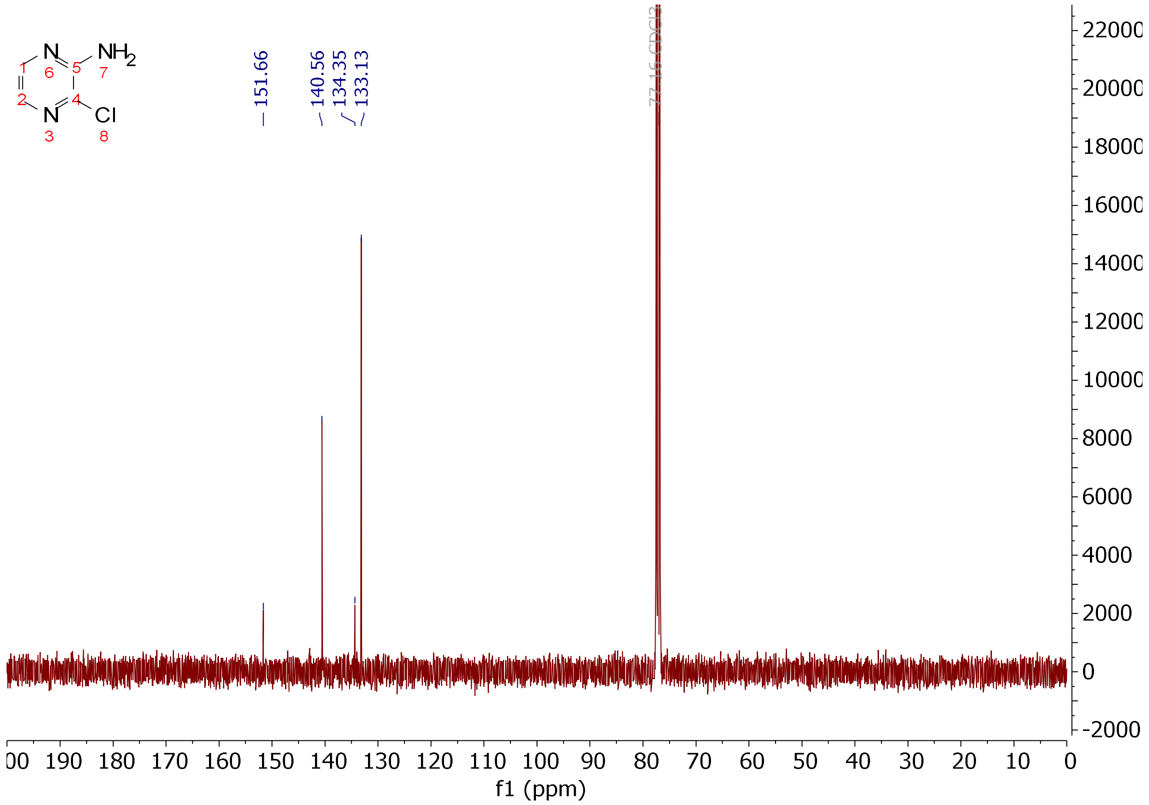
N
NH2
Cl
1
2
3
4
5
6
7
8
151.66
140.56
134.35
133.13
77.16 CDCl3
22000
20000
18000
16000
14000
12000
10000
8000
6000
4000
2000
0
-2000
00 190 180 170 160 150 140 130 120 110 100 90 80 70 60 50 40 30 20 10 0
f1 (ppm)

2-amino-3,5-dichloropyrazine as white fluffy crystals (133 mg, 0.81 mmol, 15%).

^{1}H NMR (400 MHz, C*D*Cl$_3$) δ 7.97 (s, 1H), 5.01 (br s, 2H).

^{13}C NMR (101 MHz, C*D*Cl$_3$) δ 150.5, 140.2, 134.8, 131.6.

IR (cm^{-1}) 3435, 3291, 3167, 1620, 1562.

Adapted from literature conditions, data in-line with literature values[8,9].

NMR data:

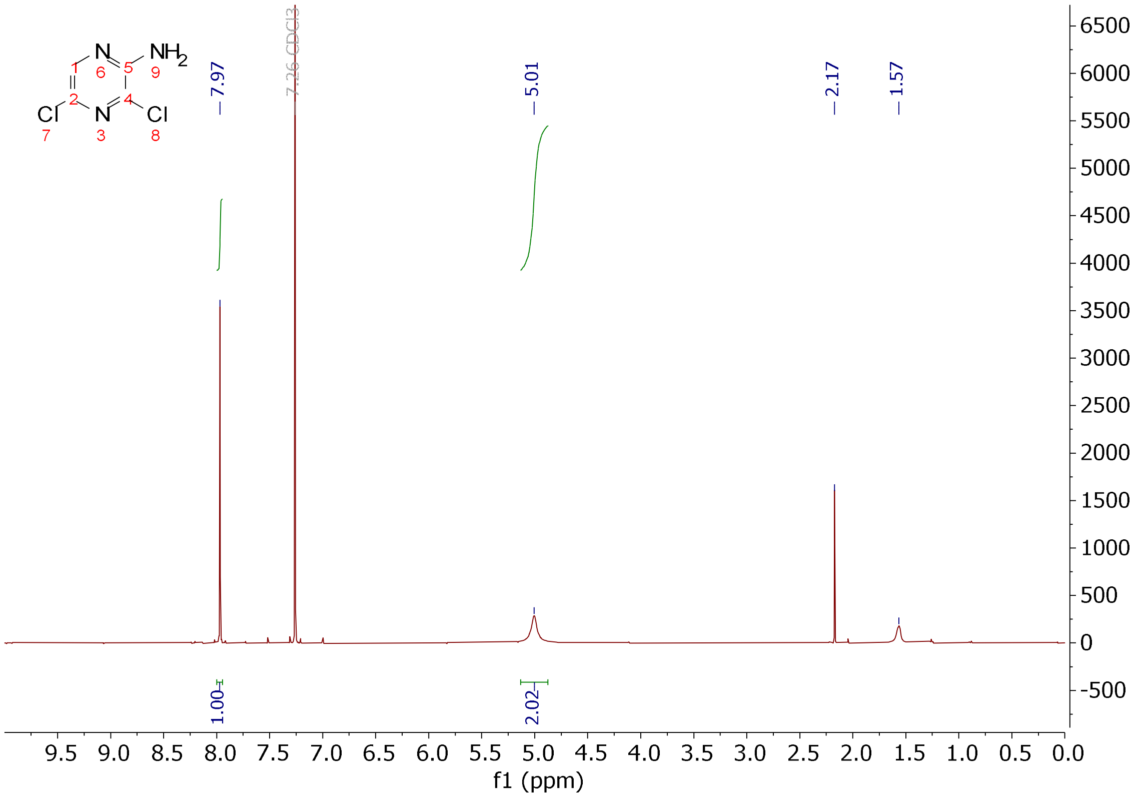

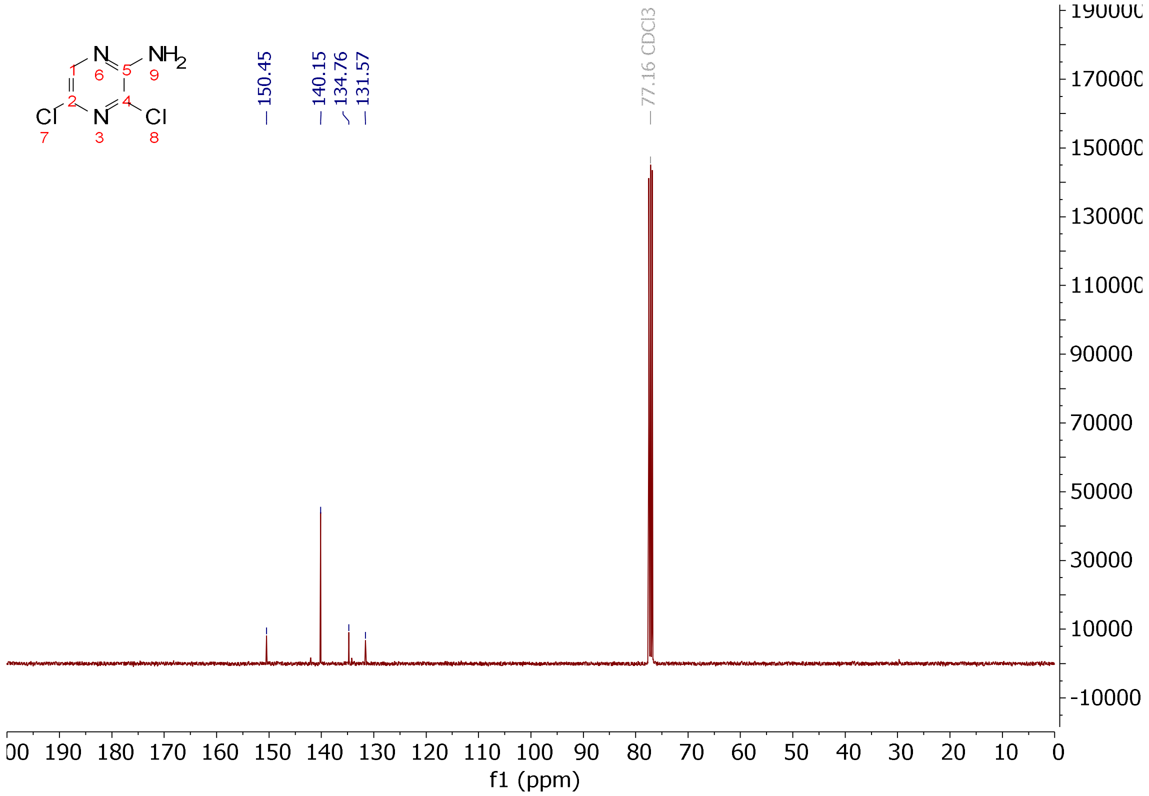

NH2
N
Cl
N
Cl
1
2
3
4
5
6
7
8
9
150.45
140.15
134.76
131.57
77.16 CDCl3
f1 (ppm)

Strategy 1_1: 2,3-diamino-5-choropyrazine

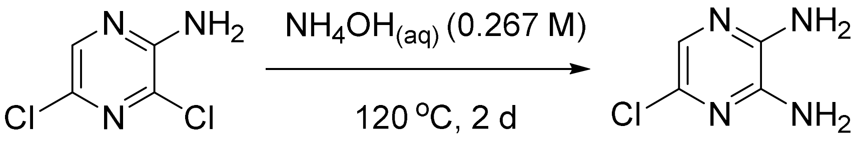

To two 30 mL microwave vials was added 2-amino-3,5-dichloropyrazine (total of 1.00 g (6.09 mmol), 0.5 g per vial) and a stirrer bar. To each vial was added ammonium hydroxide solution (12.5 mL, 0.276 M) and sealed. Vials were placed in a sand bath and heated with stirring to 120 °C (sand temp.) for 2 days. The vessels were allowed to cool to RT, contents transferred to a separatory funnel and extracted with EtOAc (3x 20 mL). The organic extracts were collected and washed with brine (20 mL) and dried ($MgSO_4$). The solvent was removed *in vacuo.*, the material was dry loaded (acetone) and purified by column chromatography (5:5 -> 2:8 -> 0:1 Hex. : EtOAc) to provide 2,3-diamino-5-chloropyrazine (0.447 g, 3.09 mmol, 50%) as a beige solid.

^{1}H NMR (400 MHz, Acetone-d_6) δ 7.24 (s, 1H), 5.72 (br s, 2H), 5.47 (br s, 2H).

^{13}C NMR (101 MHz, Acetone-d_6) δ 144.5, 143.5, 134.3, 127.7.

IR (cm^{-1}) 3440 (NH), 3375 (NH), 1646 (NH bend).

M.P. 222.7 – 223.1 °C (deg.).

NMR data:

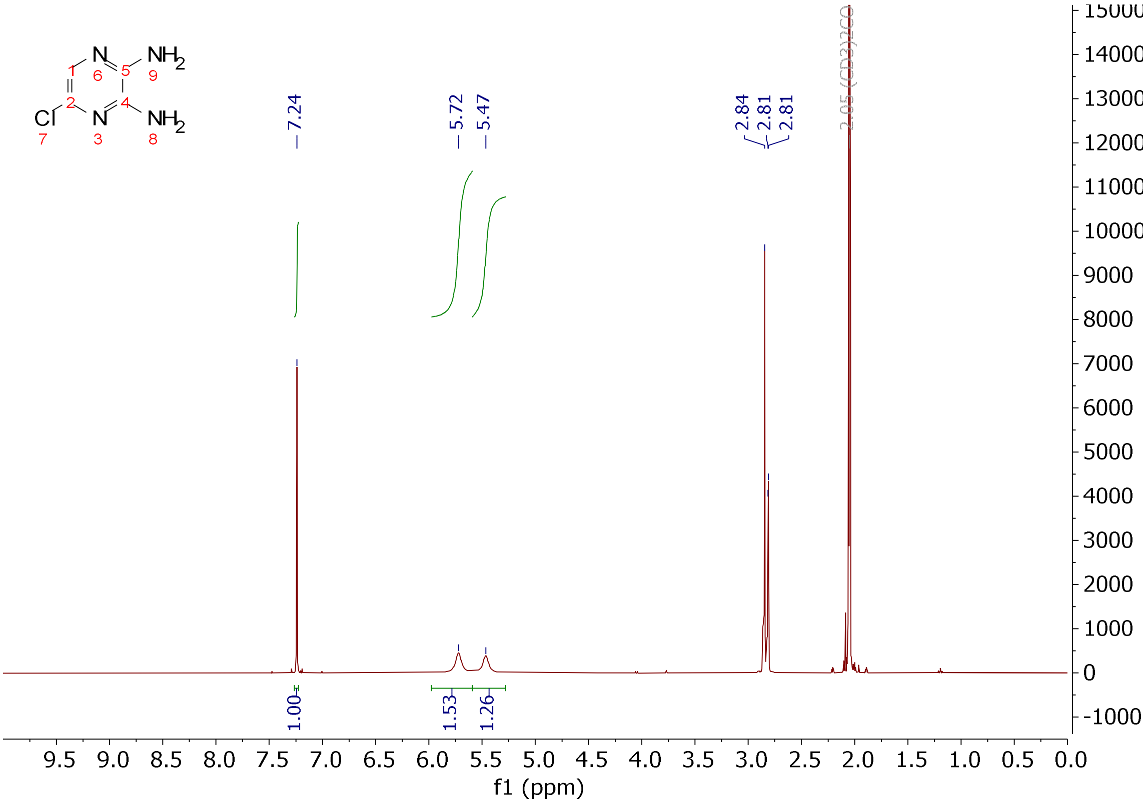

NH2
NH2
Cl
N
N
7.24
5.72
5.47
2.84
2.81
2.81
2.05 (CD3)2CO
1.00
1.53
1.26
f1 (ppm)

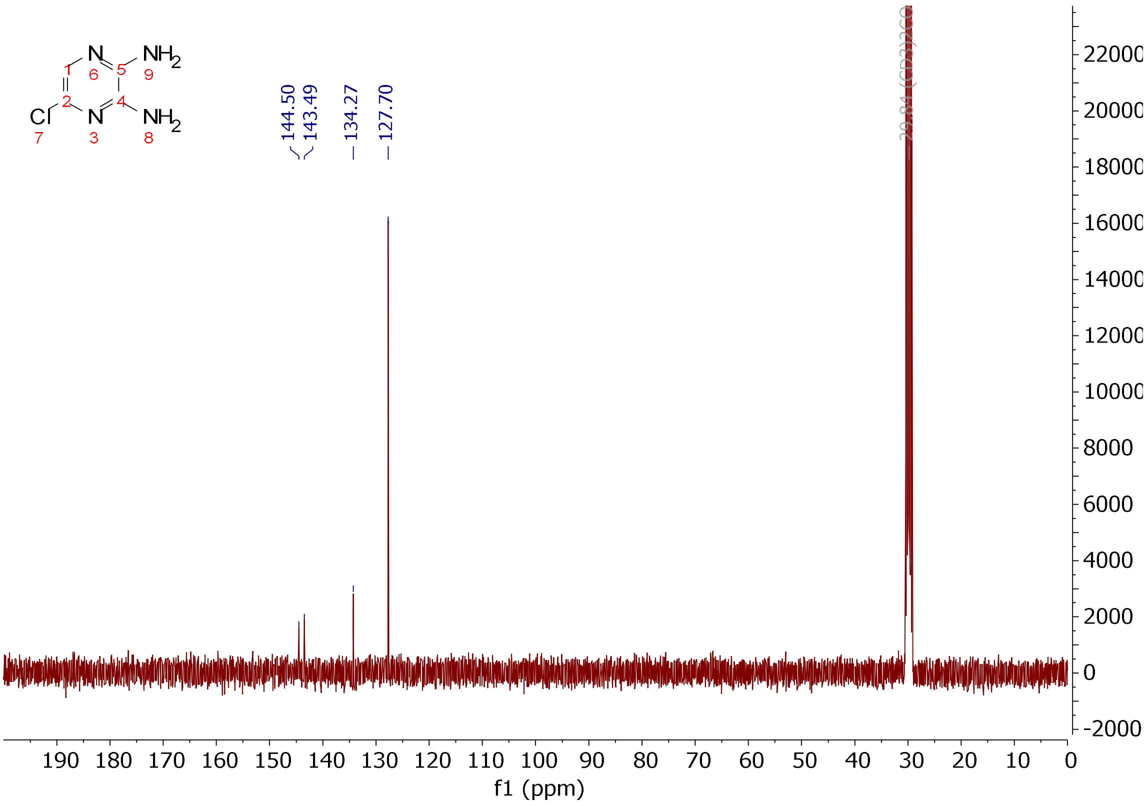

NH2
NH2
Cl
N
N
144.50
143.49
134.27
127.70
29.84 (CD3)2CO
f1 (ppm)

Strategy 1_1_: 15-chloroimidazo[4,5-b]pyrazine-2(1,3H)-thione

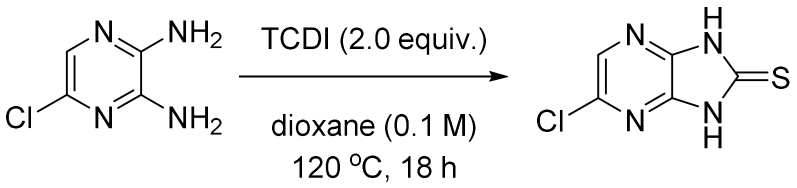

To a 100 mL RBF was added 2,3-diamino-5-chloropyrazine (0.5 g, 3.458 mmol). To the RBF was attached an air condenser, and the system placed under argon atmosphere. Dioxane (35 mL, 0.1 M, anhydrous) was added *via* syringe and the mixture was heated to 80 °C to dissolve starting material. Thiocarbonyldiimidazole (0.65 g, 1.05 equiv.) was added in one batch and the mixture heated with stirring to 120 °C. Monitored by TLC, an additional 0.5 equiv. of TCDI was added after 4 h (0.308 g). Heated to reflux for 18 h. TLC indicated complete reaction, mixture was allowed to cool to RT, dioxane removed *in vacuo.* The residue was taken up in EtOAc, washed with 1 M HCl (20 mL). The organic layer was removed and the aqueous extracted with EtOAc (3x 30 mL). The organic layers were collected and dried ($MgSO_4$) and solvent removed *in vacuo.* to provide a brown solid (593 mg, 3.17 mmol, 92%).

^{1}H NMR (400 MHz, DMSO-d_6) δ 13.63 (br s, 2H, 8), 8.16 (s, 1H).

^{13}C NMR (101 MHz, DMSO-d_6) δ 172.9, 140.4, 140.3, 140.0, 134.4.

IR (cm^{-1}) 2768 (SH), 1616 (NH bend).

M.P. 281.7 – 282.9 °C.

MS ES+ m/z calcd for $C_5H_3ClN_4S$ $(M+H)^+$: 186.9845, found: 186.9844.

NMR data:

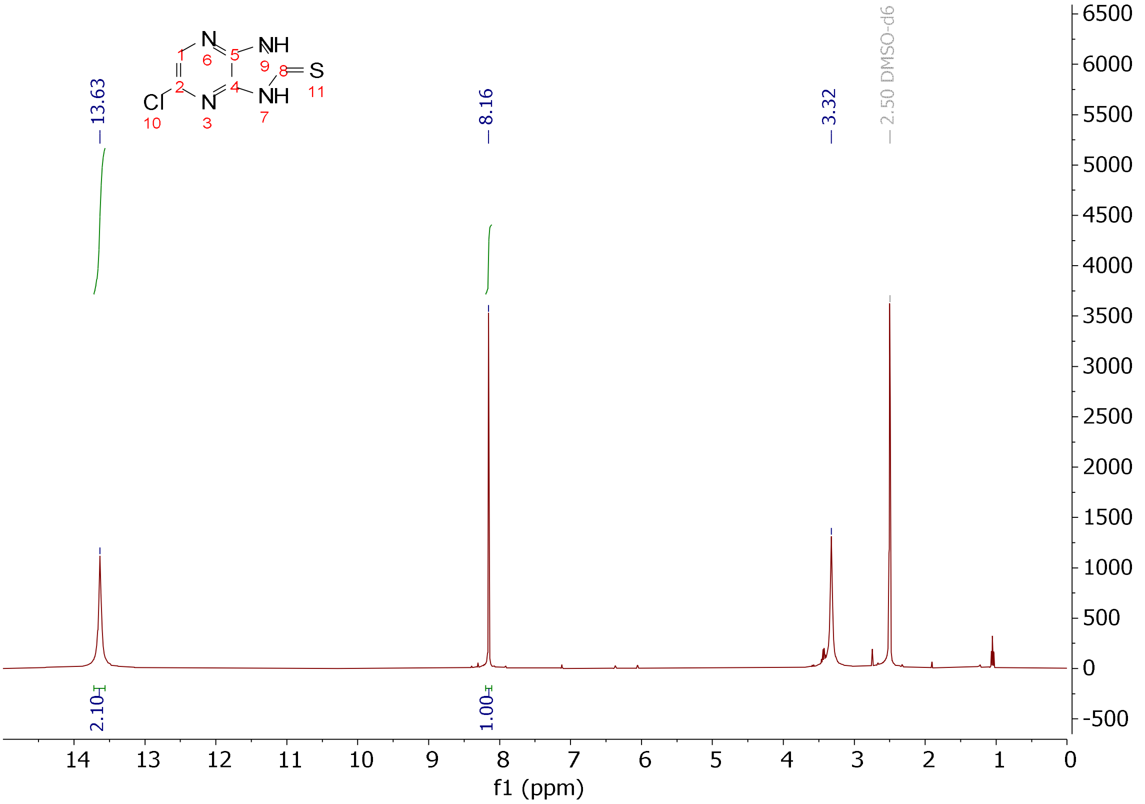
13.63
8.16
3.32
2.50 DMSO-d6
2.10
1.00
f1 (ppm)
Cl
NH
S

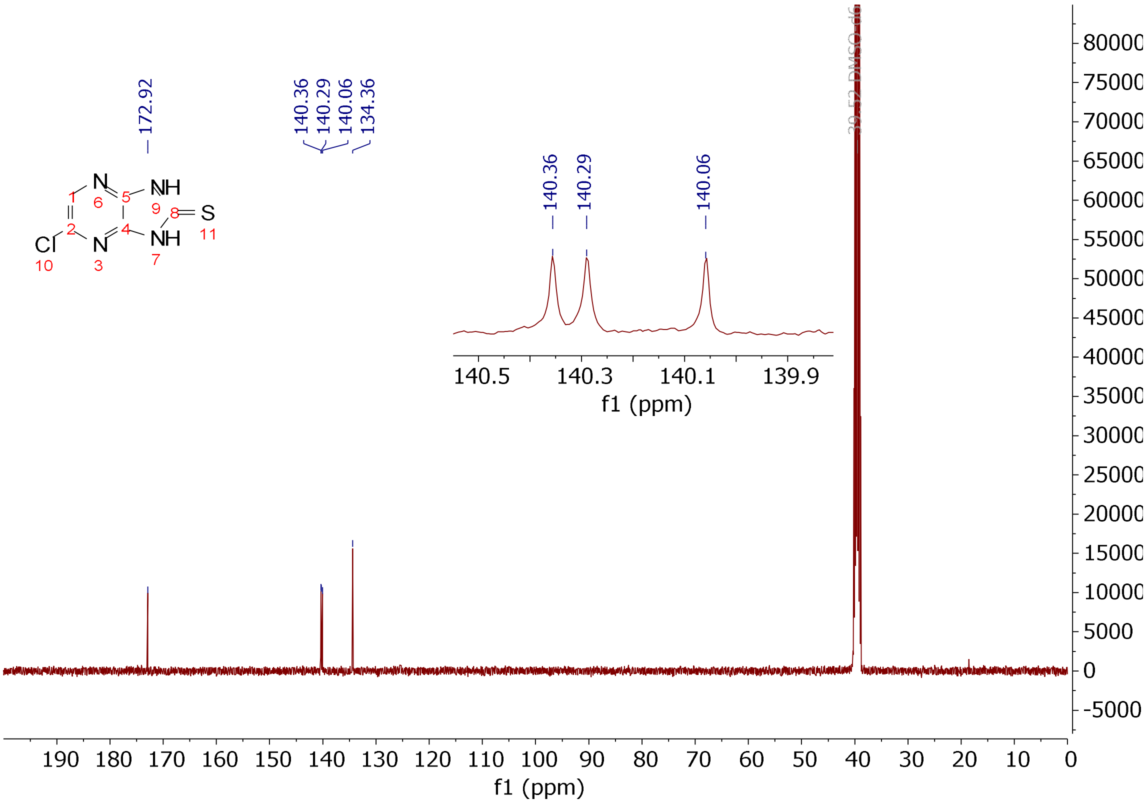
172.92
140.36
140.29
140.06
134.36
f1 (ppm)
Cl
NH
S

HRMS data:

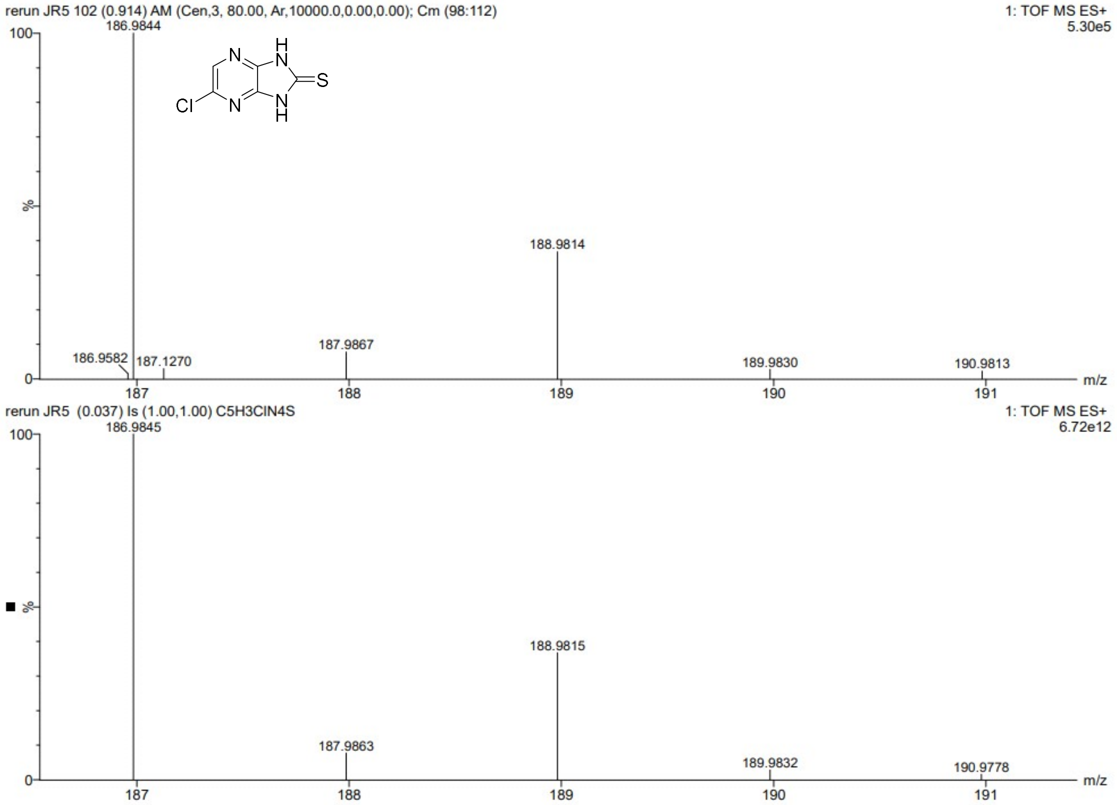

rerun JR5 102 (0.914) AM (Cen,3, 80.00, Ar,10000.0,0.00,0.00); Cm (98:112)
1: TOF MS ES+
5.30e5
186.9844
188.9814
186.9582
187.1270
187.9867
189.9830
190.9813
m/z
rerun JR5 (0.037) Is (1.00,1.00) C5H3ClN4S
1: TOF MS ES+
6.72e12
186.9845
188.9815
187.9863
189.9832
190.9778
m/z

Strategy 1_1_2: 2,3-diaminopyrazine

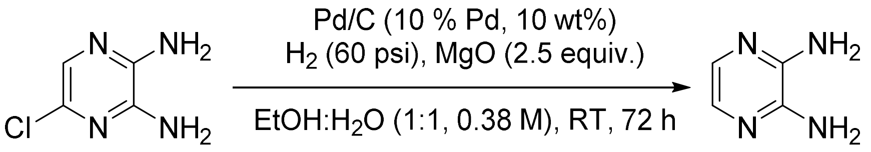

To a pressure vessel was added 2,3-diamino-5-chloropyrazine (1.0 g, 6.97 mmol), a stirrer bar, palladium on carbon (0.1 g, 10 wt. equiv., 10% Pd, un-activated), magnesium oxide (0.697 g, 17.3 mmol, 2.50 equiv., 325 mesh) and an argon-degassed ethanol : water mixture (1:1, 18.0 mL, 0.38 M). The hydrogenation vessel was sealed, and the mixture degassed twice with H_2 at 40 psi, and once at 60 psi. The mixture was then stirred at 60 psi (dynamic pressure) for 72 h at RT. The vessel was depressurised, and the contents filtered through a pad of Celite®, washing with acetone. The filtrate was then dried ($MgSO_4$) and solvent removed *in vacuo.* to provide a crude mixture as a light-beige powder (approx. 34% conversion, determined by ^{1}H NMR). Four of these reactions of this type were performed with varying degrees of conversion (13 – 34%, three at 24 h, one at 72 h). These reaction mixtures were combined and purified in a single batch of approx. 3.26 g crude, using column chromatography (95:5 DCM : MeOH) to provide 2,3-diaminopyrazine (810 mg, 7.35 mmol, 33%) as a dark orange crystalline powder.

^{1}H NMR (400 MHz, DMSO-d_6) δ 7.13 (s, 2H), 5.83 (br s, 4H).

^{13}C NMR (101 MHz, DMSO-d_6) δ 144.0, 129.0.

M.P. (205.6 °C lit.), 204.8 – 206.4 °C.

IR (cm^{-1}) 3310 (NH), 3130 (NH), 1644, 1598.

Procedure adapted from the literature[10]. Data in-line with literature data[11].

NMR data:

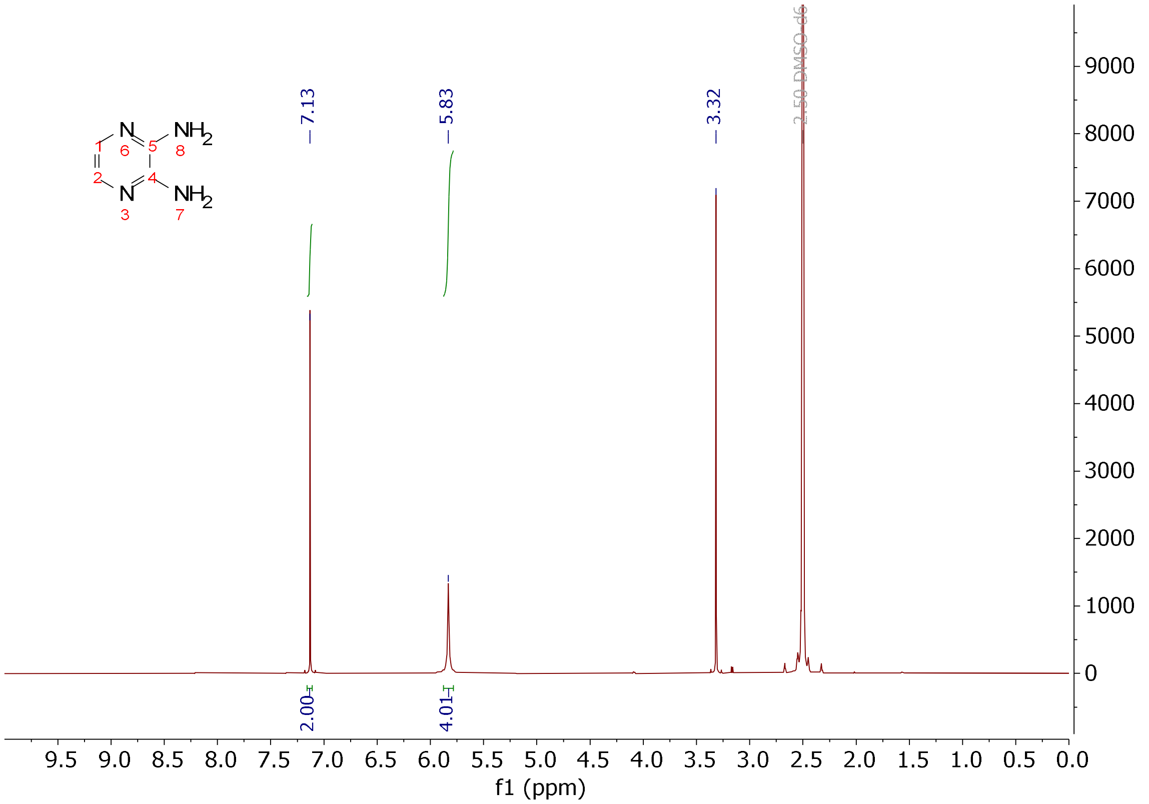

NH2
NH2
7.13
5.83
3.32
2.50 DMSO-d6
2.00
4.01
f1 (ppm)

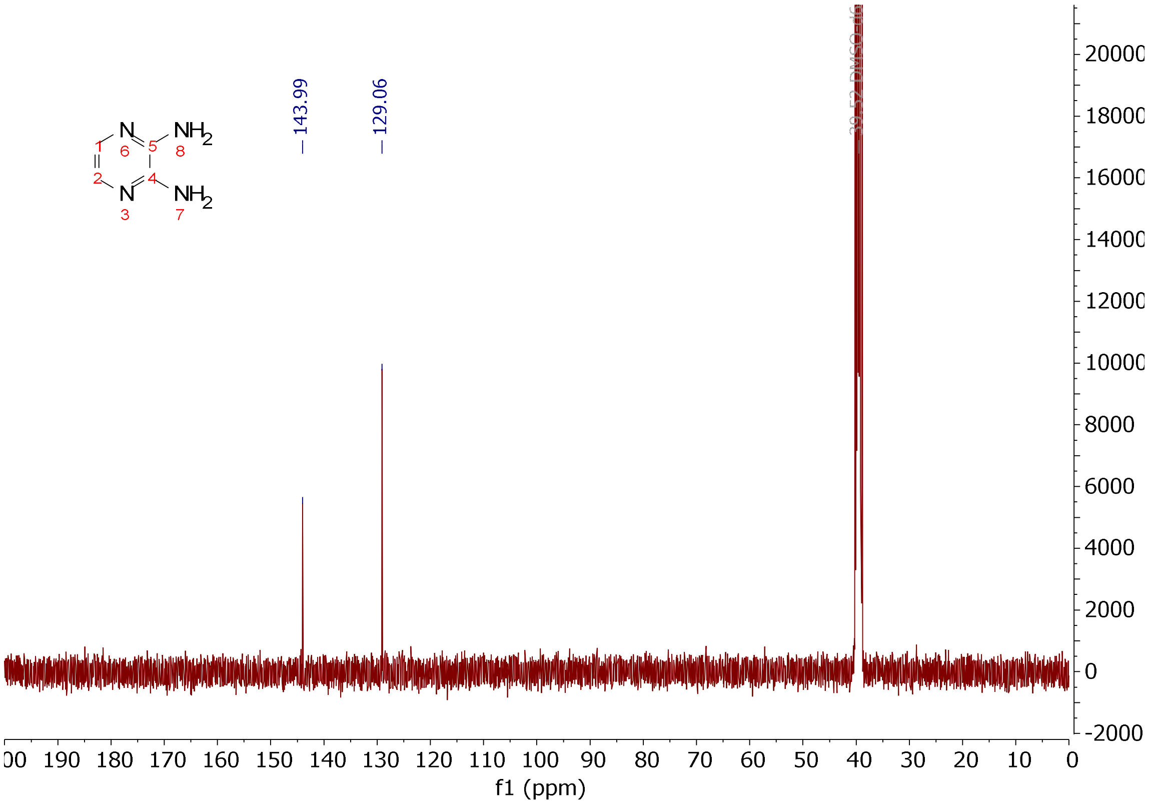

NH2
NH2
143.99
129.06
39.52 DMSO-d6
f1 (ppm)

Strategy 1_1_2_1: Imidazo[4,5-b]pyrazine-2(1,3H)-thione

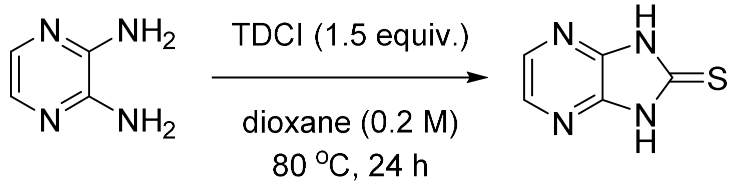

Synthesised following general procedure 2. 2,3-diaminopyrazine (0.5 g, 4.08 mmol), total TCDI (1.213 g, 1.5 equiv.). Recrystallised from EtOH : H_2O (9:1) to provide orange crystals of imidazo[4,5-*b*]pyrazine-2(1,3*H*)-thione (314 mg, 2.06 mmol, 50%).

^{1}H NMR (400 MHz, DMSO-d_6) δ 13.44 (br s, 2H), 8.07 (s, 2H).

^{13}C NMR (101 MHz, DMSO-d_6) δ 172.3*,* 141.0, 136.6.

IR (cm^{-1}): 3388 (NH), 2743 (SH), 1621 (NH bend).

M.P. (326.5 – 329 °C lit.), 322.5 – 327.8 °C.

Data in-line with literature data[11].

NMR data:

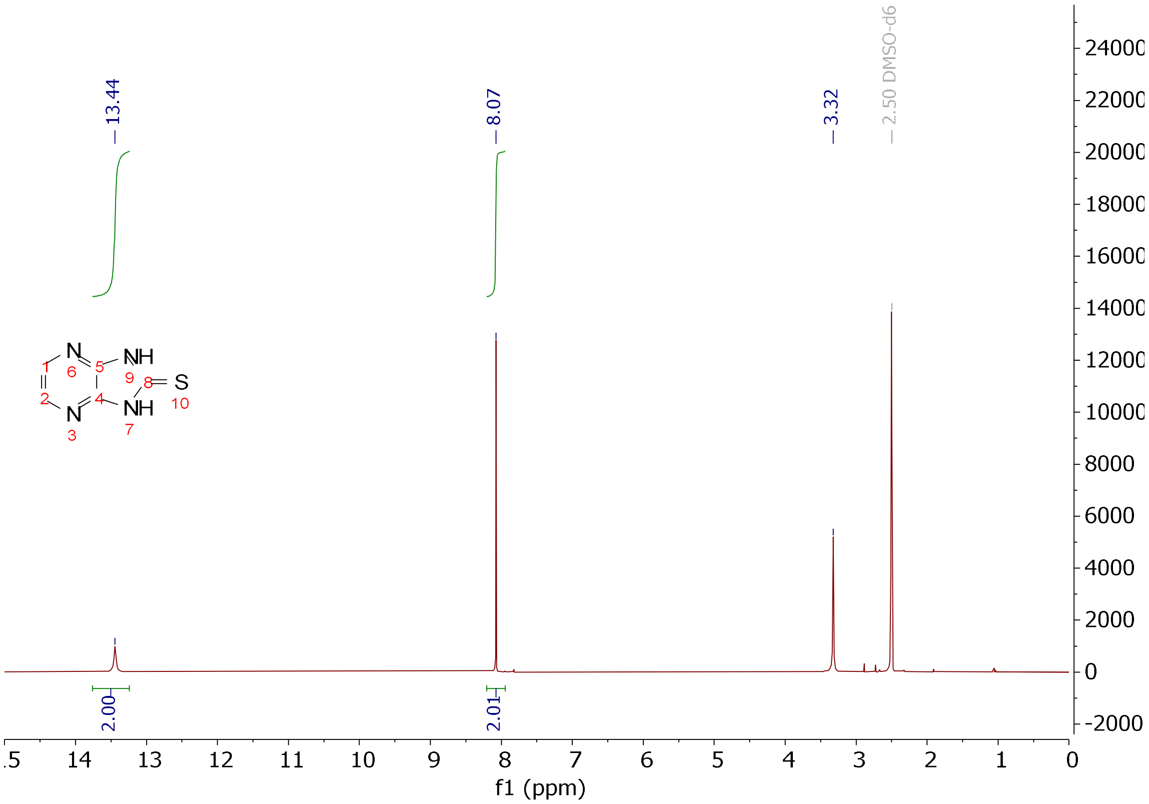

13.44
8.07
3.32
2.50 DMSO-d6
2.00
2.01
N
NH
NH
S
1
2
3
4
5
6
7
8
9
10
24000
22000
20000
18000
16000
14000
12000
10000
8000
6000
4000
2000
0
-2000
14 13 12 11 10 9 8 7 6 5 4 3 2 1 0
f1 (ppm)

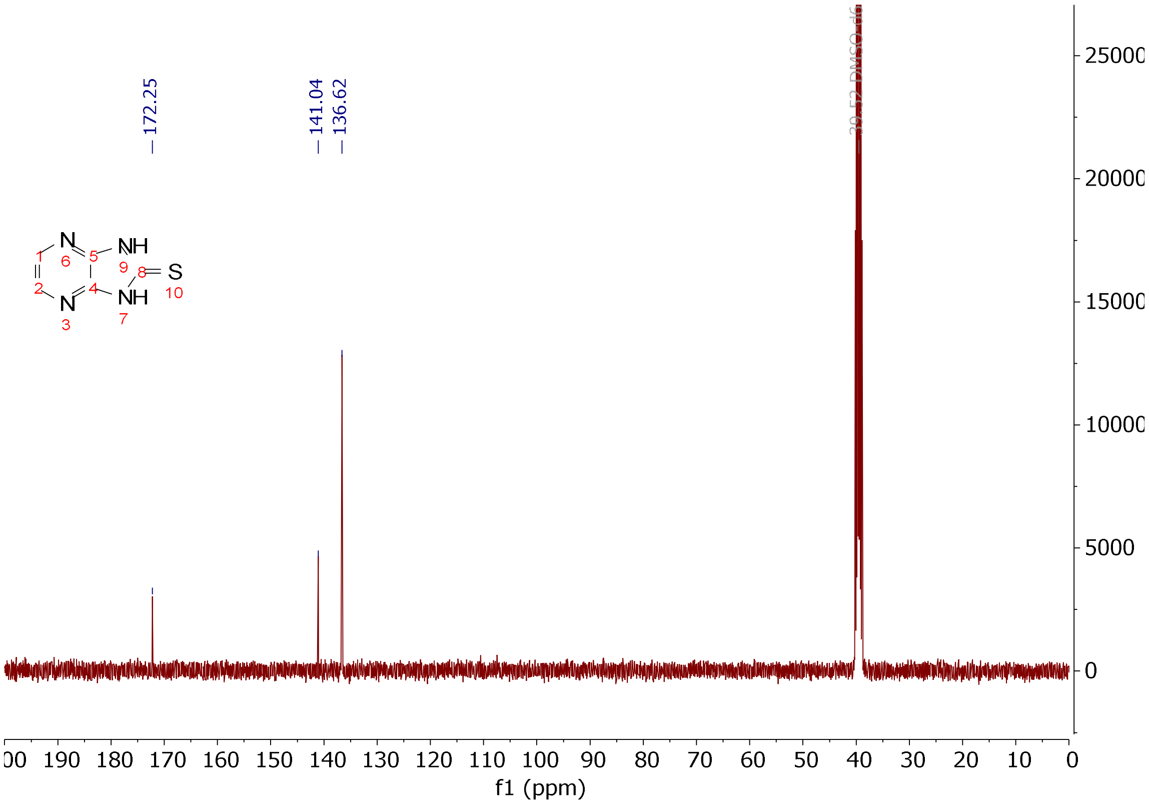

172.25
141.04
136.62
N
NH
NH
S
1
2
3
4
5
6
7
8
9
10
25000
20000
15000
10000
5000
0
190 180 170 160 150 140 130 120 110 100 90 80 70 60 50 40 30 20 10 0
f1 (ppm)

Strategy 2: 4,5-diamino-6-chloropyrimidine

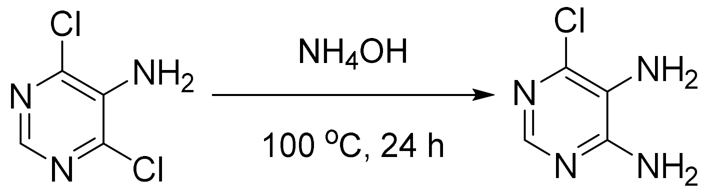

To four 30 mL microwave vials was added 5-amino-4,6-dichloropyrimidine (1.0 g, 6.09 mmol, 0.25 g per vial), a stirrer bar and ammonium hydroxide solution (25% soln., 12.5 mL per vial, 0.12 M). The vials were sealed and heated to 100 °C in a sand bath over 24 h. The vials were allowed to cool to RT and the contents transferred to a separatory funnel, washing with EtOAc. The organic and aqueous layers were separated, and the aqueous layer extracted with EtOAc (6x 20 mL). The organic layers were collected and dried ($MgSO_4$) to provide 4,5-diamino-6-chloropyrimidine (762 mg, 5.27 mmol, 87%) as an off-white solid. If the crude was impure, it was triturated with DCM to provide 4,5-diamino-6-chloropyrimidine as an off-white solid.

^{1}H NMR (500 MHz, DMSO-d_6) δ 7.63 (s, 1H), 6.72 (br s, 2H), 4.94 (br s, 2H).

^{13}C NMR (126 MHz, DMSO-d_6) δ 153.5, 145.8, 137.5, 123.1.

IR (cm^{-1}): 3331 (NH), 3269 (NH), 1671 (NH bend).

M.P. 253.6 – 255.6 °C (deg.).

Followed literature procedure, data in-line with literature data[12].

NMR data:

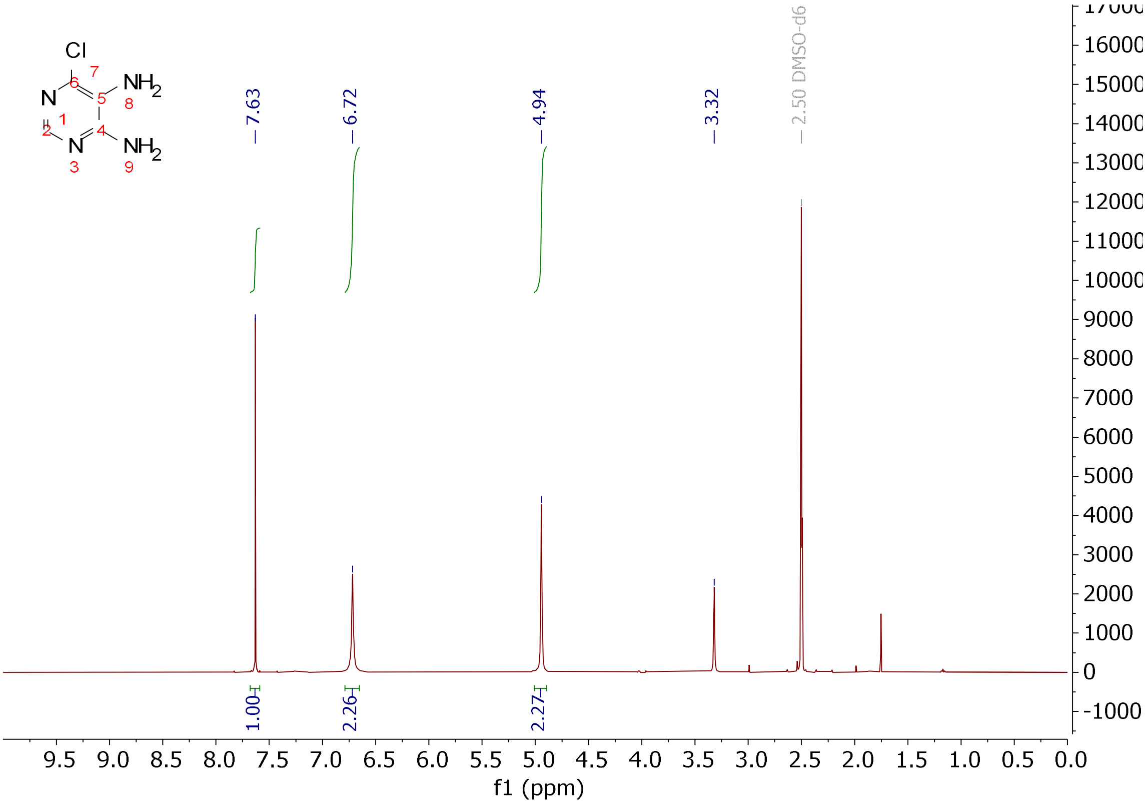

Cl
NH2
N
N
NH2
7.63
6.72
4.94
3.32
2.50 DMSO-d6
1.00
2.26
2.27
f1 (ppm)

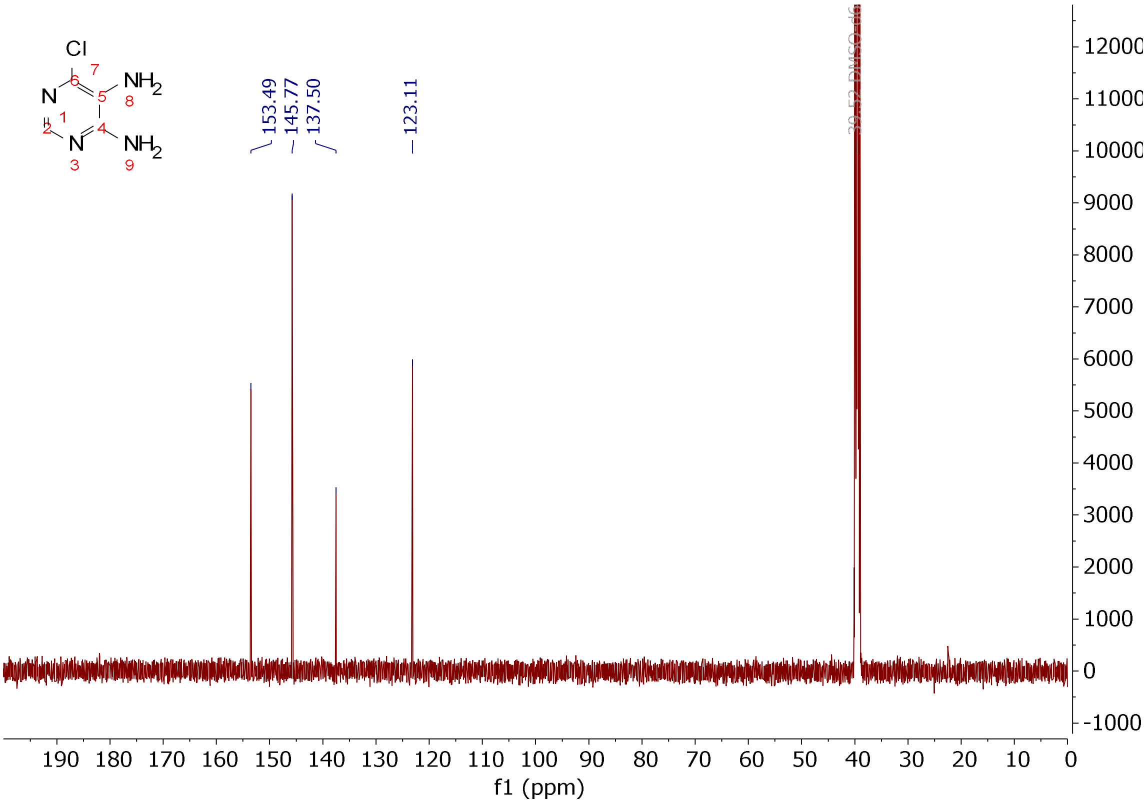

Cl
NH2
N
N
NH2
153.49
145.77
137.50
123.11
f1 (ppm)

Strategy 2_1: 6-Chloroimidazo[4,5-d]pyrimidine-8(7,9H)-thione

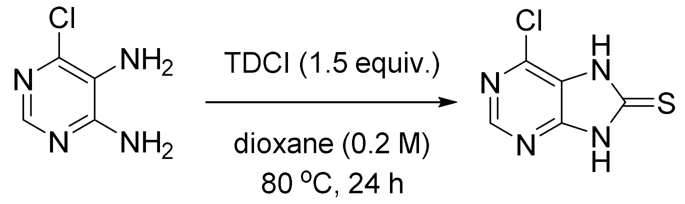

Synthesised following general procedure 2. 4,5-diamino-6-chloropyrimidine (0.5 g, 3.45 mmol), total TCDI (0.616 g, 1.5 equiv.). Dioxane (17.25 mL, 0.2 M). Provided 6-chloroimidazo[4,5-*d*]pyrimidine-8(7,9*H*)-thione as a yellow solid (173 mg, 0.92 mmol, 27%)

^{1}H NMR (400 MHz, DMSO-d_6) δ 13.71 (br s, 2H), 8.52 (s, 1H).

^{13}C NMR (101 MHz, DMSO-d_6) δ 172.0, 152.9, 151.5, 136.0, 123.0.

IR (cm^{-1}): 2782 (SH), 1602 (NH bend).

M.P. 205.2 °C (dec.).

MS ES+ m/z calcd for C5H3ClN4S (M+H)+: 188.9815, found: 188.9814.

NMR data:

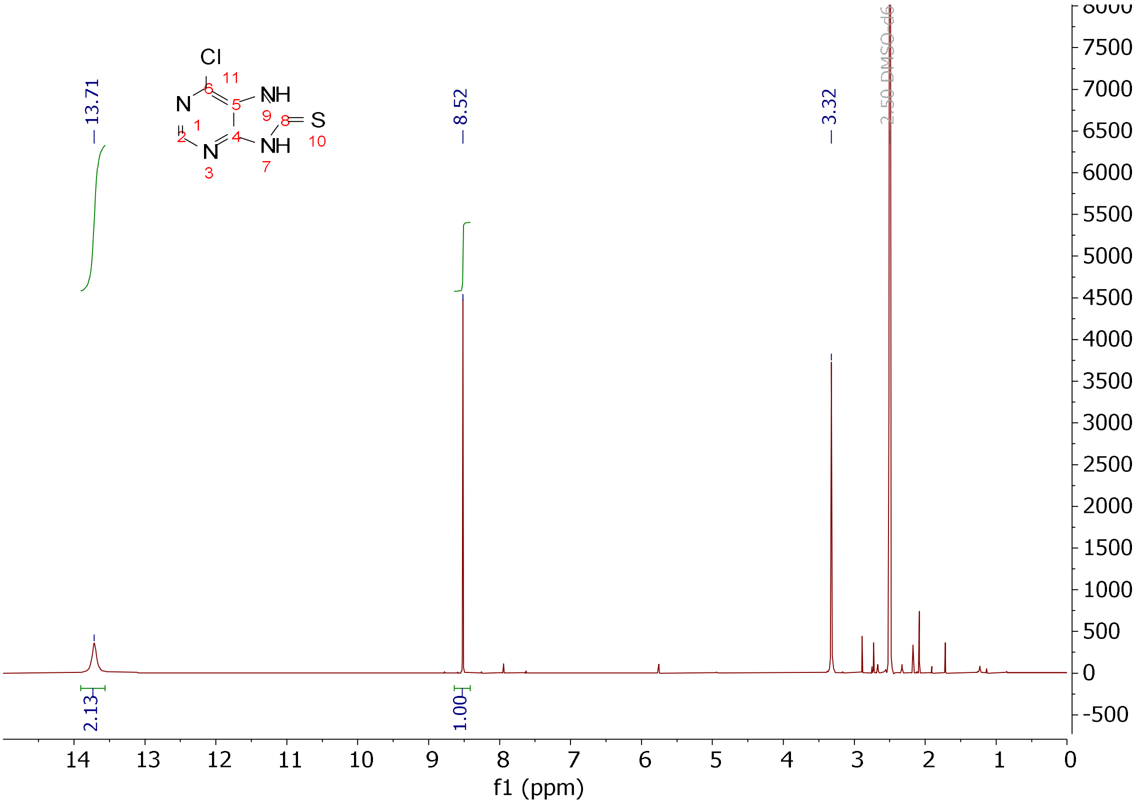
Cl
N
NH
S
NH
N
13.71
8.52
3.32
2.50 DMSO-d6
2.13
1.00
f1 (ppm)

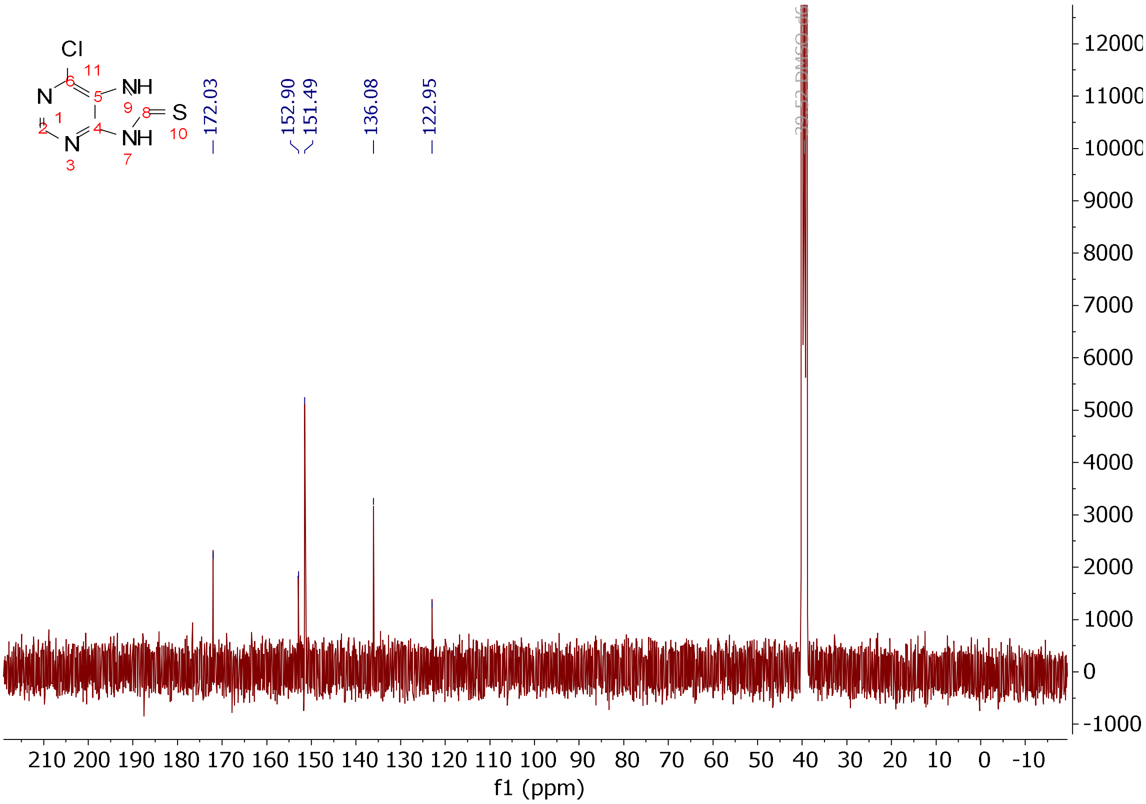
Cl
N
NH
S
NH
N
172.03
152.90
151.49
136.08
122.95
39.52 DMSO-d6
f1 (ppm)

Strategy 3: Imidazo[4,5-b]pyridine-2(1,3H)-thione

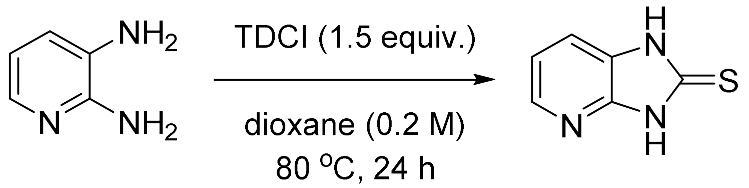

Synthesised following general procedure 2. 2,3-diaminopyridine (0.5 g, 4.58 mmol), total TCDI (1.22 g, 1.5 equiv.). Recrystallised from EtOH : H_2O (9:1) to provide off-white crystals of imidazo[4,5-*b*]pyridine-2(1,3*H*)-thione (261 mg, 1.73 mmol, 37%).

^{1}H NMR (400 MHz, DMSO-d_6) δ 13.11 (br s, 1H), 12.70 (br s, 1H), 8.10 (dd, J = 5.0, 1.4 Hz, 1H), 7.47 (dd, J = 7.9, 1.4 Hz, 1H), 7.12 (dd, J = 7.9, 5.0 Hz, 1H).

^{13}C NMR (101 MHz, DMSO-d_6) δ 169.8, 146.5, 142.3, 125.4, 118.1, 116.2.

IR (cm^{-1}): 3141 (NH), 3065 (NH), 2572 (SH), 1614 (NH bend).

M.P. (322 – 324 ºC lit.), 321.8 – 323.8 ºC.

Data is in line with literature data[13].

NMR data:

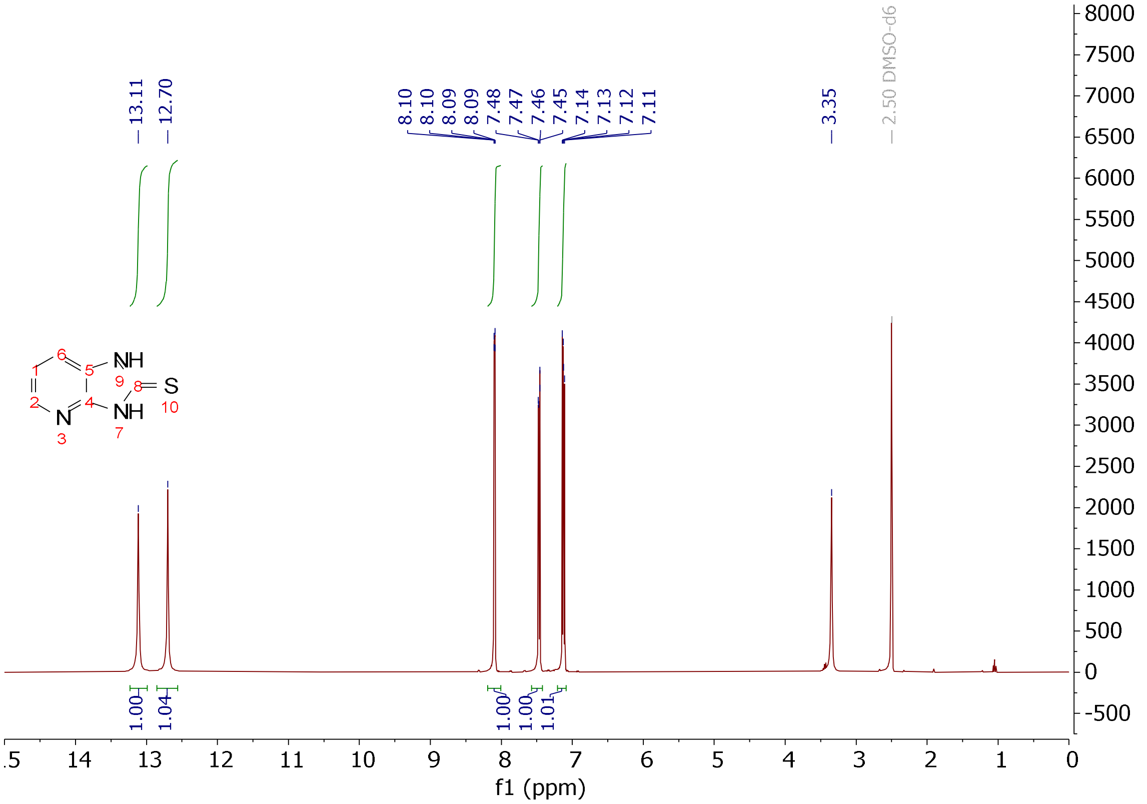

13.11
12.70
8.10
8.10
8.09
8.09
7.48
7.47
7.46
7.45
7.14
7.13
7.12
7.11
3.35
2.50 DMSO-d6
1.00
1.04
1.00
1.00
1.01
NH
S
NH
N
f1 (ppm)

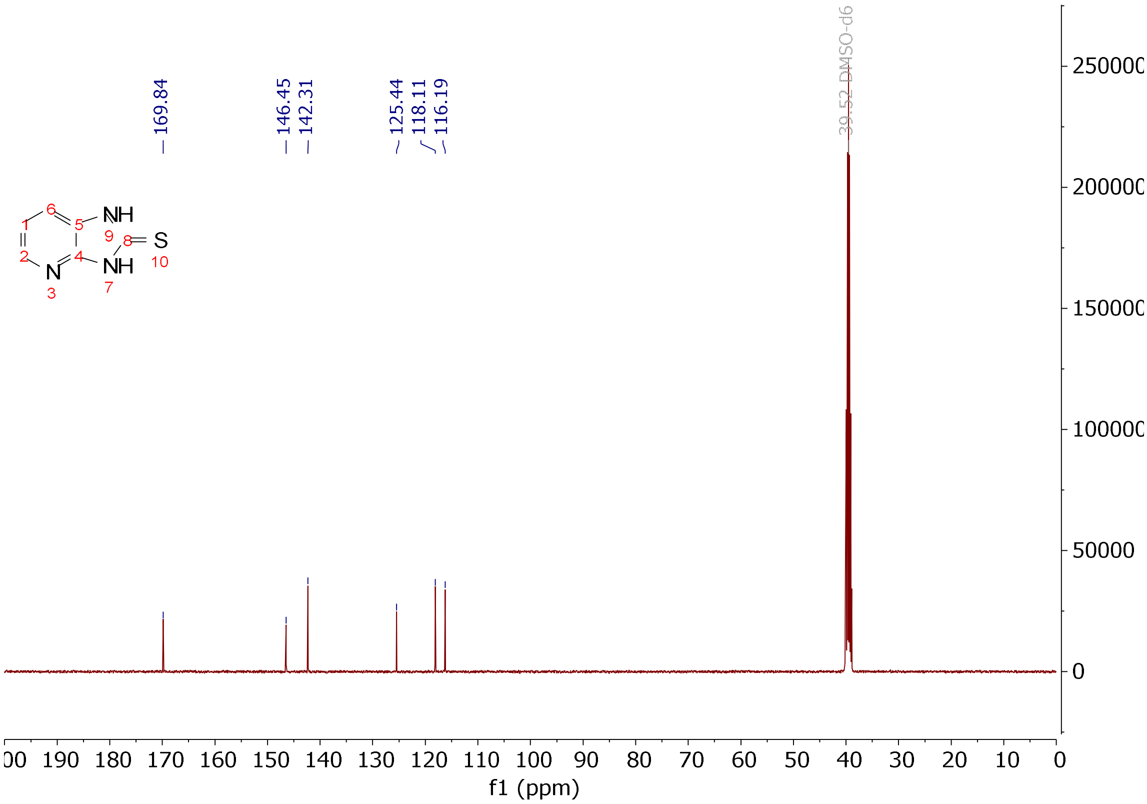

169.84
146.45
142.31
125.44
118.11
116.19
39.52 DMSO-d6
NH
S
NH
N
f1 (ppm)

Strategy 4: Imidazo[4,5-d]pyrimidine-8(7,9H)-thione

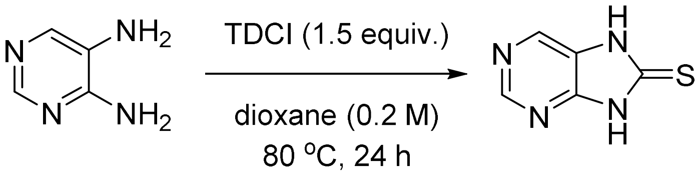

Synthesised following general procedure 2. 4,5-diaminopyrimidine (0.25 g, 2.27 mmol), total TCDI (0.606 g, 1.5 equiv.). Recrystallised from H_2O to provide an orange powder of imidazo[4,5-*d*]pyrimidine-8(7,9*H*)-thione (77.0 mg, 0.5 mmol, 22%).

^{1}H NMR (400 MHz, DMSO-d_6) δ 13.68 (br s, 1H), 13.15 (br s, 1H), 8.75 (s, 1H), 8.50 (s, 1H).

^{13}C NMR (101 MHz, DMSO-d_6) δ 172.0, 152.4, 151.2, 133.4, 125.0.

IR (cm^{-1}): 3429 (NH), 2763 (SH), 1614 (NH bend).

M.P. 310.5 – 314.4 °C (dec.).

MS ES+ m/z calculated for C5H4N4S (M+H)+: 153.0235, found: 153.0237.

NMR data:

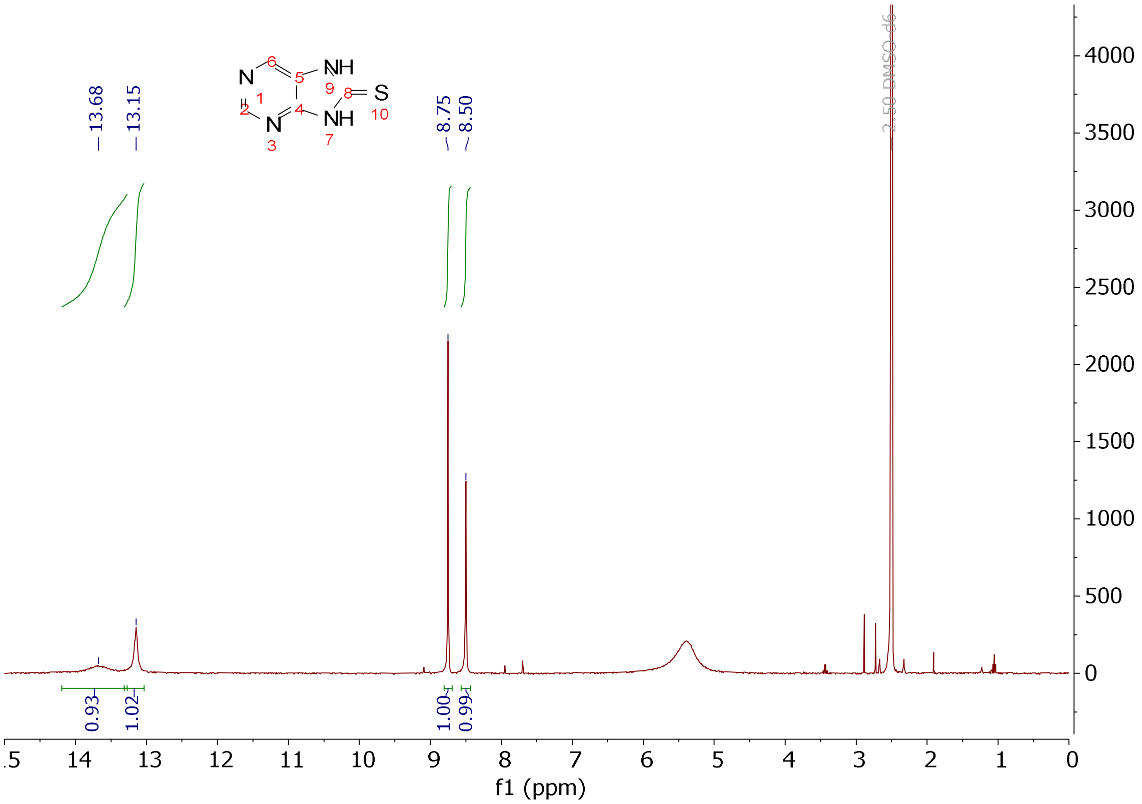
13.68
13.15
8.75
8.50
2.50 DMSO-d6
0.93
1.02
1.00
0.99
f1 (ppm)

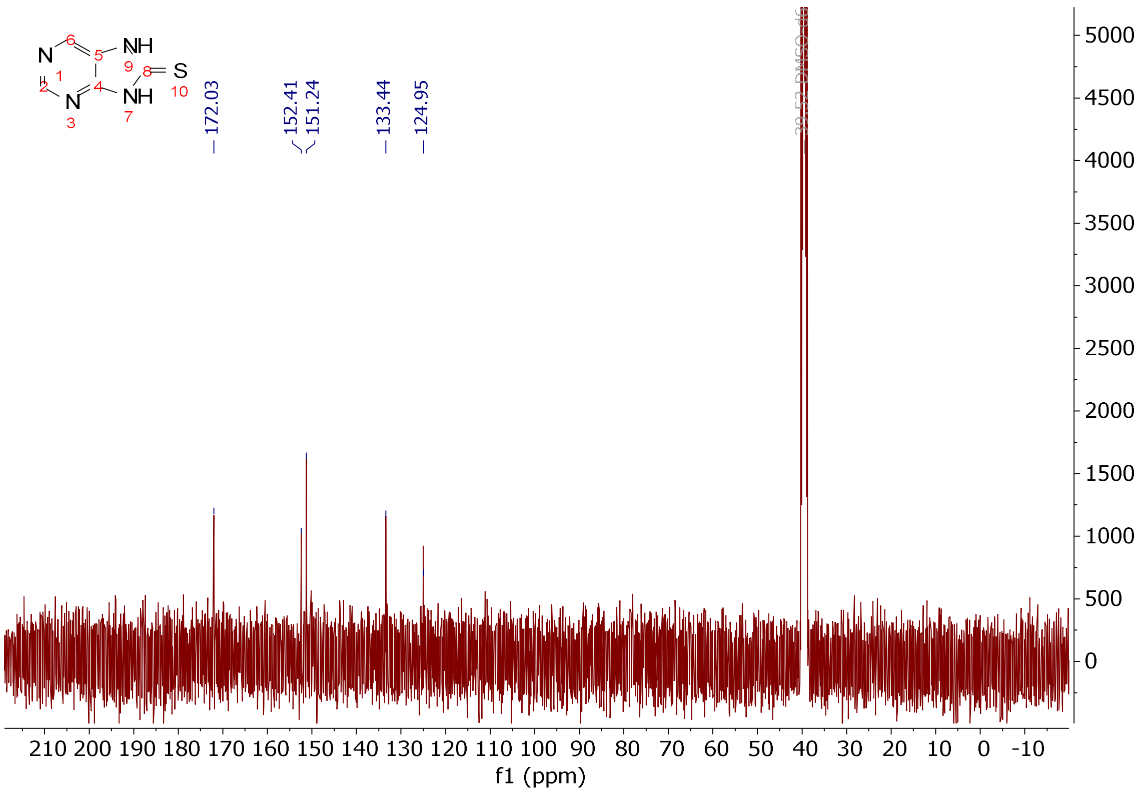
172.03
152.41
151.24
133.44
124.95
f1 (ppm)

Strategy 5: Imidazo[4,5-c]pyridine-2(1,3H)-thione

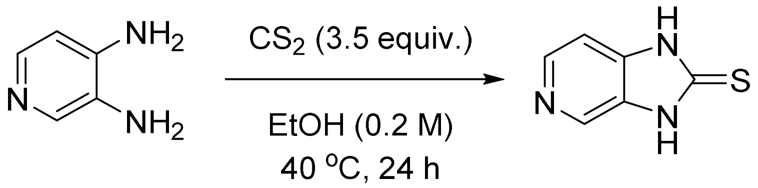

To a 50 mL RBF was added 3,4-diaminopyridine (0.25 g, 2.29 mmol) and a stirrer bar. To this was added EtOH (11.5 mL, 0.2 M) and stirred to dissolve. To the stirred solution was added CS_2 (5 mL, 3.5 equiv.) and the mixture heated with stirring to 40 °C for 24 h. The mixture was allowed to cool to RT then 0 °C. The solid precipitate was collected and filtered to provide crude imidazo[4,5-*c*]pyridine-2(1,3*H*)-thione an off-white solid.

Taken through as crude material (ca. 76% conversion as indicated by ^{1}H NMR).

^{1}H NMR (400 MHz, DMSO-d_6) δ 12.80 (br s, 2H), 8.37 (s, 1H), 8.24 (d, J = 5.2 Hz, 1H), 7.17 (d, J = 5.2 Hz, 1H).

Adapted from literature procedure, in-line with literature data[11].

NMR data:

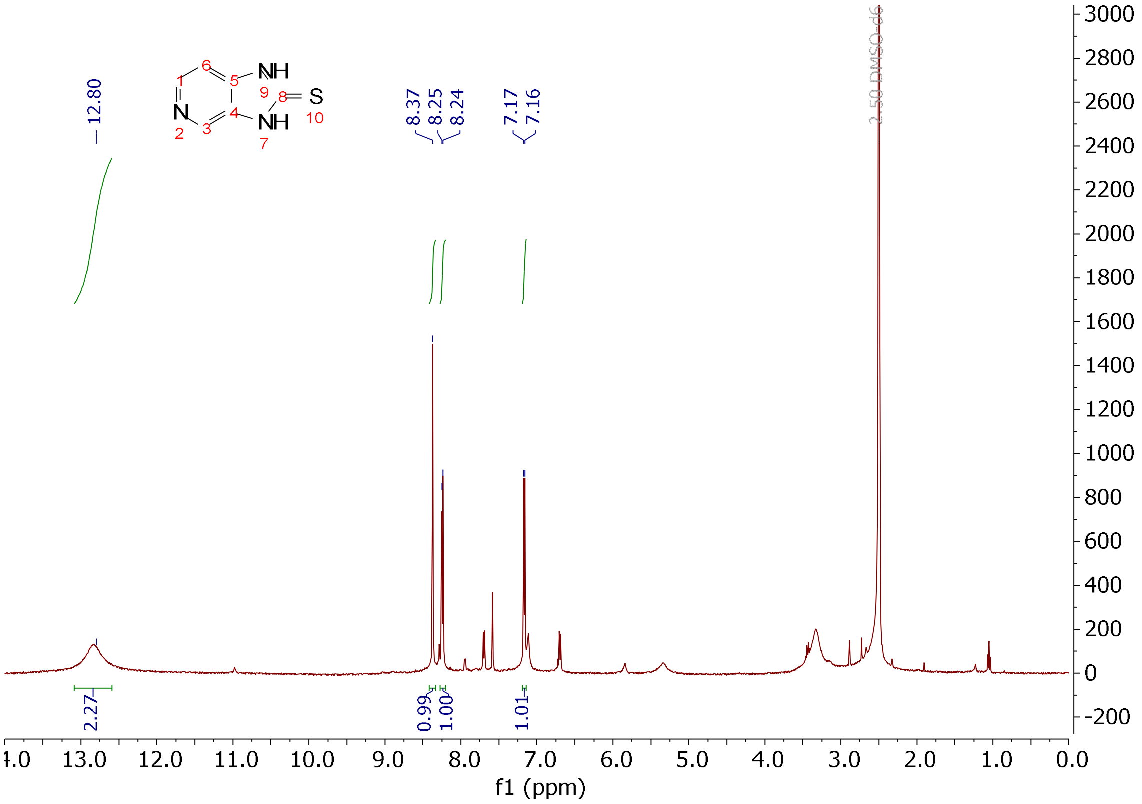

NH
S
NH
N
12.80
8.37
8.25
8.24
7.17
7.16
2.50 DMSO-d6
2.27
0.99
1.00
1.01
f1 (ppm)

Strategy 6: 2,6-diketopiperazine

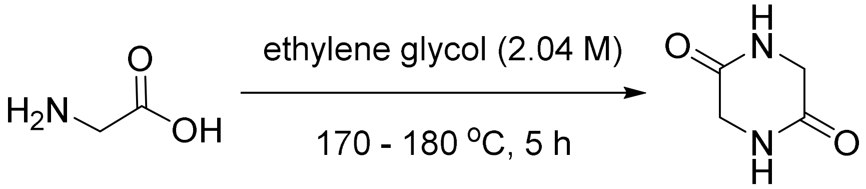

To a 500 mL RBF was added glycine (20 g, 0.266 mol) and ethylene glycol (130 mL, 2.04 M). The mixture was heated to 170-180 °C for 5 h, left to cool to RT and placed in a freezer overnight. The mixture was filtered and washed with methanol to yield a fine brown solid. This was recrystallised from water to give 2,6-diketopiperazine as a brown crystalline solid (6.88 g, 60.3 mmol, 45%).

^{1}H NMR (400 MHz, DMSO- d_6) δ 8.00 (br s, 2H), 3.70 (d, J = 2.0 Hz, 4H).

^{13}C NMR (101 MHz, DMSO- d_6) δ 166.1, 44.3.

IR (cm^{-1}) 3161 (NH), 1664 (C=O).

Followed literature procedure, data is in accordance with literature data[14].

NMR data:

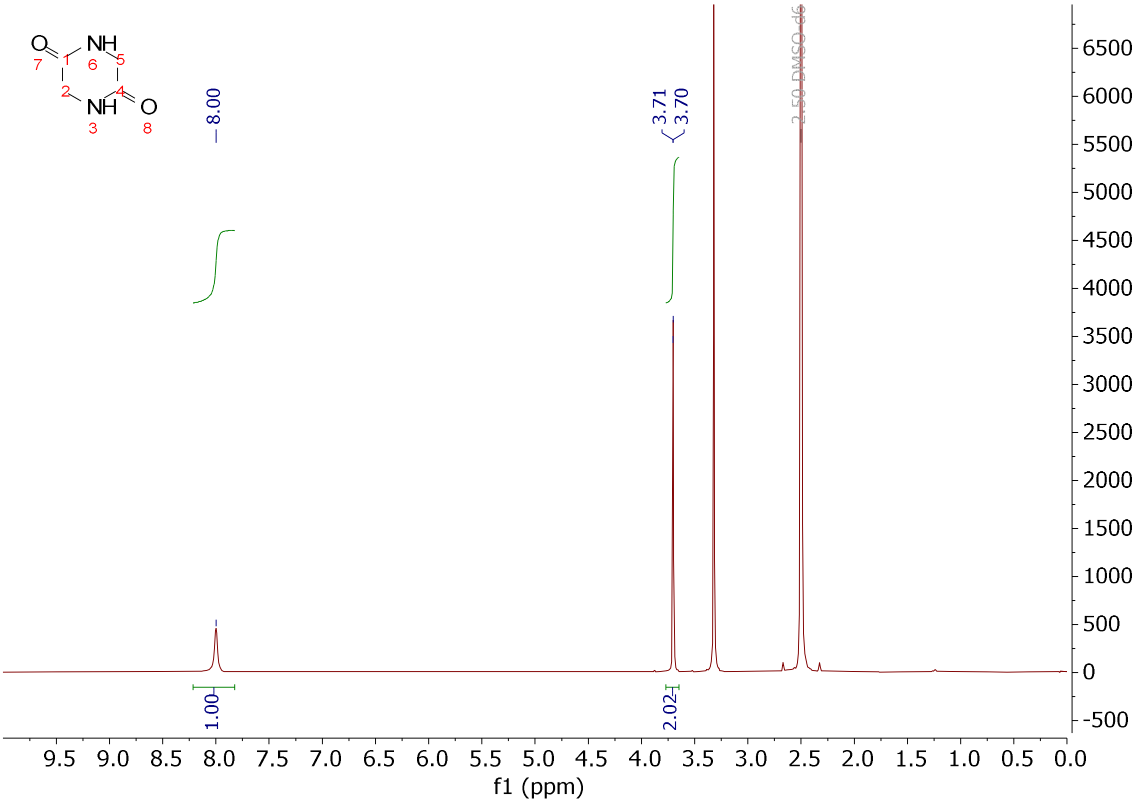

NH
O
NH
O
8.00
3.71
3.70
2.50 DMSO-d6
1.00
2.02
f1 (ppm)

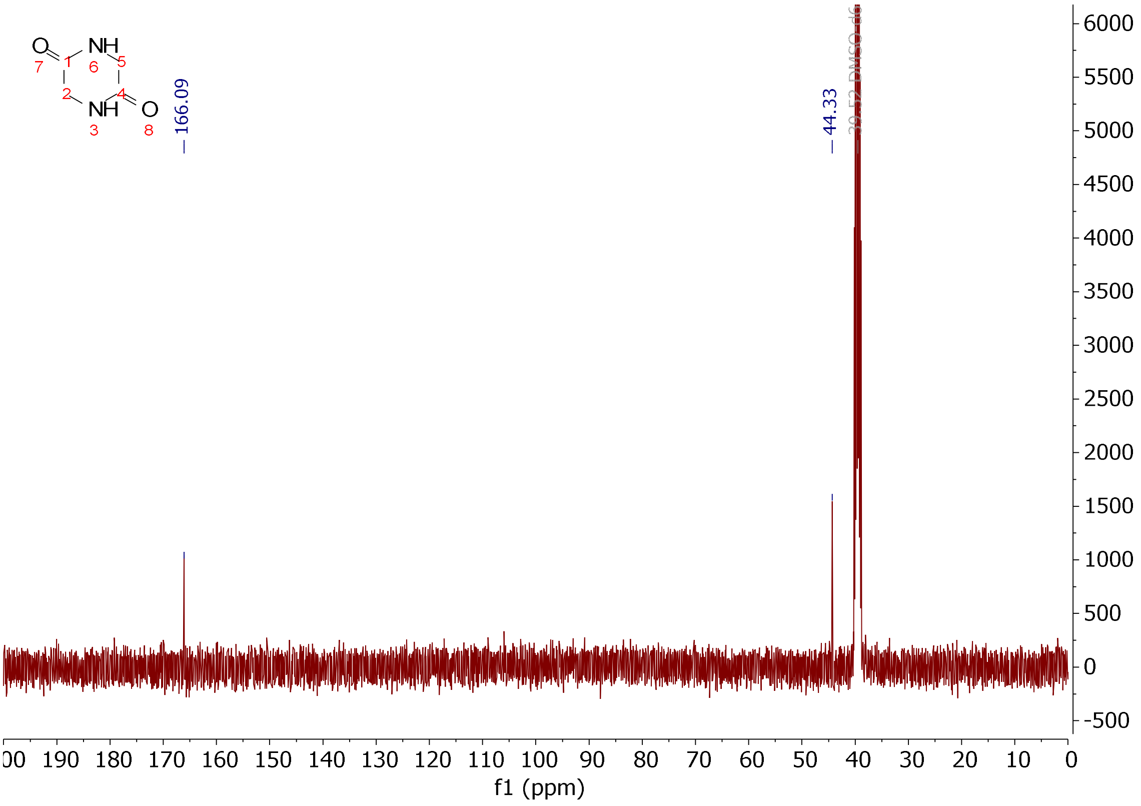

NH
O
NH
O
166.09
44.33
39.52 DMSO-d6
f1 (ppm)

Strategy 6_1: Tetrachloropyrazine

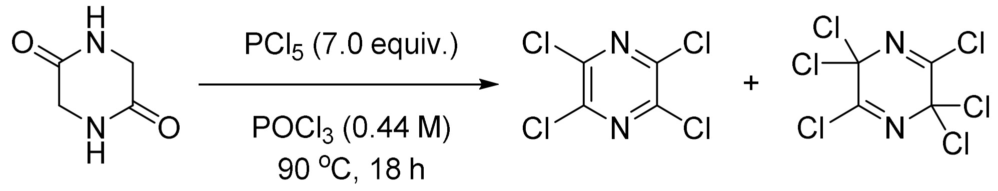

To a 250 mL RBF was added 2,6-diketopiperazine (5.00 g, 0.044 mol), phosphorus pentachloride (63 g, 7.0 equiv.), phosphorus oxychloride (100 mL, 0.44 M) and a stirrer bar, this was placed under nitrogen atmosphere. The vessel was heated at 90 °C for 18 h. The unreacted phosphorus oxychloride and phosphorus pentachloride were distilled off at 200 °C (increasing in 10 °C increments). The distillation continued until no more liquid distillate came off (approx. 4 h total, 3 h at 200 °C). The vessel was allowed to cool to RT and the solid reaction mixture was dissolved in hexane (50 mL) and aqueous HCl (1 M, 50 mL) was added to the reaction vessel, the mixture was transferred to a separatory funnel and the aqueous layer was extracted with hexane (3x 30 mL). The organic layers were collected and filtered through a pad of Florisil® (100-200 mesh), the filtrate was washed with aqueous NaOH (1 M, 20 mL) and brine (20 mL). The hexane was then removed *in vacuo*. The residue was purified *via* column chromatography (*n*-hexane) to yield tetrachloropyrazine as a white crystalline solid (3.0 g, 0.014 mol, 32%).

^{13}C NMR (101 MHz, DMSO-d_6) δ 143.8.

M.P. approx. 100 °C (lit.), 99.6 – 100.3 °C.

Adapted from literature procedure, data is in accordance with literature data[15].

2,3,3,5,6,6-Hexachloro-3,6-dihydropyrazine: Isolated as an off-white solid.

IR (cm^{-1}) 3201, 2834, 1725, 1645.

M.P. approx. 100°C (lit.) 102 – 103 °C.

Data in line with literature data[31].

NMR data:

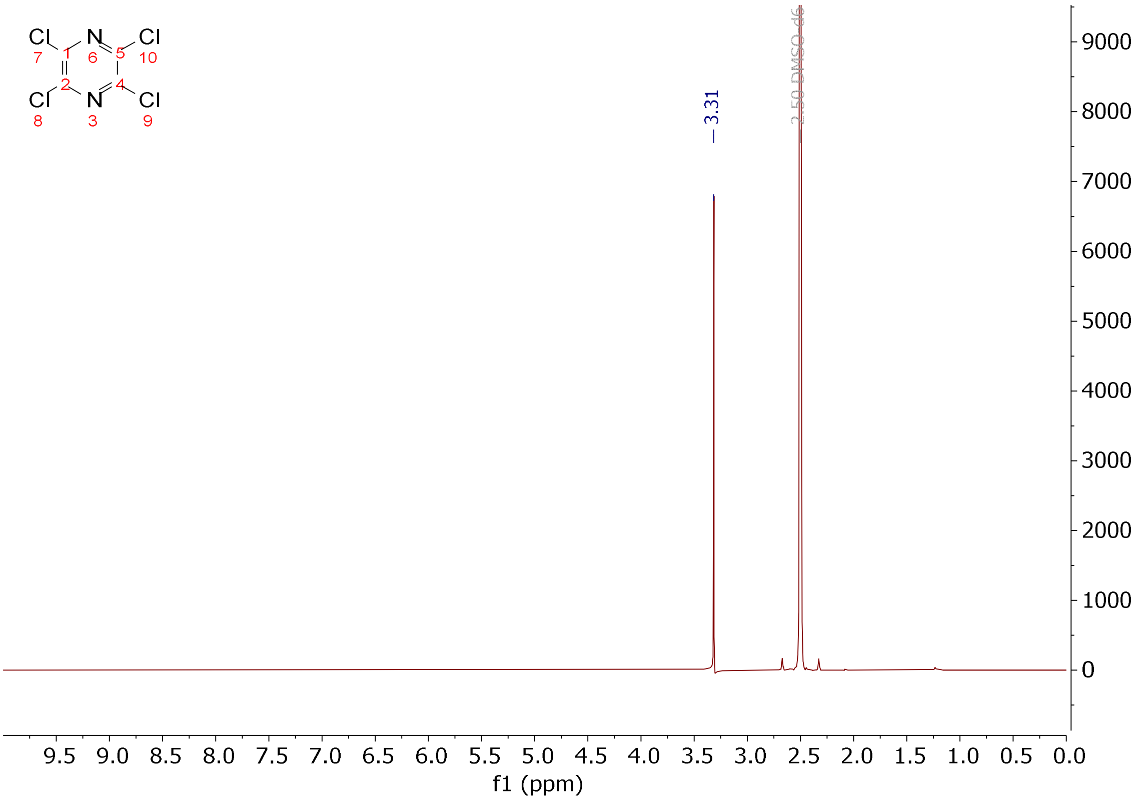

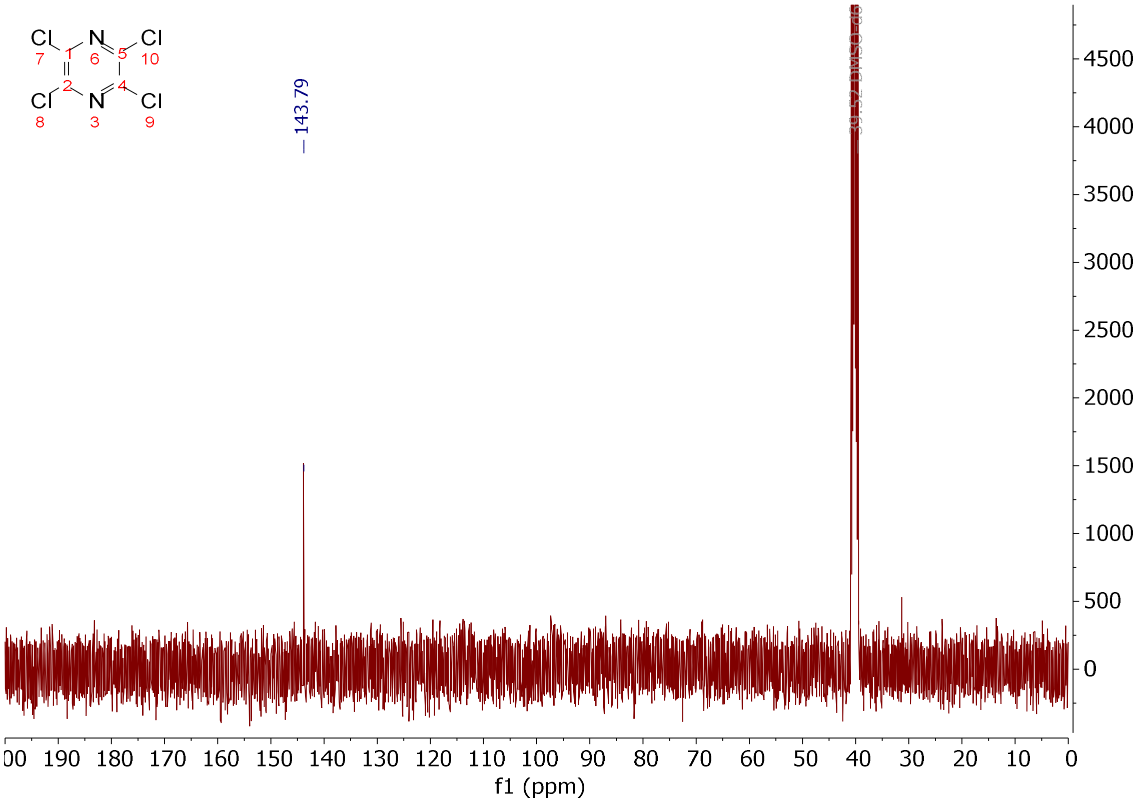

Cl
N
Cl
7
1
6
5
10
2
4
Cl
N
Cl
8
3
9
143.79
39.52 DMSO-d6
4500
4000
3500
3000
2500
2000
1500
1000
500
0
00 190 180 170 160 150 140 130 120 110 100 90 80 70 60 50 40 30 20 10 0
f1 (ppm)

Strategy 6_1_1: 2,3-diamino-5,6-dichloropyrazine

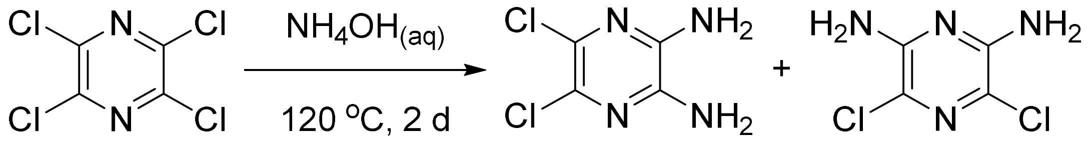

To five 30 mL microwave vials was added in equal portions tetrachloropyrazine (2.5 g, 11.50 mmol, 0.5 g/vial, 2.3 mmol/vial), a stirrer bar and ammonium hydroxide solution (25%, 12 mL/vial). The vials were sealed with a Teflon cap and stirred at 120 °C for two days in a sand bath. The vials were allowed to cool to RT and diluted with EtOAc, transferred to a separatory funnel, and neutralised with acetic acid. The organic layer was separated, and the aqueous layer extracted with EtOAc (3x 20 mL). The organic layers were collected and treated with $NaHCO_3$ (sat. soln.) until gas evolution was no longer observed. Organic phase was dried (Na_2SO_4), and solvent removed *in vacuo.* The solid recovered was purified by column chromatography (8:2 -> 7:3 Hexane : EtOAc) and yielded the desired 2,3-diamino-5,6-dichloropyrazine as an off-white solid (641 mg, 3.58 mmol, 31%).

^{1}H NMR (400 MHz, DMSO-d_6) δ 6.55 (br s, 4H).

^{13}C NMR (101 MHz, DMSO-d_6) δ 142.6, 126.2.

IR (cm^{-1}) 3435 (NH), 3294 (NH).

M.P. 275.7 – 276.1 °C.

MS ES+ m/z calcd for $C_4H_4Cl_2N_4$ $(M+H)^+$: 179.9909, found: 179.9910.

NMR data:

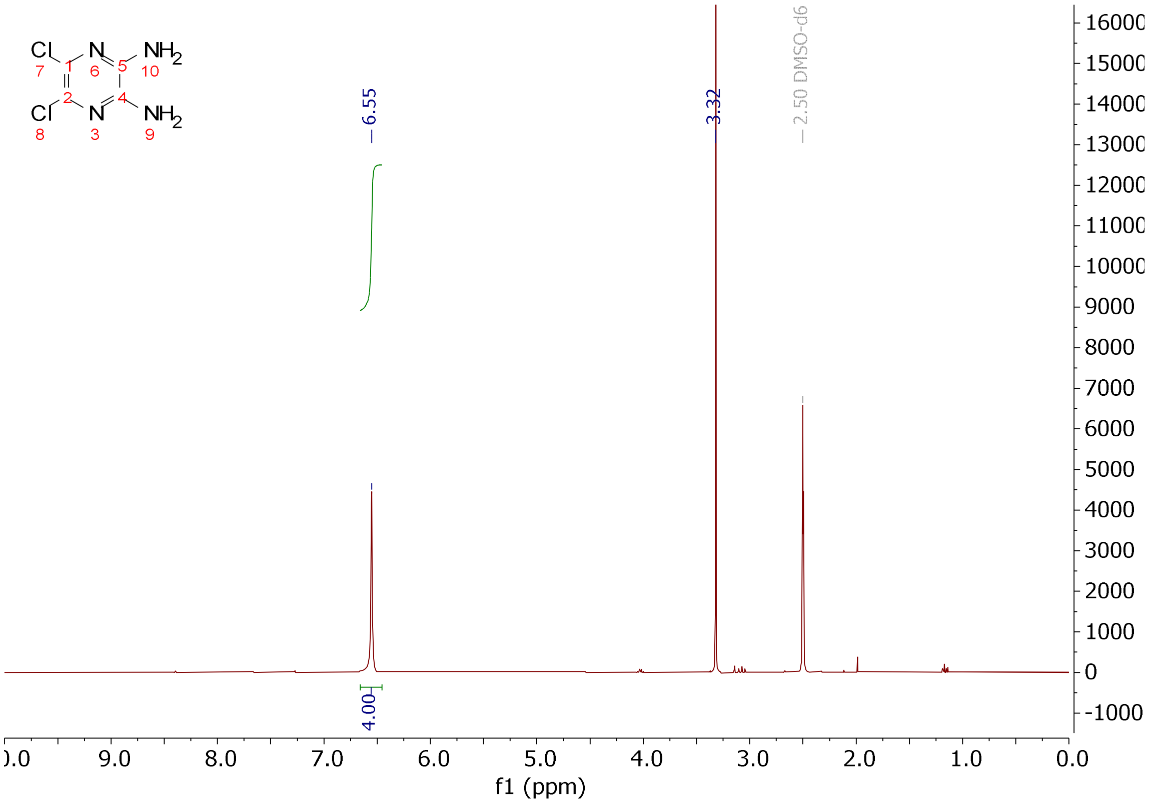
Cl
N
NH2
7
1
6
5
10
2
4
Cl
N
NH2
8
3
9
6.55
3.32
2.50 DMSO-d6
4.00
f1 (ppm)

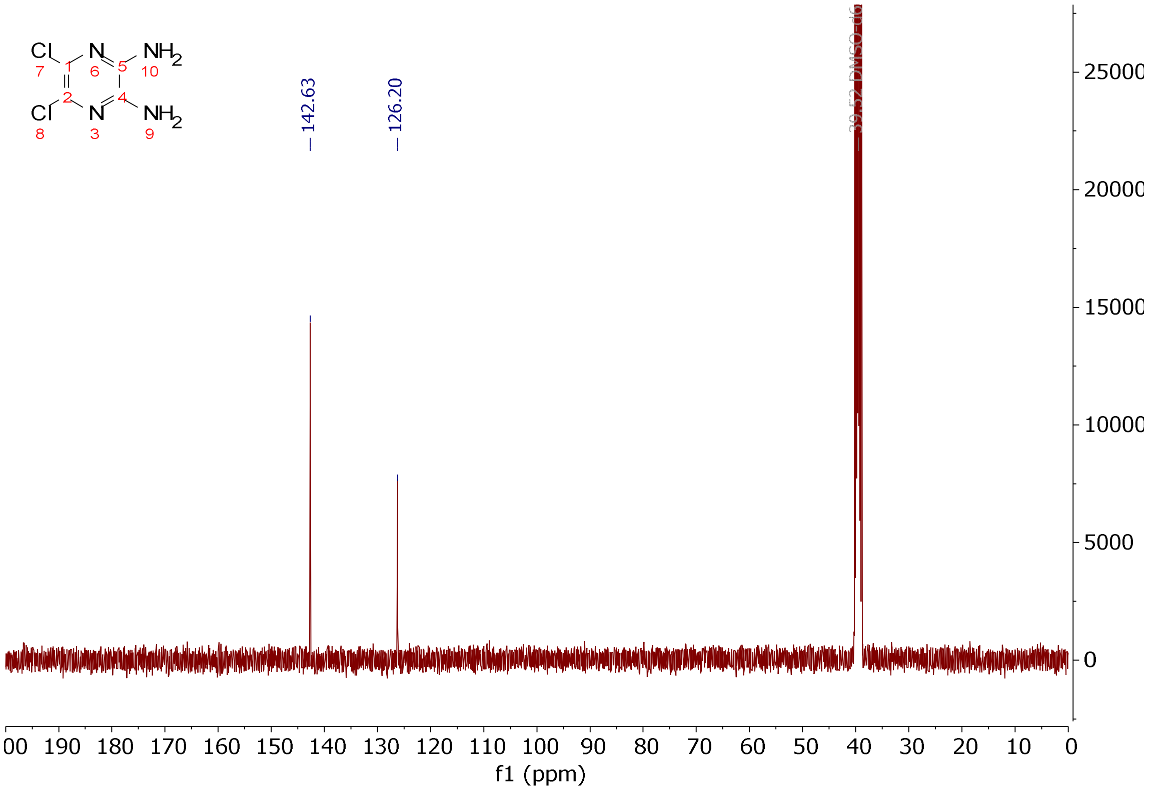
Cl
N
NH2
7
1
6
5
10
2
4
Cl
N
NH2
8
3
9
142.63
126.20
f1 (ppm)

HRMS data:

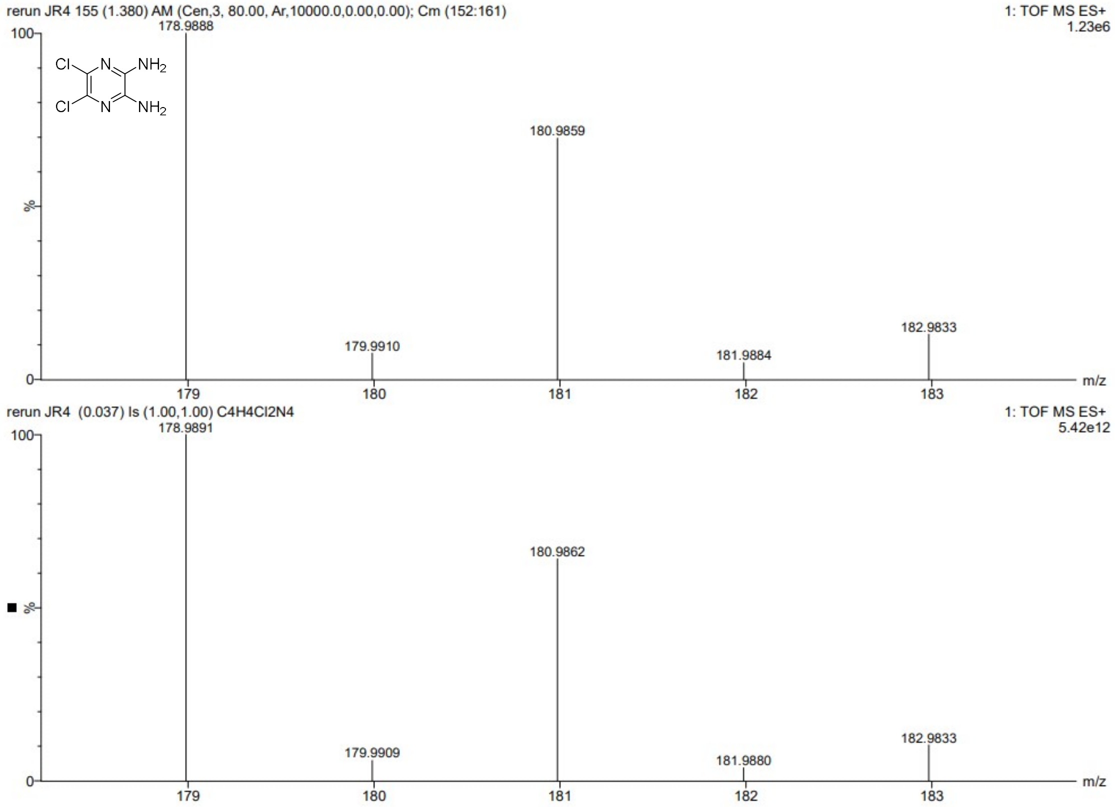
rerun JR4 155 (1.380) AM (Cen,3, 80.00, Ar,10000.0,0.00,0.00); Cm (152:161)
1: TOF MS ES+
1.23e6
Cl
N
NH2
Cl
N
NH2
178.9888
180.9859
179.9910
181.9884
182.9833
100
%
0
179
180
181
182
183
m/z
rerun JR4 (0.037) Is (1.00,1.00) C4H4Cl2N4
1: TOF MS ES+
5.42e12
178.9891
180.9862
179.9909
181.9880
182.9833
100
%
0
179
180
181
182
183
m/z

2,6-diamino-3,5-dichloropyrazine was also isolated as an off-white solid.

^{1}H NMR (400 MHz, DMSO-d_6) δ 6.33 (br s, 4H).

^{13}C NMR (101 MHz, DMSO-d_6) δ 150.1, 113.9.

IR (cm^{-1}) 3490 (NH), 3311 (NH).

M.P. 141.8 – 142.7 ºC.

NMR data:

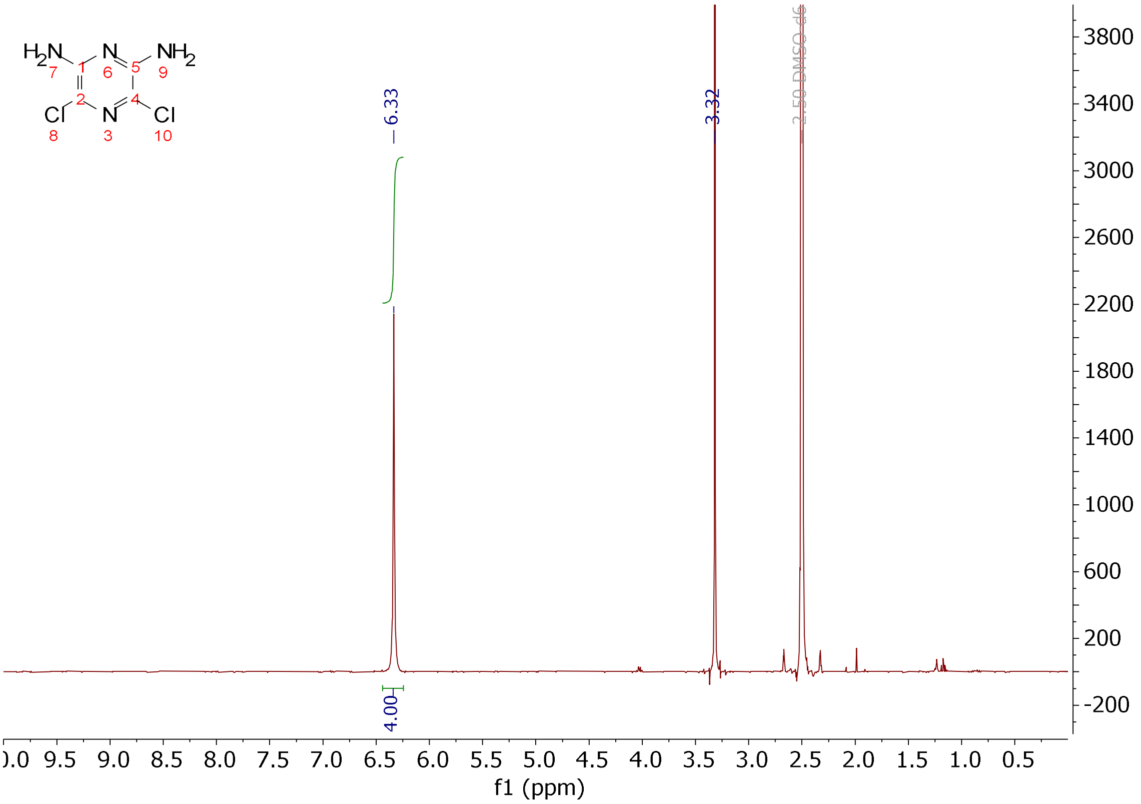

H2N NH2 Cl N Cl
6.33
3.32
2.50 DMSO-d6
4.00
f1 (ppm)

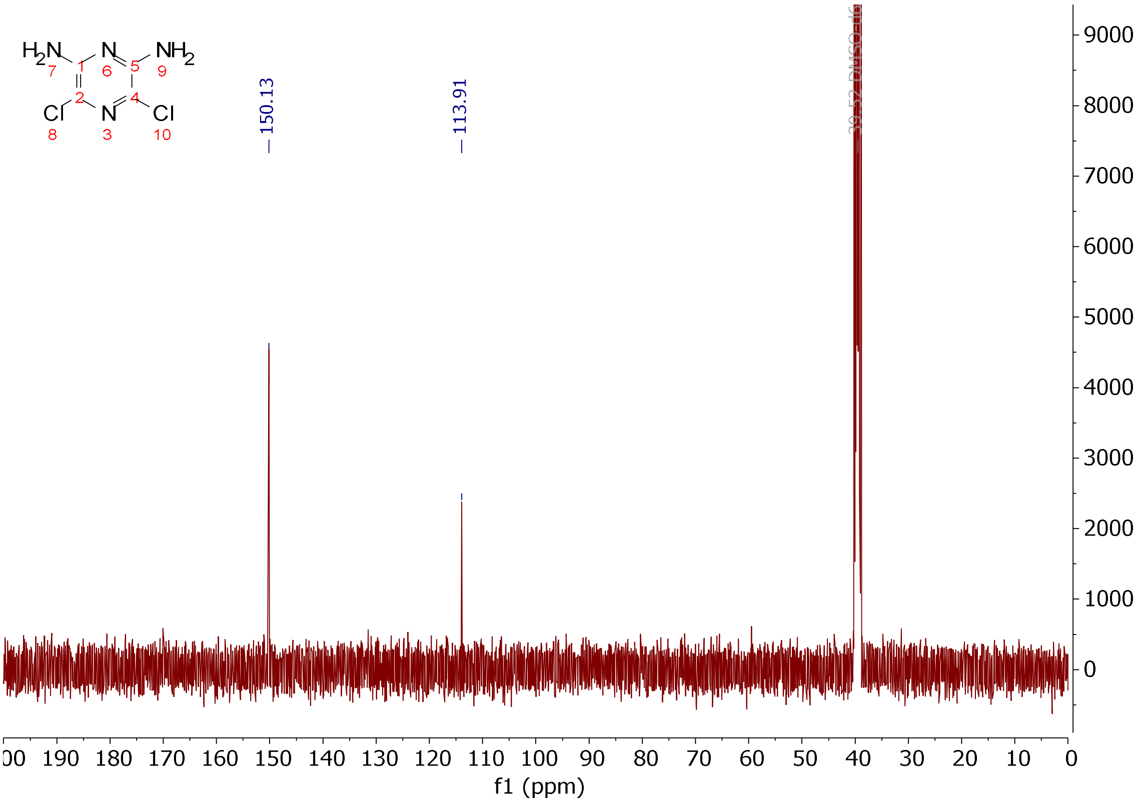

H2N NH2 Cl N Cl
150.13
113.91
39.52 DMSO-d6
f1 (ppm)

Strategy 6_1_1_1: 5,6-dichloroimidazo[4,5-b]pyrazine-2(1,3H)-thione

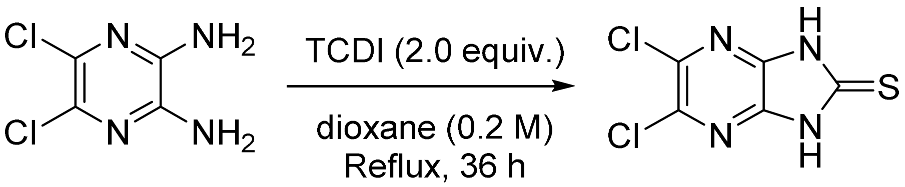

To a 100 mL RBF was added 2,3-diamino-5,6-dichloropyrazine (1.99 g, 11.11 mmol), a stirrer bar and dioxane (56 mL, 0.2 M). This was stirred to dissolve by heating to 80 °C. TCDI (1.98 g, 1.0 equiv.) was added, and the mixture heated to reflux for 24 h. An additional equivalent of TCDI (1.98 g) was added and heating was continued for 12 h. The mixture was allowed to cool to RT and the dioxane removed *in vacuo.* To the residue was added HCl (1 M, 20 mL) and EtOAc (20 mL). The mixture was transferred to a separatory funnel and separated. The aqueous layer was extracted twice with EtOAc (2x 20 mL). The organic layers were collected and dried ($MgSO_4$) and solvent removed *in vacuo.* The crude material was purified by column chromatography, eluting with 8 : 2 Pet. E : EtOAc to provide a yellow solid of 5,6-dichloroimidazo[4,5-*b*]pyrazine-2(1,3*H*)-thione (828 mg, 3.74 mmol, 34%).

^{1}H NMR (400 MHz, DMSO-d_6) δ 13.77 (br s, 2H).

^{13}C NMR (101 MHz, DMSO-d_6) δ 174.2, 140.5, 136.8.

IR (cm^{-1}) 3140 (NH), 1603.

M.P. 360 °C (deg.).

MS ES+ m/z calcd for $C_5H_2Cl_2N_4S$ $(M+H)^+$: 220.9455, found: 220.9455.

NMR data:

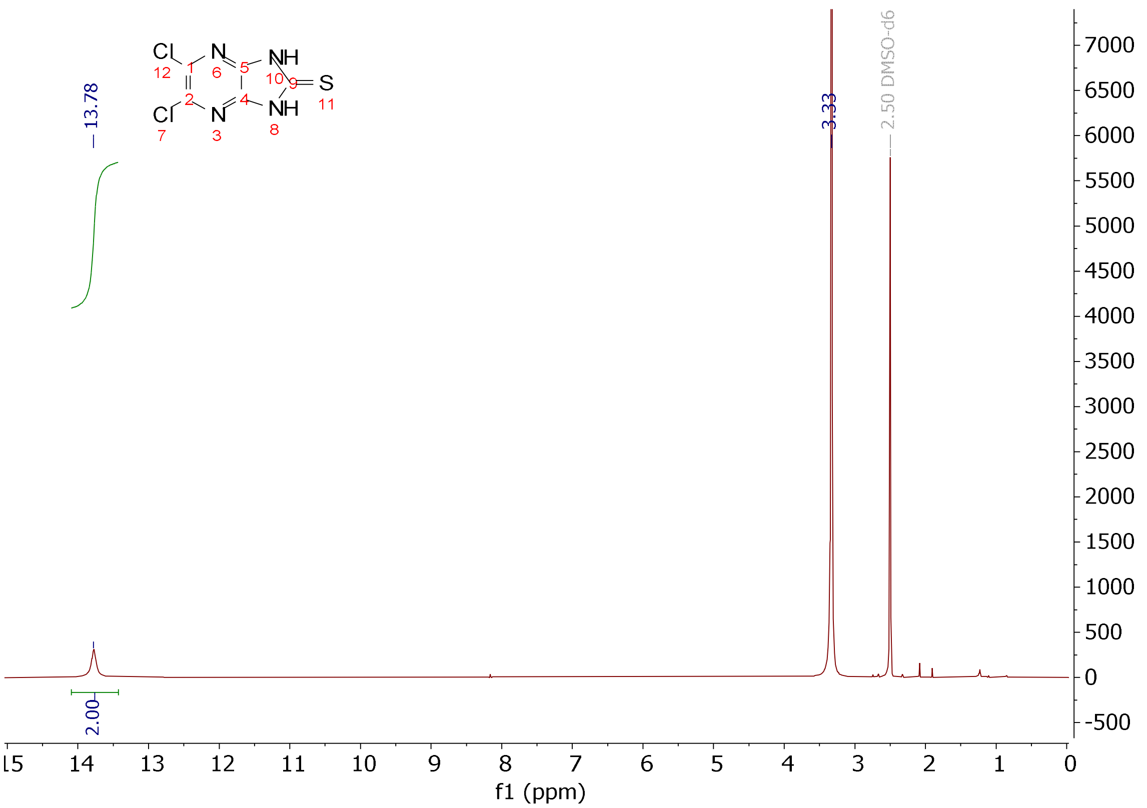

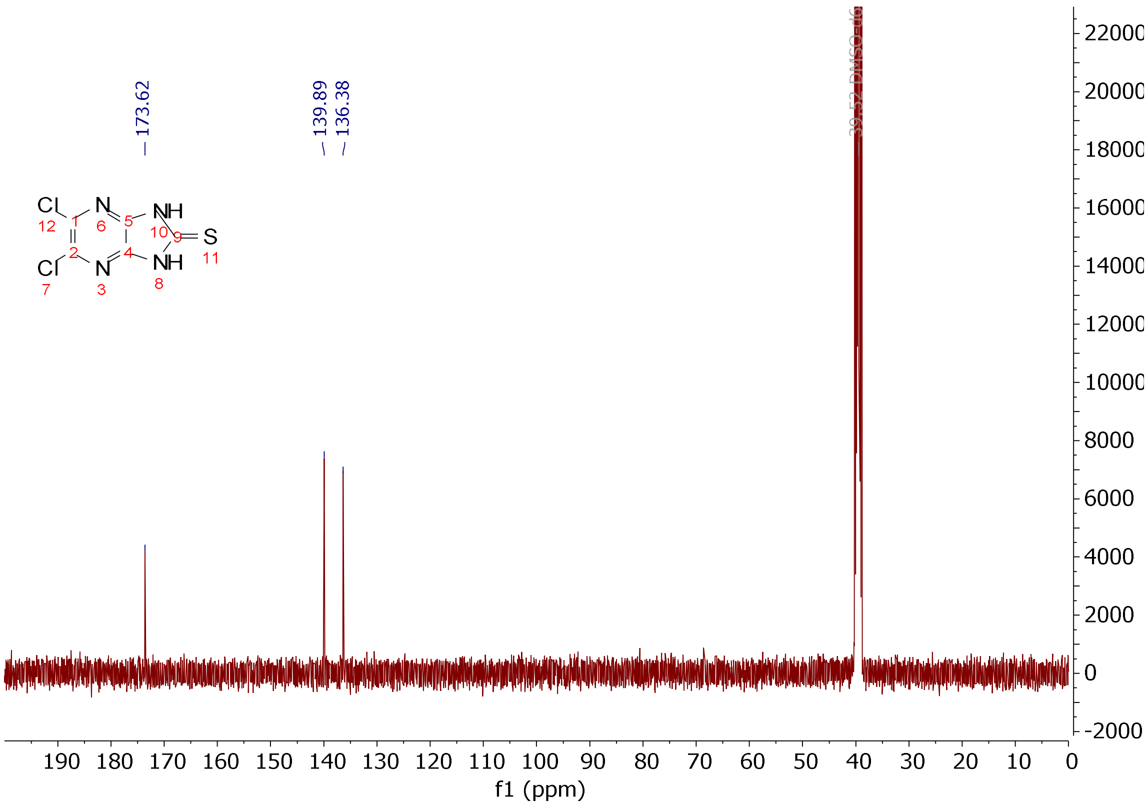

HRMS data:

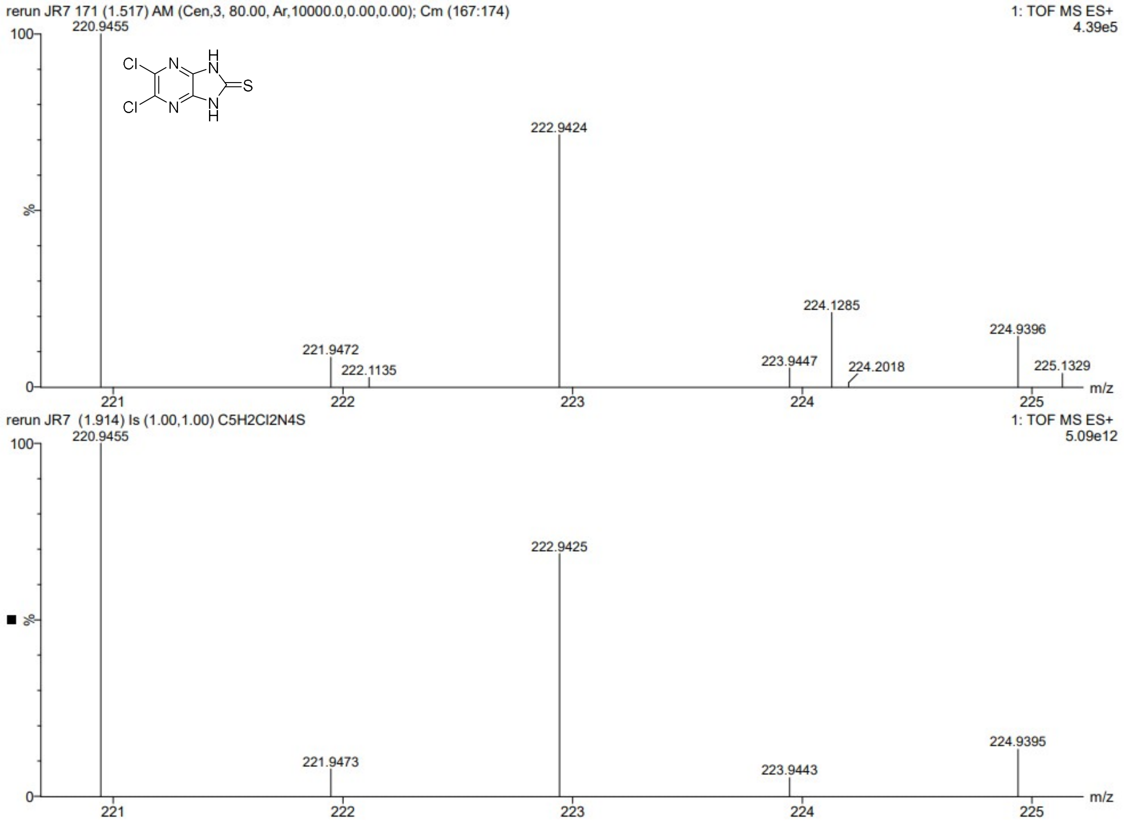

rerun JR7 171 (1.517) AM (Cen,3, 80.00, Ar,10000.0,0.00,0.00); Cm (167:174)
1: TOF MS ES+
4.39e5
100
%
0
220.9455
221.9472
222.1135
222.9424
223.9447
224.1285
224.2018
224.9396
225.1329
221
222
223
224
225
m/z
Cl
N
H
S
rerun JR7 (1.914) Is (1.00,1.00) C5H2Cl2N4S
1: TOF MS ES+
5.09e12
220.9455
221.9473
222.9425
223.9443
224.9395

Functionality Synthesis

N-(bromoacetyl)morpholine for ***compound 9***

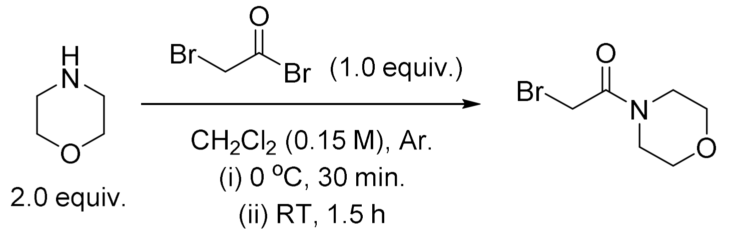

To a 25 mL RBF was added DCM (8.25 mL) and a stirrer bar. This was cooled to 0 °C and placed under argon. To this was added bromoacetyl bromide (0.107 mL, 1.0 equiv.) with stirring. To this solution was added morpholine (0.214 mL, 2.0 equiv.) and left to stir at 0 °C for 30 min. The mixture was allowed to warm to RT and stirred for 1.5 h. A sat. solution of NH_4Cl was added (10 mL), and the mixture extracted with diethyl ether (3x 10 mL). The organic layers were collected and dried (Na_2SO_4) and solvent removed *in vacuo.* to provide an off-white oil which was a mixture of bromoacetic acid and *N*-(bromoacetyl)morpholine. Re-dissolved in diethyl ether (approx. 10 mL) and washed with sat. soln. Na_2CO_3 (2x 10 mL). The ether was dried (Na_2SO_4), and solvent removed *in vacuo.* to provide a colourless oil of *N*-(bromoacetyl)morpholine (132 mg, 0.63 mmol, 52%).

^{1}H NMR (400 MHz, C*D*Cl$_3$) δ 3.87 (s, 2H), 3.78 – 3.72 (m, 2H), 3.72 – 3.68 (m, 2H), 3.67 – 3.61 (m, 2H), 3.57 – 3.48 (m, 2H).

^{13}C NMR (101 MHz, C*D*Cl$_3$) δ 165.5, 66.8, 66.5, 47.3, 42.6, 25.5.

IR (cm^{-1}): 3005, 2866 (C(sp^3)-H), 1650 (C=O).

Followed literature procedure, data in-line with literature values[16].

NMR data:

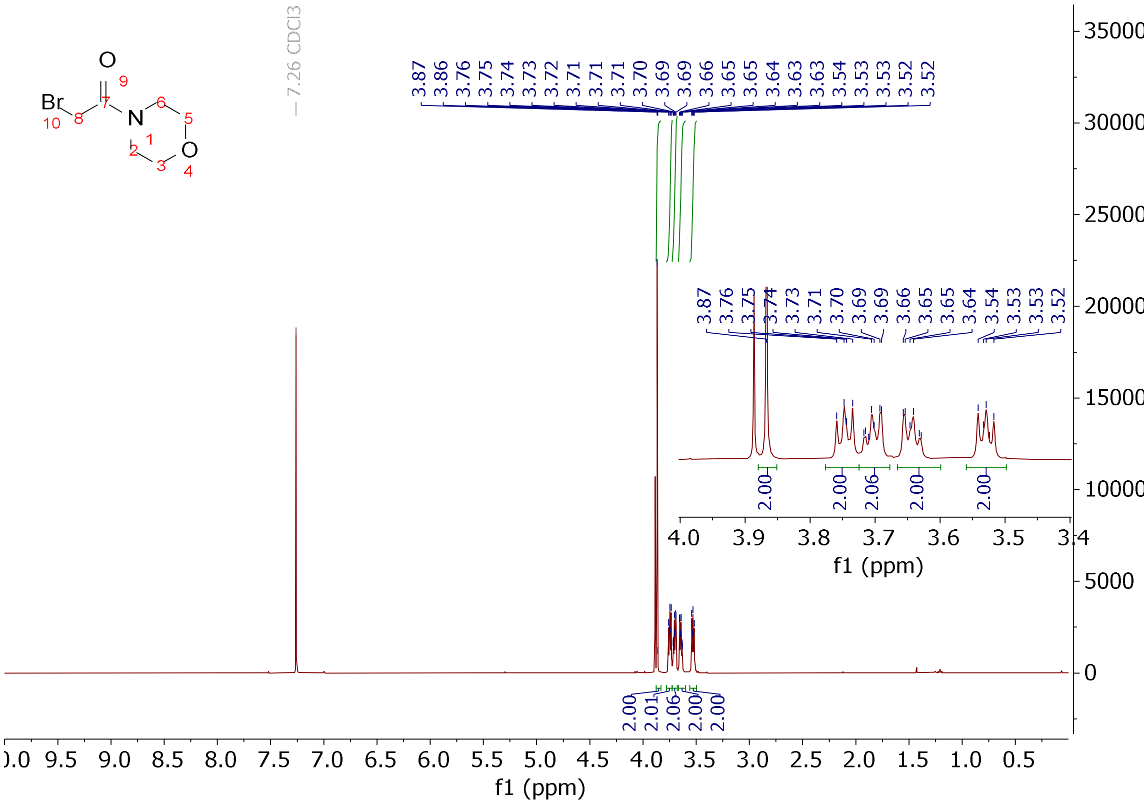

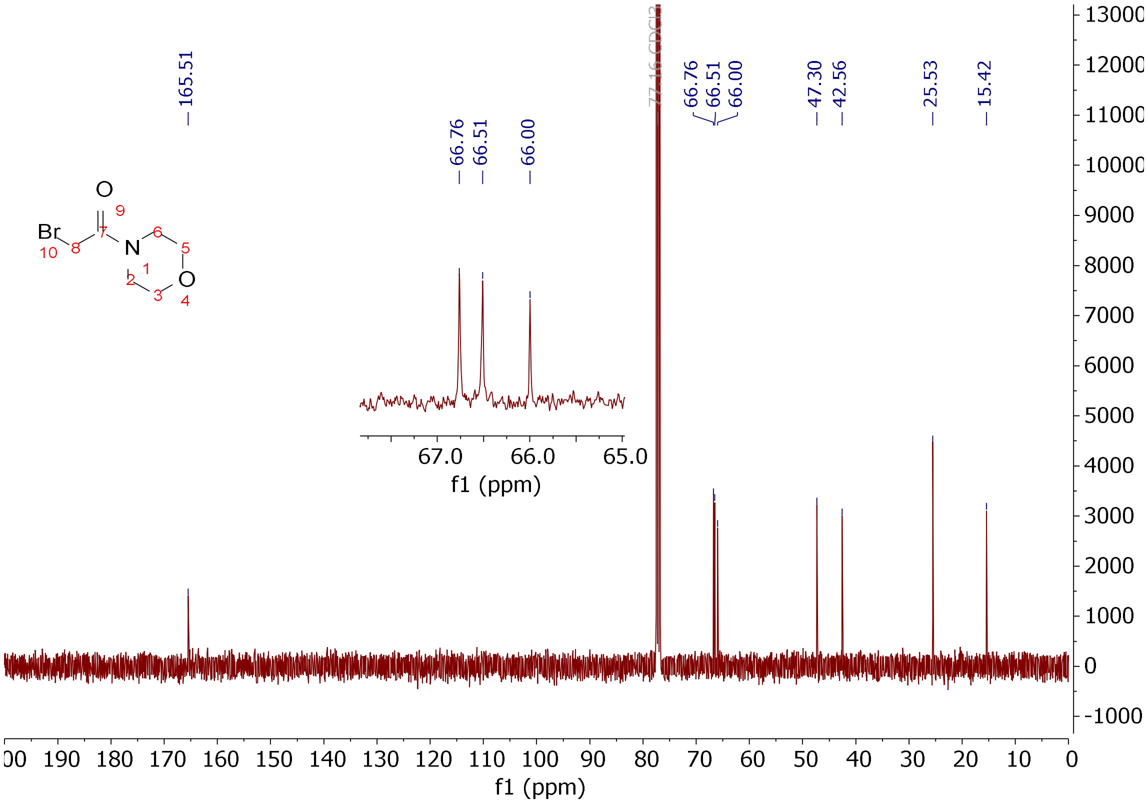

1-bromopentan-2-one for ***compound 10***

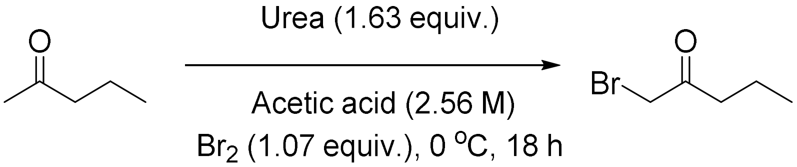

To an oven-dried 25 mL RBF was added urea (1.14 g, 1.63 equiv., 19 mmol), acetic acid (5 mL, 2.56 M) and a stirrer bar. This was cooled to 0 °C and stirred. 2-Pentanone (1.23 mL, 11.6 mmol) was added and then bromine (0.63 mL, 1.07 equiv.) was added at 0 °C with vigorous stirring. The reaction was stirred at RT for 18 h. Water (20 mL) was added, and the mixture transferred to a separatory funnel, extracted with DCM (3x 20 mL). The organic layers were collected and washed with sat. soln. Na_2CO_3 until no more gas evolution was observed. The organic layers were dried ($MgSO_4$), and solvent removed *in vacuo.* The residue was purified *via* column chromatography (3% Et_2O in Hex.) to yield 1-bromopentan-2-one as a clear oil (542 mg, 3.28 mmol, 28%).

^{1}H NMR (400 MHz, C*D*Cl$_3$) δ 3.88 (s, 2H), 2.63 (t, J = 7.3 Hz, 2H), 1.65 (p, J = 7.3 Hz, 2H), 0.94 (t, J = 7.3 Hz, 3H).

^{13}C NMR (101 MHz, C*D*Cl$_3$) δ 202.2, 41.8, 34.4, 17.5, 13.7.

Followed literature procedure, data in-line with literature values[17].

NMR data:

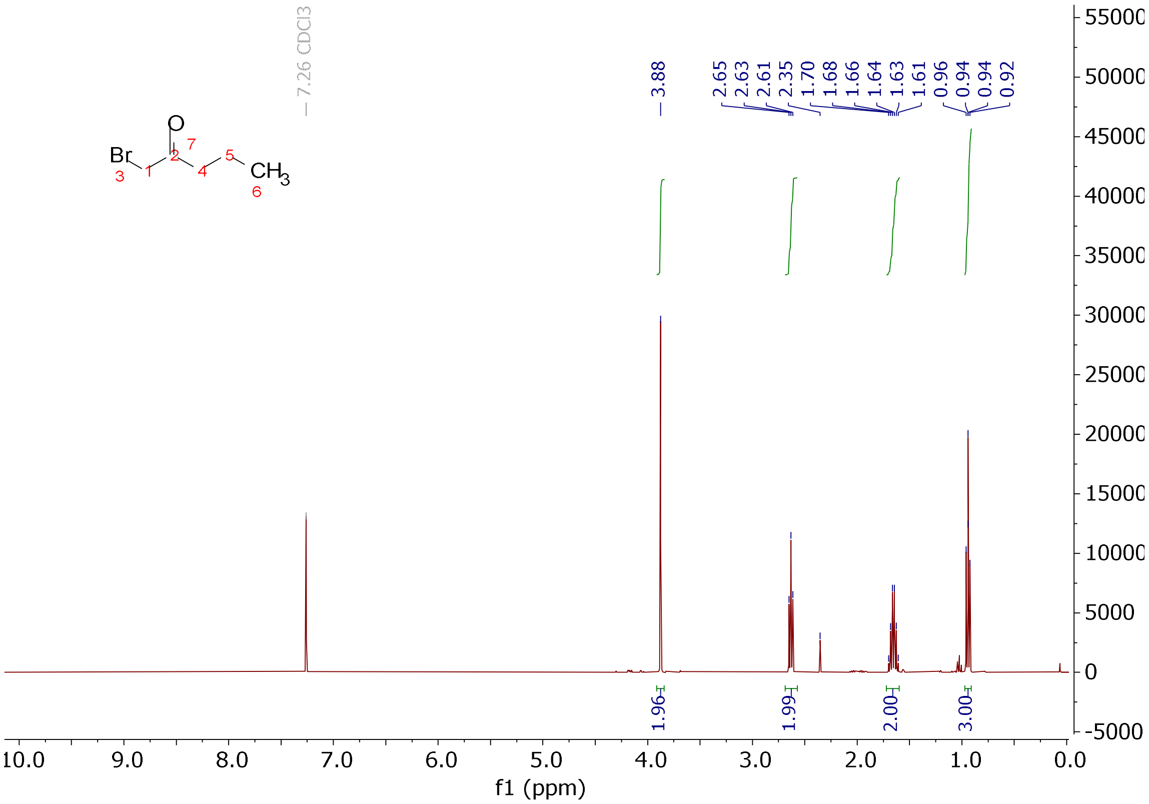

7.26 CDCl3
3.88
2.65
2.63
2.61
2.35
1.70
1.68
1.66
1.64
1.63
1.61
0.96
0.94
0.94
0.92
Br
O
CH3
1.96
1.99
2.00
3.00
f1 (ppm)

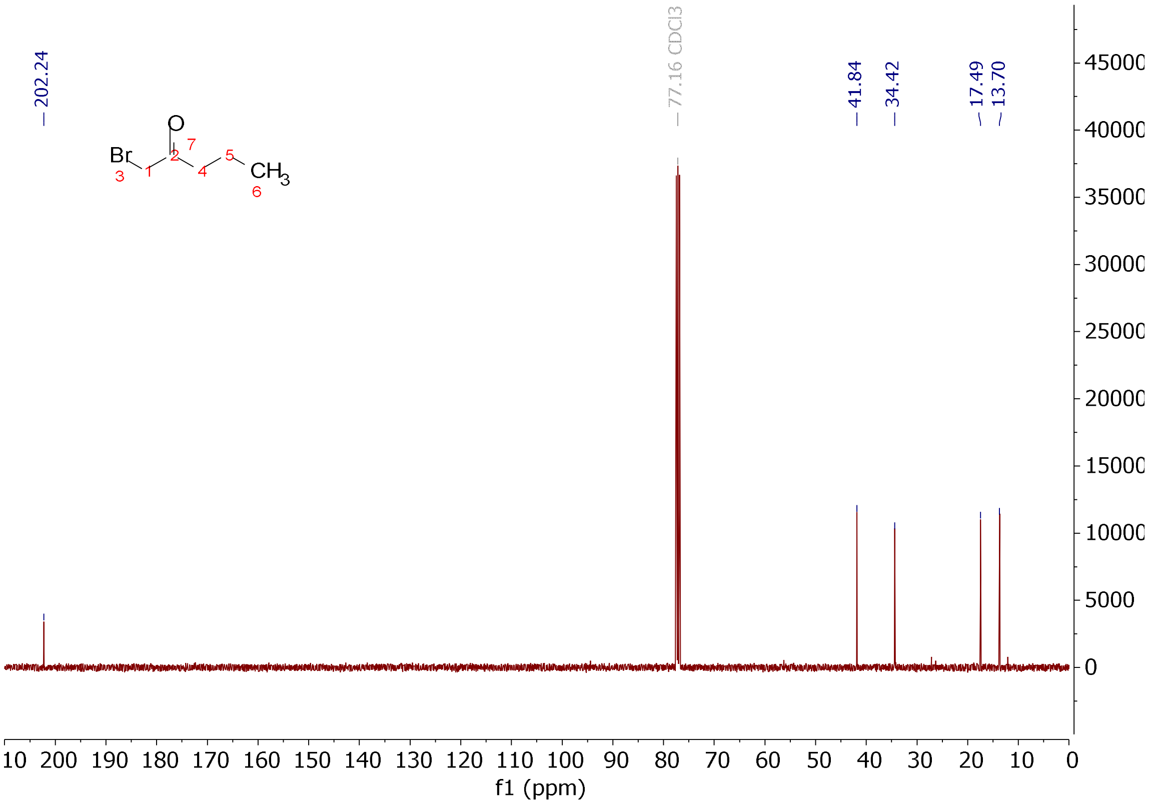

202.24
77.16 CDCl3
41.84
34.42
17.49
13.70
Br
O
CH3
f1 (ppm)

N-(bromoacetyl)glycine ethyl ester for ***compound 12***

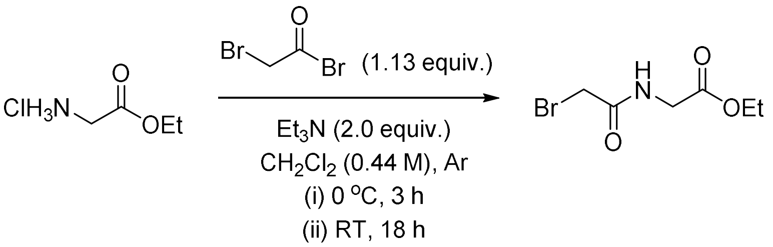

To an oven-dried 8 mL vial was added glycine ethyl ester hydrochloride (250 mg, 1.79 mmol) and placed under argon atmosphere. DCM (4.07 mL, 0.44 M) was then added. Triethylamine (0.5 mL, 2.0 equiv.) was added to the suspension with stirring and the vial cooled to 0 °C. Bromoacetyl bromide (0.179 mL, 1.13 equiv.) was added dropwise *via* syringe and the mixture allowed to stir at 0 °C for 3 h, then warmed to RT and left to stir for 18 h. The reaction was quenched with water (4 mL), transferred to a sep. funnel and more DCM (10 mL) added. The aqueous layer was removed, and the DCM extracted with 1 M HCl (10 mL), water (10 mL), sat. soln. $NaHCO_3$ (10 mL) and brine (10 mL). The organic layer was dried ($MgSO_4$), and the solvent removed *in vacuo.* to provide a brown solid. Partially purified by column chromatography using $CHCl_3$ -> EtOAc : $CHCl_3$ (1:1) to provide a light-orange solid of ethyl α-bromoacetamidoacetate (166 mg, 0.74 mmol, 41%). Can be used in the next step without further purification. Alternatively, the crude was recrystallised from EtOAc to provide an off-white crystalline solid after washing with a small amount of EtOAc.

^{1}H NMR (400 MHz, C*D*Cl$_3$) δ 7.00 (br s, 1H), 4.25 (q, J = 7.2 Hz, 2H), 4.14 – 4.06 (m, 2H), 3.92 (s, 2H), 1.30 (t, J = 7.2 Hz, 3H).

^{13}C NMR (101 MHz, C*D*Cl$_3$) δ 169.3, 165.8, 62.0, 42.1, 28.7, 14.3.

IR (cm^{-1}) 3264 (NH), 2987, 2944 (C(sp^3)-H), 1736 (C=O ester), 1643 (C=O amide).

M.P. 65 – 68 °C.

Followed literature procedure, data in-line with literature values[18].

NMR data:

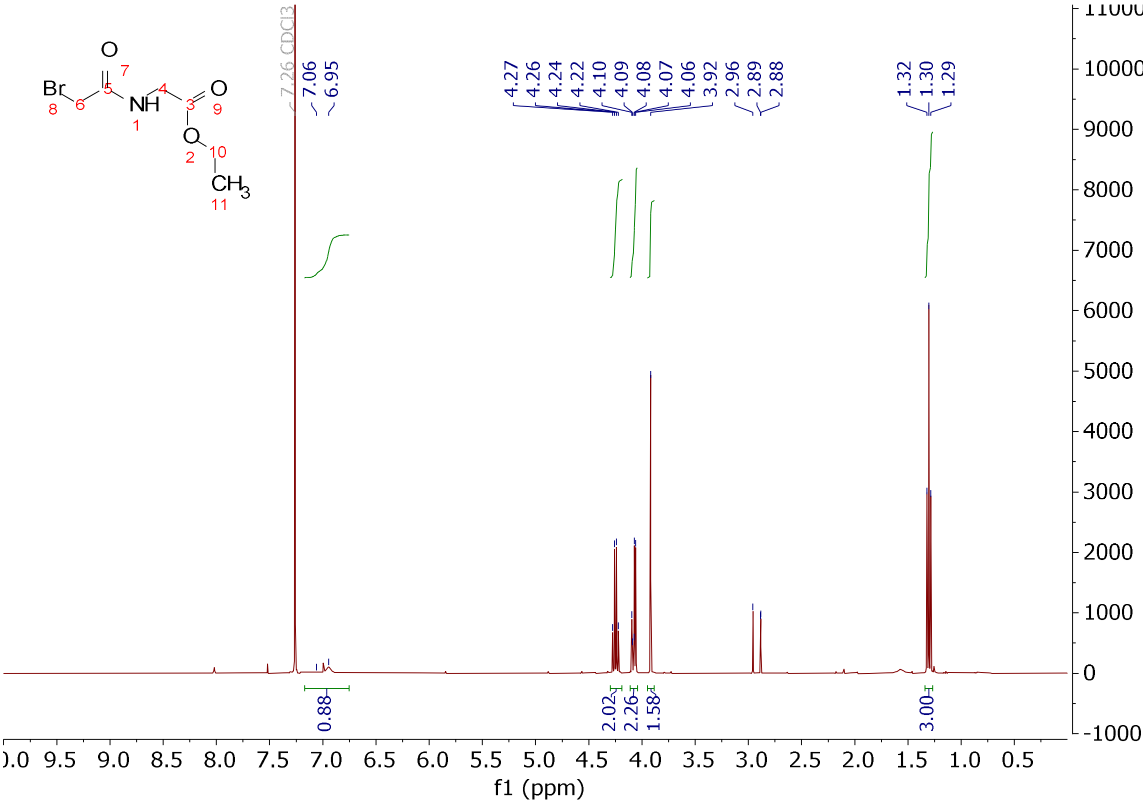

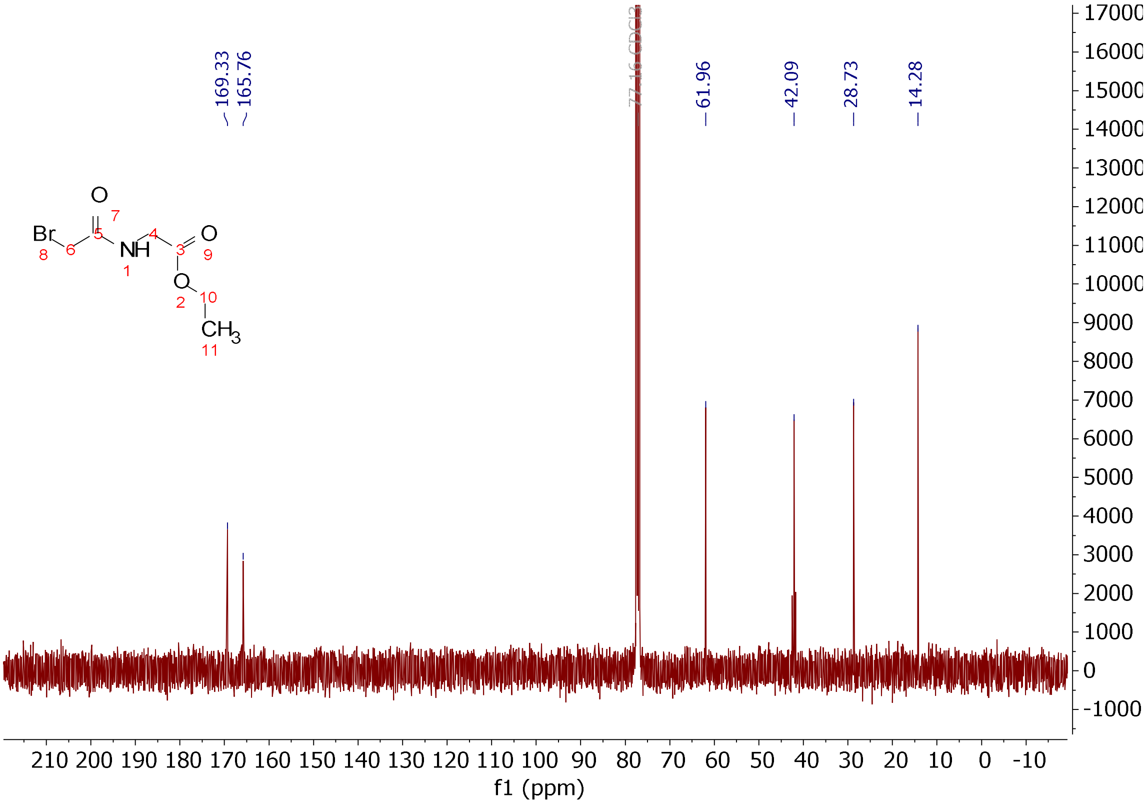

(+)-(2S)-methyl 2-(2-bromoacetylamino)-3-(tert-butoxy)propanoate for ***compound 14***

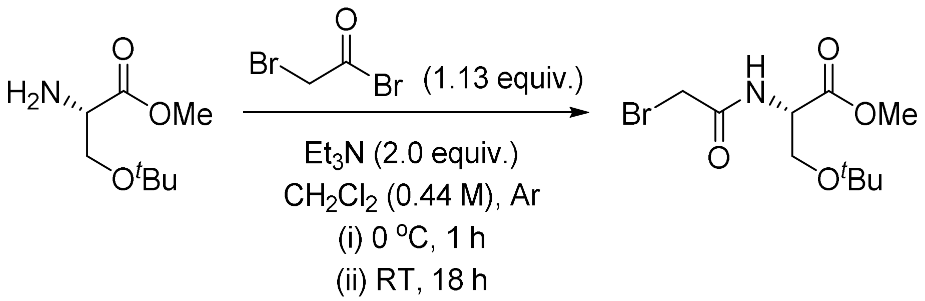

To an 8 mL vial under argon was added (*S*)-H-Serine(O^{t}Bu) methyl ester (0.25 g, 1.18 mmol), DCM (2.7 mL, 0.44 M) and triethylamine (0.33 mL, 2.0 equiv.). This was cooled to 0 °C and bromoacetyl bromide (0.116 mL, 1.13 equiv.) added dropwise *via* syringe, left to warm to RT over 1 h and left to stir at RT for 18 h. The reaction was quenched with water (4 mL), transferred to a sep. funnel and DCM (10 mL) added. The aqueous layer was removed, and the DCM extracted with 1 M HCl (10 mL), water (10 mL), sat. soln. $NaHCO_3$ (10 mL) and brine (10 mL). The organic layer was dried ($MgSO_4$), and the solvent removed *in vacuo.* to provide a brown solid. Purified by column chromatography (7:3 Pet.E : EtOAc) and collected (+)-(2*S*)-methyl 2-(2-bromoacetylamino)-3-(*tert*-butoxy)propanoate as a yellow oil (161 mg, 0.54 mmol, 46%).

^{1}H NMR (400 MHz, C*D*Cl$_3$) *δ* 7.20 (br s, 1H), 4.66 (dt, *J* = 8.3, 3.0 Hz, 1H), 3.94 (d, *J* = 13.6 Hz, 1H), 3.89 (d, *J* = 13.6 Hz, 1H), 3.84 (dd, *J* = 9.1, 3.0 Hz, 1H), 3.76 (s, 3H), 3.59 (dd, *J* = 9.1, 3.0 Hz, 1H), 1.15 (s, 9H).

^{13}C NMR (101 MHz, C*D*Cl$_3$) *δ* 170.5, 165.5, 73.8, 61.7, 53.5, 52.7, 28.9, 27.4.

IR (cm^{-1}) 3313 (NH), 1746 (C=O ester), 1658 (C=O amide).

Adapted from literature procedure[18].

NMR data:

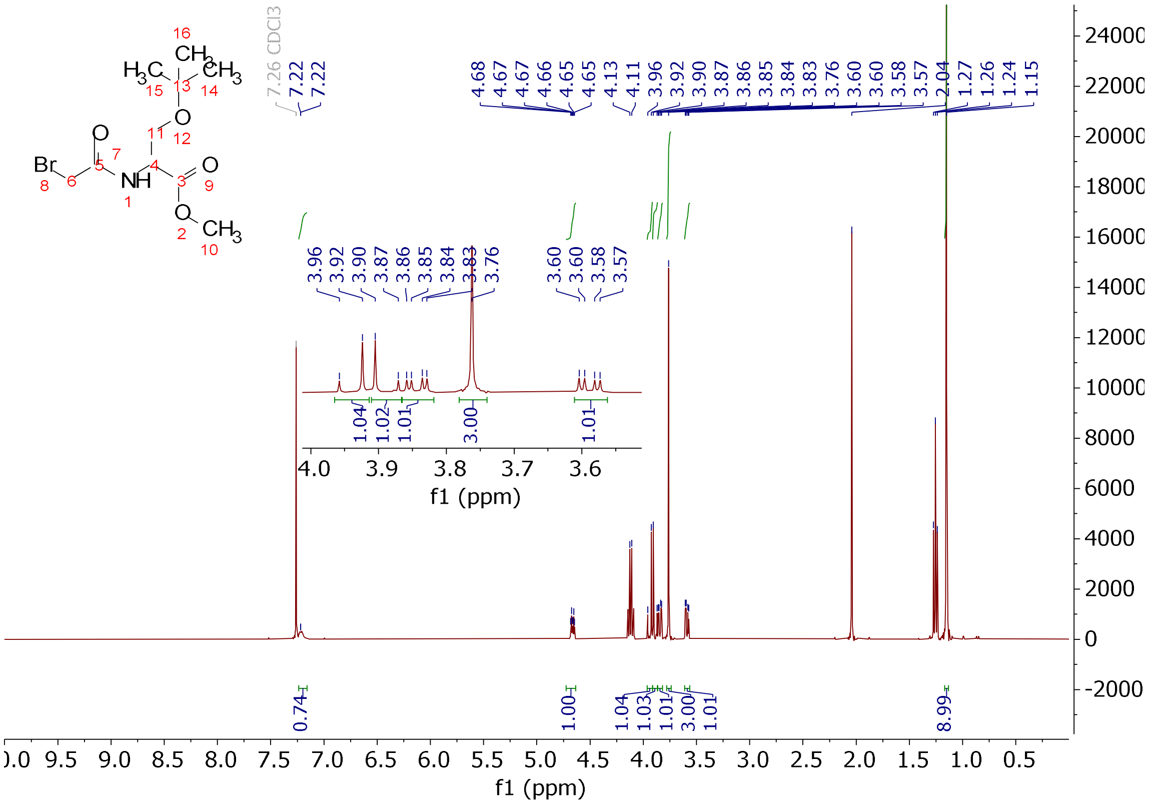

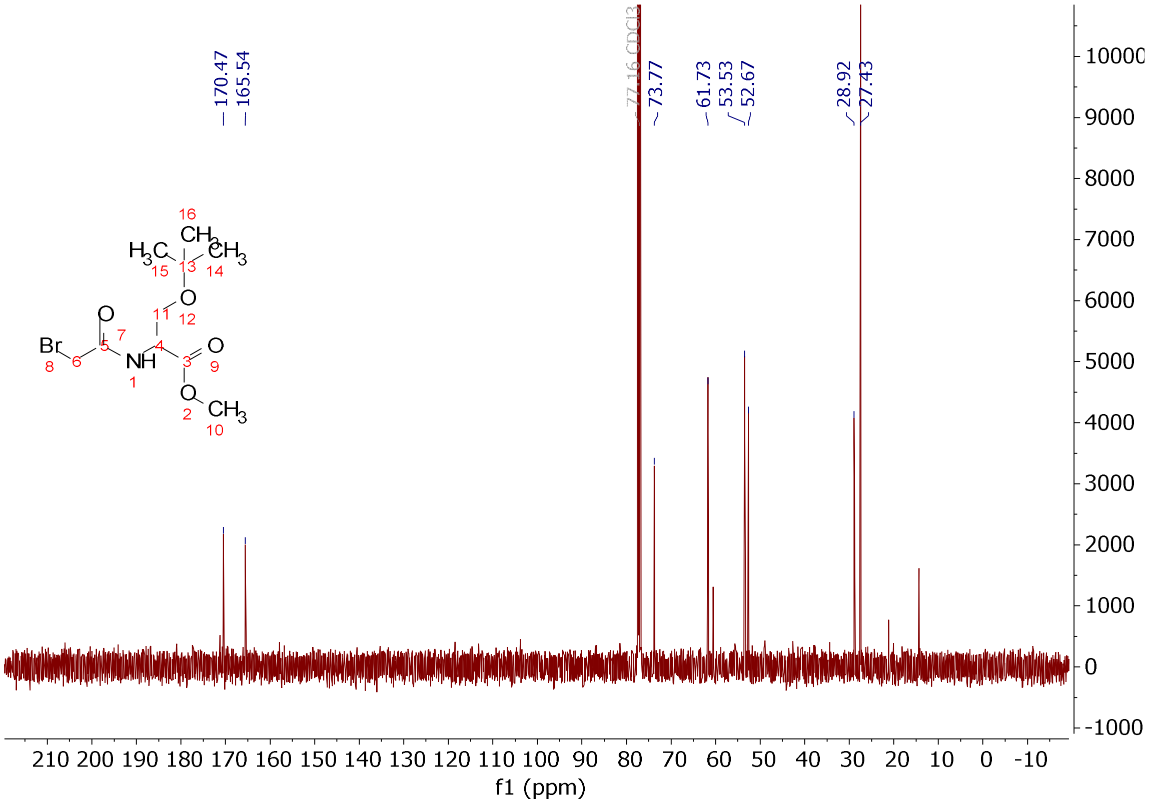

Methyl (3-hydroxy)-2(S)-({5,6-dichloro-1H-imidazo[4,5-b]pyrazine-2-yl}sulfanyl)propanioate for ***compound 14***

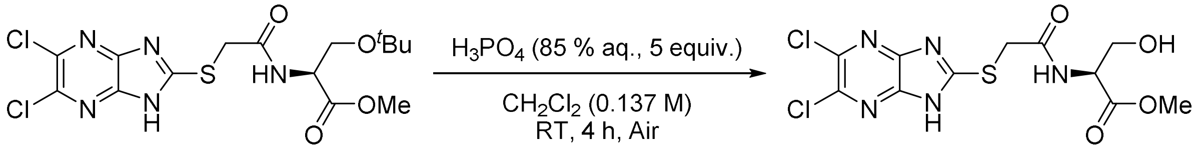

To an 8 mL vial was added a stirrer bar and methyl (3-*tert*-butoxy)-2(*S*)-[(5,6-dichloro-1*H*-imidazo[4,5-*b*]pyrazine-2-sulfanyl)acetamido]propanioate (60 mg, 0.137 mmol). DCM (1 mL, 0.137 M) was added *via* syringe. Once the material had dissolved, phosphoric acid (79 µL, 85% aq. 5 equiv.) was added dropwise with stirring at RT. Left to stir at RT over 4 h, the reaction was diluted with water (5 mL) and extracted with EtOAc (3x 10 mL). The aqueous phase was neutralised ($NaHCO_3$) and re-extracted with acetonitrile (3x 10 mL) the organic layers were collected and dried ($MgSO_4$) and solvent removed *in vacuo.* to provide methyl (3-hydroxy)-2(*S*)-({5,6-dichloro-1*H*-imidazo[4,5-*b*]pyrazine-2-yl}sulfanyl)propanioate as an off-white solid (29.7 mg, 0.078 mmol, 57%).

^{1}H NMR (400 MHz, 1:6 v/v DMSO-d_6 : CD_3CN) δ 8.17 (s, 1H), 4.43 (dt, J = 8.0, 4.1 Hz, 1H), 4.15 (d, J = 2.9 Hz, 2H), 3.80 (dd, J = 11.3, 4.3 Hz, 1H), 3.68 (dd, J = 11.3, 3.9 Hz, 1H), 3.64 (s, 3H).

^{13}C NMR (101 MHz, 1:6 v/v DMSO-d_6 : CD_3CN) δ 171.6, 168.2, 162.2, 138.7, 62.5, 56.2, 52.7, 35.3 (8 out of 9 carbon resonances observed).

Followed literature procedure[19].

NMR data:

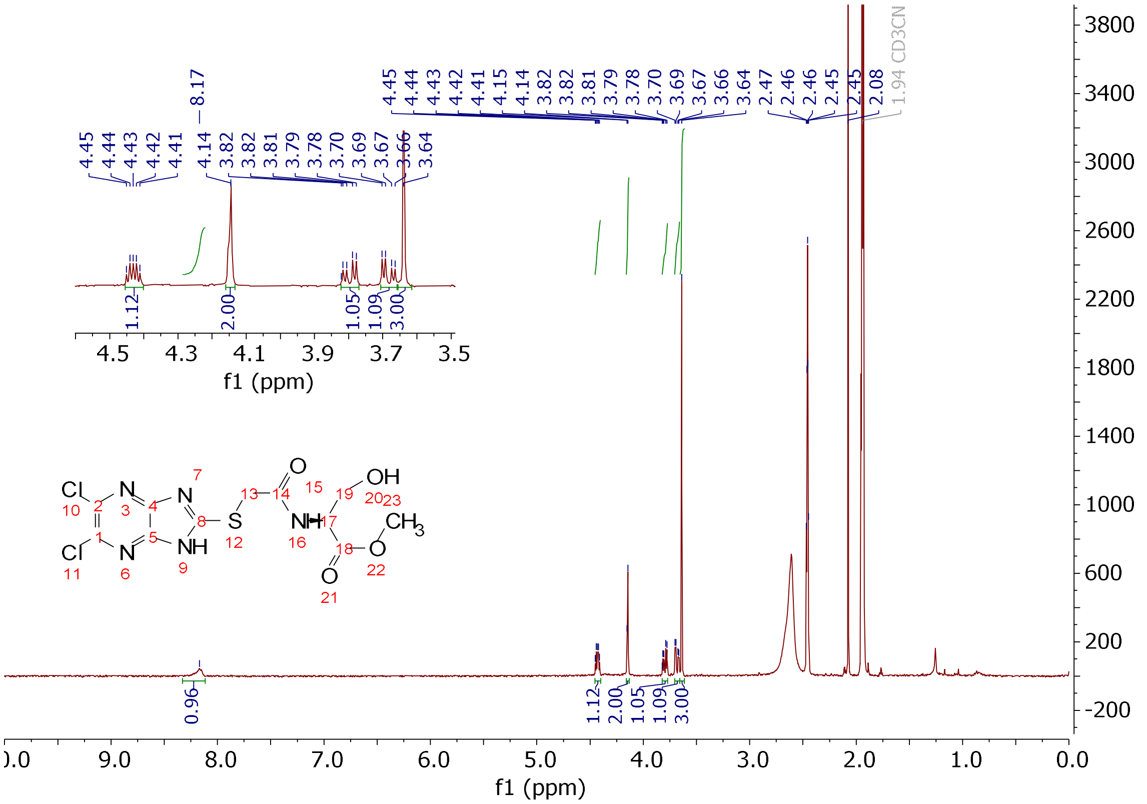

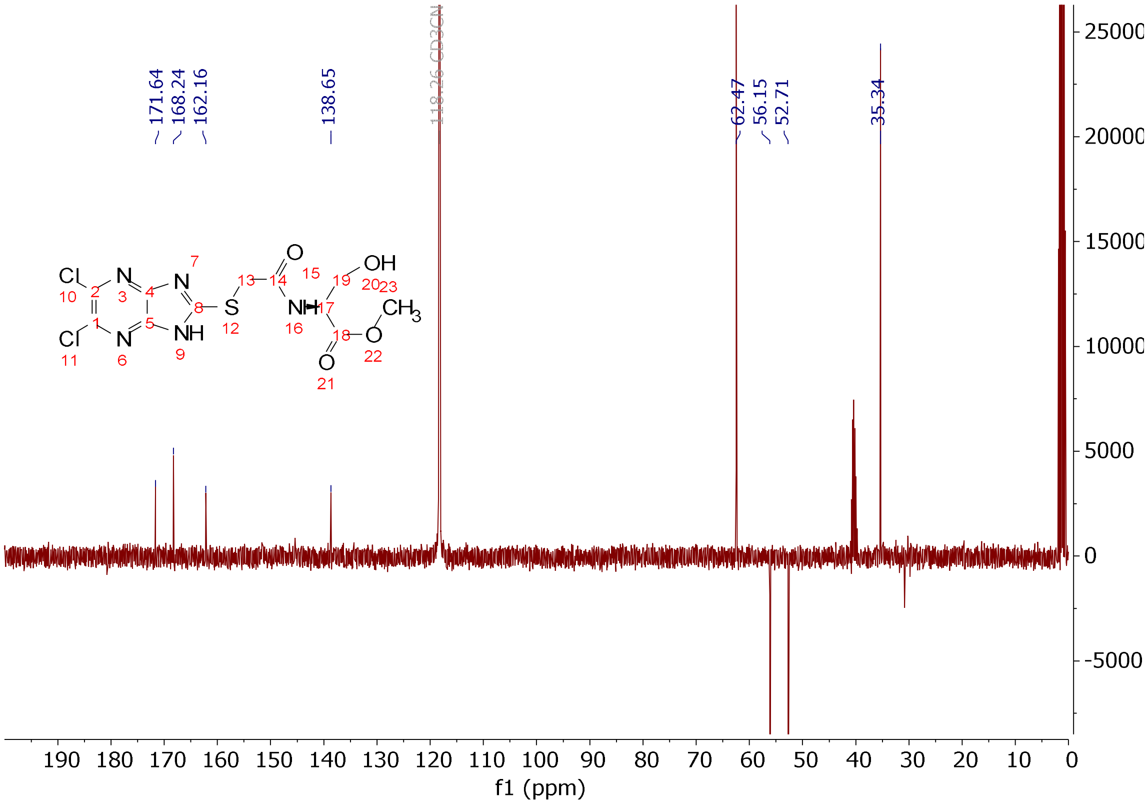

Compound Synthesis

***Compound 1** – Ethyl imidazo[1,2-a]pyrazine-2-acetate*

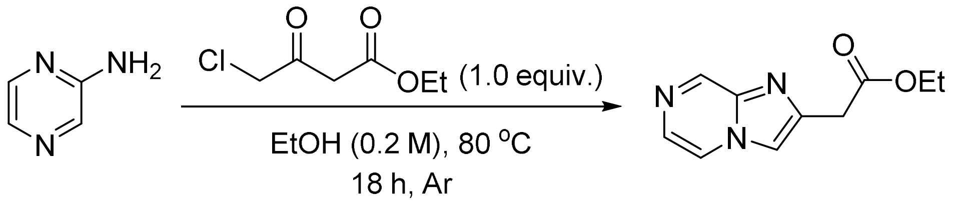

To a 100 mL RBF was added 2-aminopyrazine (500 mg, 5.25 mmol) and EtOH (26.5 mL, over mol. sieves, 0.2 M). to the stirred solution, ethyl 4-chloroacetoacetate (0.70 mL, 1.0 equiv.) was then added dropwise *via* syringe and the mixture heated to reflux for 18 h. The EtOH was removed under reduced pressure and to the residue was added $NaHCO_3$ sat. soln. (20 mL) and this was extracted with $CHCl_3$ (4x 20 mL). The organic layers were collected and dried ($MgSO_4$). Solvent removed under reduced pressure and the residue was subjected to sequential column chromatography (1% EtOH in $CHCl_3$ -> 3% EtOH in $CHCl_3$, then 7:2.5:0.5 EtOAc : Hex : Tol.) and the product (0.1349 g) was recrystallised from hexane to provide ethyl imidazo[1,2-*a*]pyrazine-2-acetate as white crystals (49.4 mg, 0.24 mmol, 5%).

^{1}H NMR (400 MHz, C*D*Cl_3) δ 9.03 (d, J = 1.5 Hz, 1H), 8.02 (dd, J = 4.6, 1.5 Hz, 1H), 7.86 (d, J = 4.6 Hz, 1H), 7.73 (d, J = 0.7 Hz, 1H), 4.21 (q, J = 7.1 Hz, 2H), 3.92 (d, J = 0.7 Hz, 2H), 1.29 (t, J = 7.1 Hz, 3H).

^{13}C NMR (101 MHz, C*D*Cl_3) δ 170.5, 143.5, 142.3, 140.3, 129.7, 118.8, 112.3, 61.4, 35.3, 14.3.

IR (cm^{-1}): 1714 (C=O).

Adapted from literature procedure, data in-line with literature data[20].

NMR data:

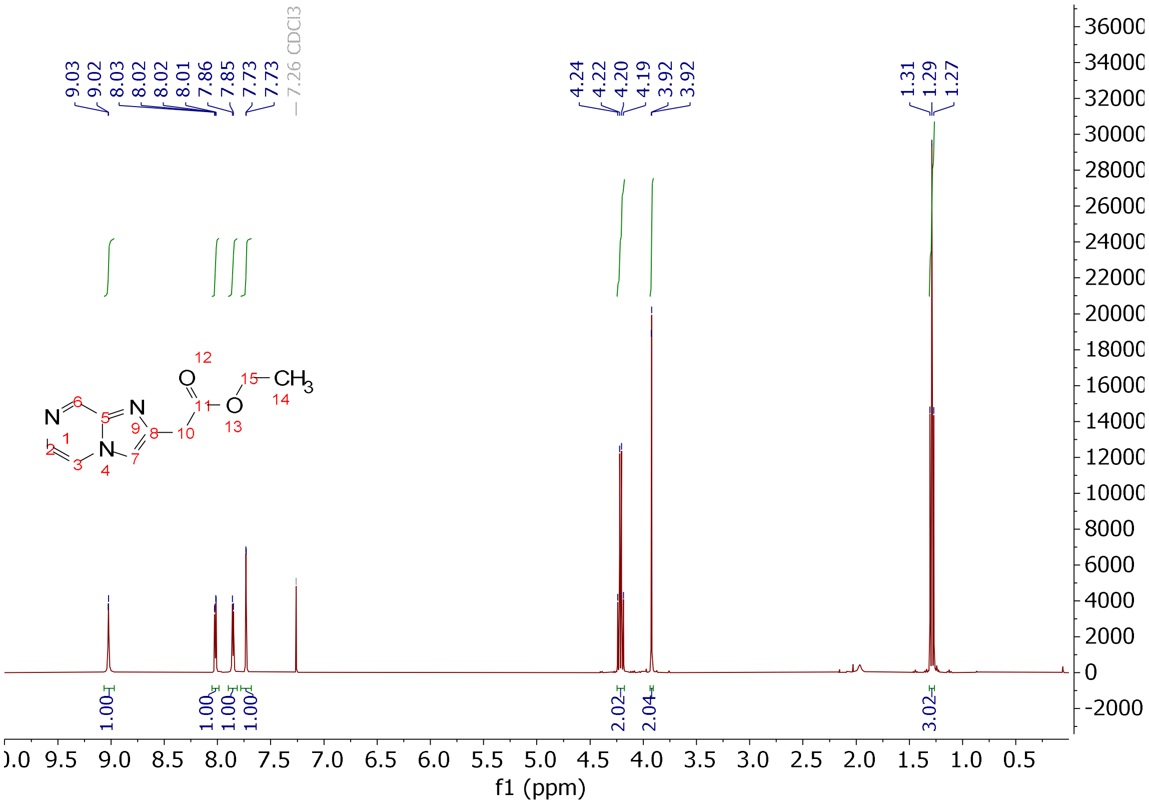
9.03
9.02
8.03
8.02
8.02
8.01
7.86
7.85
7.73
7.73
7.26 CDCl3
4.24
4.22
4.20
4.19
3.92
3.92
1.31
1.29
1.27
1.00
1.00
1.00
1.00
2.02
2.04
3.02
f1 (ppm)

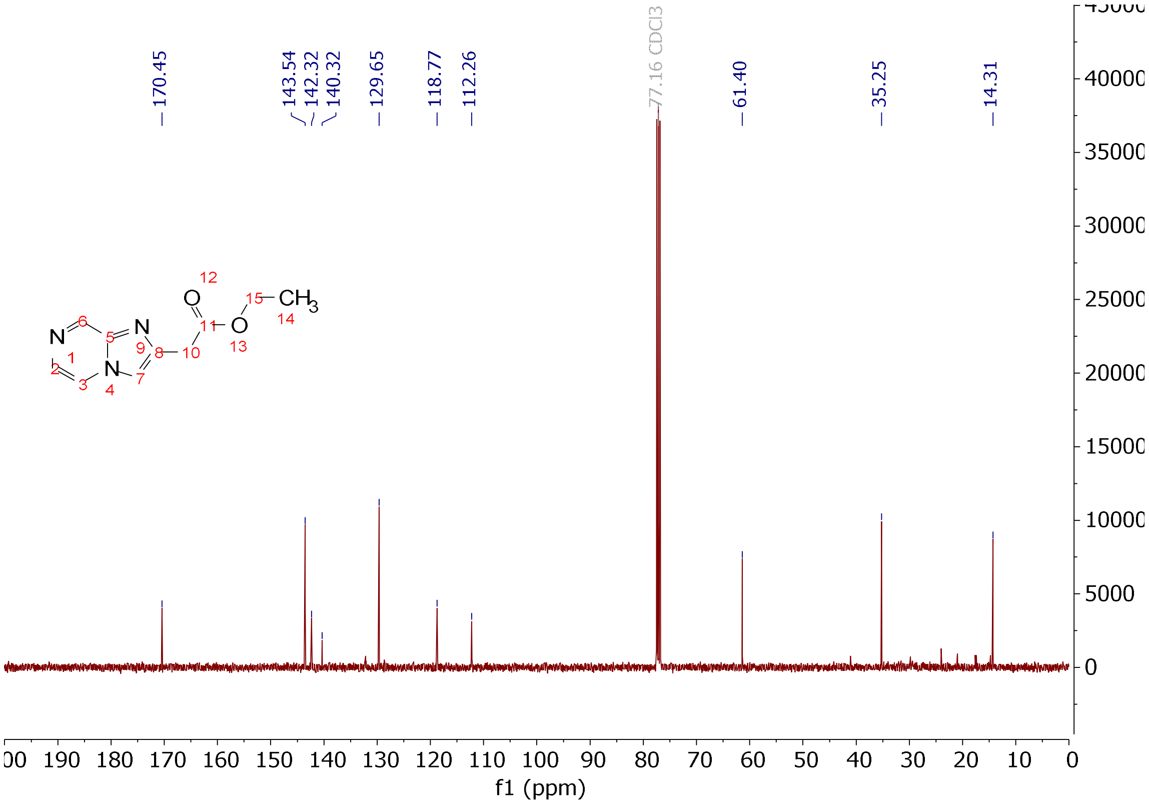
170.45
143.54
142.32
140.32
129.65
118.77
112.26
77.16 CDCl3
61.40
35.25
14.31
f1 (ppm)

Compound 2 *– Ethyl 6-chloroimidazo[1,2-a]pyrazine-2-acetate*

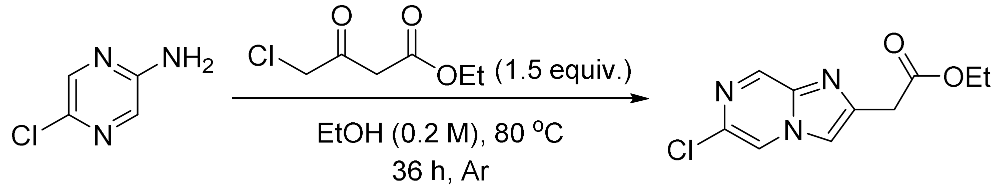

To an oven-dried 3-necked 100 mL RBF was attached a glass stopper, a reflux condenser and a suba seal. To this was added a stirrer bar, 2-amino-5-chloropyrazine (0.305 g, 2.35 mmol) and the vessel was placed under an atmosphere of argon. EtOH (over mol. sieves, 0.2 M, 12.0 mL) was added *via* syringe. To this stirred solution was added ethyl 4-chloroacetoacetate (0.47 mL, 1.5 equiv.) and the mixture heated to reflux for 36 h. The mixture was allowed to cool to RT and the EtOH removed *in vacuo.* The residue was taken up in $NaHCO_3$ sat. soln. (25 mL) and extracted with chloroform (3x 20 mL). The organic layers were collected, washed with water (20 mL), dried ($MgSO_4$) and solvent removed *in vacuo.* The residue was then loaded onto silica and purified twice *via* column chromatography (1% MeOH in DCM) and (3.5:6.0:0.5 EtOAc : Hex. : Tol.), and then triturated with cold Et_2O to afford ethyl 6-chloroimidazo[1,2-*a*]pyrazine-2-aceate as a beige crystalline solid (42 mg, 0.17 mmol, 7%).

^{1}H NMR (400 MHz, C*D*Cl_3) δ 8.85 (dd, J = 1.4, 0.7 Hz, 1H), 8.13 (d, J = 1.4 Hz, 1H), 7.77 (d, J = 0.7 Hz, 1H), 4.23 (q, J = 7.1 Hz, 2H), 3.93 (d, J = 0.7 Hz, 2H), 1.30 (t, J = 7.1 Hz, 3H).

^{13}C NMR (101 MHz, C*D*Cl_3) δ 170.0, 143.8, 141.7, 139.0, 135.2, 116.5, 113.0, 61.6, 35.1, 14.3.

IR (cm^{-1}): 1715 (C=O)

M.P. 100.2 – 100.4 °C.

MS ES+ Calcd for $C_{10}H_{10}ClN_3O_2$ $(M+H)^+$: 240.0540, found: 240.0534.

Adapted from literature procedure, data in-line with literature data[20].

NMR data:

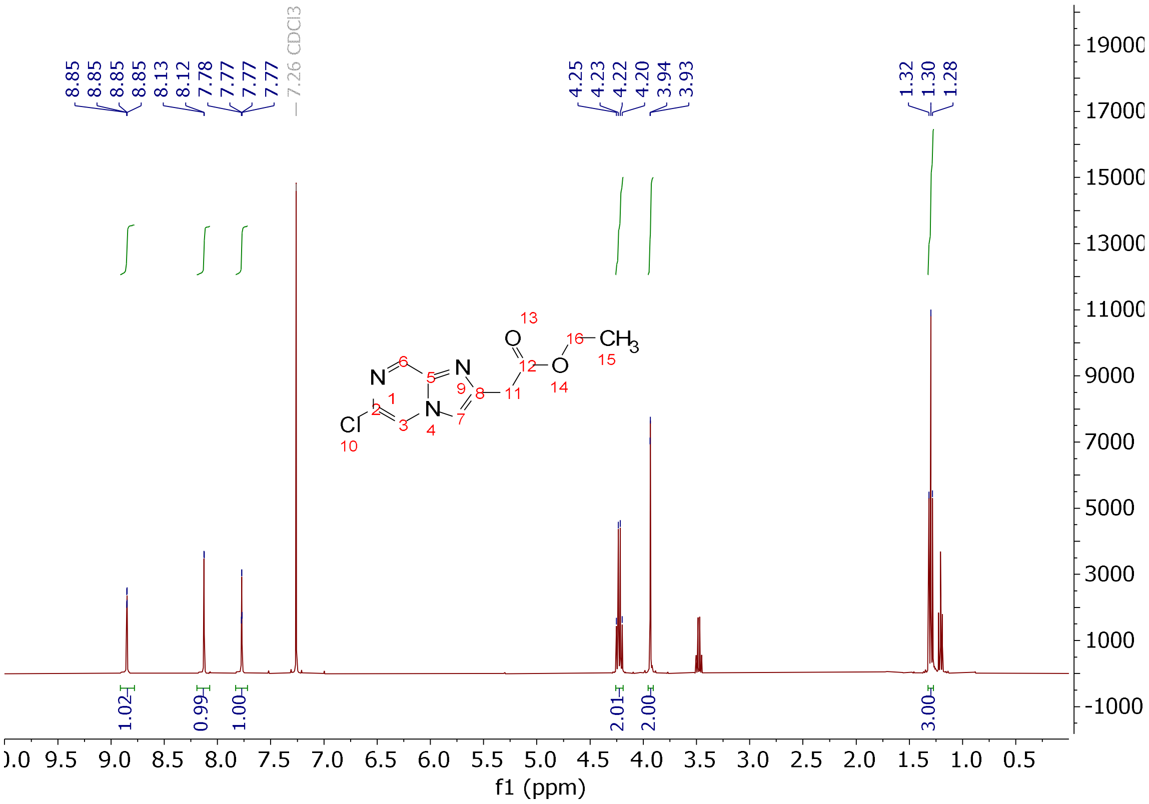

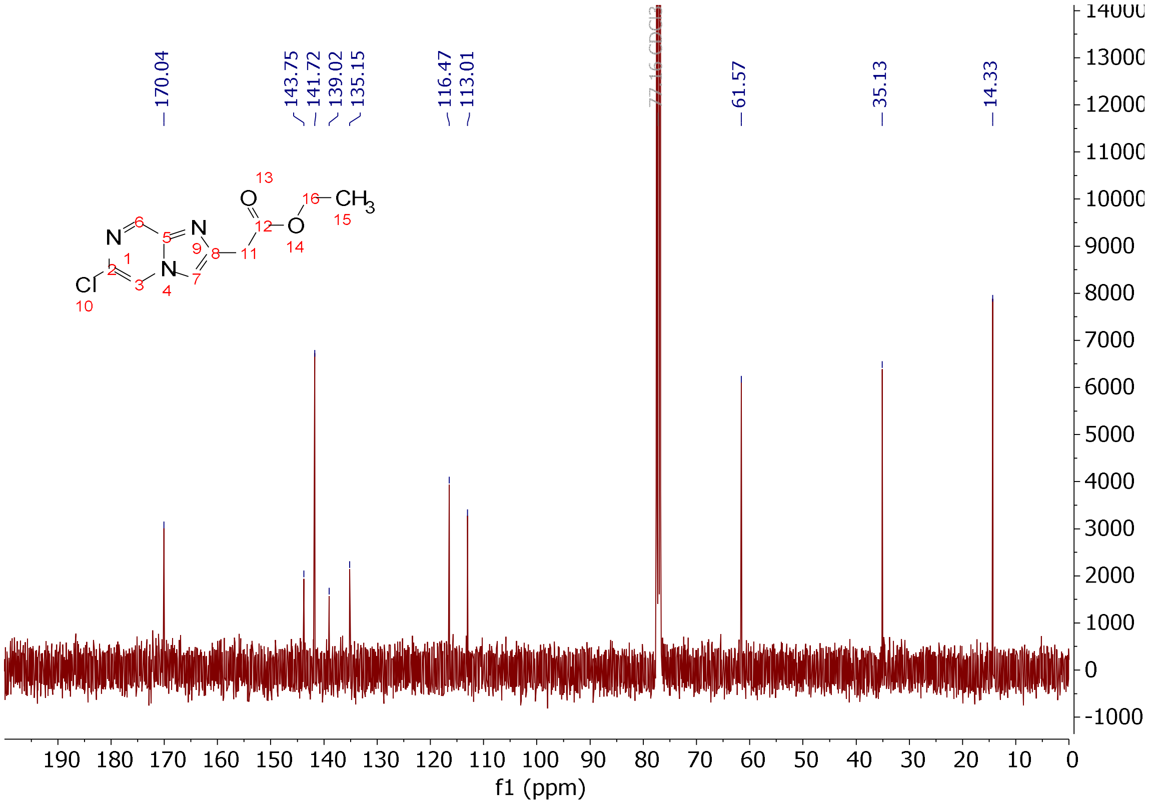
170.04
143.75
141.72
139.02
135.15
116.47
113.01
77.16 CDCl3
61.57
35.13
14.33
f1 (ppm)

HRMS data:

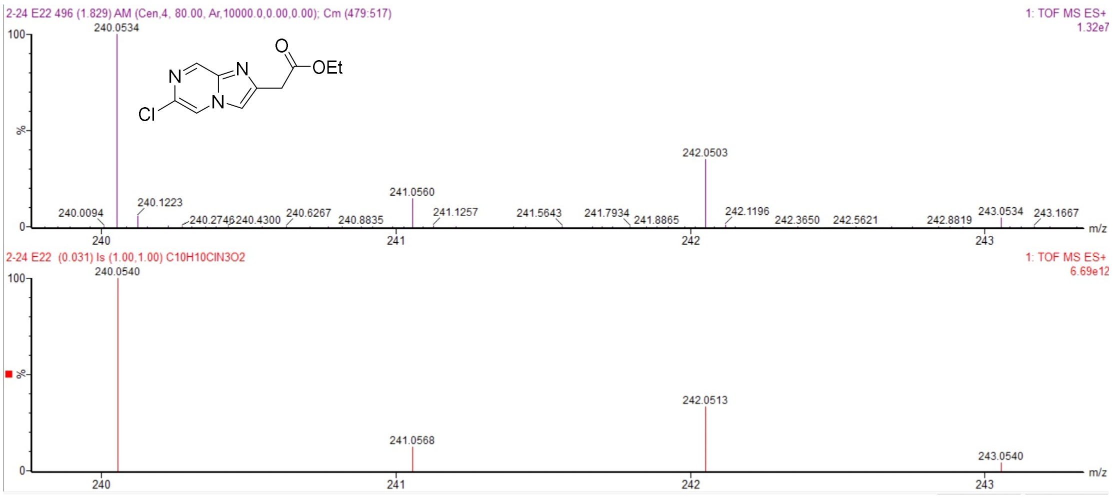
2-24 E22 496 (1.829) AM (Cen,4, 80.00, Ar,10000.0,0.00,0.00); Cm (479:517)
1: TOF MS ES+
1.32e7
240.0534
242.0503
241.0560
2-24 E22 (0.031) Is (1.00,1.00) C10H10ClN3O2
1: TOF MS ES+
6.69e12
240.0540
242.0513
241.0568
243.0540
m/z

Compound 3 *– Ethyl 2-aceto-dihydroimidazo[1,2-a]pyrazin-8(7H)-one*

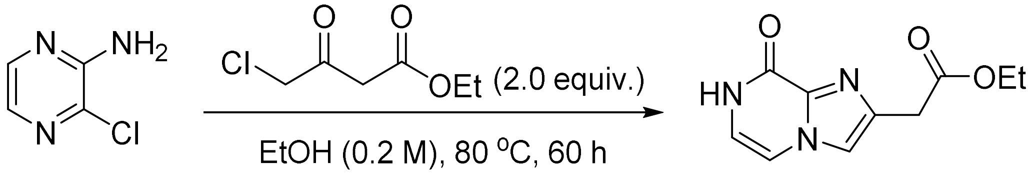

To a 10 mL RBF was added 2-amino-3-chloropyrazine (105.8 mg, 0.837 mmol) and EtOH (0.2 M, 4.2 mL). Ethyl 4-chloroacetoacetate (0.226 mL, 2 equiv.) was added and the mixture was heated to reflux for 60 h. The mixture was allowed to cool to RT and the solvent removed *in vacuo.* The residue was redissolved in $NaHCO_3$ sat. soln. (20 mL) and extracted with DCM (3x 20 mL). The organic washings were collected and washed with brine (20 mL) dried (Na_2SO_4), and solvent removed *in vacuo.* Purified *via* column chromatography (1% MeOH in DCM) to yield ethyl 2-acetoimidazo[1,2-*a*]pyrazin-8(7*H*)-one (78 mg, 0.35 mmol, 42%).

^{1}H NMR (400 MHz, C*D*Cl_3) δ 11.31 (br s, 1H), 7.50 (d, J = 0.8 Hz, 1H), 7.13 (d, J = 5.6 Hz, 1H), 6.86 (s, 1H), 4.20 (q, J = 7.1 Hz, 2H), 3.87 (d, J = 0.8 Hz, 2H), 1.29 (t, J = 7.1 Hz, 3H).

^{13}C NMR (101 MHz, C*D*Cl_3) δ 170.7, 155.7, 139.9, 136.9, 116.7, 115.6, 107.8, 61.3, 34.9, 14.3.

IR (cm^{-1}): 3043 (NH), 1723 (C=O).

M.P. 190.3 – 195.0 °C.

MS ES+ calcd. For $C_{10}H_{11}N_3O_3$ $(M+H)^+$: 223.0907, found: 223.0904.

Adapted from literature procedure[20].

NMR data:

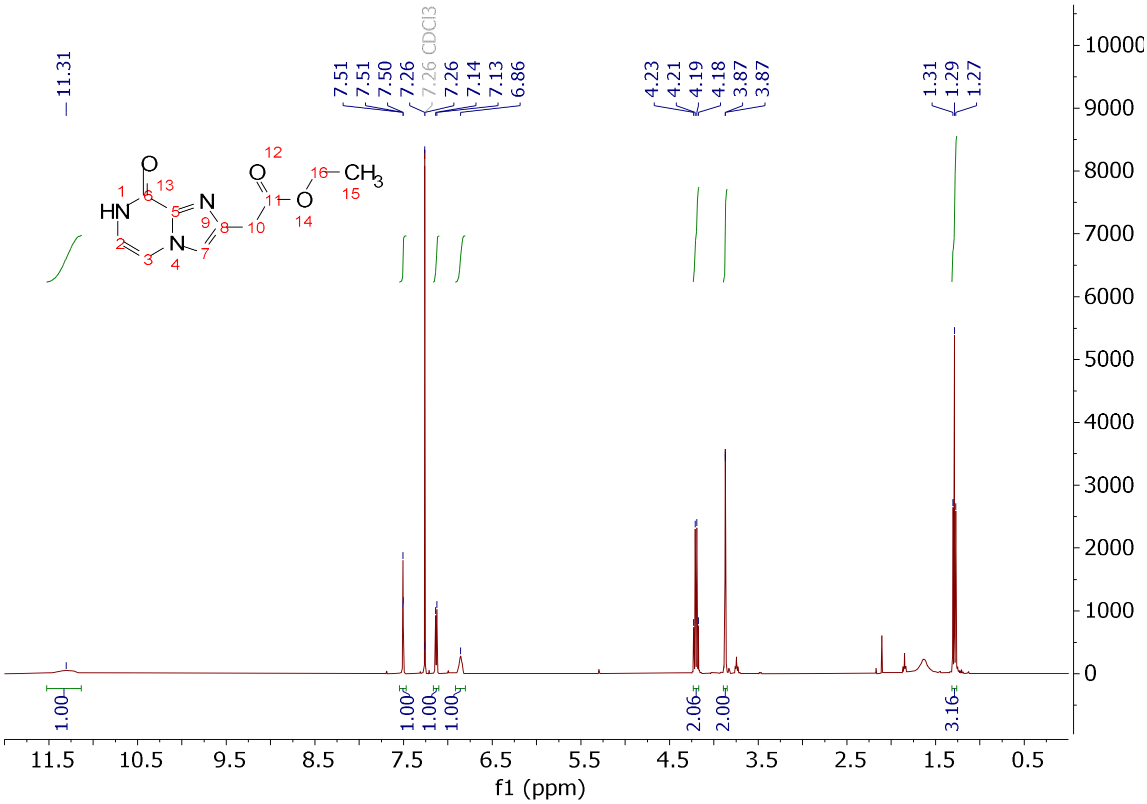
11.31
7.51
7.51
7.50
7.26
7.26 CDCl3
7.26
7.14
7.13
6.86
4.23
4.21
4.19
4.18
3.87
3.87
1.31
1.29
1.27
1.00
1.00
1.00
1.00
2.06
2.00
3.16
f1 (ppm)

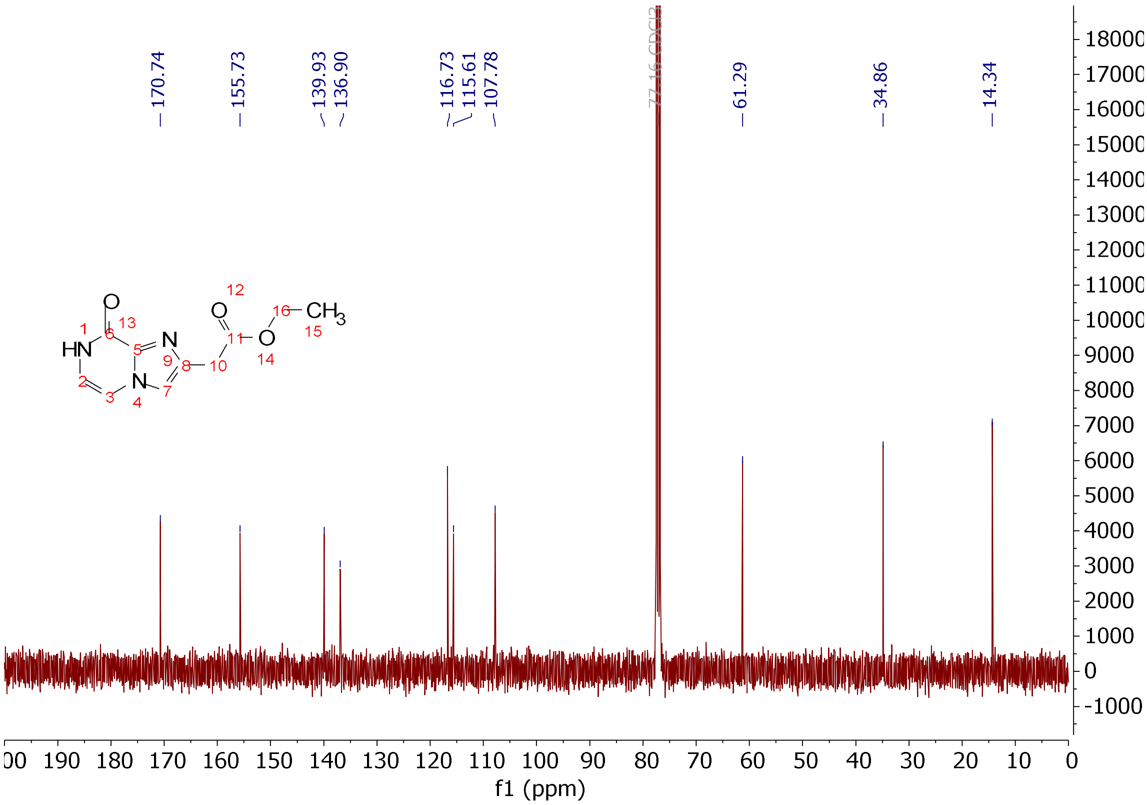
170.74
155.73
139.93
136.90
116.73
115.61
107.78
61.29
34.86
14.34
f1 (ppm)

HRMS data:

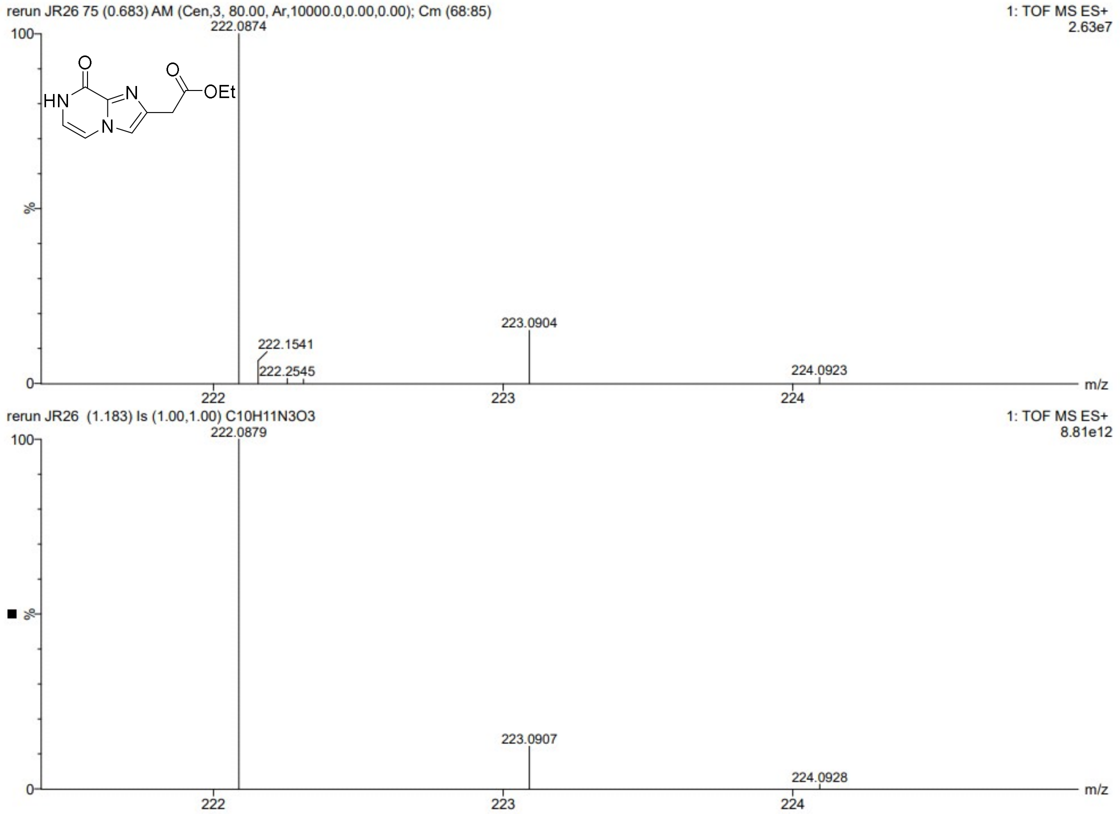

rerun JR26 75 (0.683) AM (Cen,3, 80.00, Ar,10000.0,0.00,0.00); Cm (68:85)
1: TOF MS ES+
2.63e7
222.0874
100
%
0
HN
O
N
N
O
OEt
222.1541
222.2545
223.0904
224.0923
222
223
224
m/z
rerun JR26 (1.183) Is (1.00,1.00) C10H11N3O3
1: TOF MS ES+
8.81e12
222.0879
223.0907
224.0928
m/z

***Compound 4** – Ethyl 6,8-Dichloroimidazo[1,2-a]pyrazine-2-acetate*

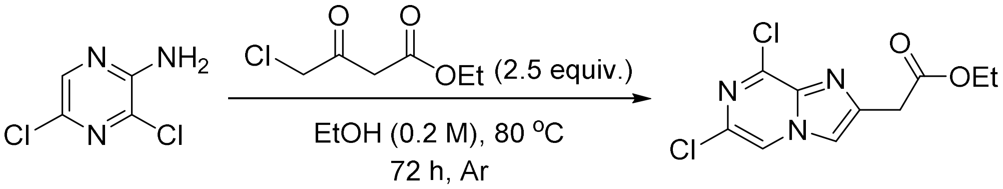

To a 50 mL RBF was added 2-amino-3,5-dichloropyrazine (0.19 g, 1.16 mmol) and EtOH (over mol. sieves, 6 mL, 0.2 M). To the stirred solution was added ethyl 4-chloroacetoacetate (0.235 mL, 1.5 equiv.) This was heated to reflux at 80 °C for 18 h. Afterwards, additional ethyl 4-chloroacetoacetate (0.16 mL, 1.0 equiv.) was added and reflux was continued for 48 h. After allowing the mixture to cool to RT, the solvent was removed *in vacuo.* and dissolved in $NaHCO_3$ sat. soln. (20 mL). This was extracted with DCM (3x 20 mL), the organic layers were collected, washed with brine (20 mL), dried (Na_2SO_4) and solvent removed *in vacuo.* the residue was purified *via* column chromatography (9:1 -> 7:3 Hexane : EtOAc) to yield ethyl 6,8-dichloroimidazo[1,2-*a*]pyrazine-2-acetate as a beige solid (132 mg, 0.48 mmol, 41%).

^{1}H NMR (400 MHz, C*D*Cl$_3$) δ 8.09 (s, 1H), 7.87 (t, J = 0.7 Hz, 1H), 4.22 (q, J = 7.1 Hz, 2H), 3.97 (d, J = 0.7 Hz, 2H), 1.30 (t, J = 7.1 Hz, 3H).

^{13}C NMR (101 MHz, C*D*Cl$_3$) δ 170.0, 144.2, 141.7, 136.6, 132.4, 116.2, 115.1, 61.6, 35.2, 14.3.

IR (cm^{-1}): 1716 (C=O).

M.P. 96.8 – 97.1 °C.

MS ES+ Calcd for $C_{10}H_9Cl_2N_3O_2$ $(M+H)^+$: 278.0096, found: 278.0090.

Adapted from literature procedure, data in-line with literature data[20].

NMR data:

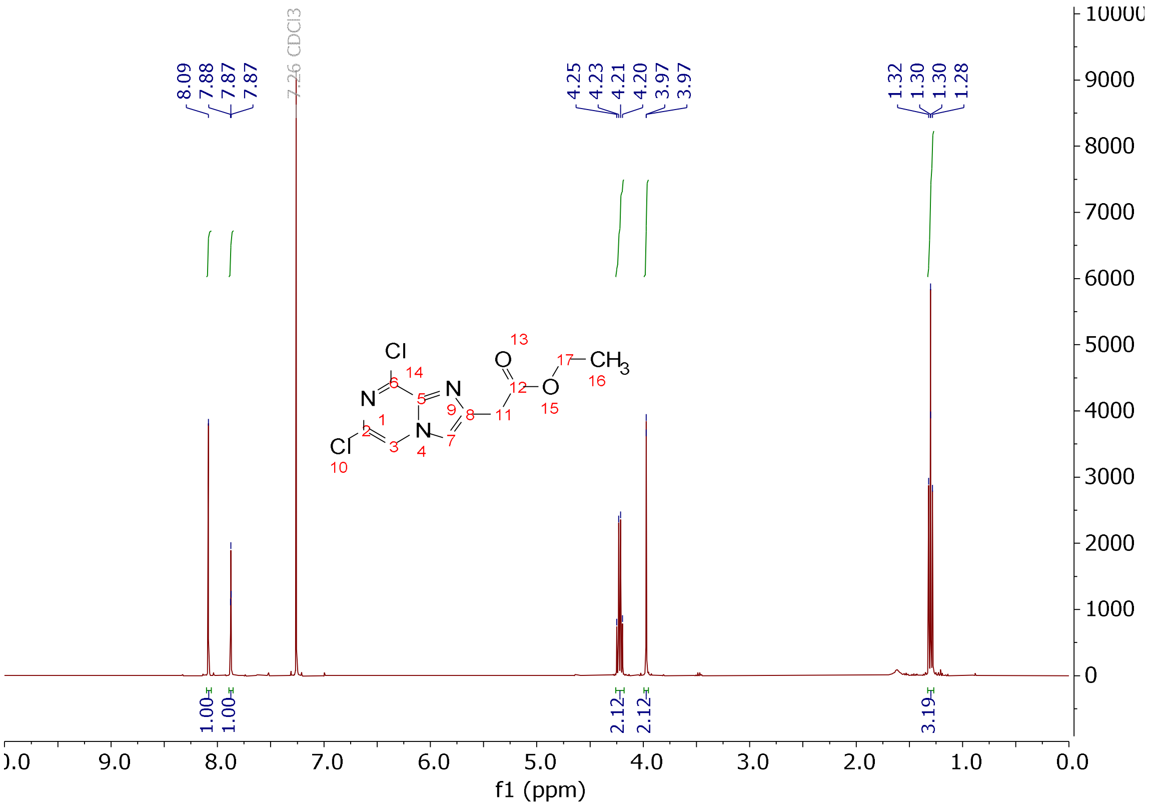
8.09
7.88
7.87
7.87
7.26 CDCl3
4.25
4.23
4.21
4.20
3.97
3.97
1.32
1.30
1.30
1.28
1.00
1.00
2.12
2.12
3.19
f1 (ppm)

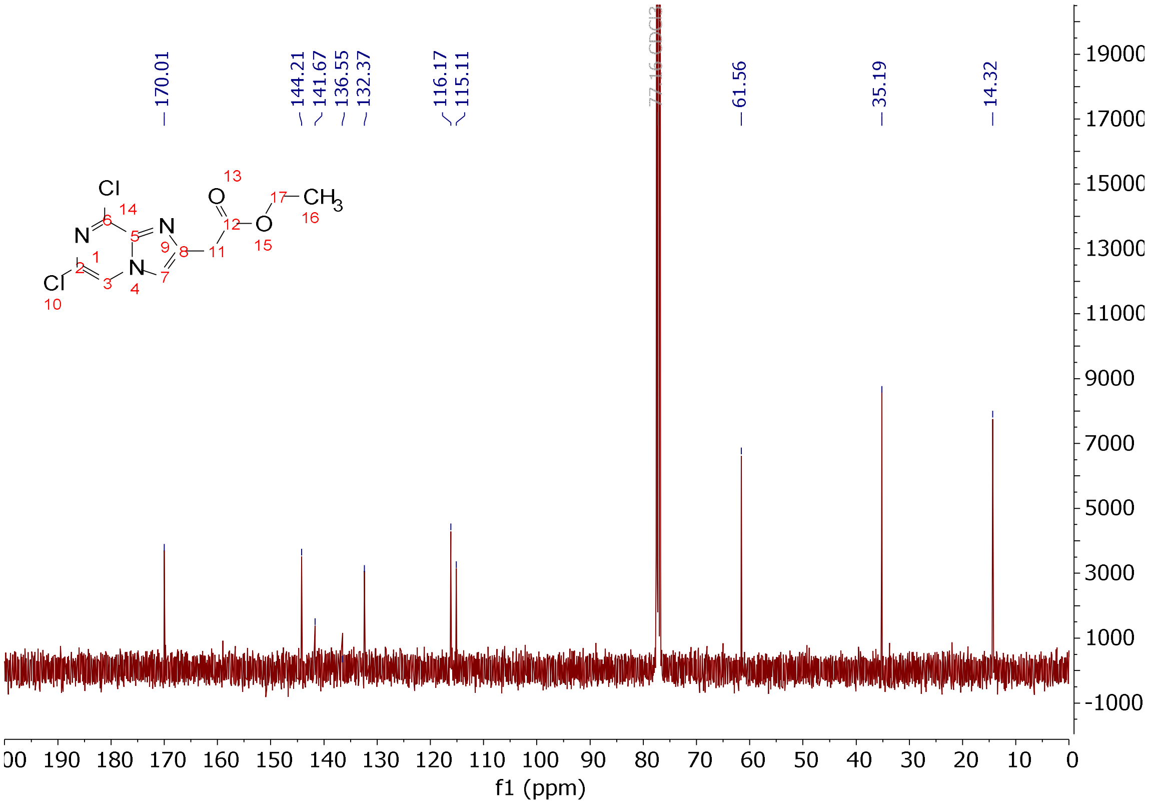
170.01
144.21
141.67
136.55
132.37
116.17
115.11
77.16 CDCl3
61.56
35.19
14.32
f1 (ppm)

HRMS data:

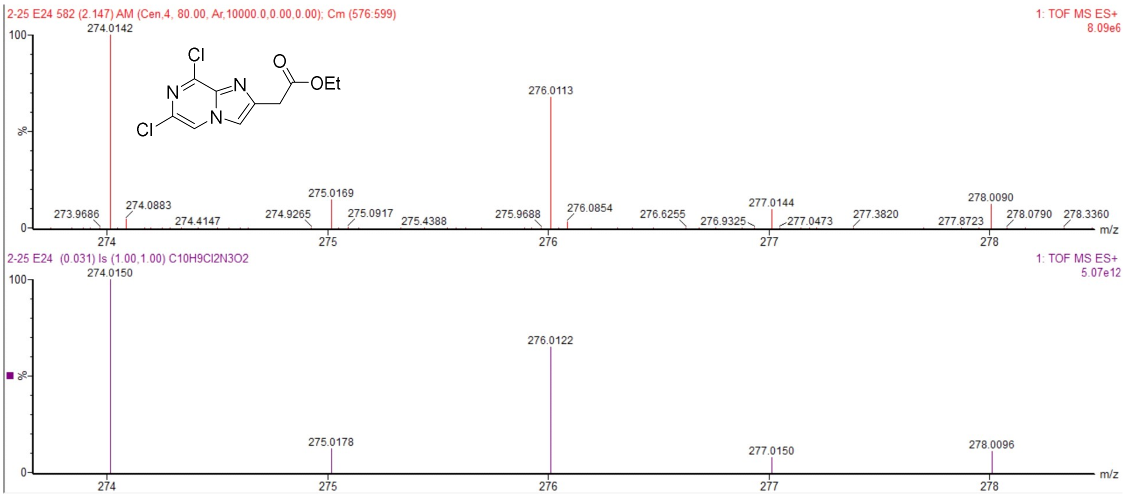
2-25 E24 582 (2.147) AM (Cen,4, 80.00, Ar,10000.0,0.00,0.00); Cm (576:599)
1: TOF MS ES+
8.09e6
274.0142
276.0113
275.0169
273.9686
274.0883
274.4147
274.9265
275.0917
275.4388
275.9688
276.0854
276.6255
276.9325
277.0144
277.0473
277.3820
277.8723
278.0090
278.0790
278.3360
Cl
N
N
N
O
OEt
Cl
%
m/z
2-25 E24 (0.031) Is (1.00,1.00) C10H9Cl2N3O2
1: TOF MS ES+
5.07e12
274.0150
276.0122
275.0178
277.0150
278.0096

Compound 5 – *Ethyl 2-({5-chloro-1H-imidazo[4,5-b]pyrazine-2-yl}sulfanyl)acetate*

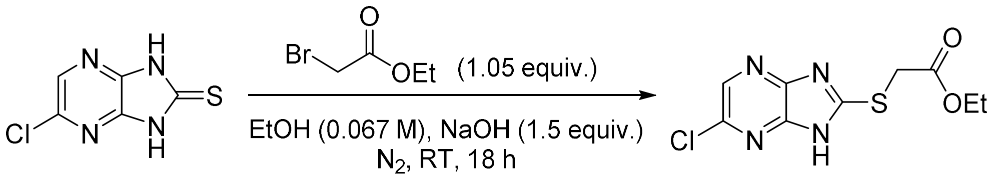

To a 50 mL RBF was added 5-chloroimidazo[4,5-*b*]pyrazine-2(1,3*H*)-thione (442 mg, 2.37 mmol), sodium hydroxide (0.14 g, 1.5 equiv.) and ethanol (35 mL, 0.067 M). To the stirred solution was added ethyl bromoacetate (0.275 mL, 1.05 equiv.) and the reaction left to stir for 18 h. The ethanol was removed *in vacuo.* Sat. soln. of NH_4Cl and EtOAc were added to dissolve the solid residue (10 mL each), and this was transferred to a separatory funnel, separated and the aqueous layer extracted with EtOAc (3x 30 mL). The organic layers were collected and dried, loaded onto silica and purified by column chromatography eluting with 8:2 -> 7:3 Pet.E : EtOAc followed by triturating with *n*-hexane after recrystallisation from ethanol. (170 mg, 0.62 mmol, 26%).

^{1}H NMR (500 MHz, CD_3CN) δ 11.15 (br s, 1H), 8.23 (s, 1H), 4.19 (q, J = 7.1 Hz, 2H), 4.17 (s, 2H), 1.24 (t, J = 7.1 Hz, 3H).

^{13}C NMR (126 MHz, CD_3CN) δ 169.0, 159.4, 142.6, 137.2, 62.8, 34.2, 14.4 (7 out of 9 carbon resonances observed).

IR (cm^{-1}): 1737 (C=O).

M.P. 118.7 – 119.2 °C.

MS ES+ m/z calcd for $C_9H_9ClN_4O_2S$ $(M+H)^+$: 273.0213, found: 273.0213.

Adapted from literature procedure[5].

NMR data:

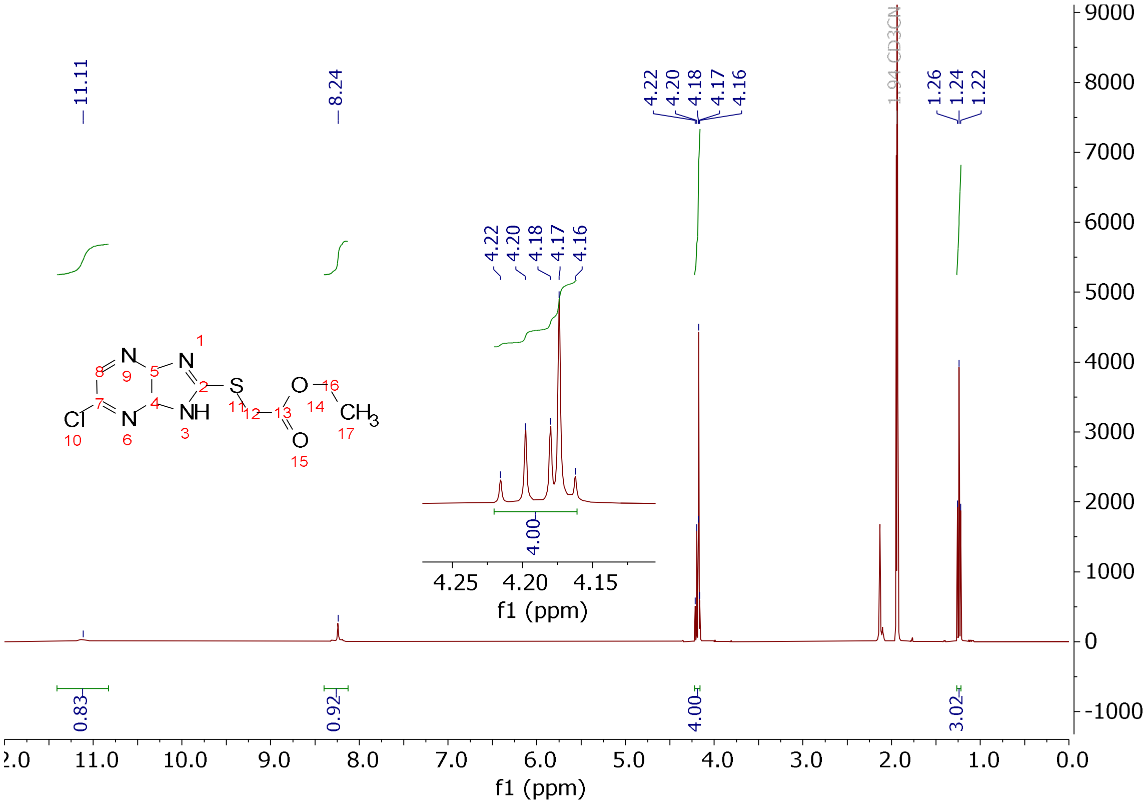
11.11
8.24
4.22
4.20
4.18
4.17
4.16
1.94 CD3CN
1.26
1.24
1.22
0.83
0.92
4.00
3.02
f1 (ppm)

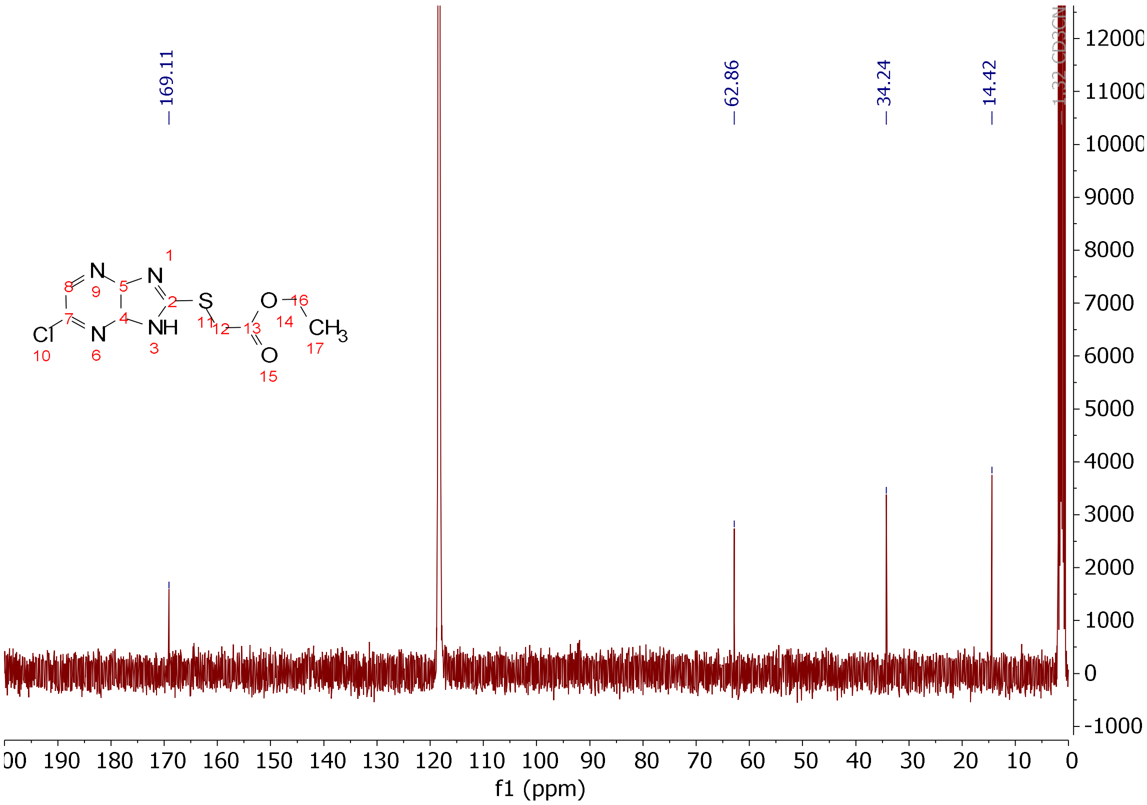
169.11
62.86
34.24
14.42
1.32 CD3CN
f1 (ppm)

HRMS data:

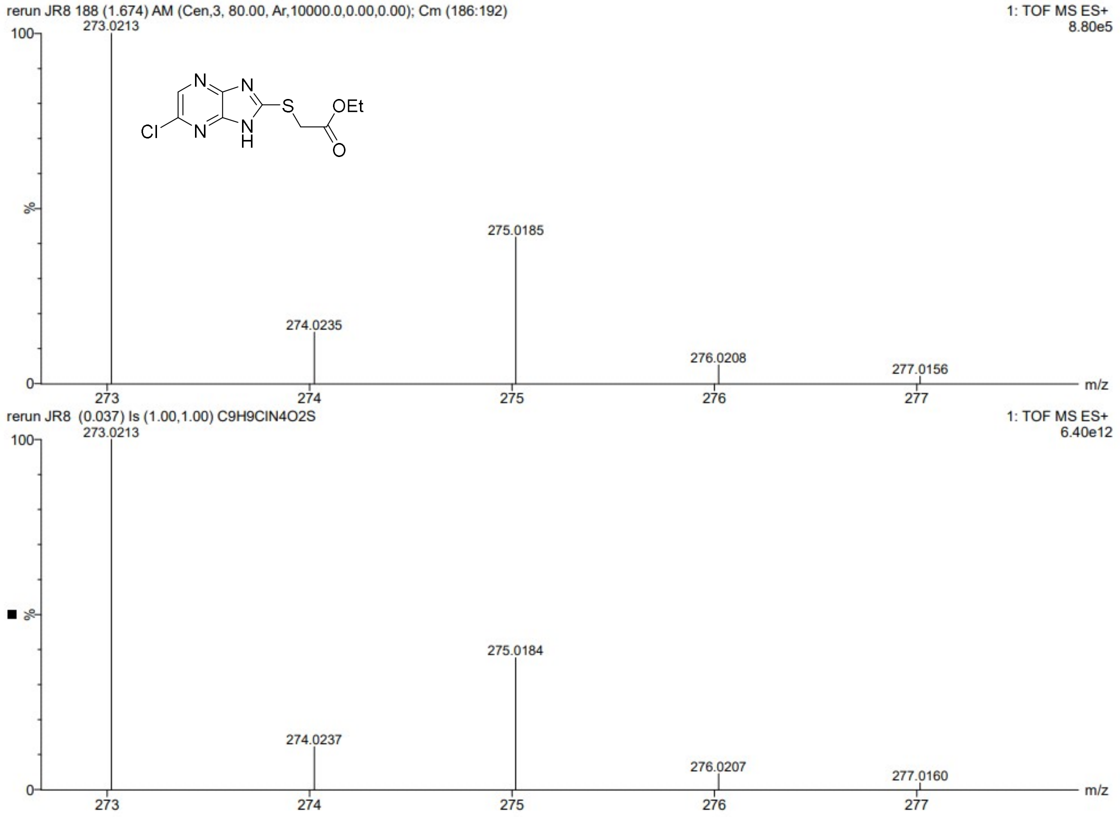
rerun JR8 188 (1.674) AM (Cen,3, 80.00, Ar,10000.0,0.00,0.00); Cm (186:192)
1: TOF MS ES+
8.80e5
273.0213
274.0235
275.0185
276.0208
277.0156
100
%
0
273
274
275
276
277
m/z
Cl
N
N
H
S
OEt
O
rerun JR8 (0.037) Is (1.00,1.00) C9H9ClN4O2S
1: TOF MS ES+
6.40e12
273.0213
274.0237
275.0184
276.0207
277.0160

Compound 6/NSC-217913 *– Ethyl 2-({5,6-dichloro-1H-imidazo[4,5-b]pyrazine-2-yl}sulfanyl)-acetate*

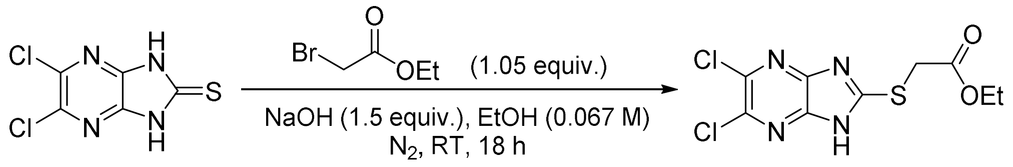

To an 8 mL vial was added 5,6-dichloroimidazo[4,5-*b*]pyrazine-2(1,3*H*)-thione (100 mg, 0.452 mmol) and sodium hydroxide (28 mg, 1.5 equiv.). The vial was then sealed with a suba seal and wrapped with parafilm. Ethanol (6.75 mL, 0.067 M) was added *via* syringe and the mixture stirred until all solids had dissolved. Ethyl bromoacetate (53 µL, 1.05 equiv.) was added *via* microsyringe and the reaction was left to stir for 18 h. The solvent was removed under reduced pressure and the solid redissolved in NH_4Cl solution and EtOAc (*ca.* 5 mL each) and the mixture transferred to a separatory funnel. The organic layer was removed, and the aqueous layer extracted with EtOAc (3x 10 mL). The organic layers were collected and dried ($MgSO_4$) and solvent removed *in vacuo.* The crude material was purified by column chromatography eluting with Pet. E. : EtOAc (7:3) followed by triturating with cold *n*-hexane (*ca.* 4 mL) and recrystallising from ethanol to provide a white solid of ethyl 2-({5,6-dichloro-1*H*-imidazo[4,5-*b*]pyrazine-2-yl}sulfanyl)-acetate (106 mg, 0.344 mmol, 76%).

^{1}H NMR (400 MHz, CD_3CN) δ 4.19 (q, J = 7.1 Hz, 2H), 4.17 (s, 2H), 1.24 (t, J = 7.1 Hz, 3H).

^{13}C NMR (126 MHz, CD_3CN) δ 168.9, 160.7, 139.5, 62.9, 34.3, 14.4, (6 out of 7 carbon resonances observed).

IR (cm^{-1}) 3084 (NH), 1731 (C=O), 1595 (NH bend).

M.P. 167.6 – 168.1 °C.

MS ES+ m/z calcd for $C_9H_8Cl_2N_4O_2S$ $(M+H)^+$ 308.9794, found: 308.9792.

Adapted from literature procedure[5].

NMR data:

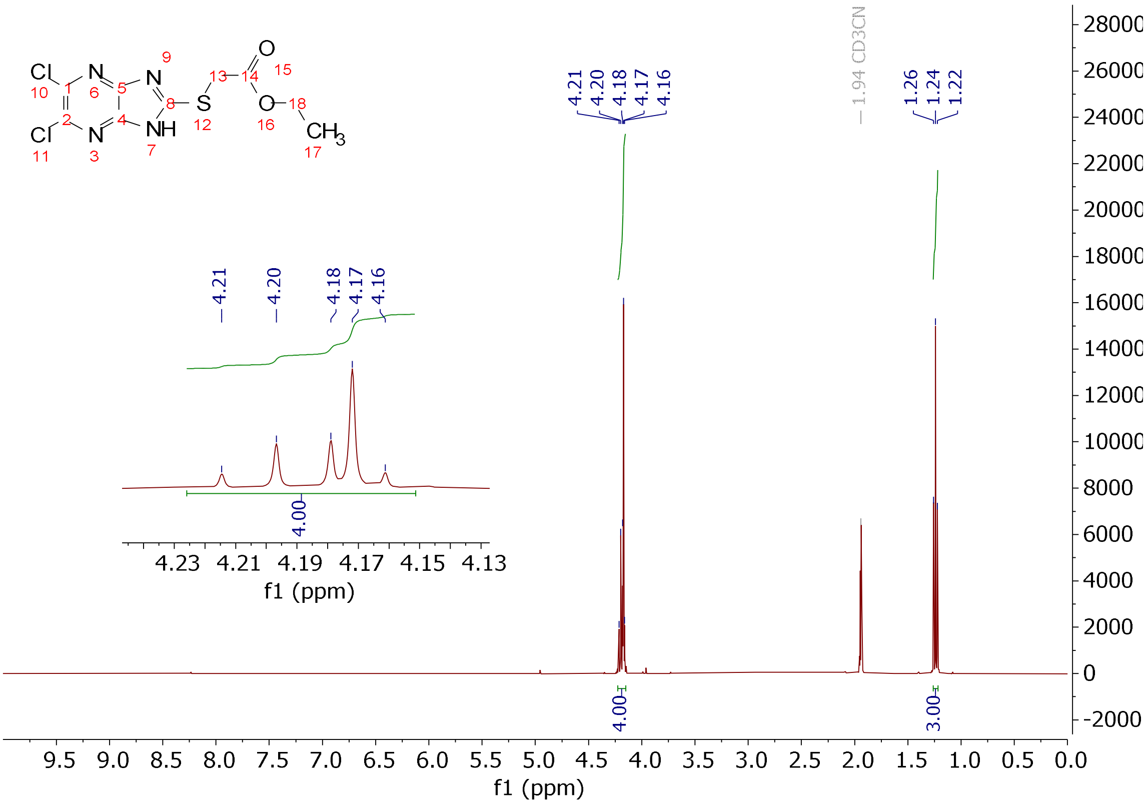

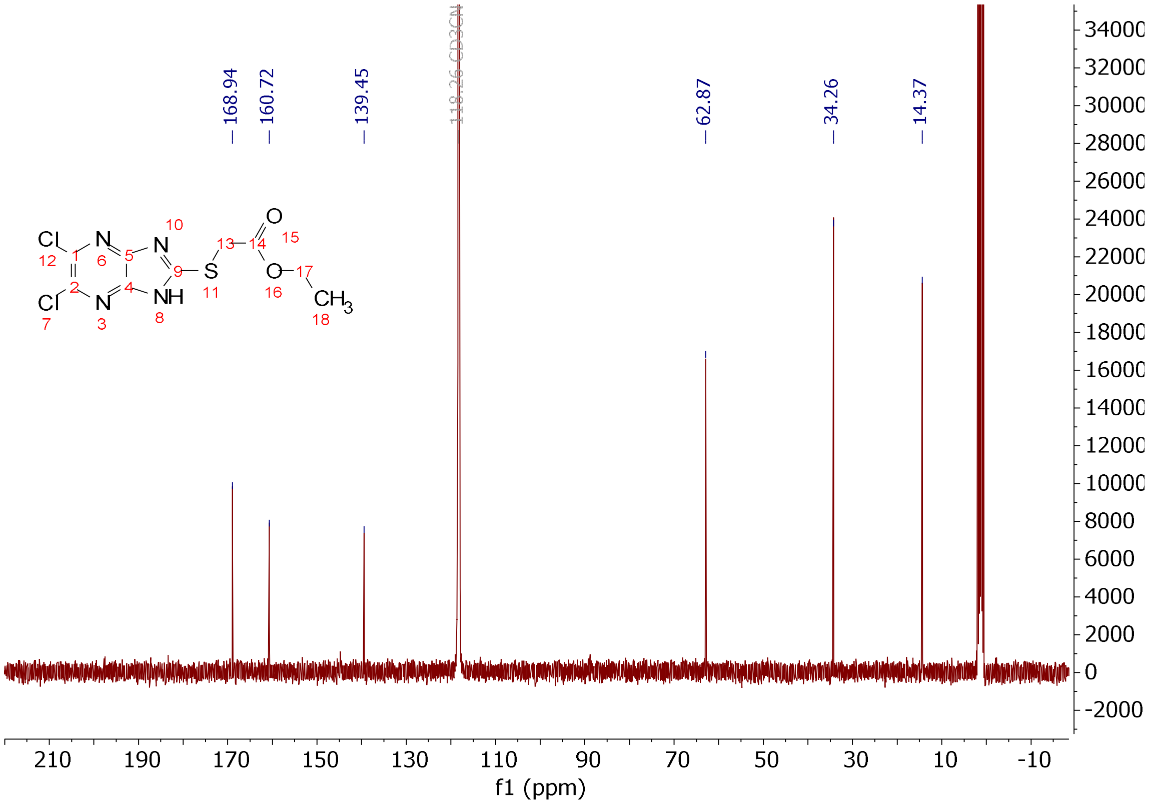
168.94
160.72
139.45
118.26 CD3CN
62.87
34.26
14.37
f1 (ppm)

HRMS data:

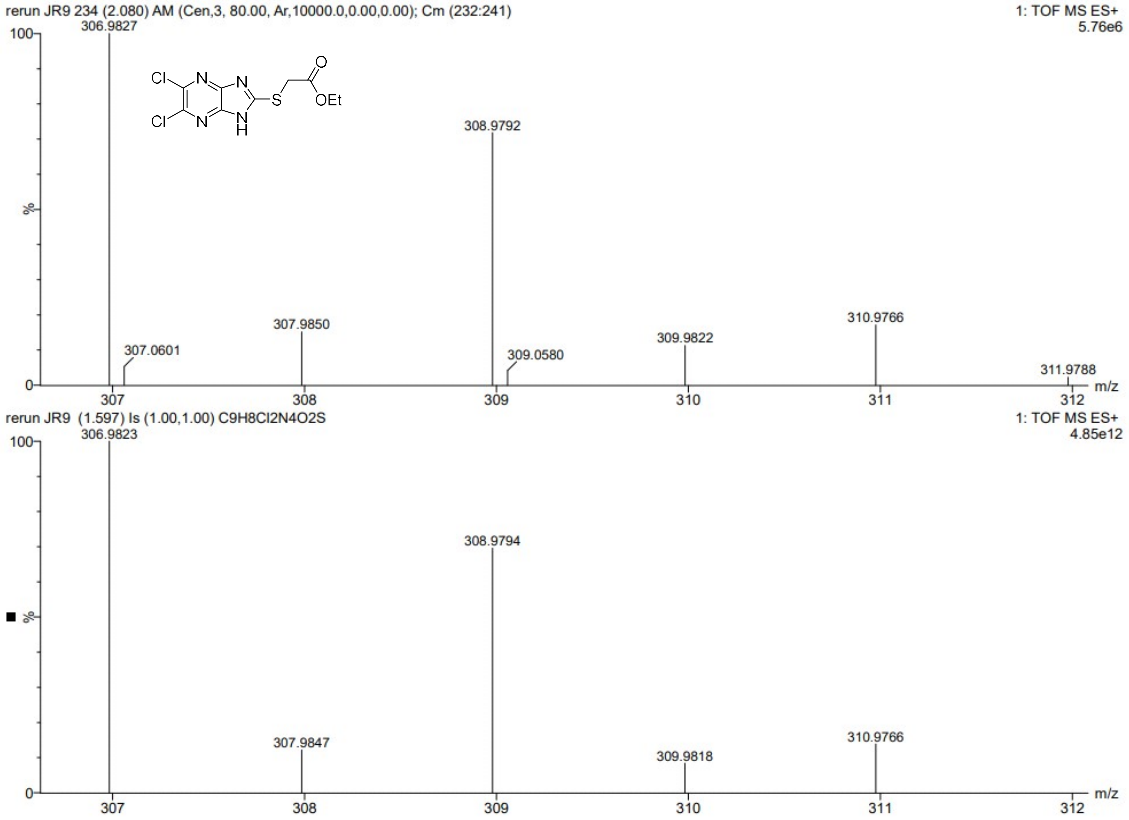
rerun JR9 234 (2.080) AM (Cen,3, 80.00, Ar,10000.0,0.00,0.00); Cm (232:241)
1: TOF MS ES+
5.76e6
306.9827
308.9792
307.9850
310.9766
309.9822
307.0601
309.0580
311.9788
rerun JR9 (1.597) Is (1.00,1.00) C9H8Cl2N4O2S
1: TOF MS ES+
4.85e12
306.9823
308.9794
307.9847
310.9766
309.9818
m/z

__Compound__ 7 – 2-({5,6-Dichloro-1H-imidazo[4,5-b]pyrazine-2-yl}sulfanyl)acetonitrile

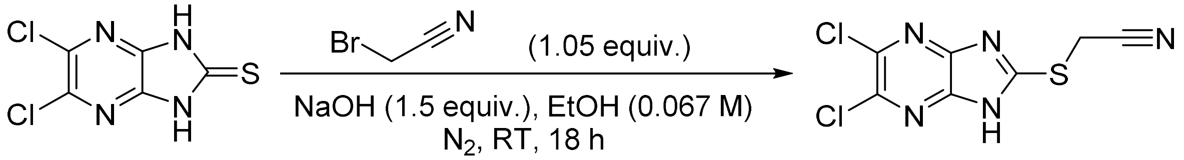

To an 8 mL vial was added 5,6-dichloroimidazo[4,5-*b*]pyrazine-2(1,3*H*)-thione (100 mg, 0.452 mmol) and sodium hydroxide (28 mg, 1.5 equiv.). The vial was then sealed with a suba seal and wrapped with parafilm. Ethanol (6.75 mL, 0.067 M) was added *via* syringe and the mixture stirred until all solids had dissolved. Bromoacetonitrile (33 µL, 1.05 equiv.) was added *via* microsyringe, and the reaction was left to stir for 18 h. The solvent was removed under reduced pressure and the solid redissolved in ammonium chloride solution and ethanol (*ca.* 5 mL each) and the mixture transferred to a separatory funnel. Separation of the single-phase mixture was achieved by adding a small amount of brine solution. The organic layer was removed, and the aqueous layer extracted with EtOAc (3x 10 mL). The organic layers were collected and dried ($MgSO_4$) and solvent removed *in vacuo.* The crude material was purified by column chromatography eluting with Pet. E. : EtOAc (7:3) followed by triturating with cold *n*-hexane (*ca.* 4 mL) and recrystallising from ethanol to provide a light-yellow solid of 2-({5,6-dichloro-1*H*-imidazo[4,5-*b*]pyrazine-2-yl}sulfanyl)acetonitrile (63.3 mg, 0.243 mmol, 54%).

^{1}H NMR (400 MHz, CD_3CN) δ 4.25 (s, 2H).

^{13}C NMR (101 MHz, CD_3CN) δ 158.3, 140.0, 117.5, 18.0 (4 out of 5 carbon resonances observed).

IR (cm^{-1}) 3100 (NH), 2248 (C≡N).

M.P. 213.5 – 216 ºC (deg.).

MS ES+ m/z calcd for $C_7H_3Cl_2N_5S$ $(M+H)^+$: 260.9584, found: 260.9583.

Adapted from literature procedure[5].

NMR data:

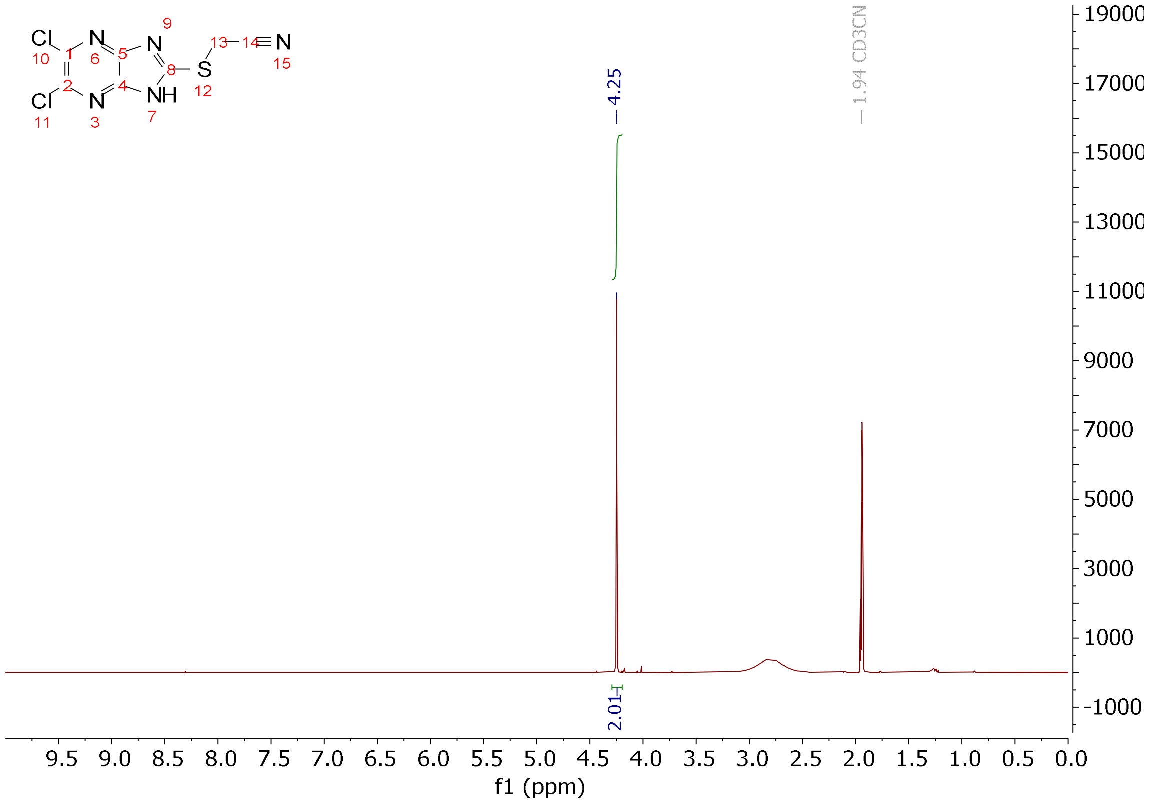

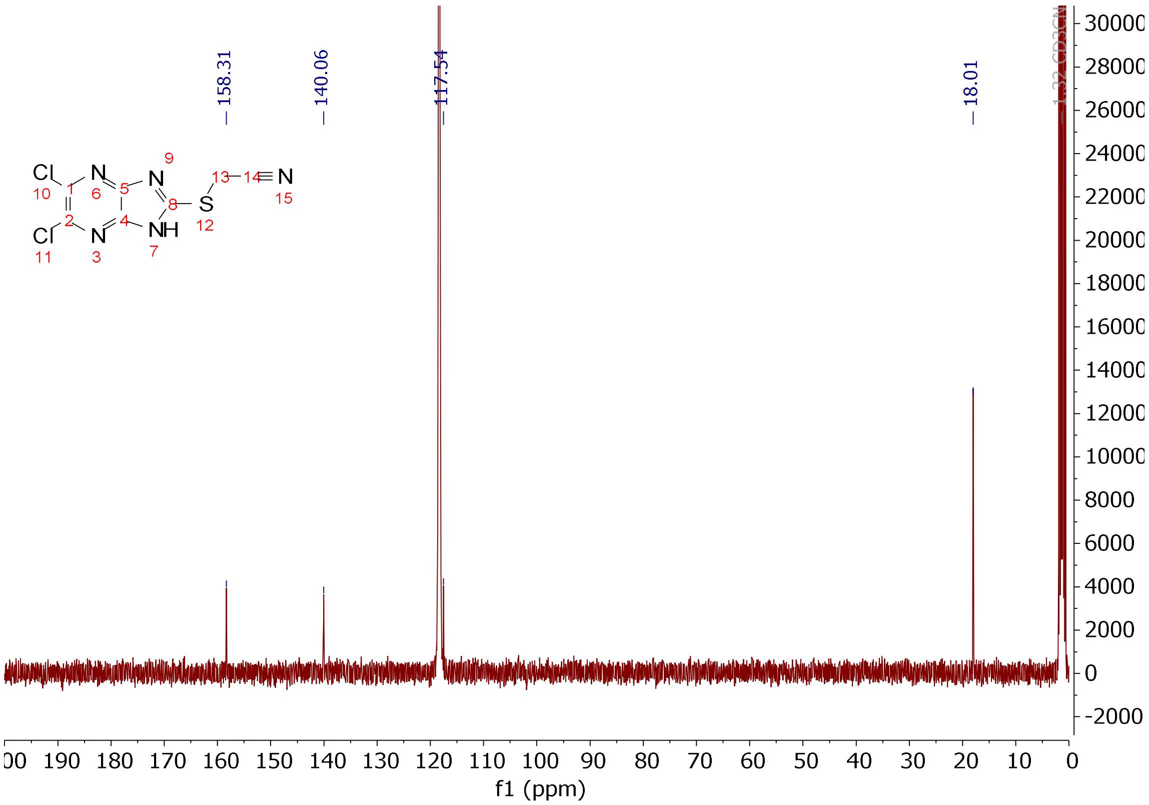
158.31
140.06
117.54
18.01
Cl
N
N
S
N
NH
Cl
N
f1 (ppm)

HRMS data:

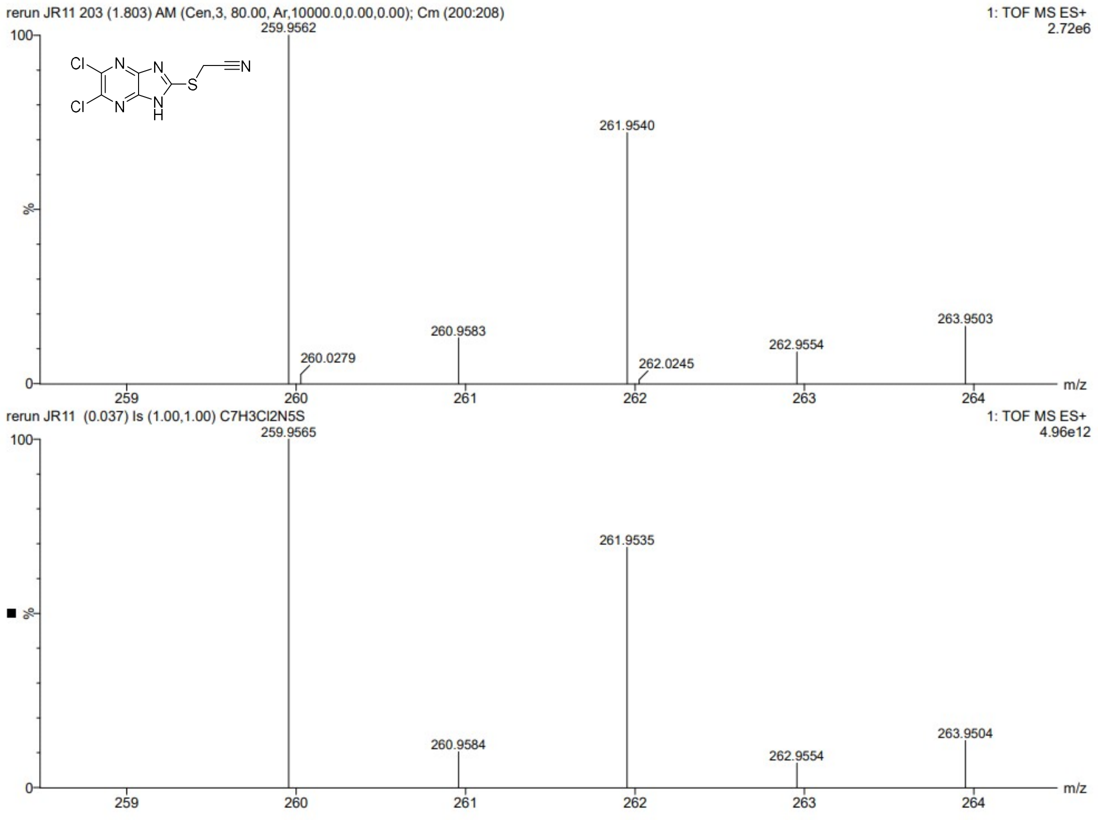
rerun JR11 203 (1.803) AM (Cen,3, 80.00, Ar,10000.0,0.00,0.00); Cm (200:208)
1: TOF MS ES+
2.72e6
259.9562
261.9540
260.9583
260.0279
262.0245
262.9554
263.9503
rerun JR11 (0.037) Is (1.00,1.00) C7H3Cl2N5S
1: TOF MS ES+
4.96e12
259.9565
261.9535
260.9584
262.9554
263.9504
m/z

Compound 8 *– 2-({5,6-dichloro-1H-imidazo[4,5-b]pyrazine-2-yl}sulfanyl)pentane*

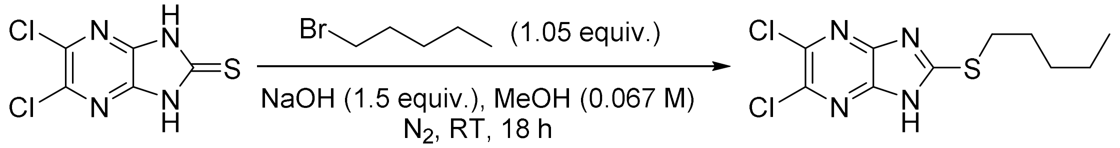

To an 8 mL vial was added 5,6-dichloroimidazo[4,5-*b*]pyrazine-2(1,3*H*)-thione (100 mg, 0.452 mmol) and sodium hydroxide (28 mg, 1.5 equiv.). The vial was then sealed with a suba seal and wrapped with parafilm. Methanol (6.75 mL, 0.067 M) was added *via* syringe and the mixture stirred until all solids had dissolved. 1-Bromopentane (59 µL, 1.05 equiv.) was added *via* microsyringe, and the reaction was left to stir for 18 h. The solvent was removed under reduced pressure and the solid redissolved in NH_4Cl solution and EtOAc (*ca.* 5 mL each) and the mixture transferred to a separatory funnel. The organic layer was removed, and aqueous layer extracted with EtOAc (3x 10 mL). The organic layers were collected and dried ($MgSO_4$) and solvent removed *in vacuo.* The crude material was purified by column chromatography eluting with Pet. E. : EtOAc (95:5) to provide 2-({5,6-dichloro-1*H*-imidazo[4,5-*b*]pyrazine-2-yl}sulfanyl)pentane as a white solid which was further triturated cold *n*-hexane (*ca.* 4 mL) (62.1 mg, 0.213 mmol, 47%).

^{1}H NMR (500 MHz, C*D*$_3$CN) *δ* 11.16 (br s, 1H), 3.43 – 3.31 (m, 2H), 1.86 – 1.76 (m, 2H), 1.50 – 1.40 (m, 2H), 1.40 – 1.29 (m, 2H), 0.91 (t, *J* = 7.2 Hz, 3H).

^{13}C NMR (126 MHz, C*D*$_3$CN) *δ* 162.5, 145.0, 139.0, 32.2, 31.4, 29.8, 22.8, 14.2.

IR (cm^{-1}) 3088 (NH), 2959, 2930, 2861 (C(sp^3)-H).

M.P. 145.9 – 146.3 °C.

MS ES+ m/z calcd for $C_{10}H_{12}Cl_2N_4S$ $(M+H)^+$: 293.0208, found: 293.0209.

Adapted from literature procedure[5].

NMR data:

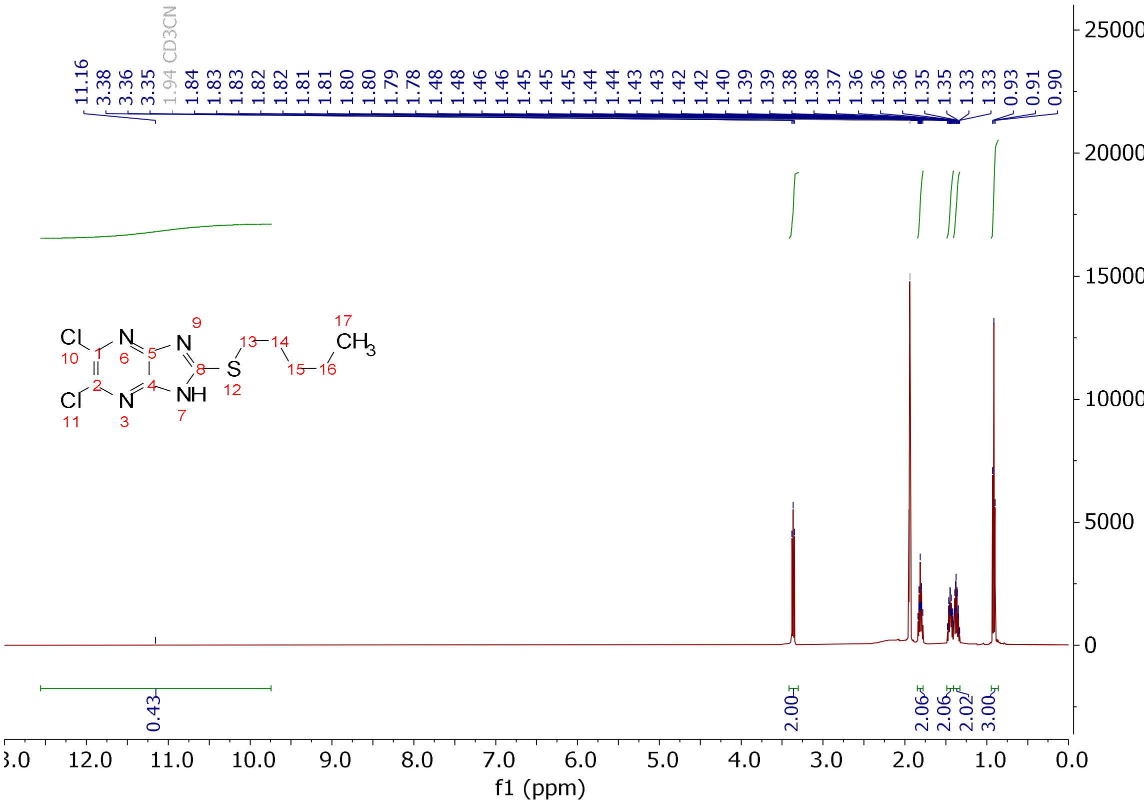

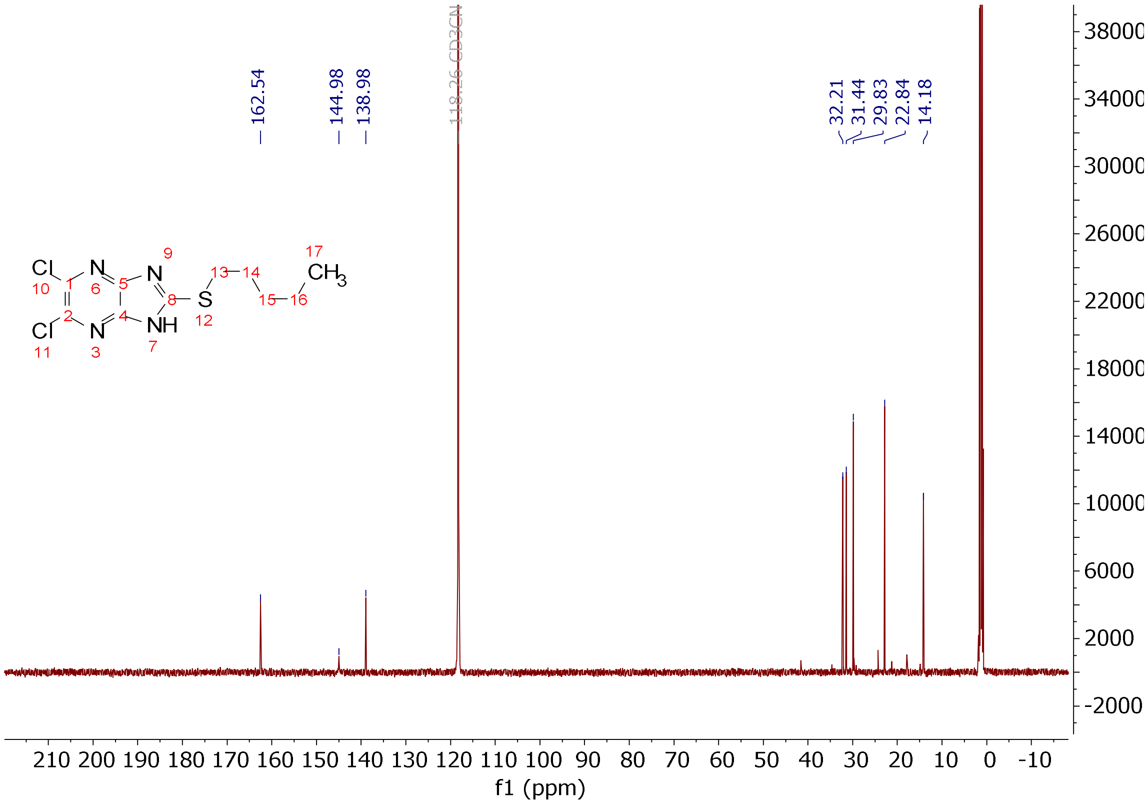

HRMS data:

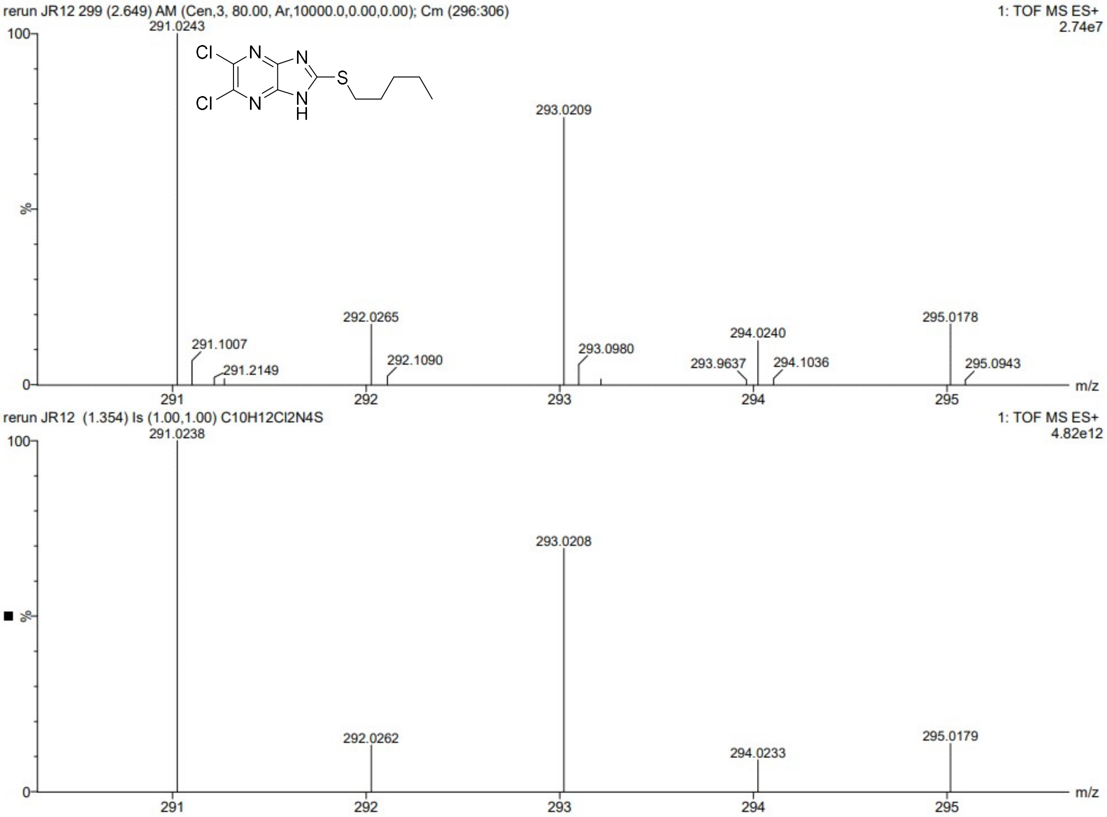
rerun JR12 299 (2.649) AM (Cen,3, 80.00, Ar,10000.0,0.00,0.00); Cm (296:306)
1: TOF MS ES+
2.74e7
291.0243
293.0209
292.0265
291.1007
291.2149
292.1090
293.0980
294.0240
293.9637
294.1036
295.0178
295.0943
Cl
N
H
S
%
m/z
rerun JR12 (1.354) Is (1.00,1.00) C10H12Cl2N4S
1: TOF MS ES+
4.82e12
291.0238
293.0208
292.0262
294.0233
295.0179
291
292
293
294
295

Compound 9 *– 2-({5,6-dichloro-1H-imidazo[4,5-b]pyrazine-2-yl}sulfanyl)acetylmorpholine*

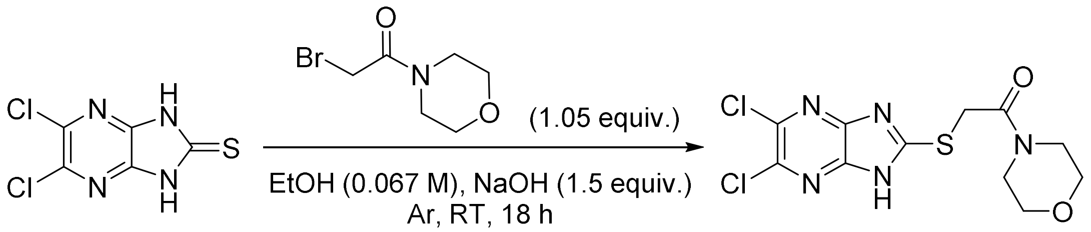

To an 8 mL vial was added a stirrer bar, 5,6-dichloroimidazo[4,5-*b*]pyrazine-2(1,3*H*)-thione (75 mg, 0.34 mmol), sodium hydroxide (20.3 mg, 1.5 equiv.). Ethanol (over mol. sieves, 5.0 mL) was added *via* syringe. *N*-(bromoacetyl)morpholine (74.1 mg, 1.05 equiv.) was added in one batch and the reaction placed under argon atmosphere. Left to stir at RT for 18 h. Afterwards, the solvent is removed *in vacuo.* and the residue redissolved in sat. soln. NH_4Cl (10 mL), and EtOAc (10 mL), transferred to a separatory funnel and the organic layer removed. Aqueous layer extracted with EtOAc (3x 10 mL), organic layers collected and dried ($MgSO_4$) and solvent removed *in vacuo.* The residue purified by column chromatography (6:4 Pet. E : EtOAc -> EtOAc) to provide an off-white solid of 2-({5,6-dichloro-1*H*-imidazo[4,5-*b*]pyrazine-2-yl}sulfanyl)acetylmorpholine (53.4 mg, 0.153 mmol, 45%).

^{1}H NMR (400 MHz, CD_3CN) δ 4.37 (s, 2H), 3.73 – 3.66 (m, 2H), 3.65 – 3.61 (m, 2H), 3.61 – 3.54 (m, 4H).

^{13}C NMR (101 MHz, CD_3CN) δ 166.8, 161.7, 139.3, 67.1, 67.1, 47.3, 43.4, 35.6 (8 out of 9 carbon resonances observed).

IR (cm^{-1}) 3143 (NH), 2771, 1623 (C=O).

M.P. 214.4 – 215.1 ^{o}C.

MS ES+ m/z calcd for $C_{11}H_{11}Cl_2N_5O_2S$ $(M+H)^+$: 349.0113, found: 349.0112.

Adapted from literature procedure[5].

NMR data:

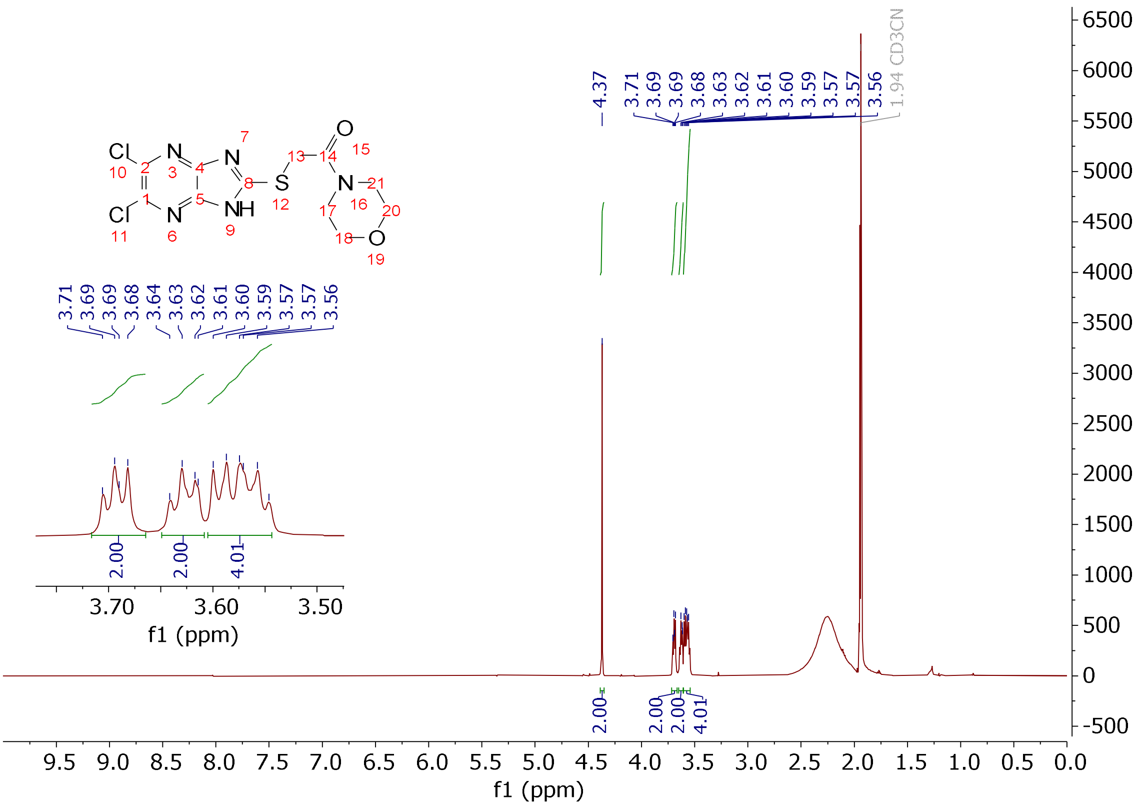

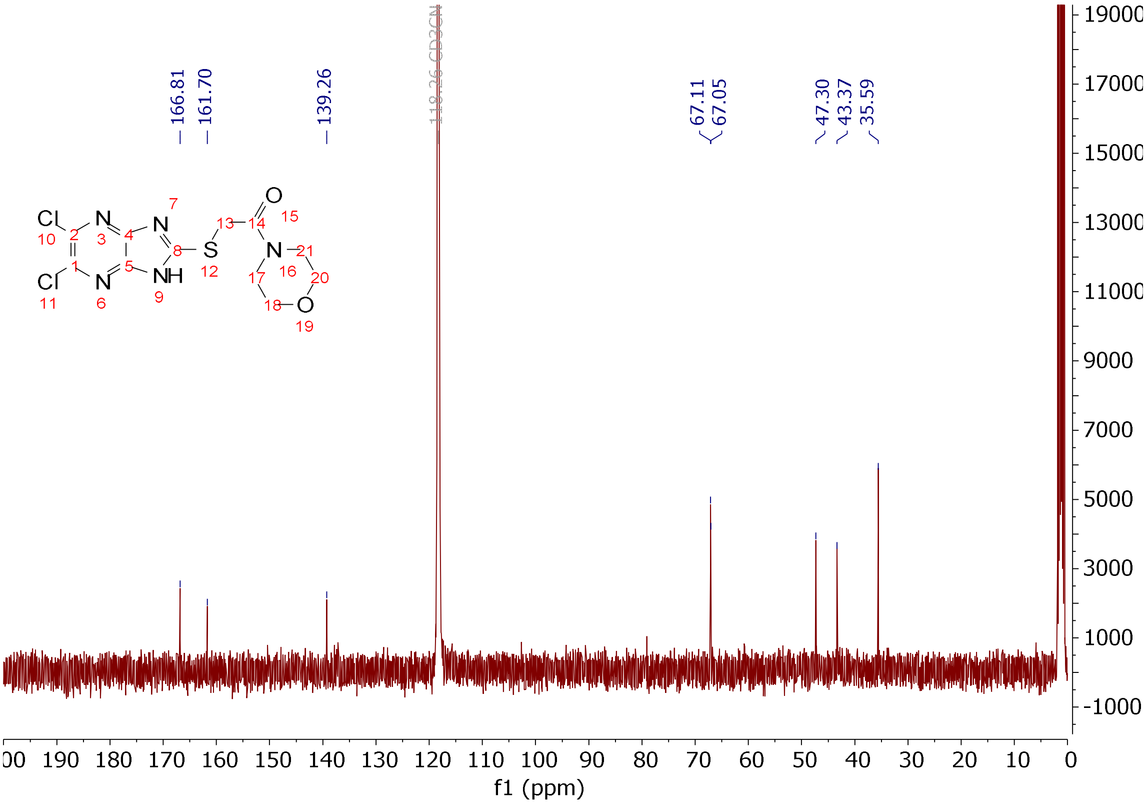

HRMS data:

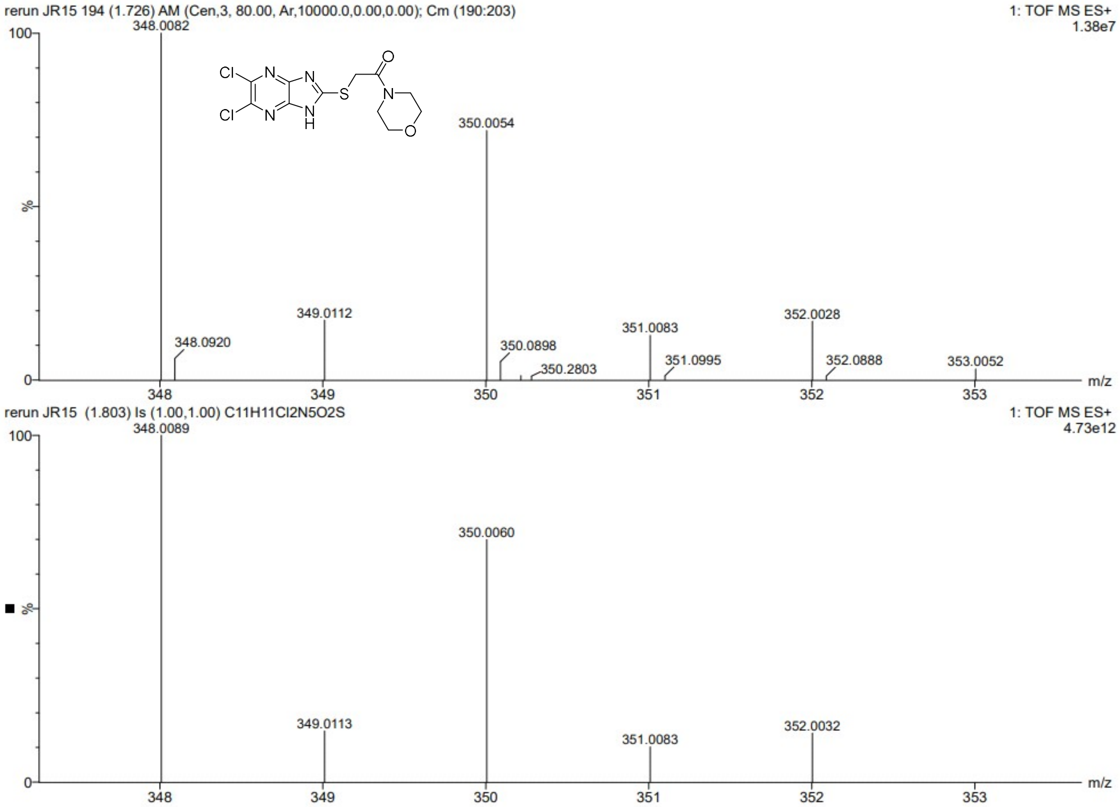
rerun JR15 194 (1.726) AM (Cen,3, 80.00, Ar,10000.0,0.00,0.00); Cm (190:203)
1: TOF MS ES+
1.38e7
100
348.0082
Cl
Cl
N
N
N
H
S
O
N
O
350.0054
%
349.0112
348.0920
350.0898
350.2803
351.0083
351.0995
352.0028
352.0888
353.0052
0
348
349
350
351
352
353
m/z
rerun JR15 (1.803) Is (1.00,1.00) C11H11Cl2N5O2S
1: TOF MS ES+
4.73e12
100
348.0089
350.0060
%
349.0113
351.0083
352.0032
0
348
349
350
351
352
353
m/z

***Compound 10** – 2-({5,6-dichloro-1H-imidazo[4,5-b]pyrazine-2-yl}sulfanyl)pentan-2-one*

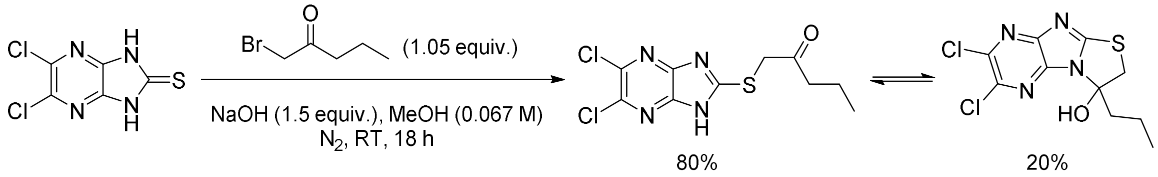

To an 8 mL vial was added 5,6-dichloroimidazo[4,5-*b*]pyrazine-2(1,3*H*)-thione (100 mg, 0.452 mmol) and sodium hydroxide (28 mg, 1.5 equiv.). The vial was then sealed with a suba seal and wrapped with parafilm. Methanol (6.75 mL, 0.067 M) was added *via* syringe and the mixture stirred until all solids had dissolved. 1-bromopentan-2-one (70 µL, 1.05 equiv.) was added *via* microsyringe and the reaction was left to stir for 18 h. The solvent was removed under reduced pressure and the solid redissolved in NH_4Cl solution and EtOAc (*ca.* 5 mL each) and the mixture transferred to a separatory funnel. The organic layer was removed, and the aqueous layer extracted with EtOAc (3x 10 mL). The organic layers were collected and dried ($MgSO_4$) and solvent removed *in vacuo.* The crude material was purified by column chromatography eluting with Pet. E. : EtOAc (8:2) to provide 2-({5,6-dichloro-1*H*-imidazo[4,5-*b*]pyrazine-2-yl}sulfanyl)pentan-2-one as a white solid (74.7 mg, 0.245 mmol, 54%). Consistent with the previous report of imidazo[4,5-b]pyrazine-2-thioacetoketone compounds, This compound is in equilibrium with its cyclic hemiaminol, as observed by ^{1}H NMR and IR.[162] The ratio of free ketone to tricyclic alcohol is approx. 8:2 (molar) by ^{1}H NMR.

Free ketone: ^{1}H NMR (400 MHz, CD_3CN) δ 11.11 (br s, 1H), 4.32 (s, 2H), 2.65 (t, J = 7.3 Hz, 2H), 1.62 (p, J = 7.3 Hz, 2H), 0.92 (t, J = 7.3 Hz, 3H).

^{13}C NMR (101 MHz, CD_3CN) δ 204.2, 161.3, 139.3, 44.0, 42.1, 17.9, 13.8 (7 out of 8

carbon resonances observed).

Hemiaminol: ^{1}H NMR (400 MHz, C*D*$_3$CN) δ 4.12 (d, *J* = 12.3 Hz, 1H), 3.75 (d, *J* = 12.3 Hz, 1H), 2.62 – 2.55 (m, 2H), 2.33 (ddd, *J* = 13.9, 12.0, 4.6 Hz, 2H), 1.00 (t, *J* = 7.4 Hz, 3H).

^{13}C NMR (101 MHz, C*D*$_3$CN) δ 45.9, 40.6, 18.0, 13.8.

IR (cm^{-1}) 3249 (OH), 1743 (C=O), 1584 (NH bend).

M.P. 151.2 – 151.9 °C.

MS ES+ m/z calcd for $C_{10}H_{10}Cl_2N_4OS$ $(M+H)^+$: 305.0031, found: 305.0032.

Adapted from literature procedure[5].

NMR data:

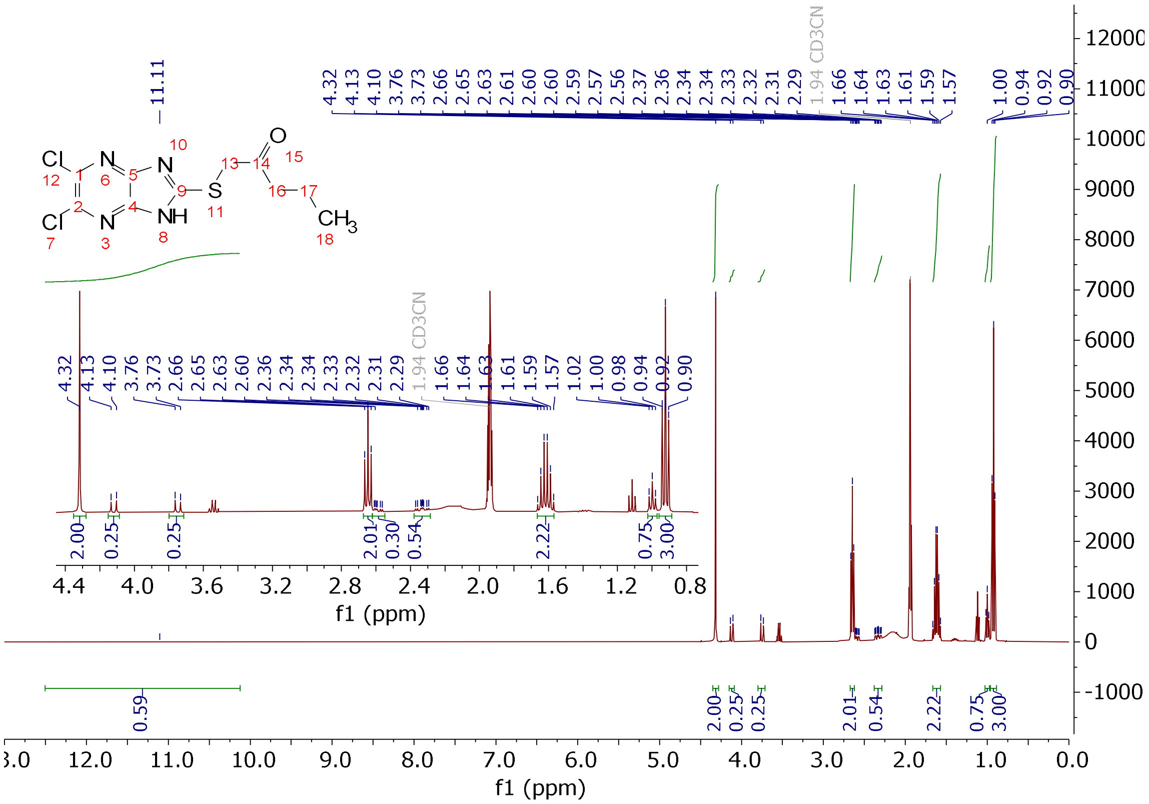

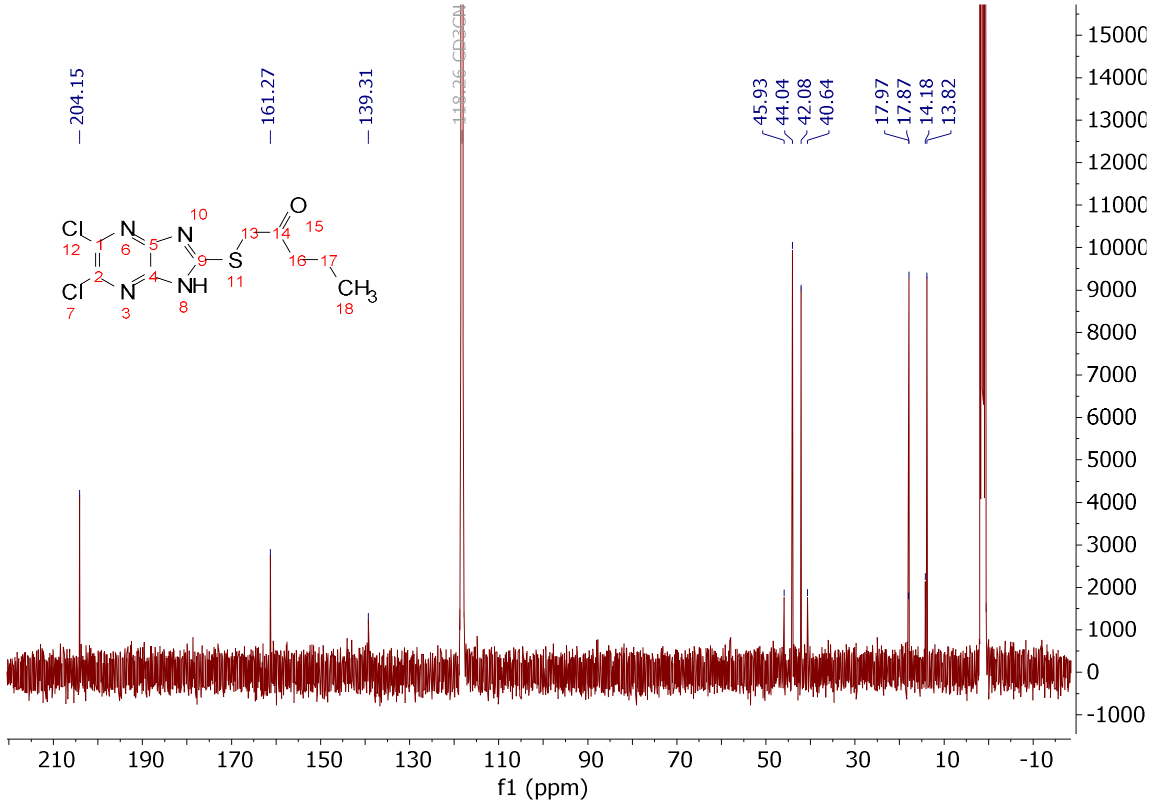
204.15
161.27
139.31
118.26 CD3CN
45.93
44.04
42.08
40.64
17.97
17.87
14.18
13.82
f1 (ppm)

HRMS data:

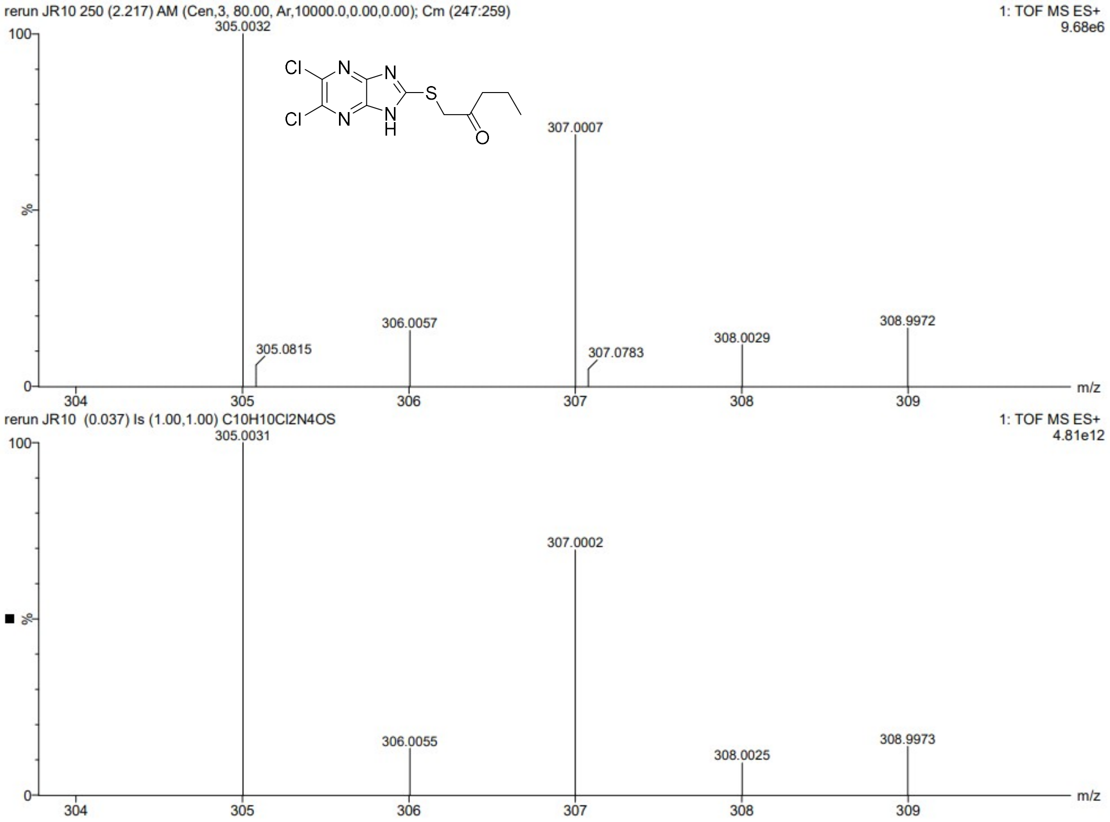
rerun JR10 250 (2.217) AM (Cen,3, 80.00, Ar,10000.0,0.00,0.00); Cm (247:259)
1: TOF MS ES+
9.68e6
305.0032
307.0007
306.0057
305.0815
307.0783
308.0029
308.9972
m/z
rerun JR10 (0.037) Is (1.00,1.00) C10H10Cl2N4OS
1: TOF MS ES+
4.81e12
305.0031
307.0002
306.0055
308.0025
308.9973
m/z

Compound 11 *– Ethyl 2-({5,6-dichloro-1H-imidazo[4,5-b]pyrazine-2-yl}sulfanyl)butyrate*

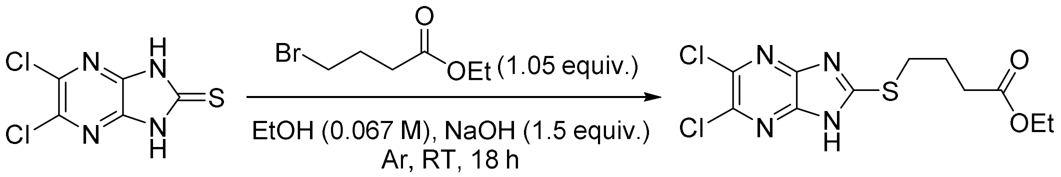

To a 25 mL RBF was added a stirrer bar, 5,6-dichloroimidazo[4,5-*b*]pyrazine-2(1,3*H*)-thione (100 mg, 0.452 mmol) and sodium hydroxide (27.4 mg, 1.5 equiv.) and placed under argon atmosphere. Ethanol (over mol. sieves, 6.75 mL) was added with stirring. Ethyl 4-bromobutyrate (67.9 µL, 1.05 equiv.) was added via syringe with stirring. Allowed to stir at RT for 18 h. The solvent was removed under reduced pressure and the solid redissolved in sat. soln. NH_4Cl (10 mL) and EtOAc (10 mL). Transferred to a separatory funnel and the organic layer separated. The aqueous layer was extracted with EtOAc (3x 10 mL), the organic layers were collected and dried ($MgSO_4$). EtOAc was removed *in vacuo.* to provide a beige solid which was purified by column chromatography, eluting with 8:2 Pet. E : EtOAc to provide a colourless oil which solidifies over time into a light-orange solid. Further purified by triturating with *n*-hexane (5 mL) and ice-cold ethanol (5 mL) to provide a white solid of ethyl 2-({5,6-dichloro-1*H*-imidazo[4,5-*b*]pyrazine-2-yl}sulfanyl)butyrate (70.0 mg, 0.20 mmol, 46%).

^{1}H NMR (400 MHz, CD_3CN) δ 11.19 (br s, 1H), 4.11 (q, *J* = 7.2 Hz, 1H), 3.39 (t, *J* = 7.3 Hz, 1H), 2.47 (t, *J* = 7.3 Hz, 1H), 2.09 (p, *J* = 7.3 Hz, 1H), 1.21 (t, *J* = 7.2 Hz, 1H).

^{13}C NMR (101 MHz, CD_3CN) *δ* 173.7, 162.1, 139.1, 61.3, 33.3, 31.3, 25.7, 14.5 (8 out of 9 carbon resonances observed).

IR (cm^{-1}): 3151 (NH), 1691 (C=O).

M.P. 120.3 – 121.3 °C.

MS ES+ m/z calcd for $C_{11}H_{12}Cl_2N_4O_2S$ $(M+H)^+$: 335.0136, found: 335.0135.

Adapted from literature procedure[5].

NMR data:

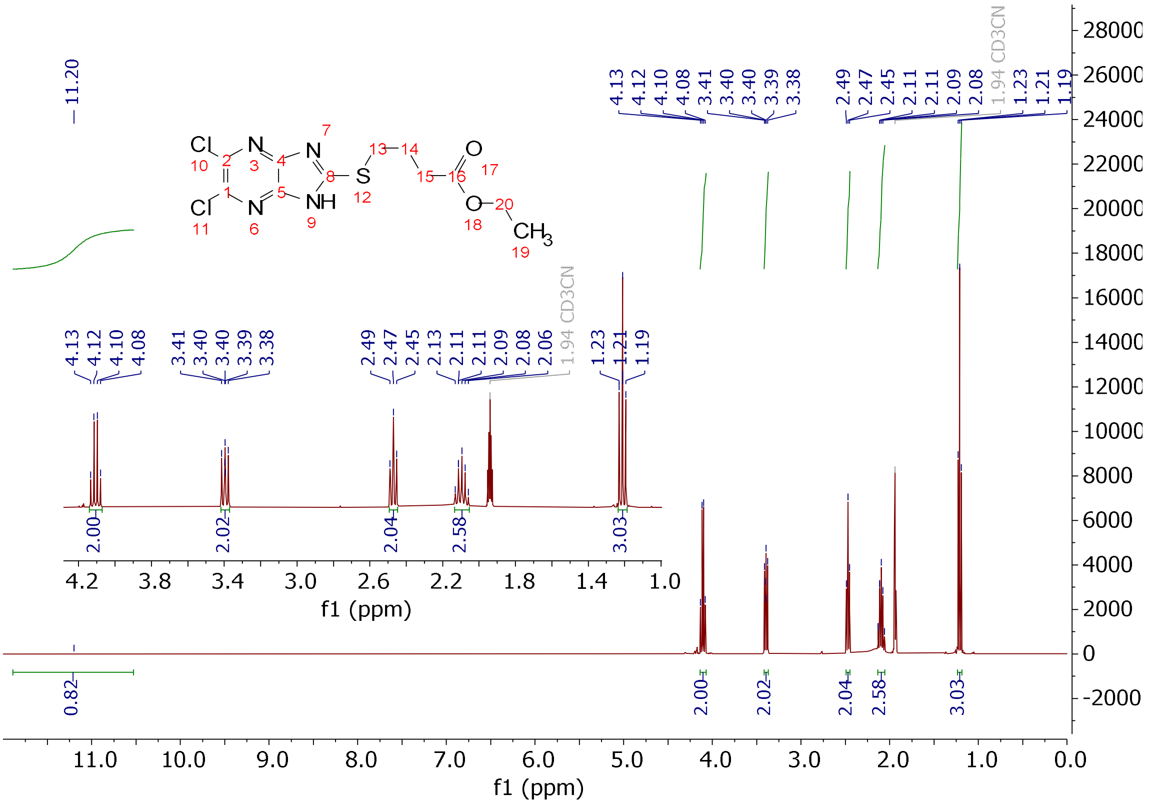

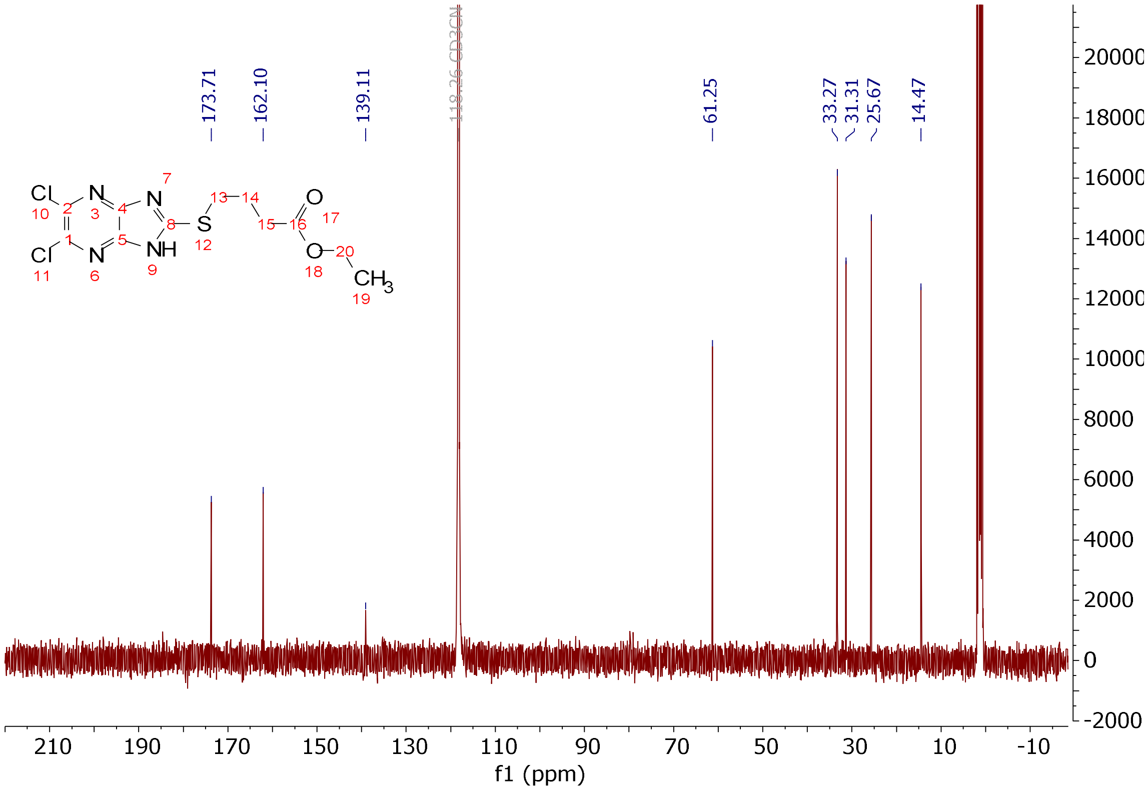
173.71
162.10
139.11
118.26 CD3CN
61.25
33.27
31.31
25.67
14.47
f1 (ppm)

HRMS data:

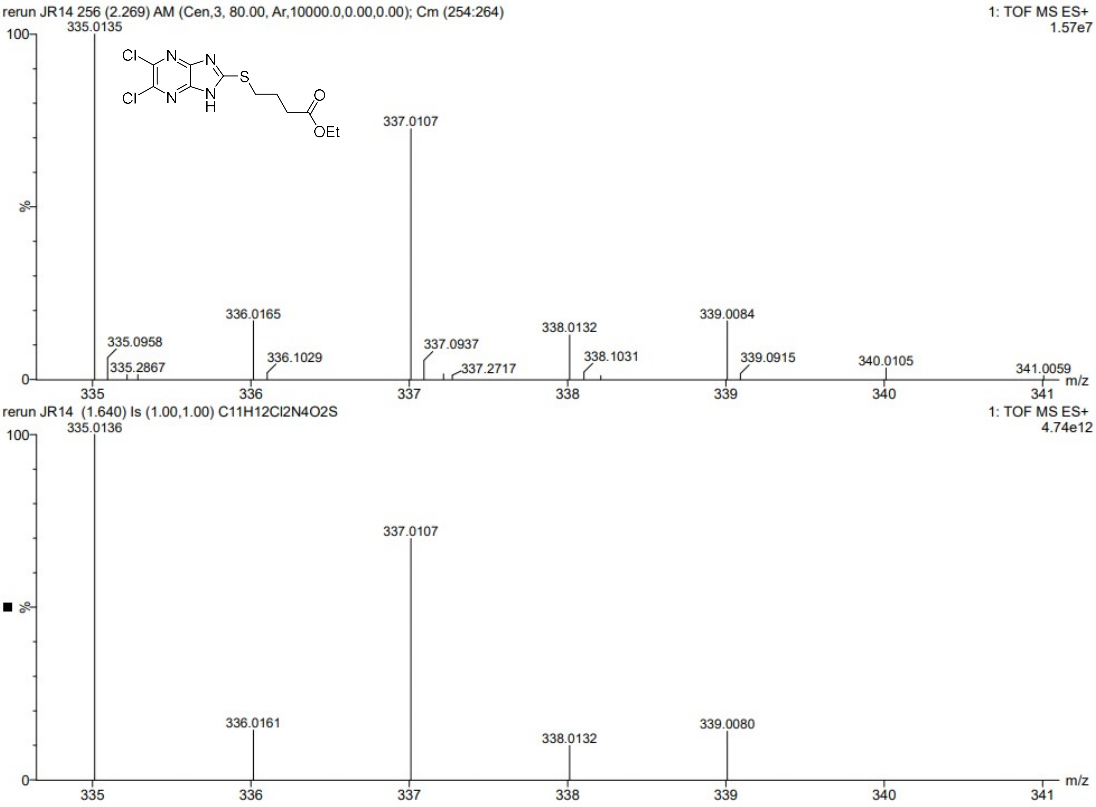
rerun JR14 256 (2.269) AM (Cen,3, 80.00, Ar,10000.0,0.00,0.00); Cm (254:264)
1: TOF MS ES+
1.57e7
335.0135
337.0107
336.0165
339.0084
338.0132
rerun JR14 (1.640) Is (1.00,1.00) C11H12Cl2N4O2S
1: TOF MS ES+
4.74e12
335.0136
337.0107
336.0161
338.0132
339.0080
m/z

Compound 12 – *Ethyl 2-({5,6-dichloro-1H-imidazo[4,5-b]pyrazine-2-yl}sulfanyl)acetamidoacetate*

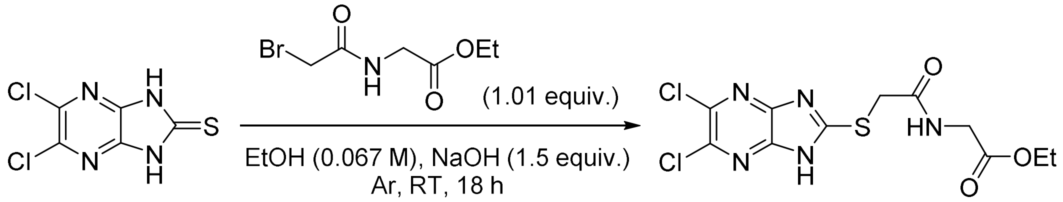

To a 25 mL RBF was added a stirrer bar, 5,6-dichlorimidazo[4,5-*b*]pyrazine-2(1,3*H*)-thione (150 mg, 0.678 mmol), sodium hydroxide (40.7 mg, 1.5 equiv.) and placed under argon atmosphere. Ethanol (over mol. sieves, 5.0 mL) was added with stirring to dissolve starting material. Ethanol (over mol. sieves, 5.0 mL) was added to a separate vial containing ethyl bromo-acetamidoacetate (0.154 g, 1.01 equiv.) and stirred to dissolve. This solution was then transferred to the 25 mL RBF *via* syringe and left to stir at RT for 18 h. The solvent was removed *in vacuo.* and the residue redissolved in sat. soln. NH_4Cl (10 mL), and EtOAc (10 mL), transferred to a separatory funnel and the organic layer removed. Aqueous layer extracted with EtOAc (3x 10 mL), organic layers collected and dried ($MgSO_4$) and solvent removed *in vacuo.* and residue purified by column chromatography (6:4 Pet. E : EtOAc -> EtOAc) to provide a white fluffy powder of ethyl 2-({5,6-dichloro-1*H*-imidazo[4,5-*b*]pyrazine-2-yl}sulfanyl)acetamidoacetate (133 mg, 0.36 mmol, 54%).

^{1}H NMR (400 MHz, CD_3CN) δ 7.41 (br s, 1H), 4.12 (q, J = 7.1 Hz, 2H), 4.07 (s, 2H), 3.92 (d, J = 5.8 Hz, 2H), 1.19 (t, J = 7.1 Hz, 3H).

^{13}C NMR (101 MHz, CD_3CN) δ 169.9, 168.7, 160.8, 139.2, 61.6, 42.0, 34.8, 14.0. (8 out of 9 carbon resonances observed).

IR (cm^{-1}) 3292 (NH), 3219 (NH), 1717 (C=O ester), 1648 (C=O amide).

M.P. 187.7 – 188.3 °C.

MS ES+ m/z calcd. for $C_{11}H_{11}Cl_2N_5O_3S$ $(M+H)^+$: 365.0062, found: 365.0063.

Adapted from literature procedure[5].

NMR data:

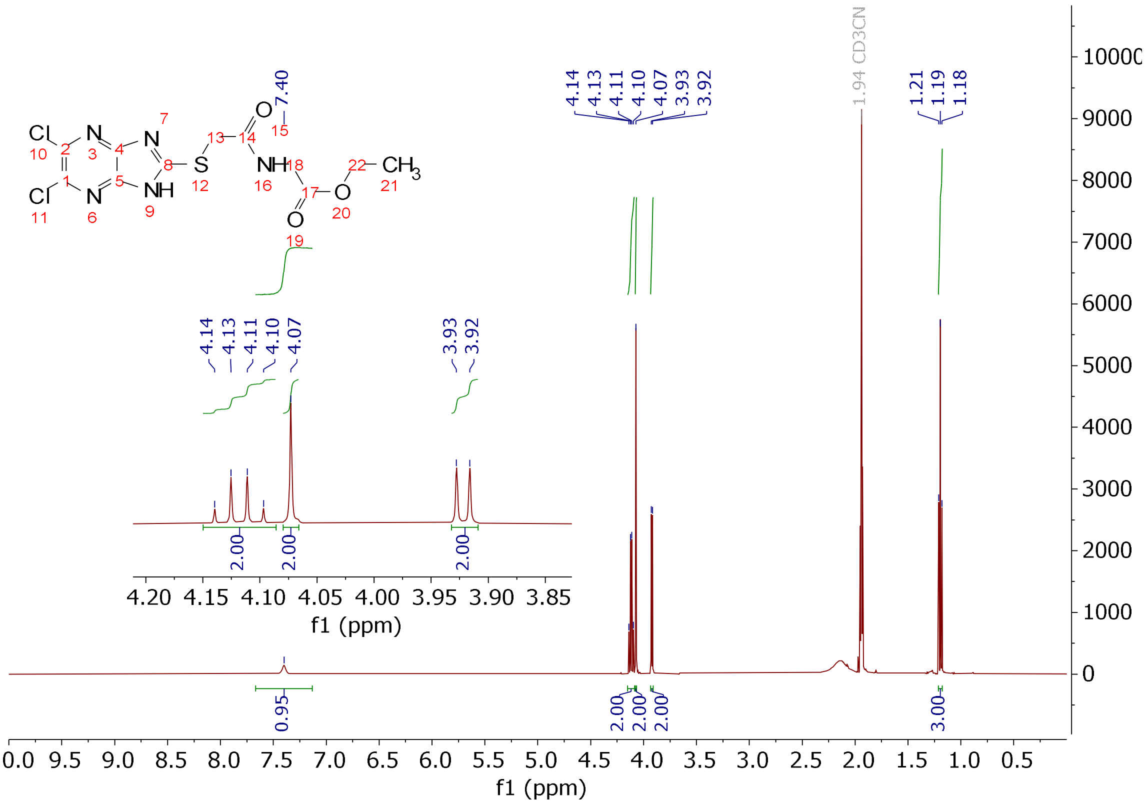

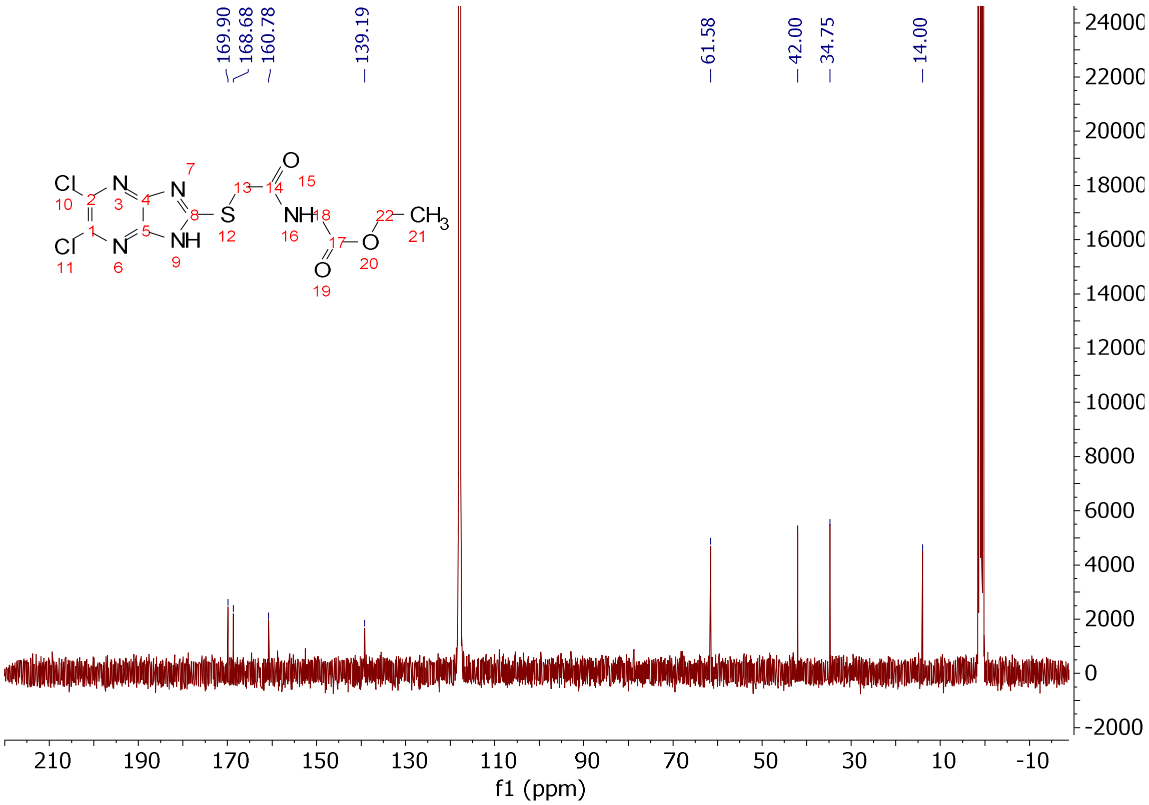
169.90
168.68
160.78
139.19
61.58
42.00
34.75
14.00
f1 (ppm)

HRMS data:

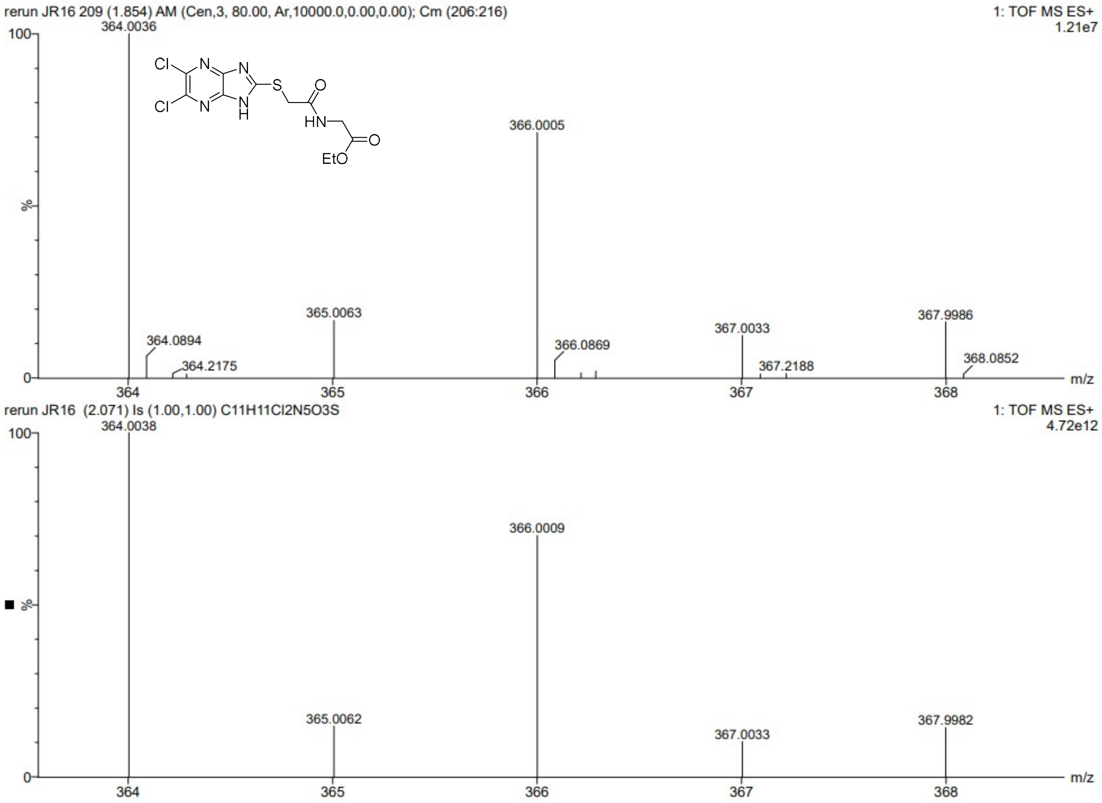
rerun JR16 209 (1.854) AM (Cen,3, 80.00, Ar,10000.0,0.00,0.00); Cm (206:216)
1: TOF MS ES+
1.21e7
364.0036
366.0005
365.0063
364.0894
364.2175
366.0869
367.0033
367.2188
367.9986
368.0852
m/z
rerun JR16 (2.071) Is (1.00,1.00) C11H11Cl2N5O3S
1: TOF MS ES+
4.72e12
364.0038
366.0009
365.0062
367.0033
367.9982
m/z

Compound 13 – *3-({5,6-Dichloro-1H-imidazo[4,5-b]pyrazine-2-yl}sulfanyl)propionic acid*

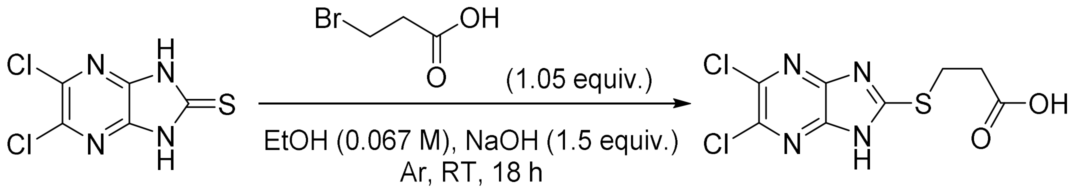

To an 8 mL vial was added a stirrer bar, 5,6-dichloroimidaazo[4,5-*b*]pyrazine-2(1,3*H*)-thione (75 mg, 0.34 mmol), sodium hydroxide (20. 3 mg, 1.5 equiv.) and ethanol (over mol. sieves, 5.0 mL), stirred to dissolve. 3-bromopropionic acid (54.5 mg, 1.05 equiv.) was added and the vial placed under argon, left to stir for 18 h at RT. The solvent was removed *in vacuo.* and the residue dissolved in water (5 mL), extracted twice with diethyl ether (2x 10 mL), the aqueous layer was then acidified to pH approx. 2 and re-extracted with EtOAc (3x 10 mL), the EtOAc layers were then dried ($MgSO_4$) and solvent removed *in vacuo.* Further purified by column chromatography, eluting with Pet. E : EtOAc (6:4 with 0.4% acetic acid) to provide 2-({5,6-dichloro-1*H*-imidazo[4,5-b]pyrazine-2-yl}sulfanyl)propionic acid (23.8 mg, 0.08 mmol, 24%) as a yellow solid.

^{1}H NMR (400 MHz, CD_3CN) δ 3.55 (t, J = 6.8 Hz, 2H), 2.87 (t, J = 6.8 Hz, 2H).

^{13}C NMR (101 MHz, CD_3CN) δ 173.0, 161.9, 139.2, 34.4, 27.4 (5 out of 6 carbon resonances observed).

IR (cm^{-1}): 3111 (NH), 3023 (OH), 1721 (C=O).

M.P. 205.1 – 206.2 °C (deg.).

MS ES+ m/z calcd for $C_8H_6Cl_2N_4O_2S$ $(M+H)^+$: 293.9689, found: 293.9688.

Adapted from literature procedure[5].

NMR data:

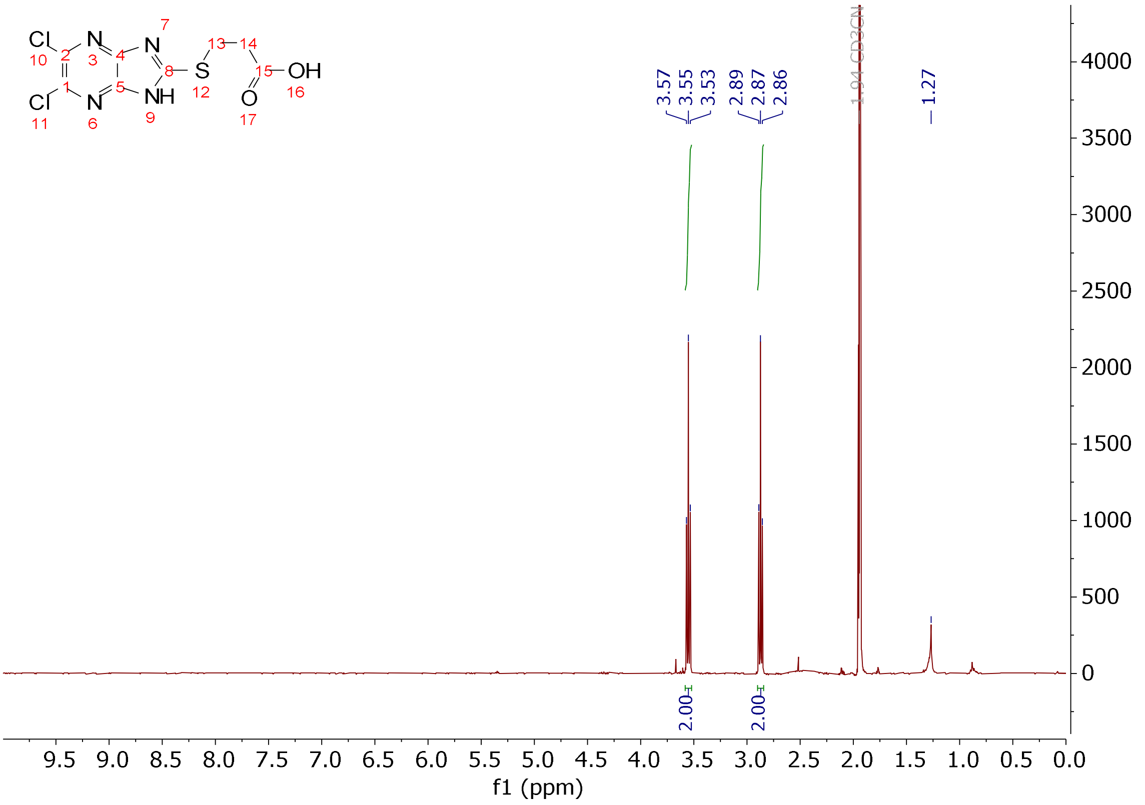

3.57
3.55
3.53
2.89
2.87
2.86
1.94 CD3CN
1.27
2.00
2.00
f1 (ppm)

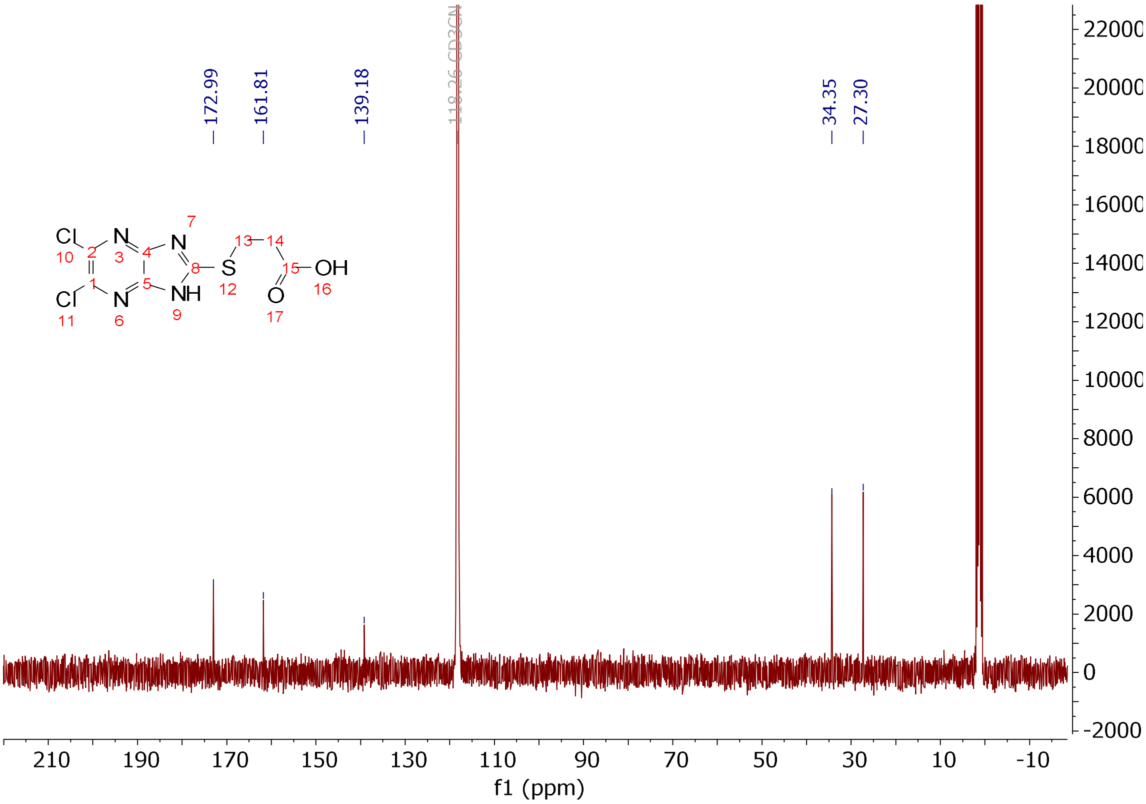

172.99
161.81
139.18
118.26 CD3CN
34.35
27.30
f1 (ppm)

HRMS data:

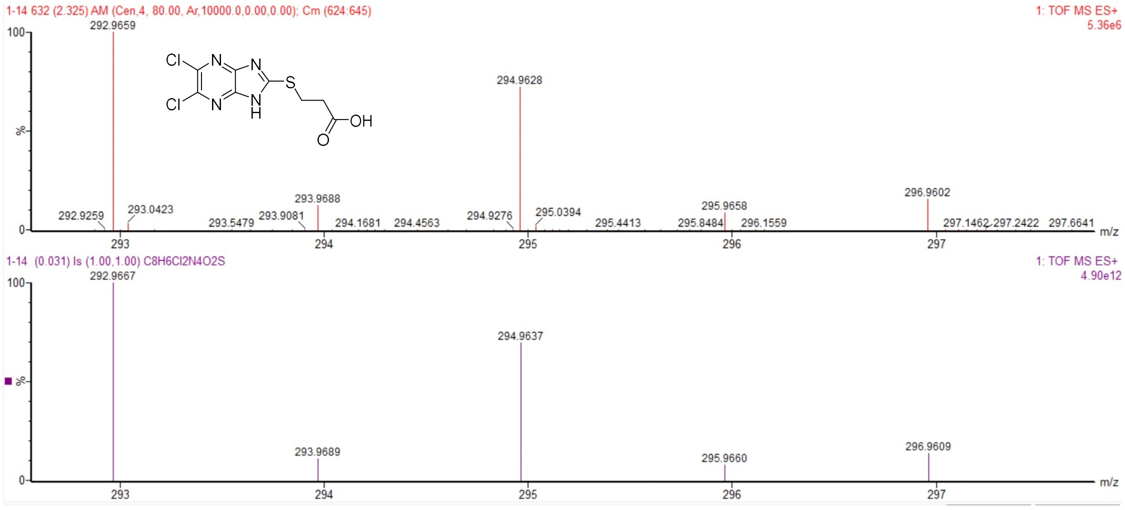

1-14 632 (2.325) AM (Cen,4, 80.00, Ar,10000.0,0.00,0.00); Cm (624:645)
1: TOF MS ES+
5.36e6
292.9659
294.9628
293.9688
295.9658
296.9602
292.9259
293.0423
293.5479
293.9081
294.1681
294.4563
294.9276
295.0394
295.4413
295.8484
296.1559
297.1462
297.2422
297.6641
m/z
1-14 (0.031) Is (1.00,1.00) C8H6Cl2N4O2S
1: TOF MS ES+
4.90e12
292.9667
294.9637
293.9689
295.9660
296.9609

Compound 14 *– Methyl 3-(tert-butoxy)-2(S)-[2-({5,6-dichloro-1H-imidazo[4,5-b]pyrazine-2-yl}sulfanyl)acetamido]propionate*

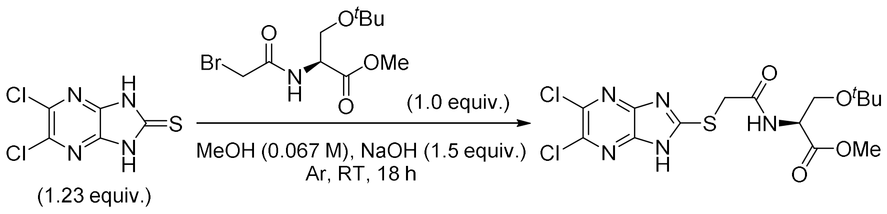

To a 20 mL vial was added 5,6-dichloroimidazo[4,5-*b*]pyrazine-2(1,3*H*)-thione (150 mg, 0.678 mmol, 1.23 equiv.), sodium hydroxide (40 mg, 1.5 equiv.) and placed under argon atmosphere. To a separate vial was added (+)-(2*S*)-methyl 2-(2-bromoacetylamino)-3-(*tert*-butoxy)propanoate (167 mg, 0.55 mmol) and this was dissolved with methanol (5 mL). This solution was transferred *via* syringe to the reaction vial and stirred at RT for 18 h. The solvent is removed *in vacuo.* and the residue taken up in ammonium chloride and EtOAc (5 mL each). The layers were separated and the aqueous extracted with EtOAc (3x 10 mL). The organic layers were collected and dried ($MgSO_4$) and solvent removed under reduced pressure. The crude material was purified by column chromatography (6:4 -> 5:5, Pet E. : EtOAc) to provide methyl 3-(*tert*-butoxy)-2(*S*)-[2-({5,6-dichloro-1*H*-imidazo[4,5-*b*]pyrazine-2-yl}sulfanyl)acetamido]propionate as an off-white solid (173 mg, 0.397 mmol, 59%).

^{1}H NMR (400 MHz, Acetone-d_6) δ 12.93 (br s, 1H), 8.06 (br d, J = 8.5 Hz, 1H), 4.63 (dt, J = 8.5, 3.5 Hz, 1H), 4.24 (s, 2H), 3.80 (dd, J = 9.2, 3.5 Hz, 1H), 3.66 (s, 3H), 3.59 (dd, J = 9.2, 3.5 Hz, 1H), 1.08 (s, 9H).

^{13}C NMR (101 MHz, Acetone-d_6) δ 171.0, 168.3, 161.7, 139.2, 73.8, 62.6, 54.4, 52.4, 35.1, 27.5 (10 out of 11 carbon resonances observed).

IR (cm^{-1}) 3272 (NH), 1756 (C=O ester), 1719 (C=O amide).

M.P. 145.7 – 146.2 ºC.

MS ES+ m/z calcd for $C_{15}H_{19}Cl_2N_5O_4S$ $(M+H)^+$: 436.0613, found: 436.0623.

$[\alpha]^{25}_{589}$ = 115.38 (EtOAc, 0.026 M, l = 1 cm, α = 0.03).

Adapted from literature procedure[5].

NMR data:

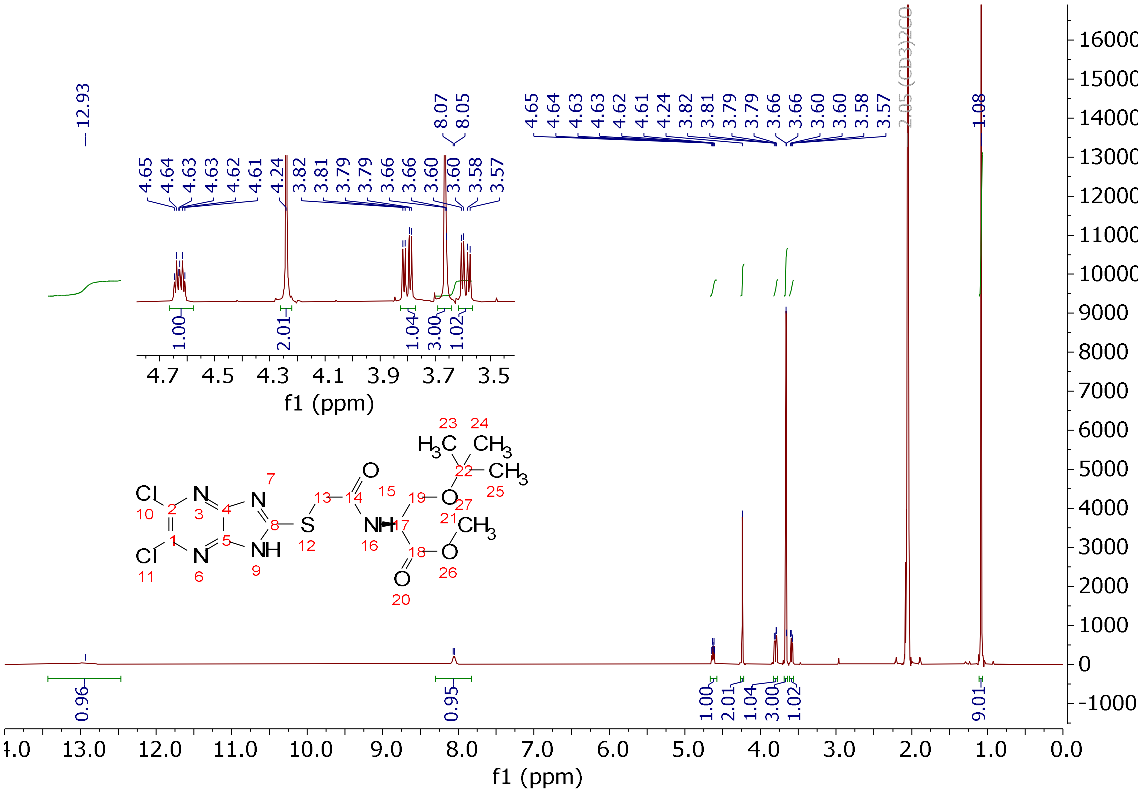

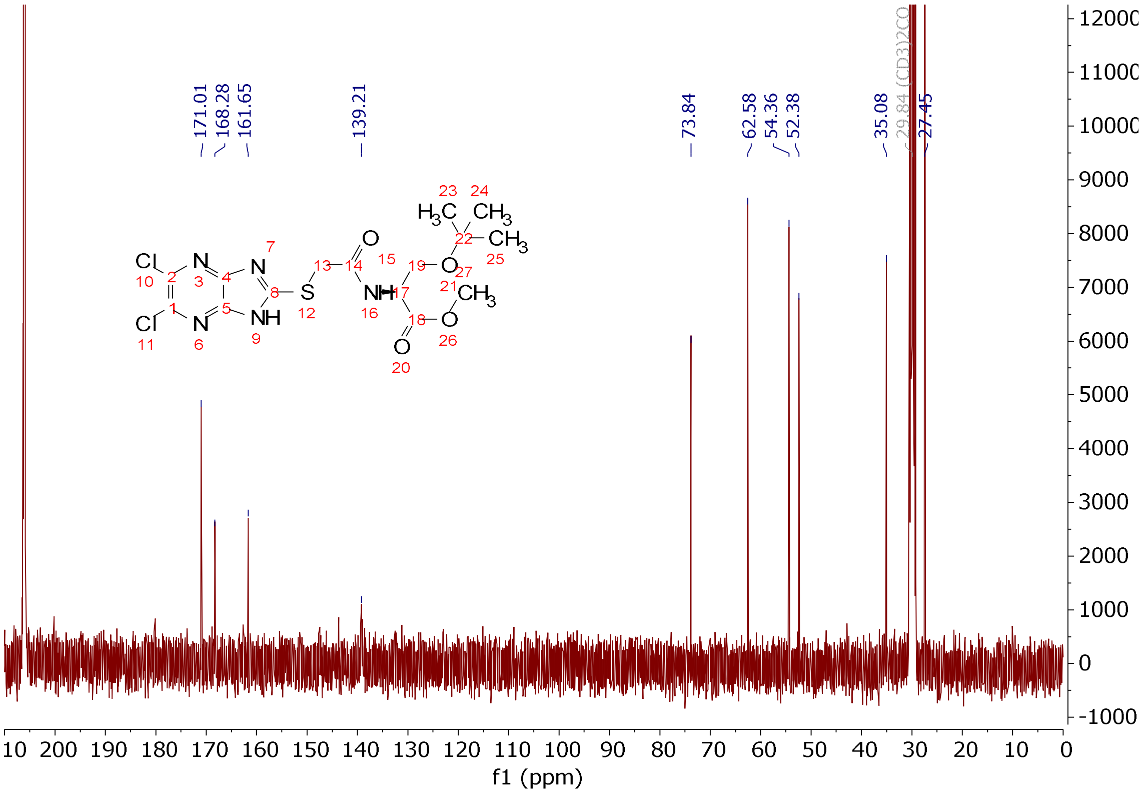
171.01
168.28
161.65
139.21
73.84
62.58
54.36
52.38
35.08
29.84 (CD3)2CO
27.45
f1 (ppm)

HRMS data:

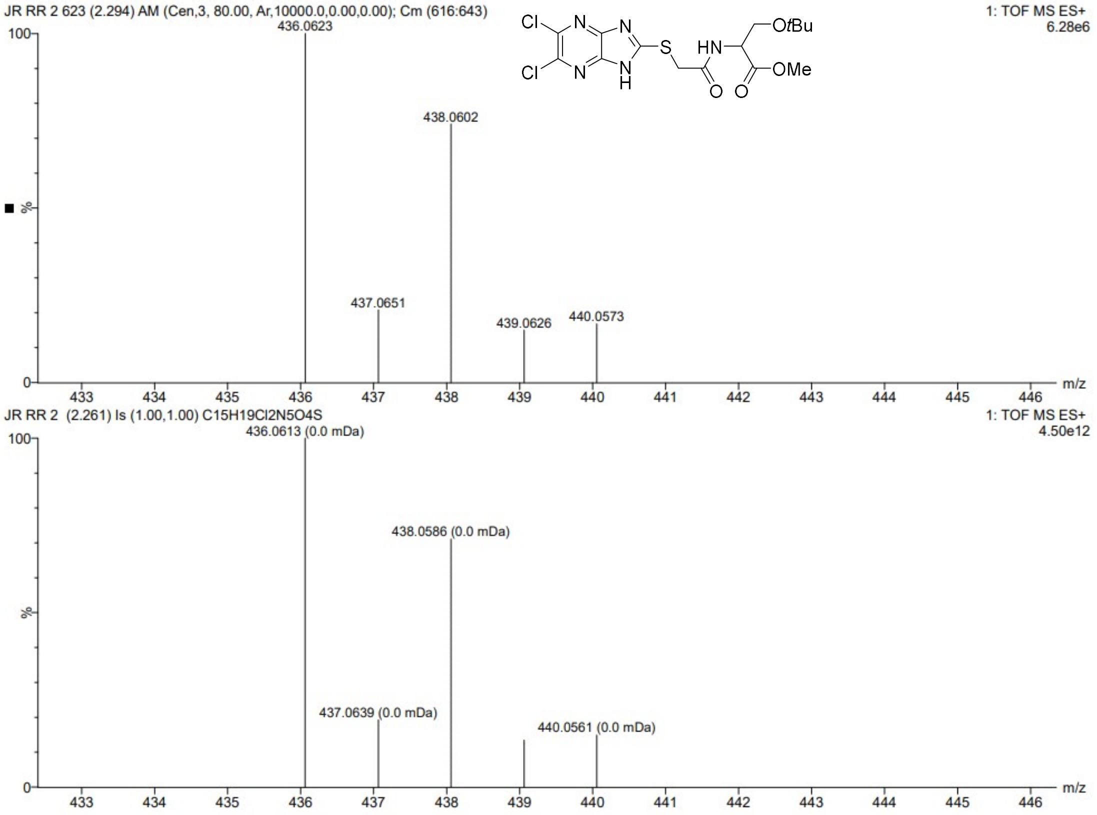
JR RR 2 623 (2.294) AM (Cen,3, 80.00, Ar,10000.0,0.00,0.00); Cm (616:643)
1: TOF MS ES+
6.28e6
436.0623
438.0602
437.0651
439.0626
440.0573
OtBu
OMe
m/z
JR RR 2 (2.261) Is (1.00,1.00) C15H19Cl2N5O4S
1: TOF MS ES+
4.50e12
436.0613 (0.0 mDa)
438.0586 (0.0 mDa)
437.0639 (0.0 mDa)
440.0561 (0.0 mDa)
m/z

Compound 15 *– ({5,6-dichloro-1H-imidazo[4,5-b]pyrazine-2-yl}sulfanyl)acetic acid*

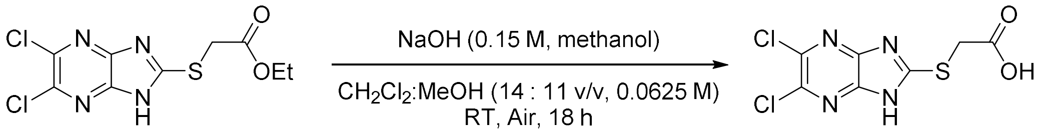

Followed general procedure 1. Ethyl 2-({5,6-dichloro-1*H*-imidazo[4,5-*b*]pyrazine-2-yl}sulfanyl)-acetate (50 mg, 0.162 mmol), DCM : MeOH solution (1.62 mL). NaOH (0.97 mL). Provided ({5,6-dichloro-1*H*-imidazo[4,5-*b*]pyrazine-2-yl}sulfanyl)acetic acid as a light-yellow solid (31.4 mg, 0.11 mmol, 69%).

^{1}H NMR (400 MHz, C*D*$_3$CN) *δ* 4.14 (s, 2H).

^{13}C NMR (101 MHz, C*D*$_3$CN) *δ* 169.6, 161.2, 144.8, 139.5, 34.2.

IR (cm^{-1}) 3111 (NH), 3027 (OH), 1740 (C=O).

M.P. 219.1 – 220.7 ºC.

MS ES+ m/z calcd for $C_7H_4Cl_2N_4O_2S$ $(M+H)^+$: 278.9510, found (-1.979 average neutral loss): 276.9719.

Adapted from literature procedure[21].

NMR data:

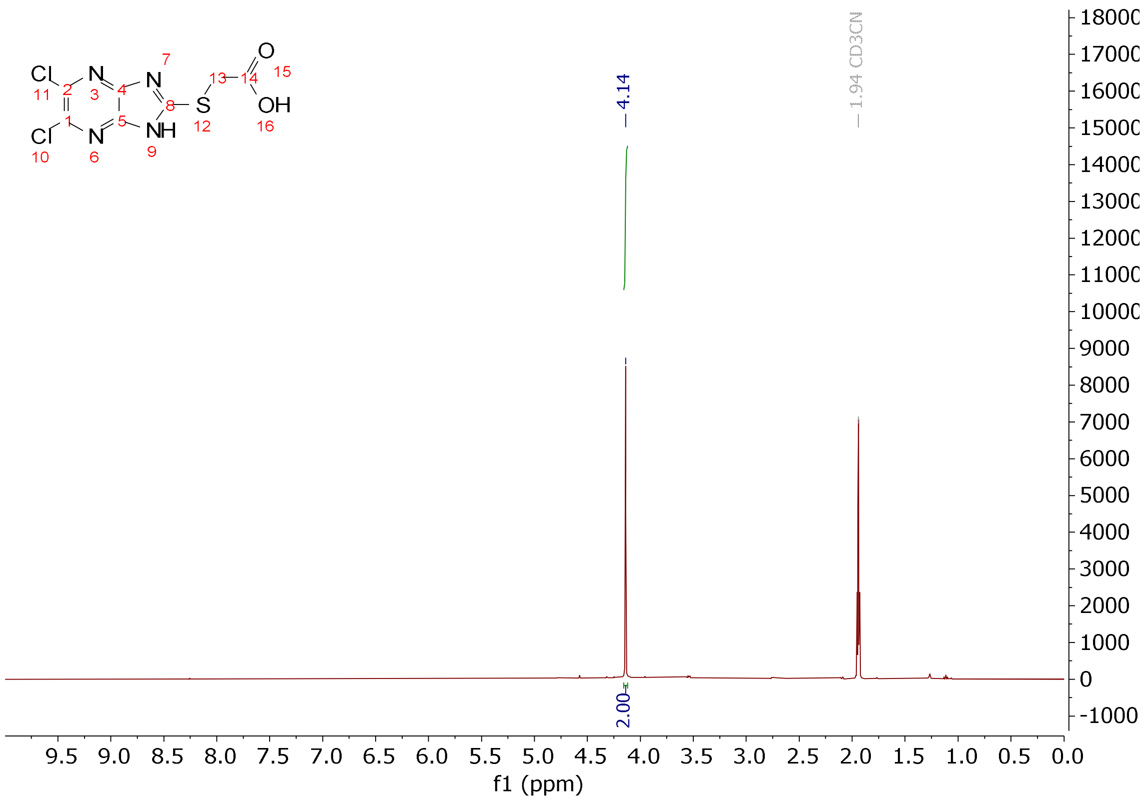

Cl
N
N
S
O
OH
Cl
N
NH
11 2 3 4 7 13 14 15 8 12 16
10 1 6 5 9
4.14
1.94 CD3CN
2.00
f1 (ppm)

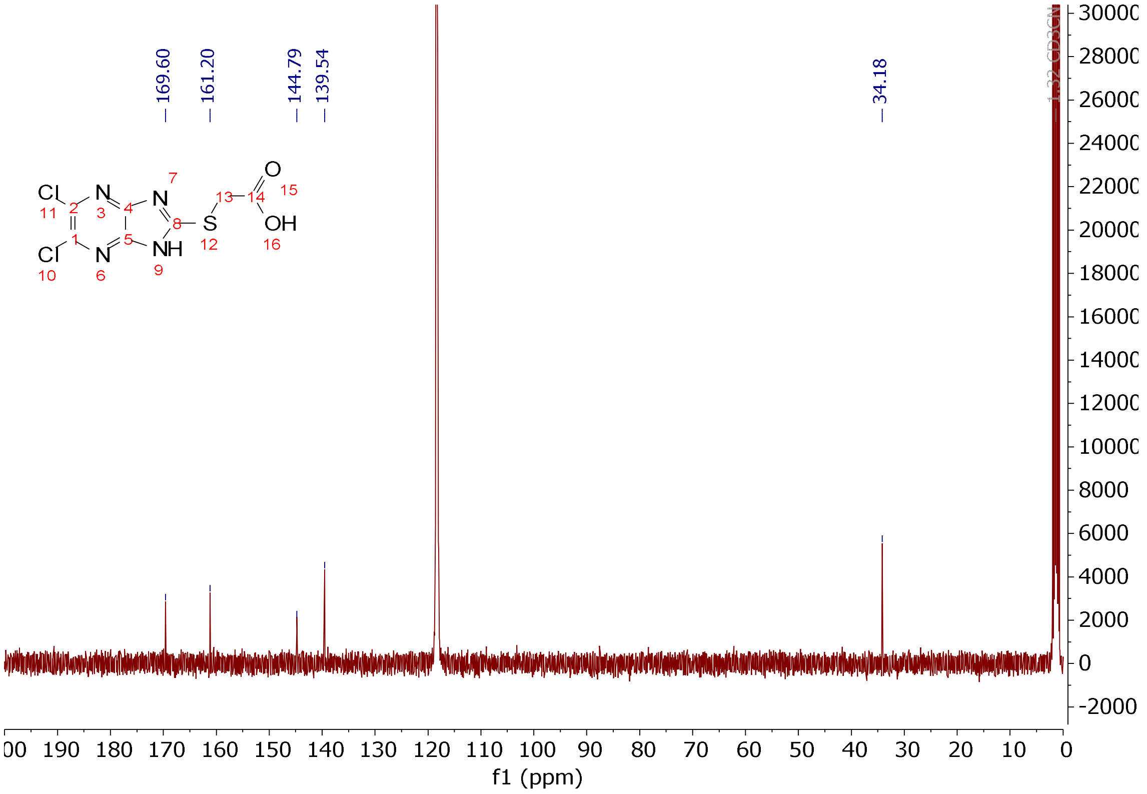

169.60
161.20
144.79
139.54
34.18
Cl
N
N
S
O
OH
Cl
N
NH
11 2 3 4 7 13 14 15 8 12 16
10 1 6 5 9
f1 (ppm)

HRMS data:

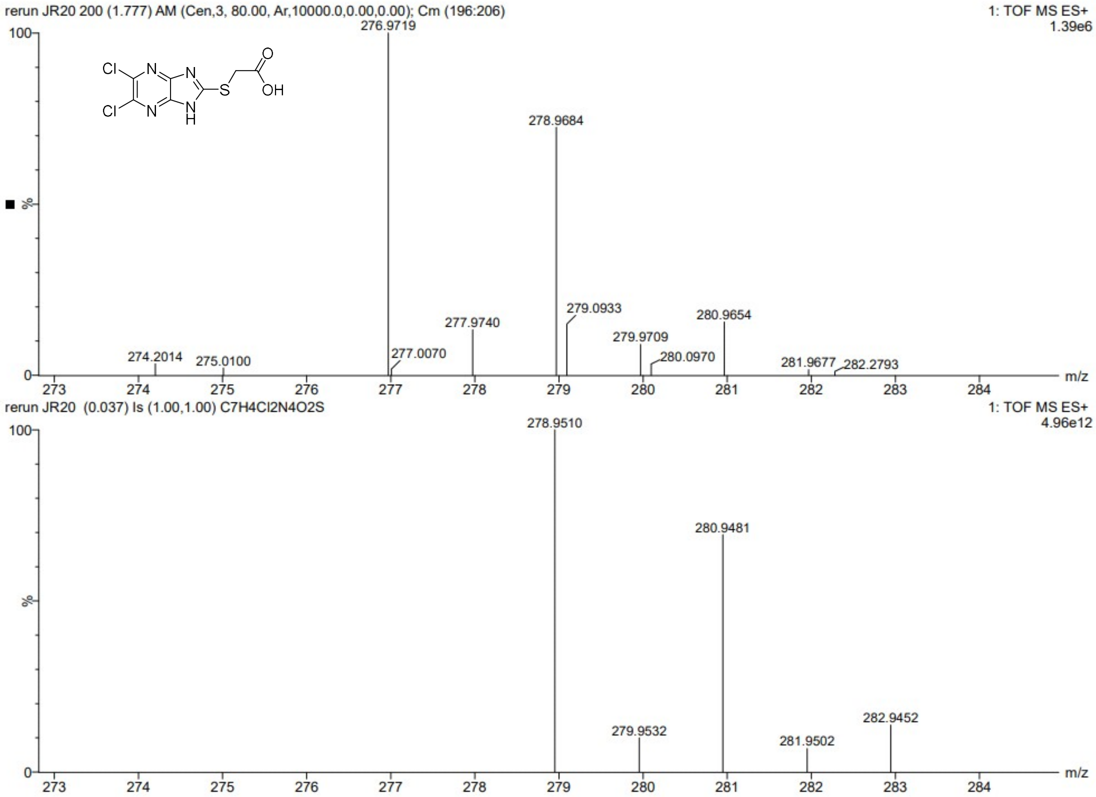
rerun JR20 200 (1.777) AM (Cen,3, 80.00, Ar,10000.0,0.00,0.00); Cm (196:206)
1: TOF MS ES+
1.39e6
Cl
Cl
N
N
N
H
S
O
OH
276.9719
278.9684
277.9740
279.0933
279.9709
280.9654
280.0970
281.9677
282.2793
274.2014
275.0100
277.0070
%
m/z
rerun JR20 (0.037) Is (1.00,1.00) C7H4Cl2N4O2S
1: TOF MS ES+
4.96e12
278.9510
280.9481
279.9532
281.9502
282.9452
%
m/z

Compound 16 – *2-({5,6-dichloro-1H-imidazo[4,5-b]pyrazine-2-yl}sulfanyl)acetamidoacetic acid*

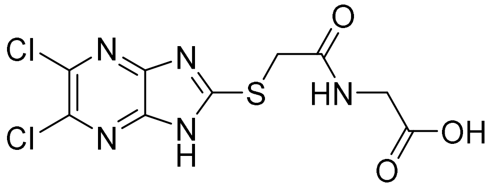

Followed general procedure 1. Ethyl 2-({5,6-dichloro-1*H*-imidazo[4,5-*b*]pyrazine-2-yl}sulfanyl)acetamidoacetate (50 mg, 0.137 mmol), DCM : MeOH solution (1.37 mL). NaOH (0.82 mL). Provided 2-({5,6-dichloro-1*H*-imidazo[4,5-*b*]pyrazine-2-yl}sulfanyl)acetamidoacetic acid as a light-yellow solid (26.0 mg, 0.077 mmol, 56%).

^{1}H NMR (400 MHz, DMSO-d_6) δ 14.11 (br s, 1H), 12.66 (br s, 1H), 8.65 (br t, J = 5.8 Hz, 1H), 4.23 (s, 2H), 3.81 (d, J = 5.8 Hz, 2H).

^{13}C NMR (101 MHz, DMSO) δ 170.9, 166.7, 161.0, 41.1, 34.6 (5 out of 7 carbon resonances observed).

IR (cm^{-1}) 3347 (NH), 3259 (OH), 1716 (C=O acid), 1649 (C=O amide).

M.P. 237.8 – 238.5 ºC (deg.)

MS ES+ m/z calcd for $C_9H_7Cl_2N_5O_3S$ $(M+H)^+$: 335.9725, found: 335.9718

Adapted from literature procedure[21].

NMR data:

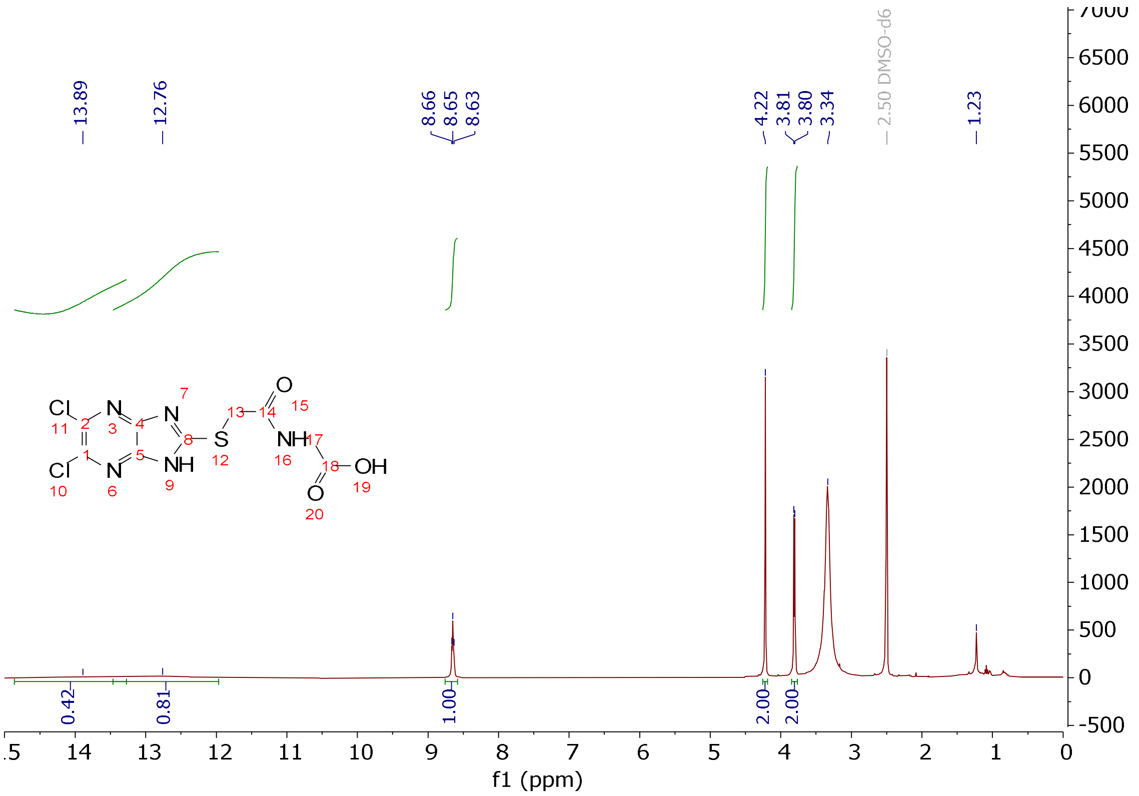
13.89
12.76
8.66
8.65
8.63
4.22
3.81
3.80
3.34
2.50 DMSO-d6
1.23
0.42
0.81
1.00
2.00
2.00
f1 (ppm)
Cl
N
N
S
O
NH
OH
O
NH
N
Cl

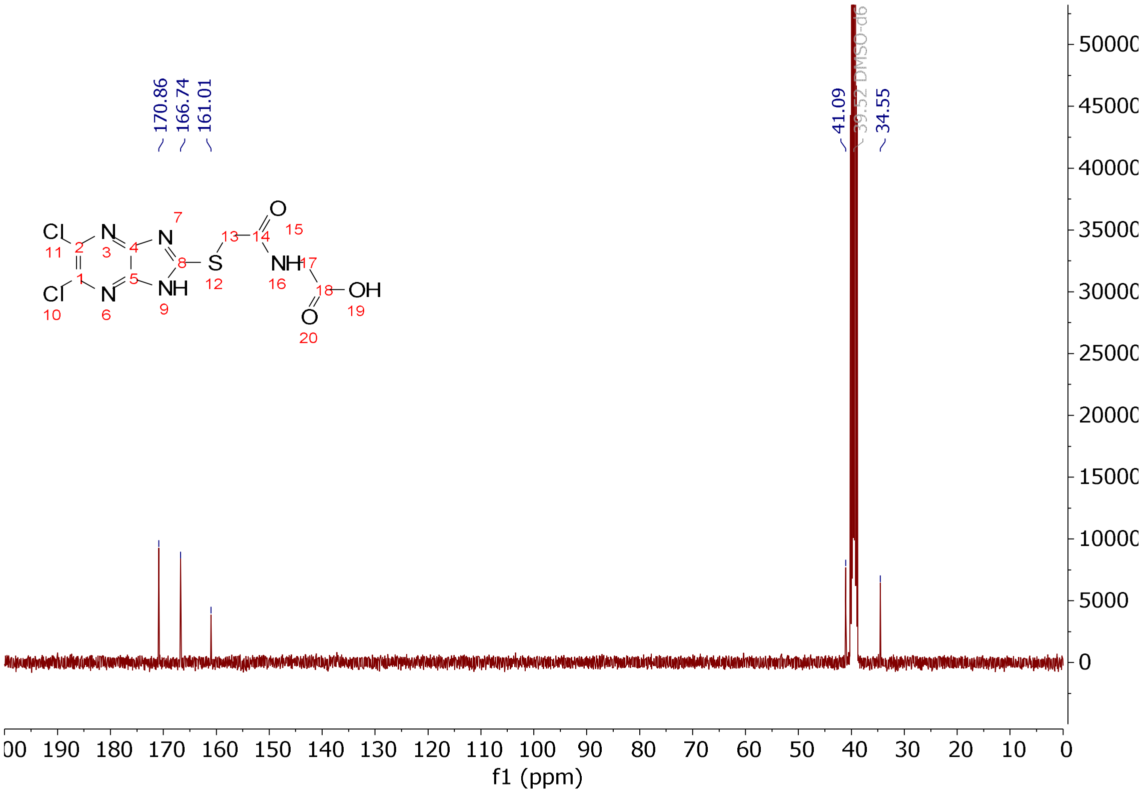
170.86
166.74
161.01
41.09
39.52 DMSO-d6
34.55
f1 (ppm)
Cl
N
N
S
O
NH
OH
O
NH
N
Cl

HRMS data:

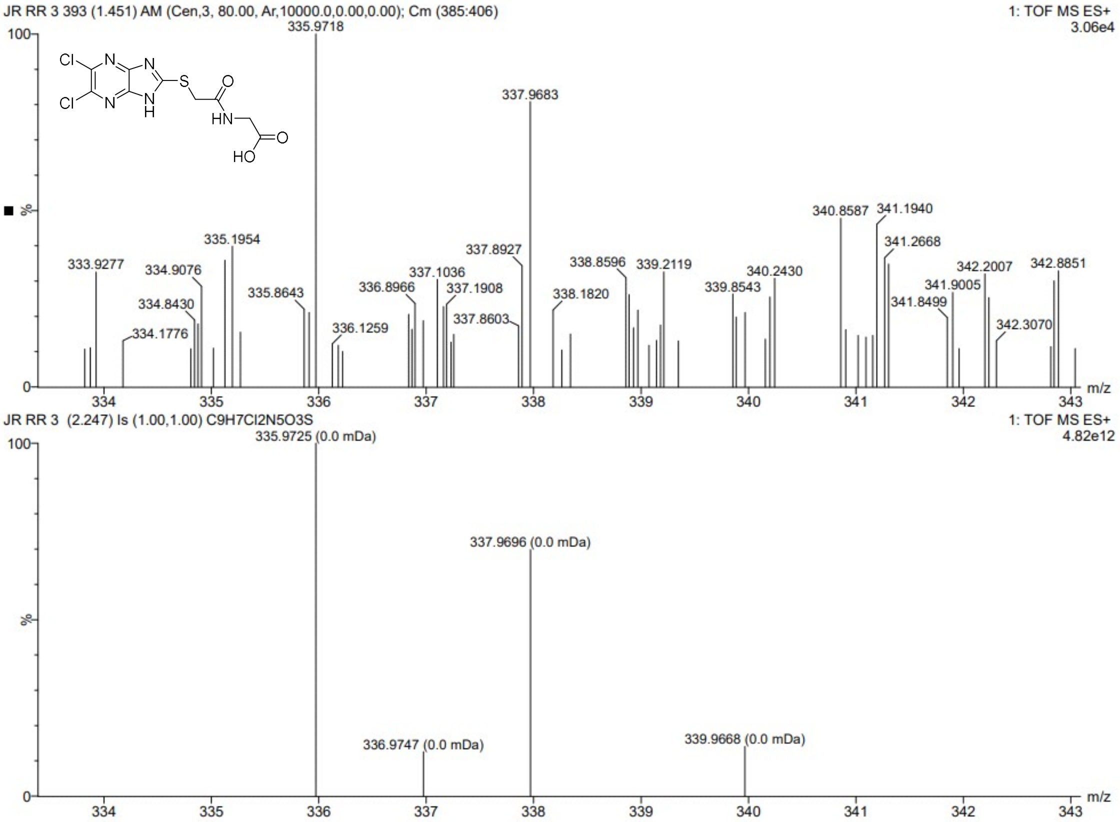
JR RR 3 393 (1.451) AM (Cen,3, 80.00, Ar,10000.0,0.00,0.00); Cm (385:406)
1: TOF MS ES+
3.06e4
335.9718
337.9683
JR RR 3 (2.247) Is (1.00,1.00) C9H7Cl2N5O3S
1: TOF MS ES+
4.82e12
335.9725 (0.0 mDa)
337.9696 (0.0 mDa)
336.9747 (0.0 mDa)
339.9668 (0.0 mDa)
m/z

Compound 17 – *3-hydroxy-2(S)-[({5,6-dichloro-1H-imidazo[4,5-b]pyrazine-2-yl}sulfanyl)acetamido]propanoic acid*

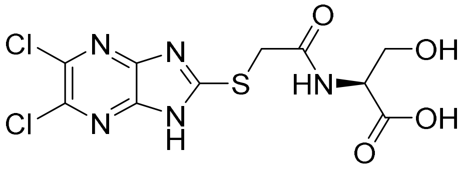

Followed general procedure 1. Methyl 3-hydroxy-2(*S*)-({5,6-dichloro-1*H*-imidazo[4,5-*b*]pyrazine-2-yl}sulfanyl)propanioate (29.7 mg, 0.078 mmol), DCM : MeOH solution (0.781 mL, 0.1 M), NaOH solution (0.468 mL). Provided 3-hydroxy-2(*S*)-[({5,6-dichloro-1*H*-imidazo[4,5-*b*]pyrazine-2-yl}sulfanyl)acetamido]propionic acid as a white solid (24.6 mg, 0.067 mmol, 86%).

^{1}H NMR (400 MHz, DMSO-d_6) δ 14.04 (br s, 1H), 12.69 (br s, 1H), 8.57 (d, J = 7.9 Hz, 1H), 5.03 (br s, 1H), 4.29 (dt, J = 7.9, 4.6 Hz, 1H), 4.26 (s, 2H), 3.71 (dd, J = 10.9, 5.0 Hz, 1H), 3.63 (dd, J = 10.8, 4.2 Hz, 1H).

^{13}C NMR (101 MHz, DMSO-d_6) δ 171.6, 166.5, 161.1, 61.2, 55.1, 34.7 (6 out of 8 carbon resonances observed).

M.P. 139.3 – 140.2 °C (deg.).

IR (cm^{-1}): 3326 (OH), 1721 (C=O acid), 1657 (C=O amide).

MS ES+ m/z calcd for $C_{10}H_9Cl_2N_4O_4S$ (M+H)$^+$: 351.9800, found (1.992 average neutral loss): 349.9878.

Adapted from literature procedure[21].

NMR data:

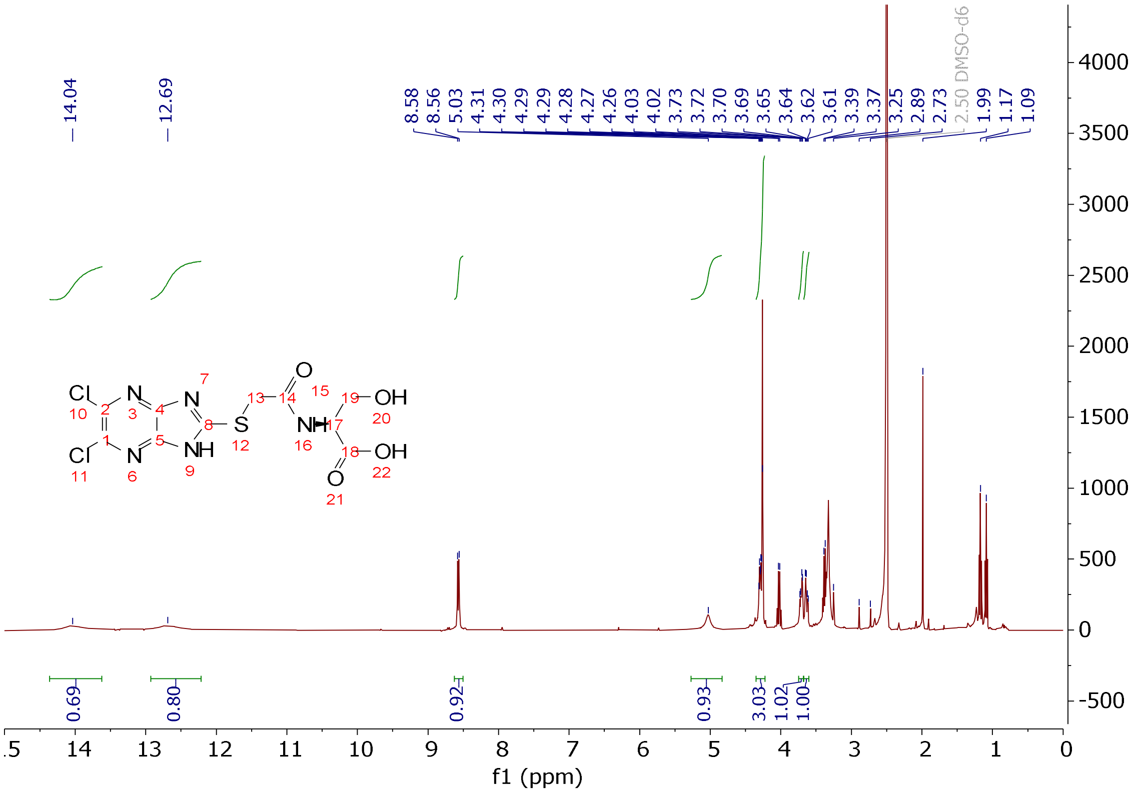

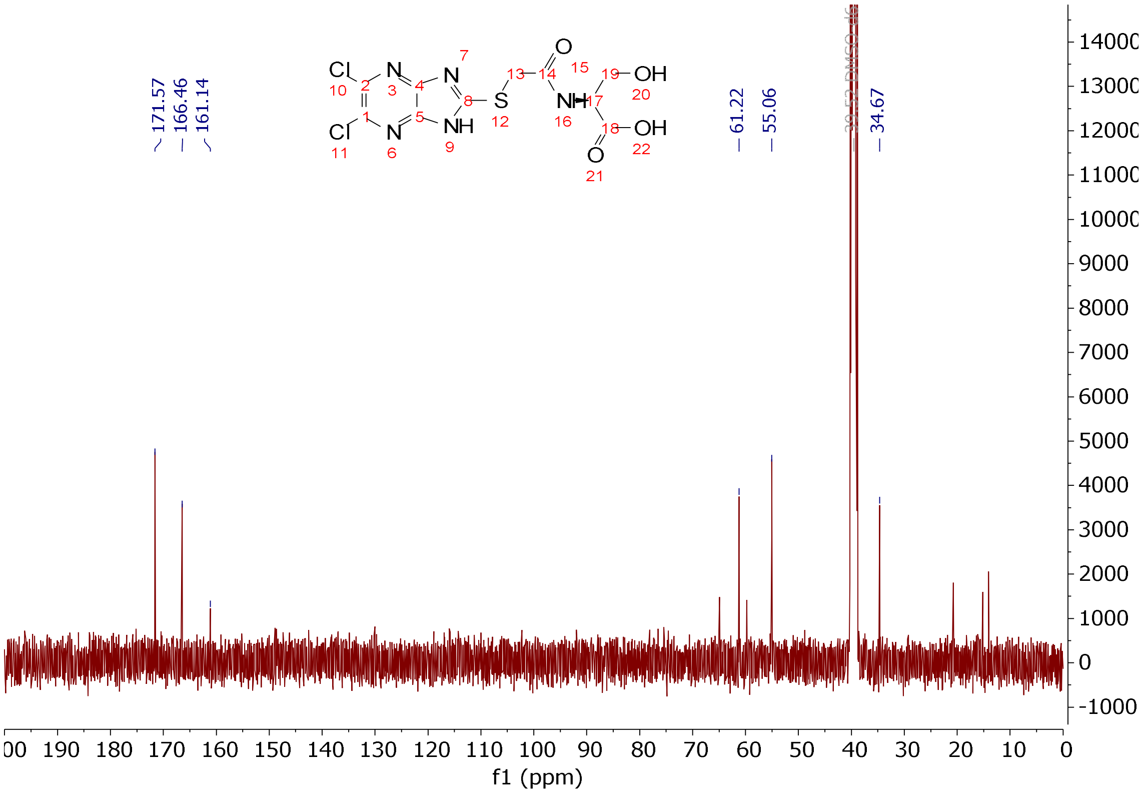

HRMS data:

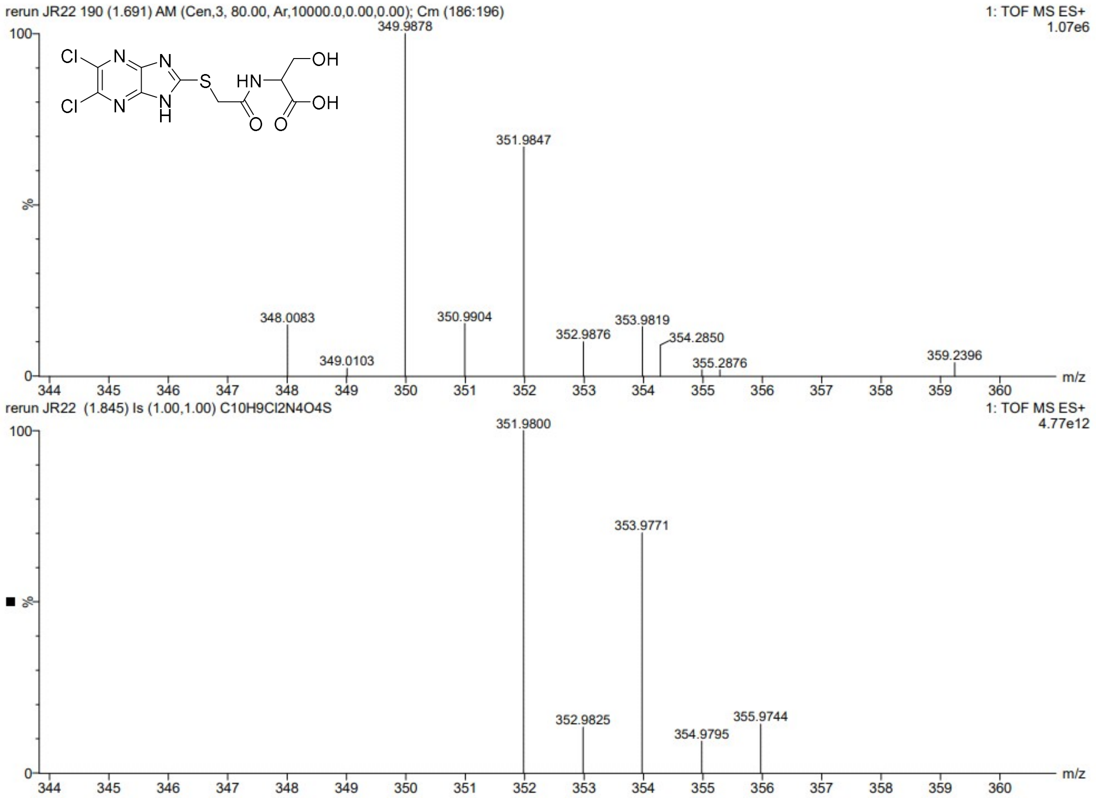
rerun JR22 190 (1.691) AM (Cen,3, 80.00, Ar,10000.0,0.00,0.00); Cm (186:196)
1: TOF MS ES+
1.07e6
Cl
Cl
N
N
N
N
H
S
HN
OH
O
O
OH
349.9878
351.9847
348.0083
349.0103
350.9904
352.9876
353.9819
354.2850
355.2876
359.2396
%
m/z
rerun JR22 (1.845) Is (1.00,1.00) C10H9Cl2N4O4S
1: TOF MS ES+
4.77e12
351.9800
353.9771
352.9825
354.9795
355.9744
%
m/z

Compound 18 – *5,6-dichloroimidazo[4,5-b]pyrazine-2(1,3H)-thione*

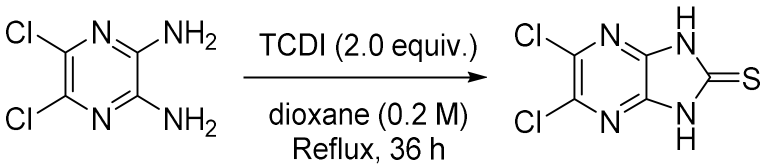

To a 100 mL RBF was added 2,3-diamino-5,6-dichloropyrazine (1.99 g, 11.11 mmol), a stirrer bar and dioxane (56 mL, 0.2 M). This was stirred to dissolve by heating to 80 °C. TCDI (1.98 g, 1.0 equiv.) was added, and the mixture heated to reflux for 24 h. An additional equivalent of TCDI (1.98 g) was added and heating was continued for 12 h. The mixture was allowed to cool to RT and the dioxane removed *in vacuo.* To the residue was added HCl (1 M, 20 mL) and EtOAc (20 mL). The mixture was transferred to a separatory funnel and separated. The aqueous layer was extracted twice with EtOAc (2x 20 mL). The organic layers were collected and dried ($MgSO_4$) and solvent removed *in vacuo.* The crude material was purified by column chromatography, eluting with 8 : 2 Pet. E : EtOAc to provide a yellow solid of 5,6-dichloroimidazo[4,5-*b*]pyrazine-2(1,3*H*)-thione (828 mg, 3.74 mmol, 34%).

^{1}H NMR (400 MHz, DMSO-d_6) δ 13.77 (br s, 2H).

^{13}C NMR (101 MHz, DMSO-d_6) δ 174.2, 140.5, 136.8.

IR (cm^{-1}) 3140 (NH), 1603.

M.P. 360 °C (deg.).

MS ES+ m/z calcd for $C_5H_2Cl_2N_4S$ $(M+H)^+$: 220.9455, found: 220.9455.

NMR data:

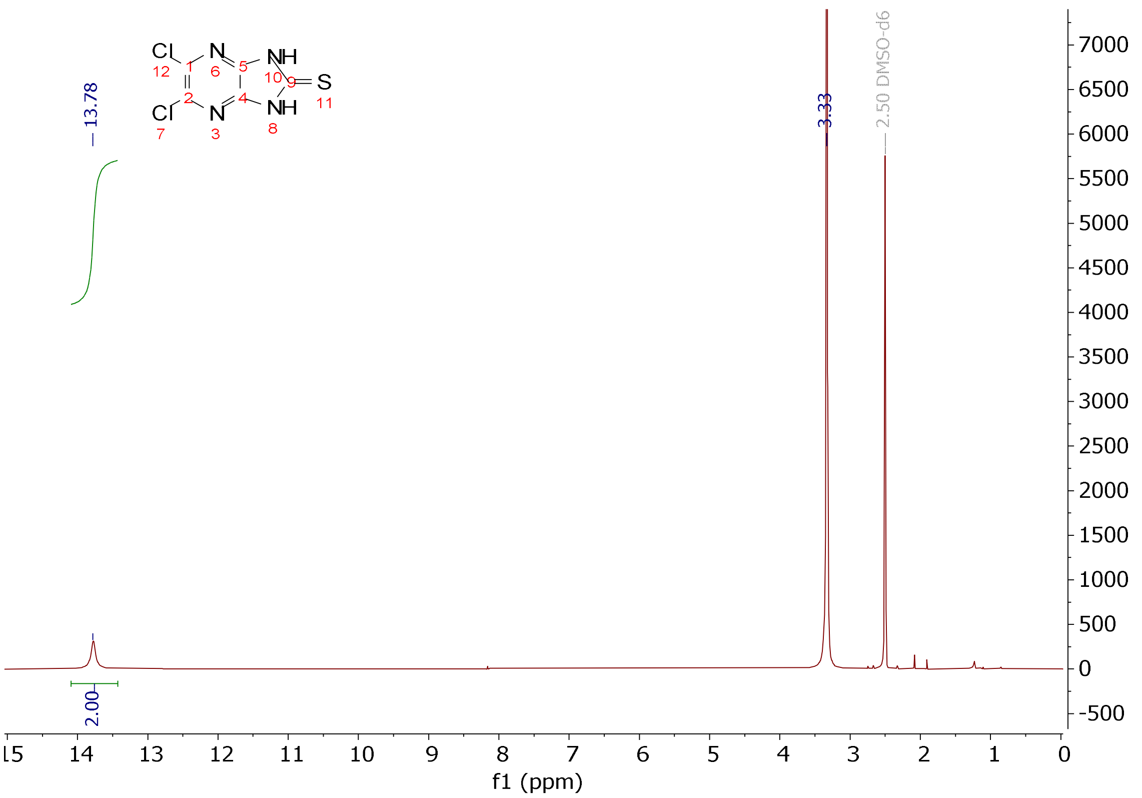

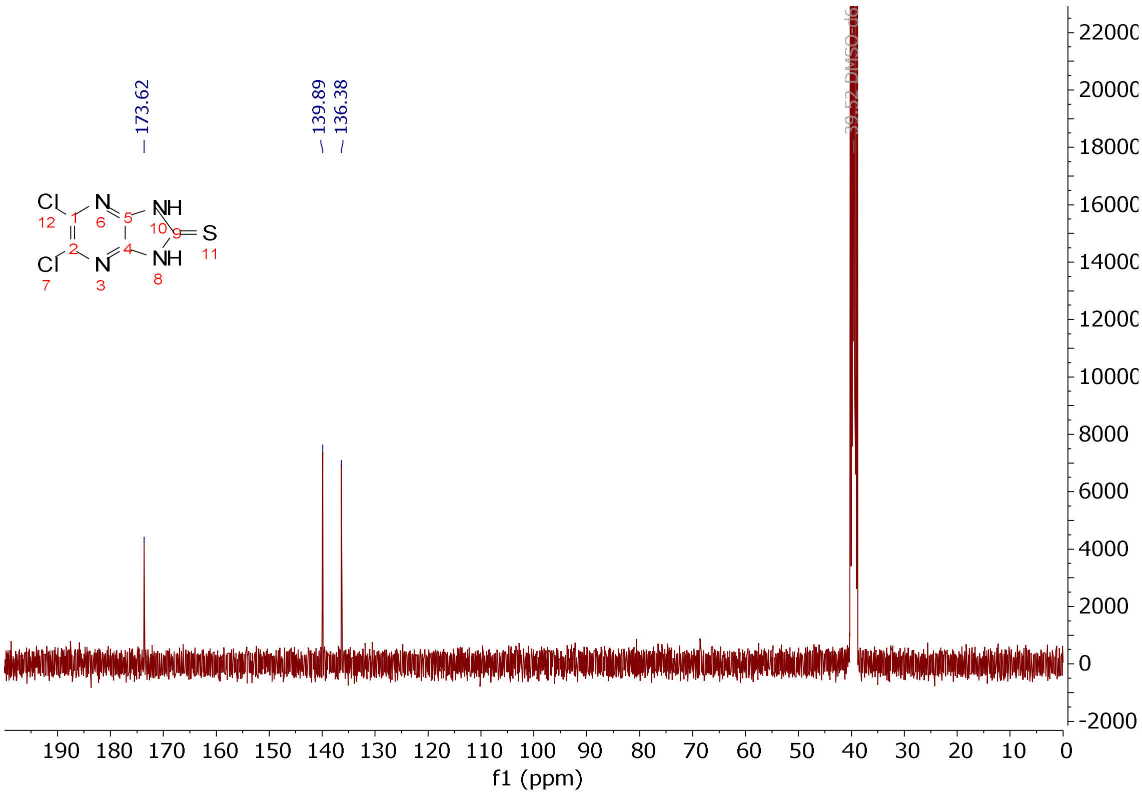

HRMS data:

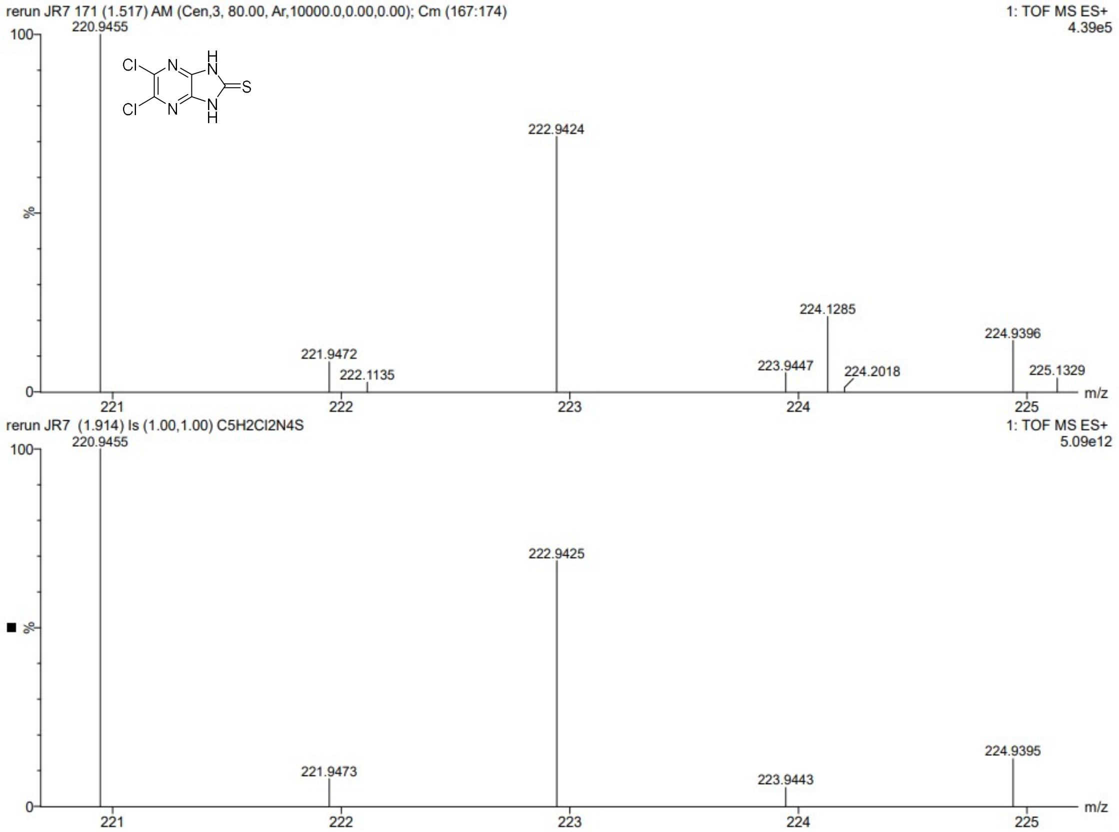
rerun JR7 171 (1.517) AM (Cen,3, 80.00, Ar,10000.0,0.00,0.00); Cm (167:174)
1: TOF MS ES+
4.39e5
220.9455
222.9424
221.9472
222.1135
223.9447
224.1285
224.2018
224.9396
225.1329
m/z
%
Cl
N
H
S
rerun JR7 (1.914) Is (1.00,1.00) C5H2Cl2N4S
1: TOF MS ES+
5.09e12
220.9455
222.9425
221.9473
223.9443
224.9395
221
222
223
224
225

Compound 19 – *Ethyl 2-({1H-imidazo[4,5-b]pyridine}sulfan-2-yl)acetate*

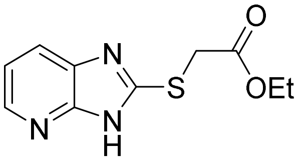

Prepared according to general procedure 3. Imidazo[4,5-*b*]pyridine-2(1,3*H*)-thione (0.1 g, 0.66 mmol), ethyl bromoacetate (77 µL), sodium hydroxide (39.6 mg), EtOH (3.3 mL). Purified by column chromatography using 5:5 -> 6:4 EtOAc : Pet.E. Recrystallised from EtOH to provide ethyl 2-({1*H*-imidazo[4,5-*b*]pyridine}sulfan-2-yl)acetate a white solid (51.4 mg, 0.22 mmol, 33%).

^{1}H NMR (400 MHz, DMSO-d_6) δ 13.10 (br s, 1H), 8.20 (dd, J = 4.9, 1.5 Hz, 1H), 7.82 (dd, J = 7.9, 1.5 Hz, 1H), 7.15 (dd, J = 7.9, 4.9 Hz, 1H), 4.24 (s, 2H), 4.13 (q, J = 7.1 Hz, 2H), 1.18 (t, J = 7.1 Hz, 3H).

^{13}C NMR (101 MHz, DMSO-d_6) δ 168.4, 152.4, 142.5, 117.4, 61.2, 32.9, 14.0.

IR (cm^{-1}): 3003 (NH), 1744 (C=O).

M.P. 157.6 – 160.2 ºC.

MS ES+ m/z calcd for $C_{10}H_{11}N_3O_2S$ $(M+H)^+$: 239.0676, found: 239.0675.

Data in-line with literature data[22].

NMR data:

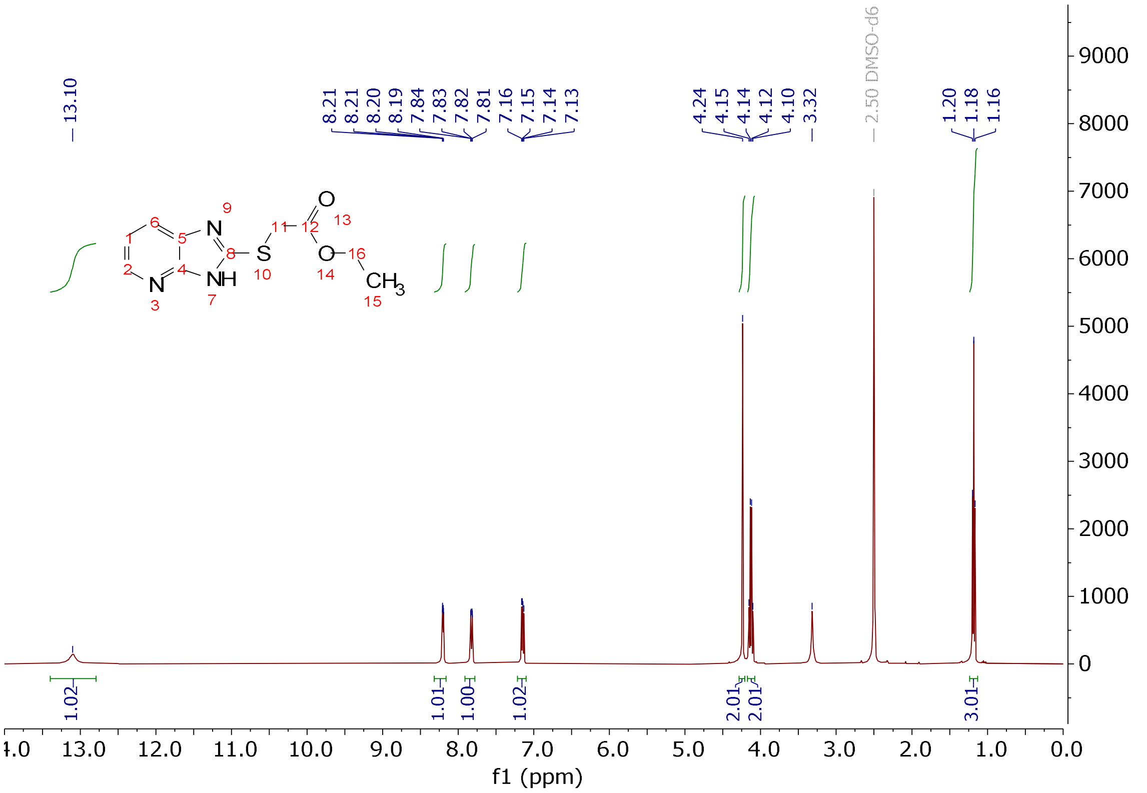

13.10
8.21
8.21
8.20
8.19
7.84
7.83
7.82
7.81
7.16
7.15
7.14
7.13
4.24
4.15
4.14
4.12
4.10
3.32
2.50 DMSO-d6
1.20
1.18
1.16
1.02
1.01
1.00
1.02
2.01
2.01
3.01
f1 (ppm)

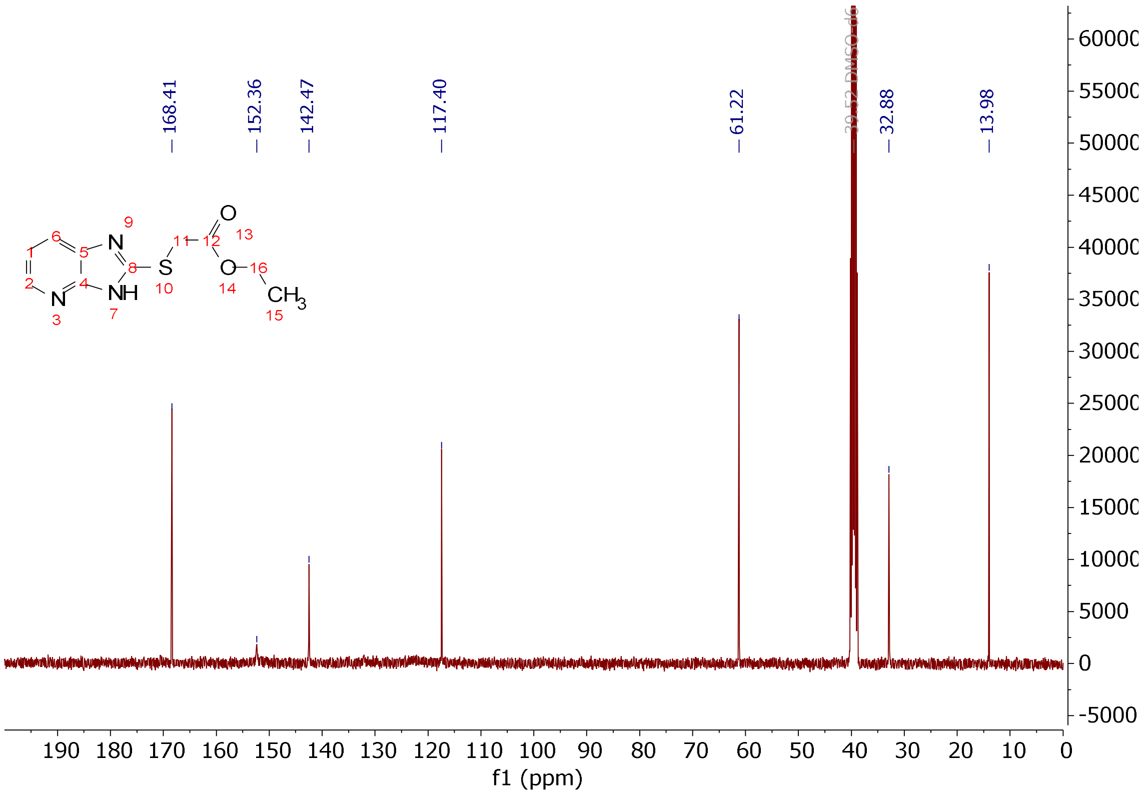

168.41
152.36
142.47
117.40
61.22
32.88
13.98
f1 (ppm)

HRMS data:

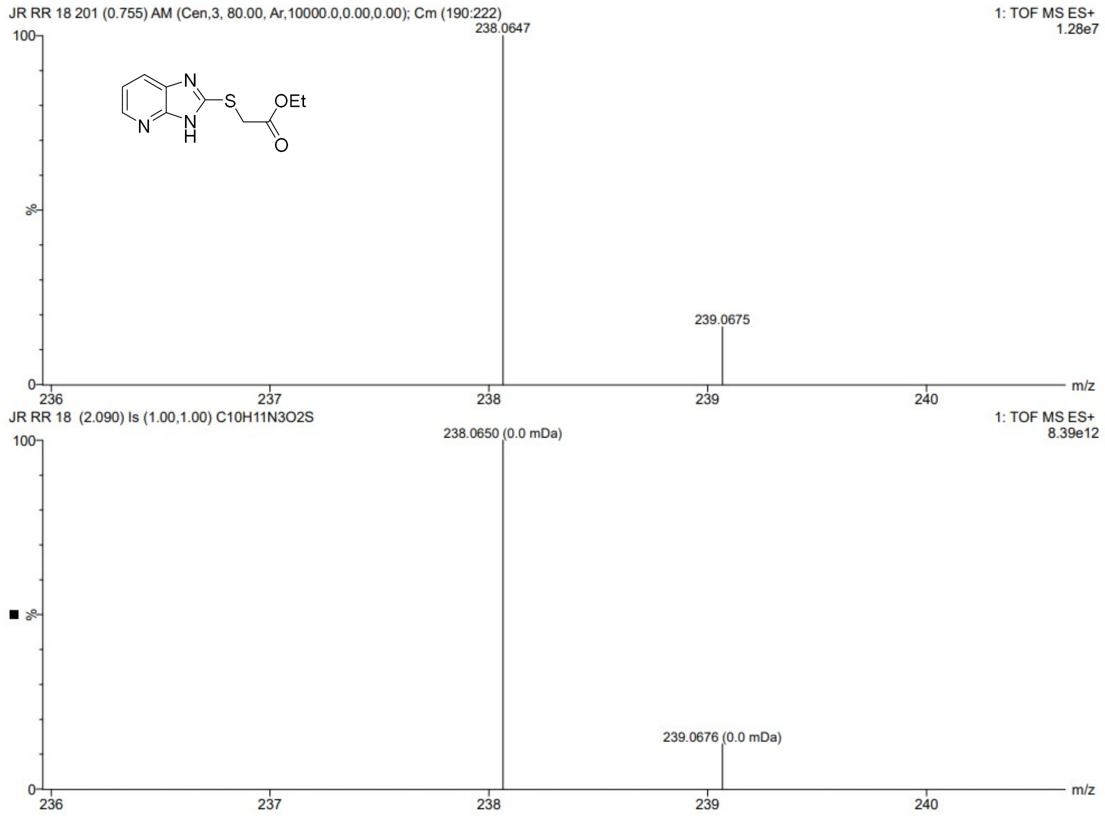

JR RR 18 201 (0.755) AM (Cen,3, 80.00, Ar,10000.0,0.00,0.00); Cm (190:222)
1: TOF MS ES+
1.28e7
238.0647
239.0675
N
S
OEt
N
N
H
O
100
%
0
236
237
238
239
240
m/z
JR RR 18 (2.090) Is (1.00,1.00) C10H11N3O2S
1: TOF MS ES+
8.39e12
238.0650 (0.0 mDa)
239.0676 (0.0 mDa)
100
%
0
236
237
238
239
240
m/z

Compound 20 – *Ethyl 2-({1H-imidazo[4,5-b]pyrazine}sulfanyl-2-yl)acetate*

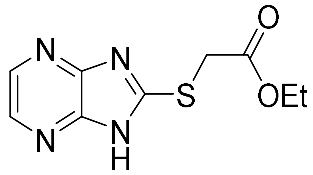

Prepared according to general procedure 3. Imidazo[4,5-*b*]pyrazine-2(1,3*H*)-thione (0.1 g, 0.66 mmol), ethyl bromoacetate (76.5 µL), sodium hydroxide (39.4 mg), EtOH (3.3 mL). Purified by column chromatography using 7:3 -> 5:5 -> 4:6 Pet.E : EtOAc to provide ethyl 2-({1*H*-imidazo[4,5-*b*]pyrazine}sulfanyl-2-yl)acetate a white solid. Recrystallised from EtOH with a hot filtration (41.9 mg, 0.18 mmol, 27%).

^{1}H NMR (400 MHz, DMSO-d_6) δ 13.68 (br s, 1H), 8.25 (s, 2H), 4.29 (s, 2H), 4.15 (q, J = 7.1 Hz, 2H), 1.20 (t, J = 7.1 Hz, 3H).

^{13}C NMR (101 MHz, DMSO-d_6) δ 168.1, 157.4, 146.0, 137.4, 61.4, 32.8, 14.0.

IR (cm^{-1}): 1733 (C=O).

M.P.: 177.4 – 175.2 ºC.

MS ES+ m/z calcd for $C_9H_{10}N_4O_2S$ $(M+H)^+$: 239.0603, found: 239.0628.

NMR data:

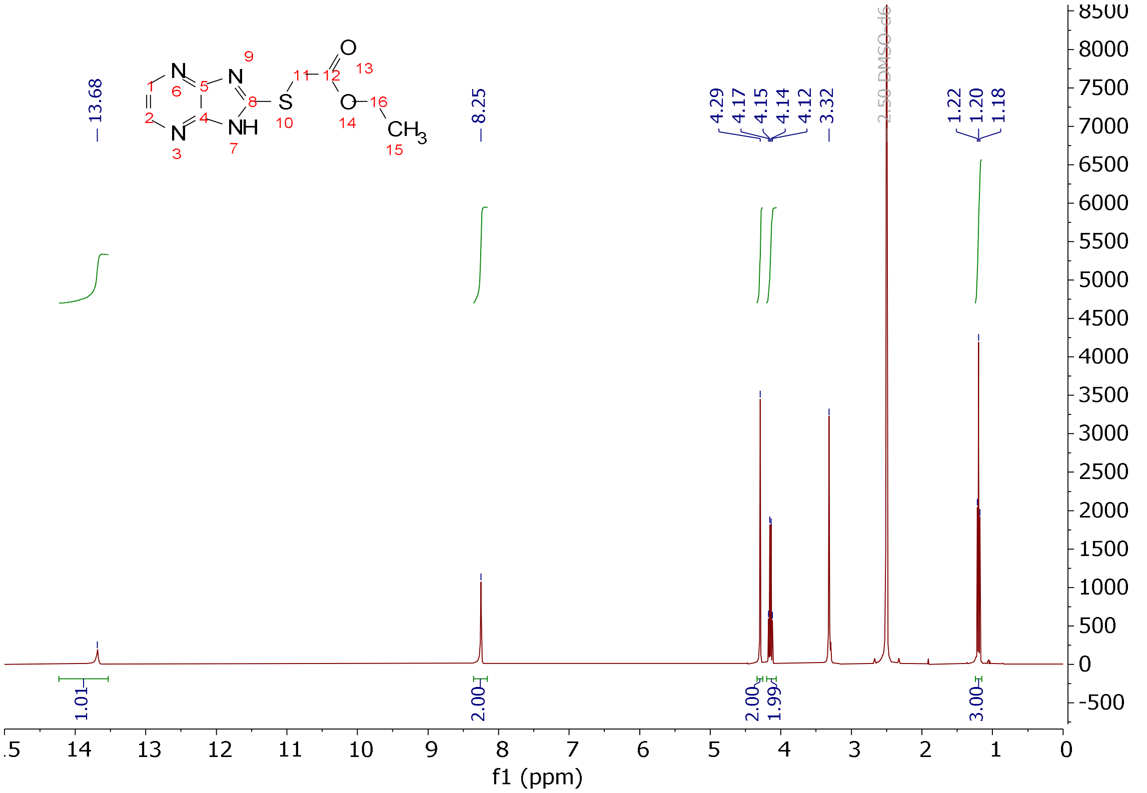
13.68
8.25
4.29
4.17
4.15
4.14
4.12
3.32
2.50 DMSO-d6
1.22
1.20
1.18
1.01
2.00
2.00
1.99
3.00
f1 (ppm)

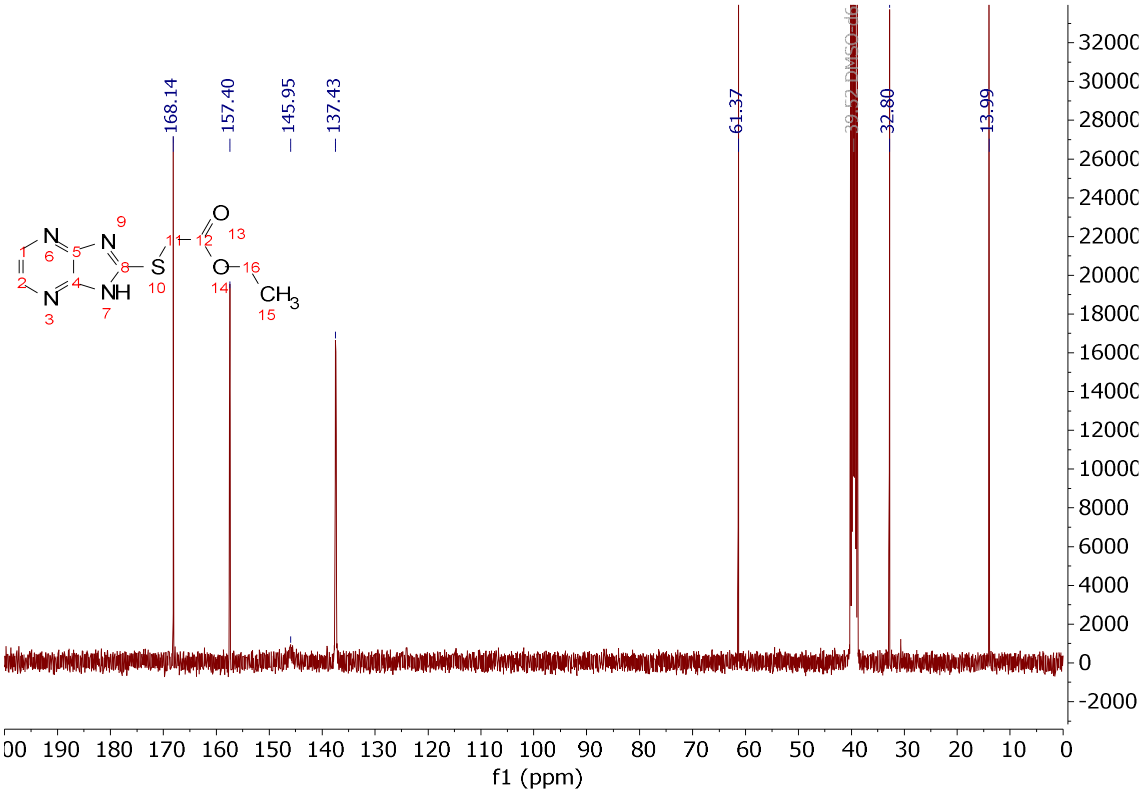
168.14
157.40
145.95
137.43
61.37
39.52 DMSO-d6
32.80
13.99
f1 (ppm)

HRMS data:

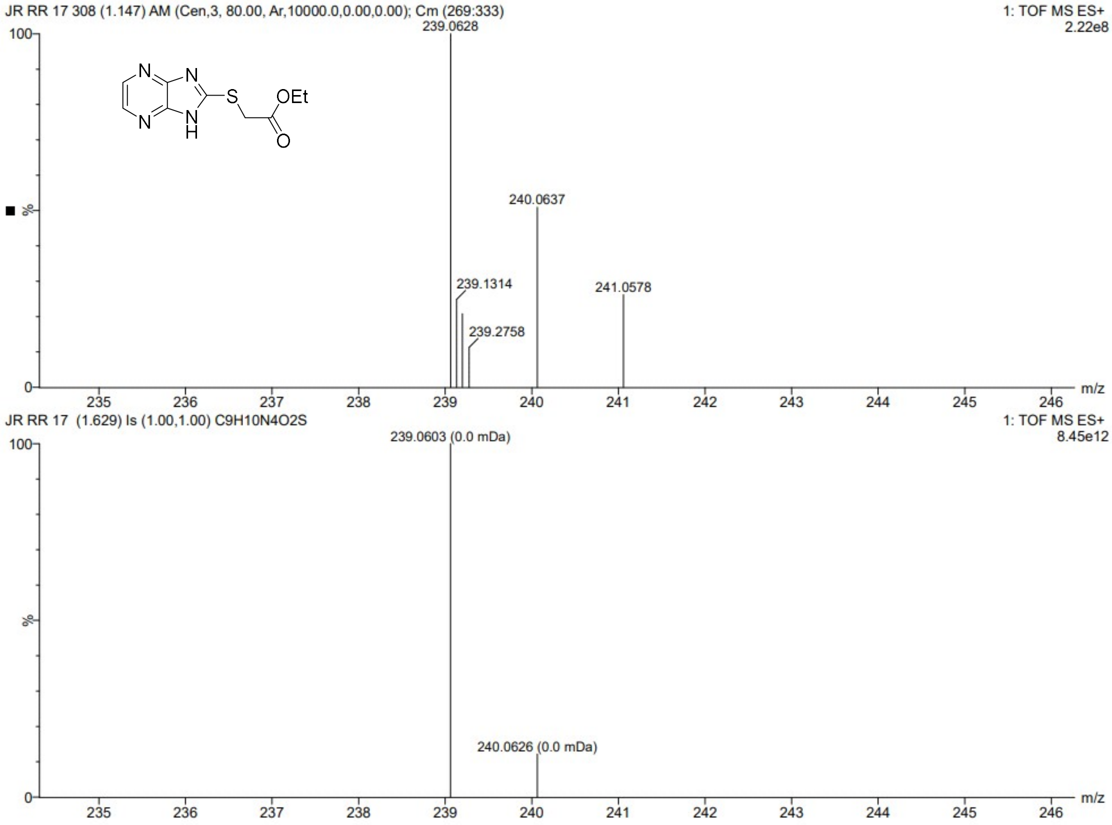
JR RR 17 308 (1.147) AM (Cen,3, 80.00, Ar,10000.0,0.00,0.00); Cm (269:333)
1: TOF MS ES+
2.22e8
239.0628
240.0637
239.1314
241.0578
239.2758
OEt
S
N
H
O
m/z
JR RR 17 (1.629) Is (1.00,1.00) C9H10N4O2S
1: TOF MS ES+
8.45e12
239.0603 (0.0 mDa)
240.0626 (0.0 mDa)
%
100
0
235
236
237
238
239
240
241
242
243
244
245
246

Compound 21 – *Ethyl 2-({9H-imidazo[4,5-d]pyrimidine}sulfan-8-yl)acetate*

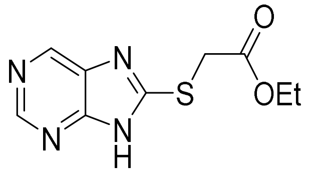

Prepared according to general procedure 3. Imidazo[4,5-*b*]pyrimidine-2(1,3*H*)-thione (0.075 g, 0.474 mmol), ethyl bromoacetate (55.2 μL), sodium hydroxide (28 mg), EtOH (2.4 mL). Purified by column chromatography using 5:5 -> 4:6 Pet.E : EtOAc to provide ethyl 2-({9*H*-imidazo[4,5-*d*]pyrimidine}sulfan-8-yl)acetate as a white solid (41.9 mg, 0.18 mmol, 37%).

^{1}H NMR (400 MHz, DMSO-d_6) δ 13.62 (br s, 1H), 8.88 (s, 1H), 8.78 (s, 1H), 4.27 (s, 2H), 4.14 (q, J = 7.1 Hz, 2H), 1.19 (t, J = 7.1 Hz, 3H).

^{13}C NMR (101 MHz, DMSO-d_6) δ 168.0, 156.2, 150.8, 141.4, 131.97, 61.1, 32.8, 13.8.

IR (cm^{-1}): 1742 (C=O).

M.P. 140.3 – 141.0 °C.

MS ES+ m/z calcd for $C_9H_{10}N_4O_2S$ $(M+H)^+$: 240.0626, found: 240.0632.

NMR data:

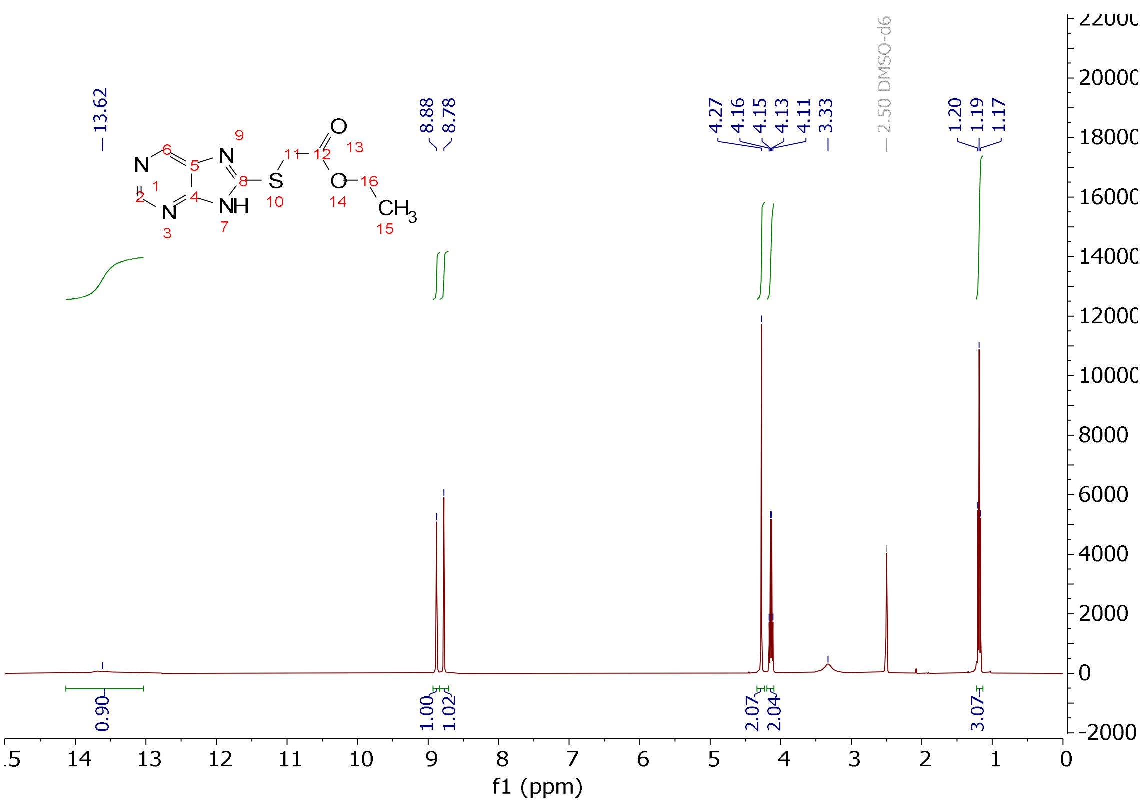
13.62
8.88
8.78
4.27
4.16
4.15
4.13
4.11
3.33
2.50 DMSO-d6
1.20
1.19
1.17
0.90
1.00
1.02
2.07
2.04
3.07
f1 (ppm)

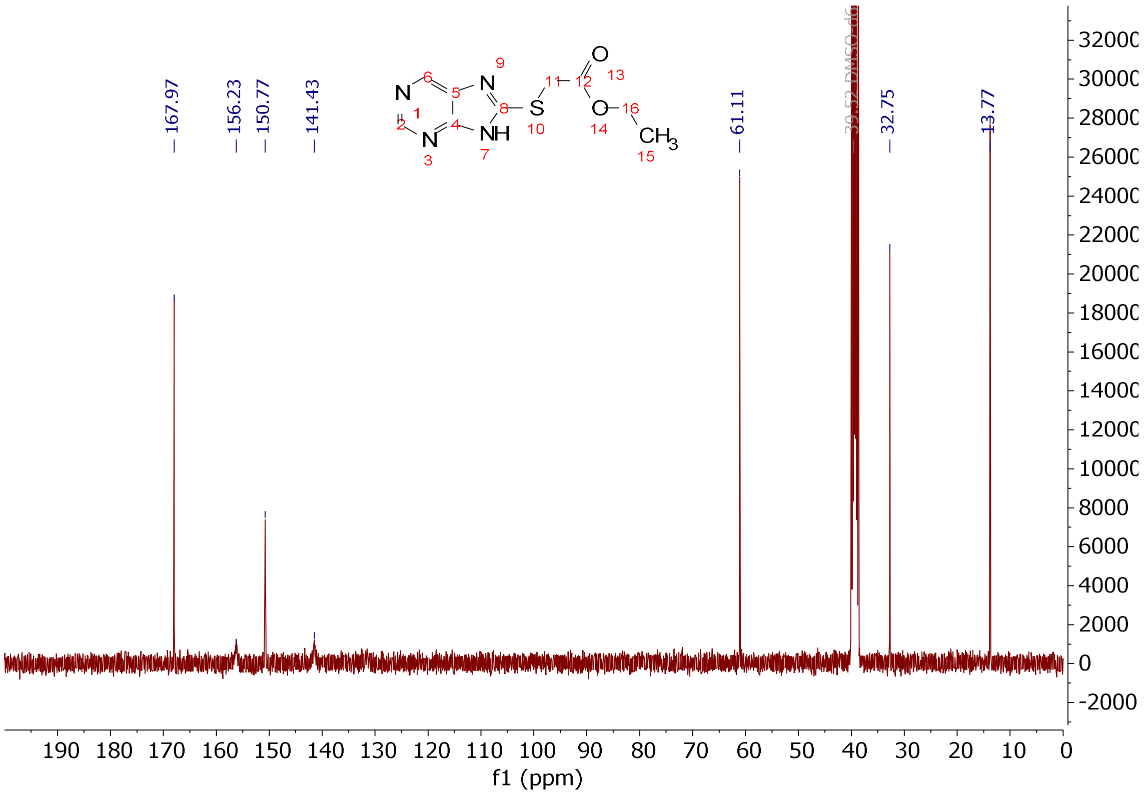
167.97
156.23
150.77
141.43
61.11
32.75
13.77
f1 (ppm)

HRMS data:

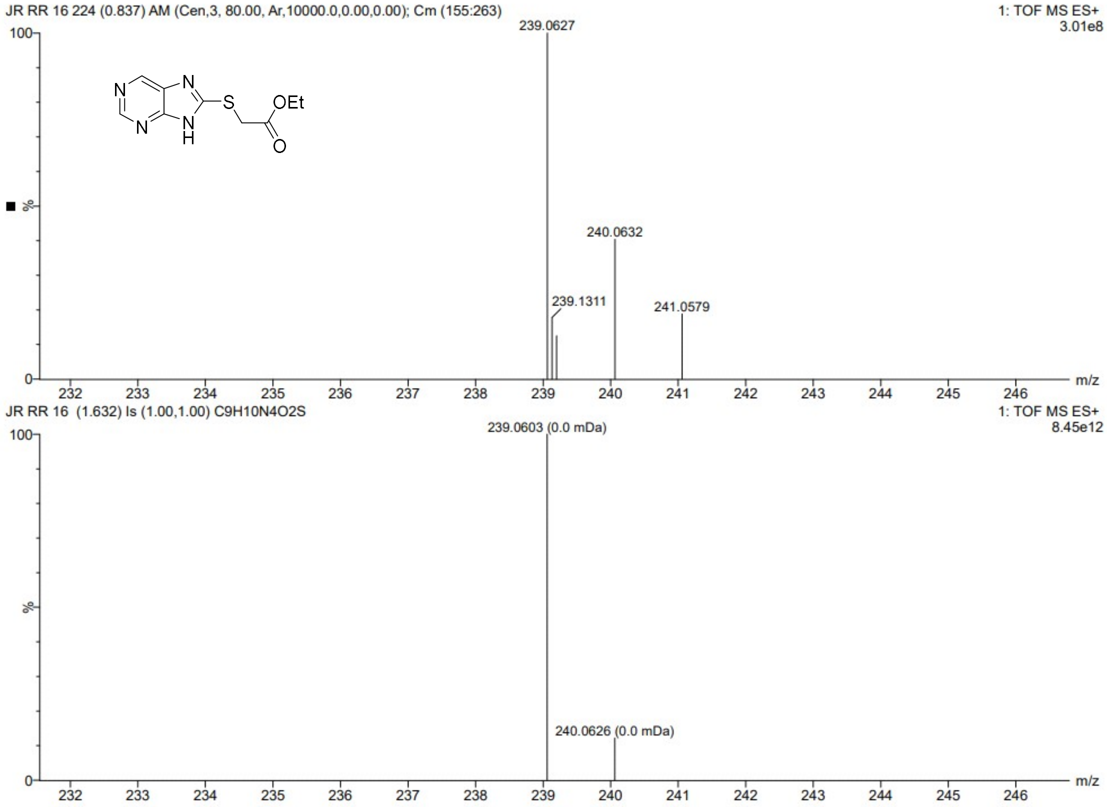
JR RR 16 224 (0.837) AM (Cen,3, 80.00, Ar,10000.0,0.00,0.00); Cm (155:263)
1: TOF MS ES+
3.01e8
239.0627
240.0632
239.1311
241.0579
N
N
N
N
H
S
OEt
O
%
m/z
JR RR 16 (1.632) Is (1.00,1.00) C9H10N4O2S
1: TOF MS ES+
8.45e12
239.0603 (0.0 mDa)
240.0626 (0.0 mDa)
%
m/z

Compound 22 – *Ethyl 2-({6-chloro-9H-imidazo[4,5-d]pyrimidine}sulfan-8-yl)acetate*

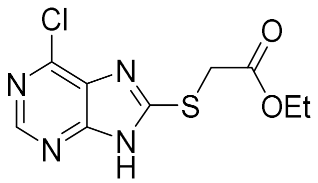

Prepared according to general procedure 3. 6-chloroimidazo[4,5-*d*]pyrimidine-8(7,9*H*)-thione (0.1 g, 0.535 mmol), ethyl bromoacetate (62.3 µL), sodium hydroxide (32 mg). EtOH (2.7 mL). Purified by column chromatography using 5:5 Pet. E : EtOAc. Recrystallised from EtOH to provide ethyl 2-({6-chloro-9*H*-imidazo[4,5-*d*]pyrimidine}sulfan-8-yl)acetate a white solid (35.5 mg, 0.13 mmol, 24%).

^{1}H NMR (400 MHz, DMSO-d_6) δ 14.09 (br s, 1H), 8.61 (s, 1H), 4.29 (s, 2H), 4.15 (q, J = 7.1 Hz, 2H), 1.20 (t, J = 7.1 Hz, 3H).

^{13}C NMR (101 MHz, DMSO-d_6) δ 168.5, 155.5, 151.2, 61.8, 33.5, 14.5 (6 out of 9 carbon resonances observed).

IR (cm^{-1}): 3062 (NH), 1727 (C=O).

M.P. 151.5 – 153.3 °C.

MS ES+ m/z calcd for $C_9H_9ClN_4O_2S$ $(M+H)^+$: 274.0237, found: 274.0244.

NMR data:

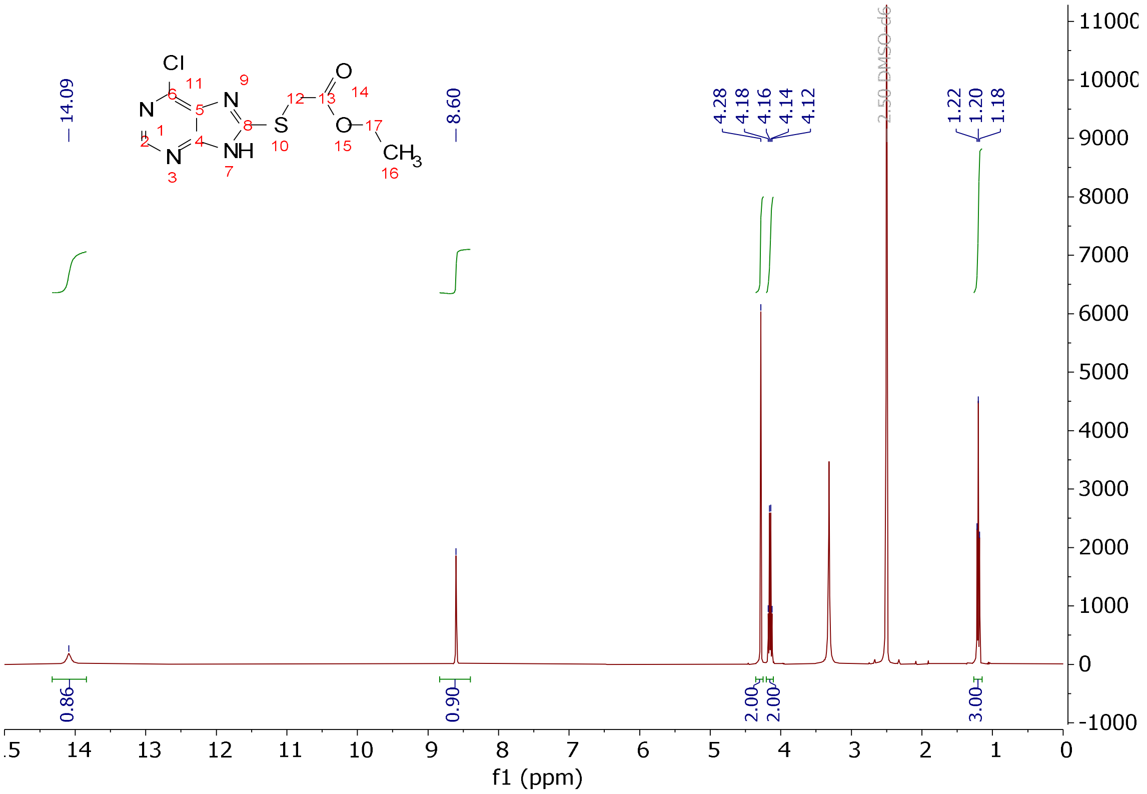

14.09
8.60
4.28
4.18
4.16
4.14
4.12
2.50 DMSO-d6
1.22
1.20
1.18
0.86
0.90
2.00
2.00
3.00
f1 (ppm)

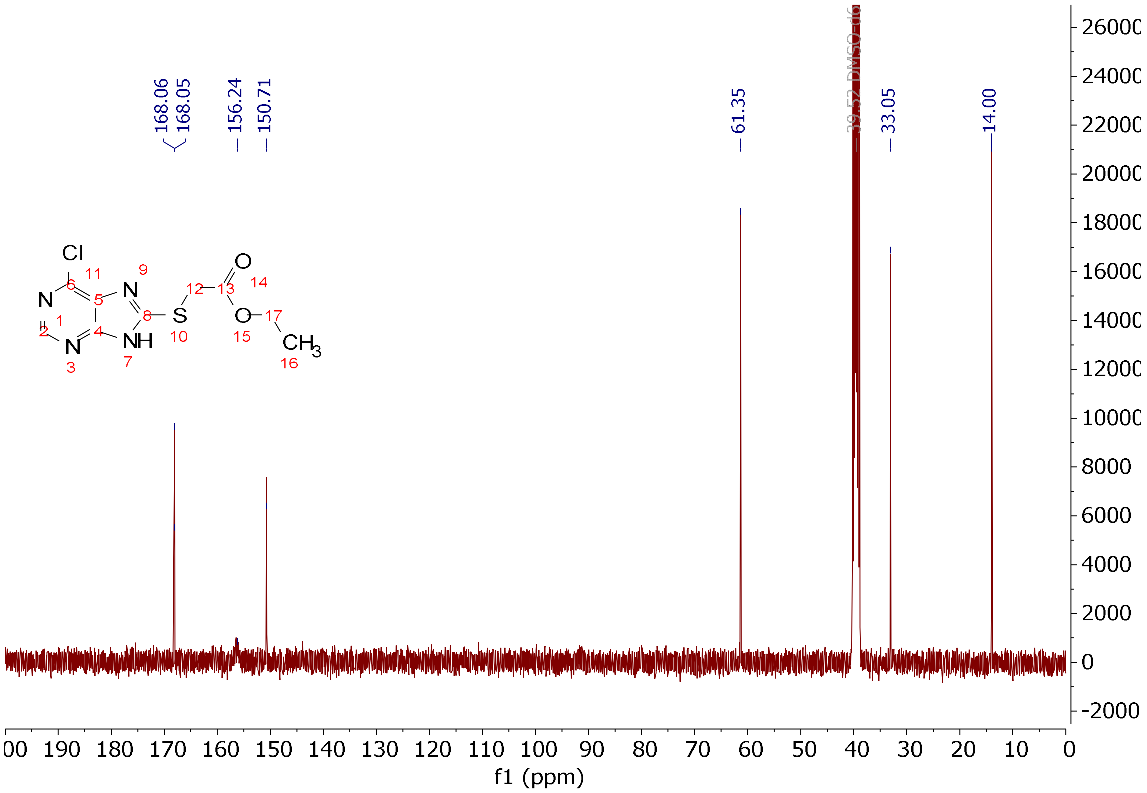

168.06
168.05
156.24
150.71
61.35
39.52 DMSO-d6
33.05
14.00
f1 (ppm)

HRMS data:

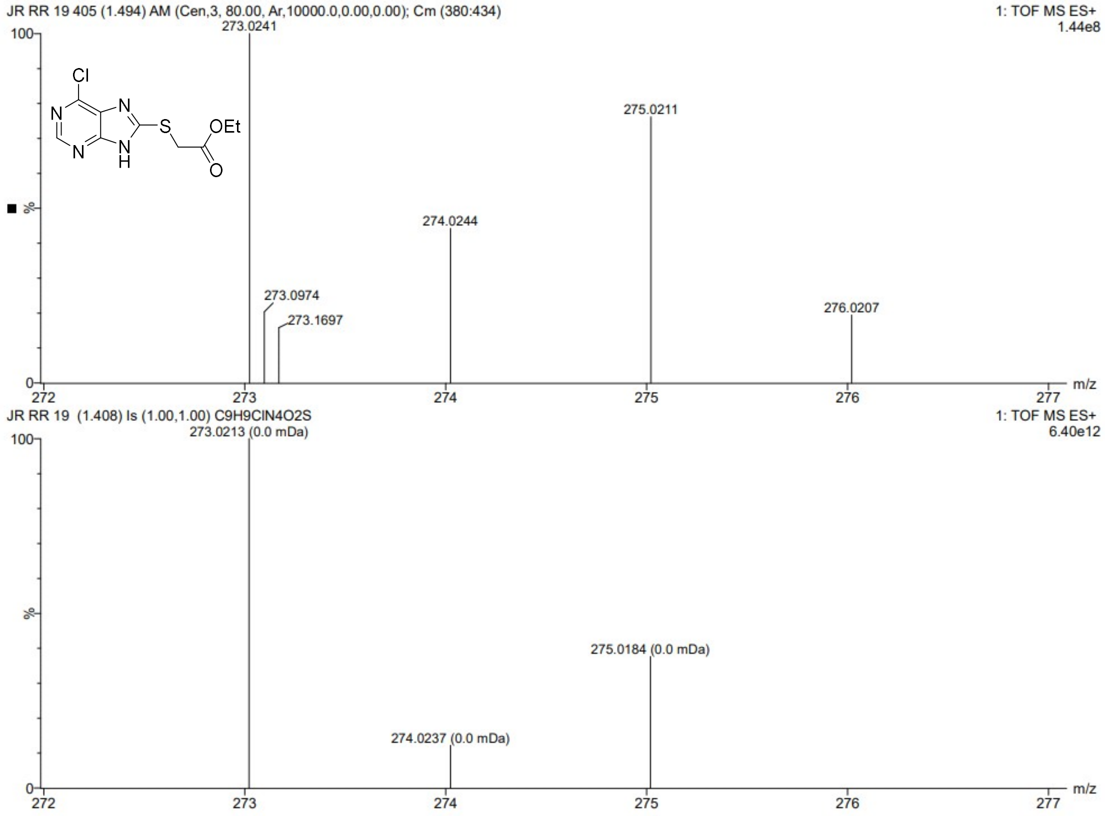
JR RR 19 405 (1.494) AM (Cen,3, 80.00, Ar,10000.0,0.00,0.00); Cm (380:434)
1: TOF MS ES+
1.44e8
273.0241
275.0211
274.0244
273.0974
273.1697
276.0207
Cl
N
N
N
N
H
S
OEt
O
100
%
0
272
273
274
275
276
277
m/z
JR RR 19 (1.408) Is (1.00,1.00) C9H9ClN4O2S
1: TOF MS ES+
6.40e12
273.0213 (0.0 mDa)
275.0184 (0.0 mDa)
274.0237 (0.0 mDa)

Compound 23 – *Ethyl 2-[({imidazo[4,5-c]pyridine}sulfan-2-yl-5N-(ethyl-2-acetate))]acetate*

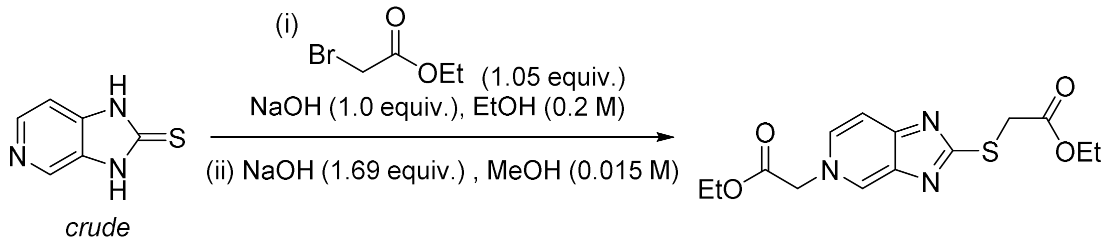

To the crude imidazo[4,5-*c*]pyridine (0.392 g) was added EtOH (13 mL), sodium hydroxide (1.0 equiv. 130 mg, accounting for the 76% conversion) and ethyl bromoacetate (1.05 equiv., 0.187 mL, accounting for the 76% conversion). Stirred at RT for 18 h. The solvent was removed, and residue taken up in NH_4Cl sat. soln. and EtOAc (*ca.* 5 mL each) the layers were separated and the aqueous extracted with EtOAc (2x 10 mL). Organic layers collected and dried ($MgSO_4$) and solvent removed to provide an oil. Purified using column chromatography (5% MeOH / DCM) to isolate a white solid which was recrystallised from EtOH and triturated using EtOH to provide what is thought to be the hydrobromide (or chloride) salt.

Material from the EtOH trituration (30 mg) was dissolved in MeOH (*ca.* 5 mL) and sodium hydroxide was added (5 mg). Left to stir for 18 h at RT. The mixture was diluted with H_2O and EtOAc, transferred to a separatory funnel and extracted with EtOAc (3x 10 mL). Organic layers were collected and dried ($MgSO_4$). Crude material was purified by column chromatography (3 -> 4% MeOH / DCM) to provide product as a white solid (24.5 mg, 0.075 mmol).

^{1}H NMR (400 MHz, DMSO-d_6) δ 8.68 (d, J = 1.5 Hz, 1H), 7.99 (dd, J = 6.9, 1.5 Hz, 1H), 7.54 (d, J = 6.9 Hz, 1H), 5.38 (s, 2H), 4.20 (q, J = 7.1 Hz, 2H), 4.15 (s, 2H), 4.12 (q, J = 7.1 Hz, 2H), 1.23 (t, J = 7.1 Hz, 2H), 1.19 (t, J = 7.1 Hz, 3H).

^{13}C NMR (101 MHz, DMSO-d_6) δ 172.1, 169.5, 168.0, 156.4, 144.9, 132.8, 130.2, 109.9, 61.9, 61.1, 58.4, 33.5, 14.3, 14.2.

IR (cm^{-1}): 1730 (C=O).

M.P. 146.8 – 147.3 °C.

MS ES+ m/z calcd for $C_{14}H_{17}N_3O_4S$ $(M+H)^+$: 325.1046, found: 325.1067.

Adapted from literature procedure[5].

NMR data:

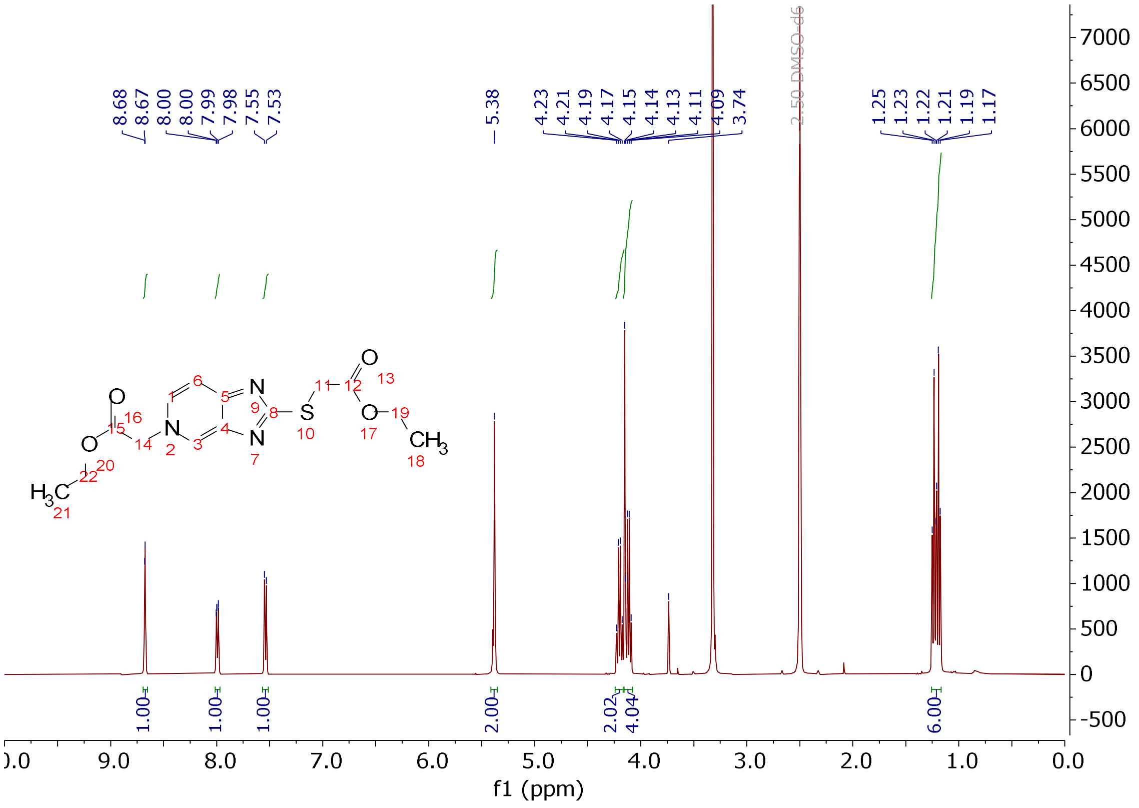

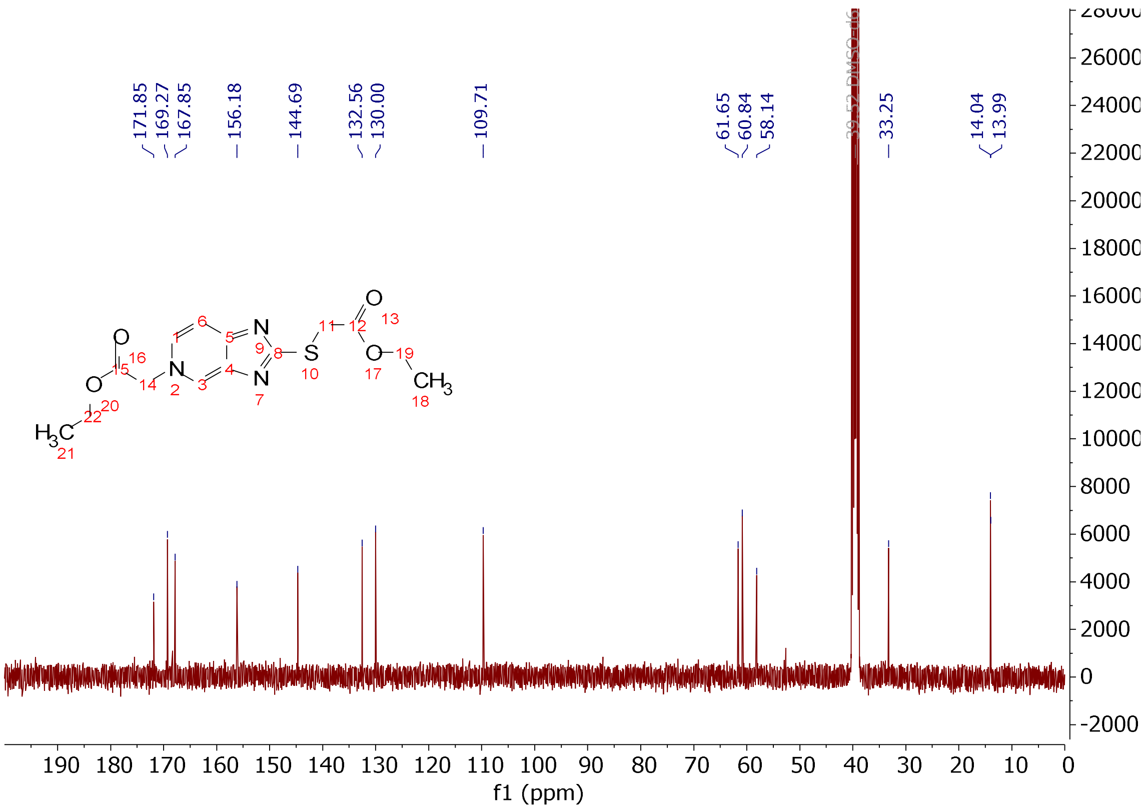
171.85
169.27
167.85
156.18
144.69
132.56
130.00
109.71
61.65
60.84
58.14
33.25
14.04
13.99
f1 (ppm)

HRMS data:

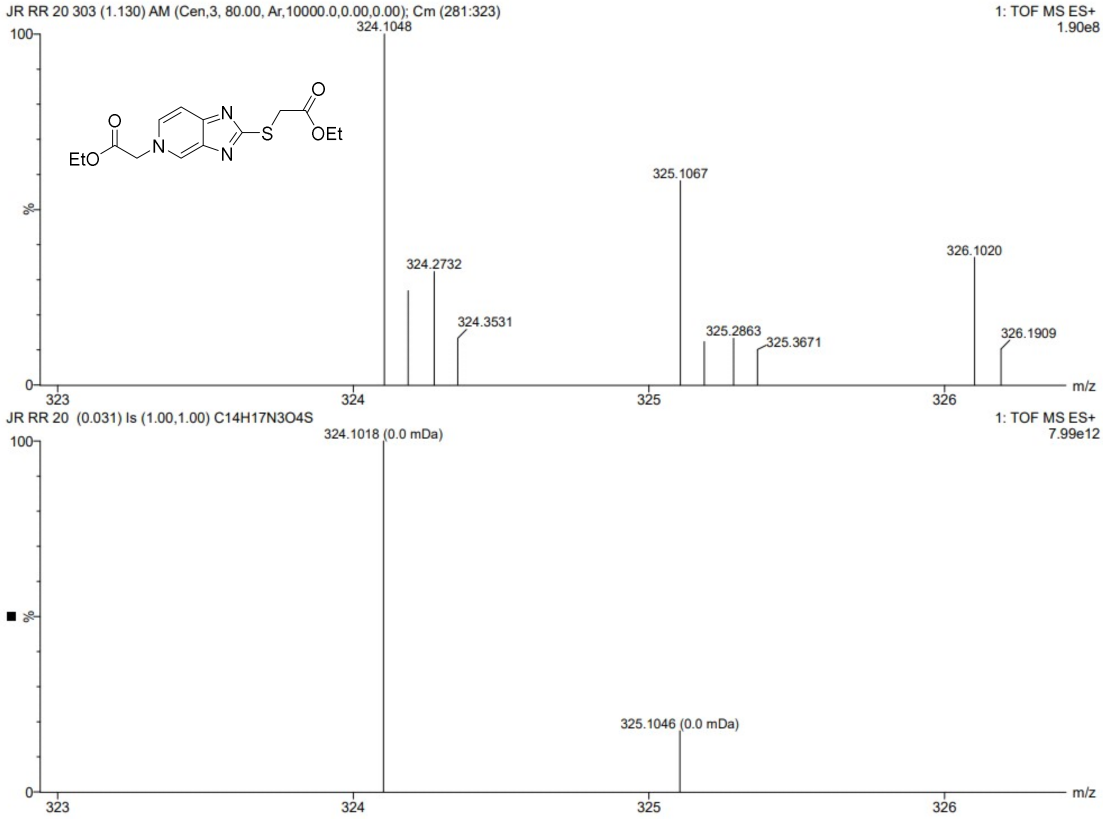
JR RR 20 303 (1.130) AM (Cen,3, 80.00, Ar,10000.0,0.00,0.00); Cm (281:323)
1: TOF MS ES+
1.90e8
324.1048
325.1067
324.2732
324.3531
325.2863
325.3671
326.1020
326.1909
m/z
JR RR 20 (0.031) Is (1.00,1.00) C14H17N3O4S
1: TOF MS ES+
7.99e12
324.1018 (0.0 mDa)
325.1046 (0.0 mDa)
m/z

Compound 24 *– Ethyl imidazo-(1H)2-sulfanyl-1-yl acetate*

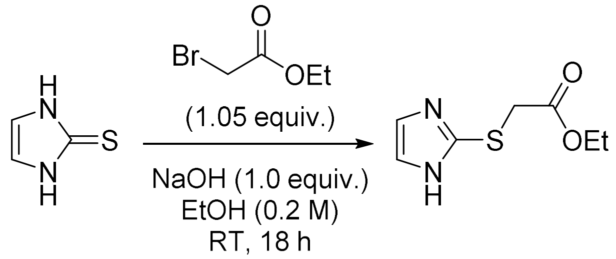

To a 25 mL RBF was added imidazole-2(1,3*H*)-thione (200 mg, 1.99 mmol) and NaOH (80 mg, 1.0 equiv.), EtOH (9.98 mL) was added, and the mixture stirred. To the stirred solution was added ethyl bromoacetate (0.22 mL, 1.05 equiv.) *via* syringe and the mixture left to stir at RT overnight. Afterwards the solvent was removed under reduced pressure and dissolved in EtOAc and NH_4Cl sat. soln., transferred to a sep. funnel, separated and the aqueous layer extracted with EtOAc (2 x 10 mL). The organic layers were collected and dried ($MgSO_4$) and solvent removed. The residue was purified by column chromatography (eluting with 2% MeOH / DCM) to provide ethyl imidazo-(1*H*)2-sulfanyl-1-yl acetate as a white solid (0.155 g, 42%).

^{1}H NMR (400 MHz, DMSO-d_6) δ 12.26 (br s, 1H), 7.03 (s, 2H), 4.06 (q, J = 7.1 Hz, 2H), 3.86 (s, 2H), 1.14 (t, J = 7.1 Hz, 3H).

^{13}C NMR (101 MHz, DMSO-d_6) δ 168.9, 137.6, 60.9, 35.1, 13.9 (5 out of 6 carbon resonances observed).

IR (cm^{-1}): 2989, 2628, 1720.

M.P. 73.2 – 74.8 °C.

MS ES+ Calcd for $C_7H_{10}N_2O_2S$ $(M+H)^+$: 188.0567, found: 188.0563.

Adapted from literature procedure[5].

NMR data:

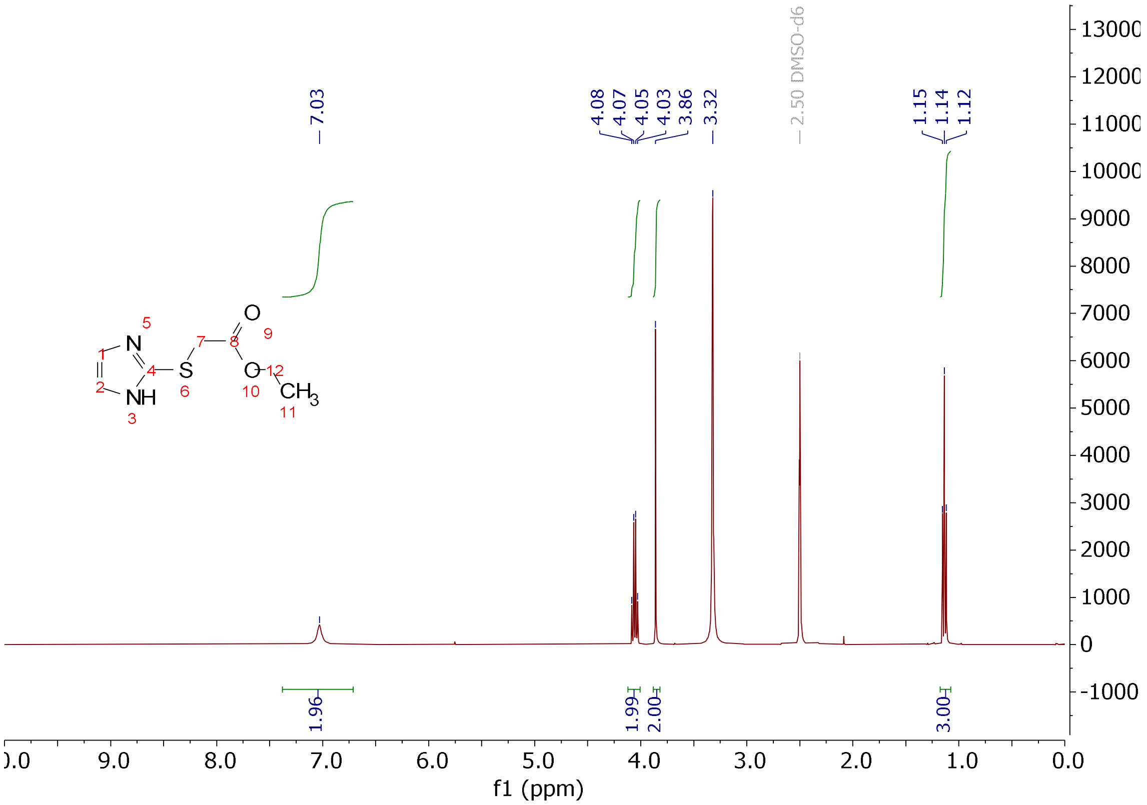

7.03
4.08
4.07
4.05
4.03
3.86
3.32
2.50 DMSO-d6
1.15
1.14
1.12
1.96
1.99
2.00
3.00
f1 (ppm)

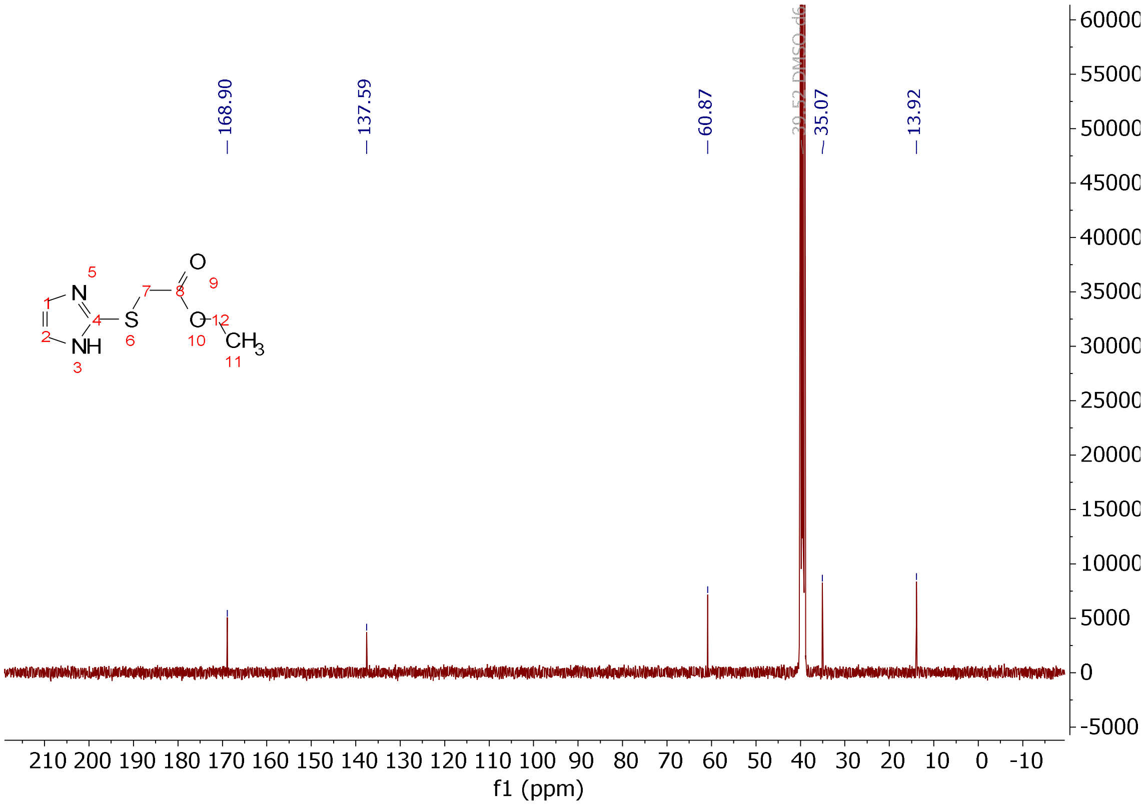

168.90
137.59
60.87
39.52 DMSO-d6
35.07
13.92
f1 (ppm)

Compound 25 *– 4-phenylpyrimidine-2-thiol*

NMR data:

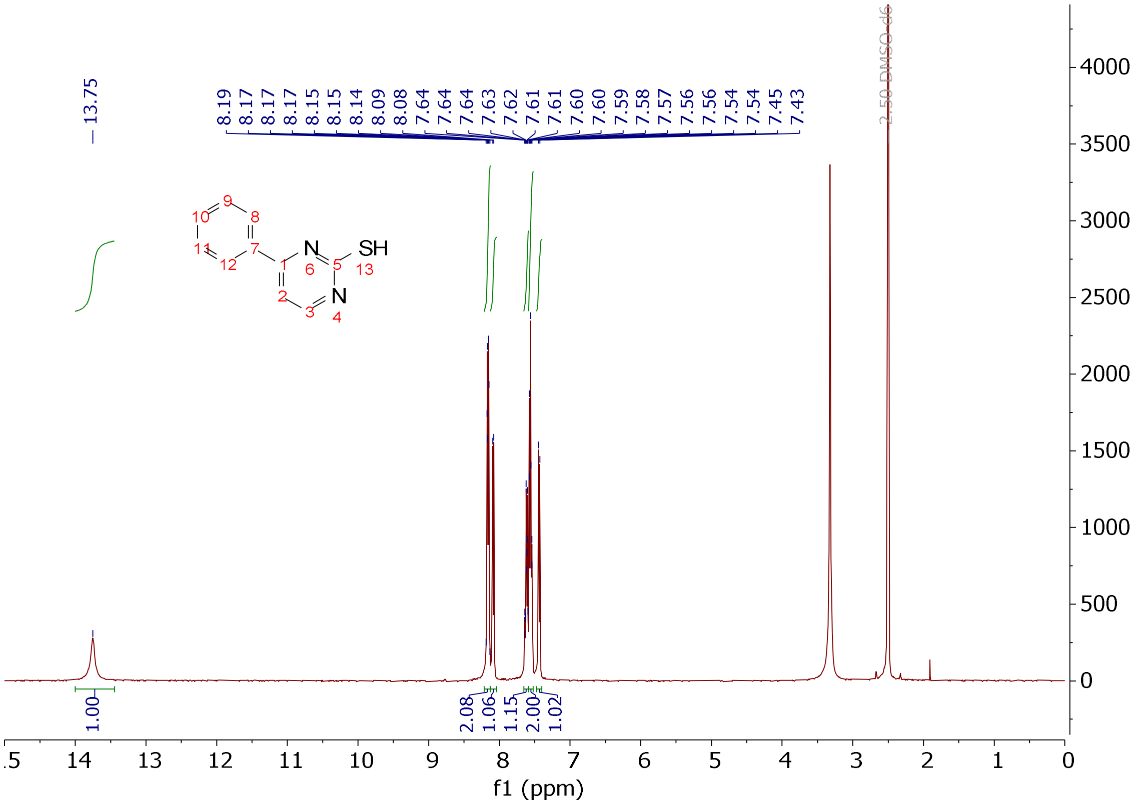

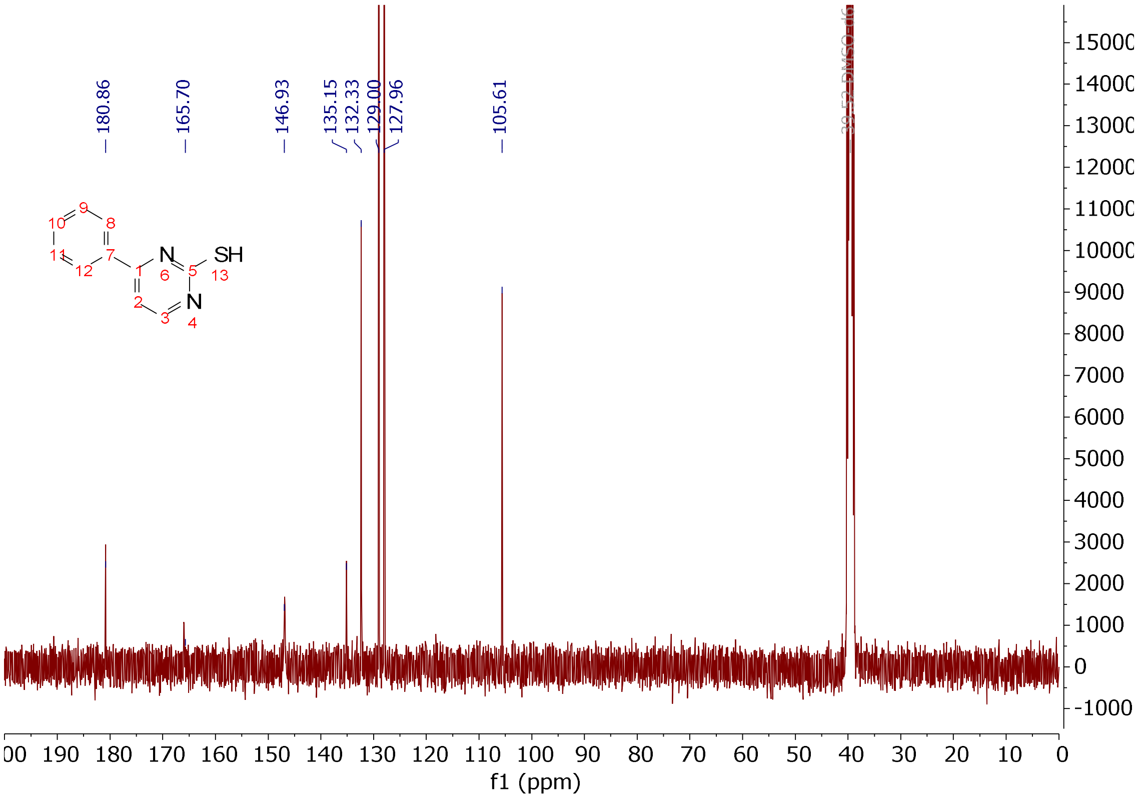

Compound 26 – *5-bromothiazolo[4,5-b]pyrazine-2-amine*

NMR data:

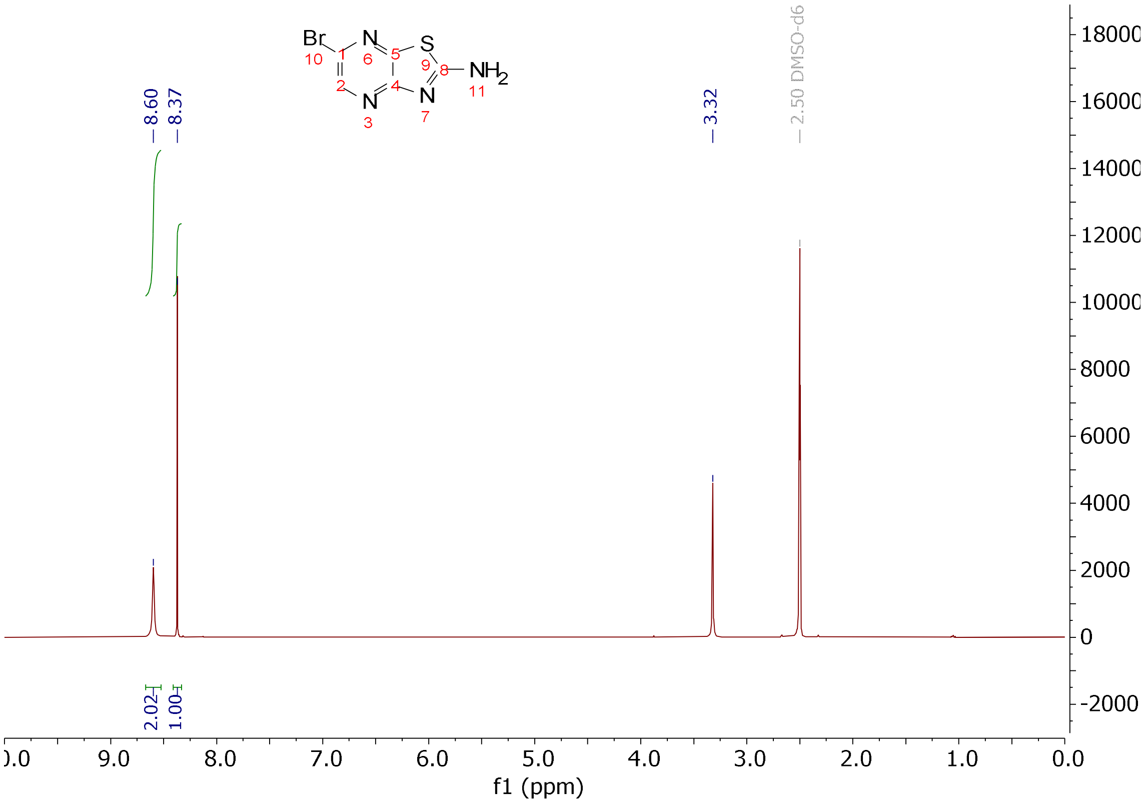

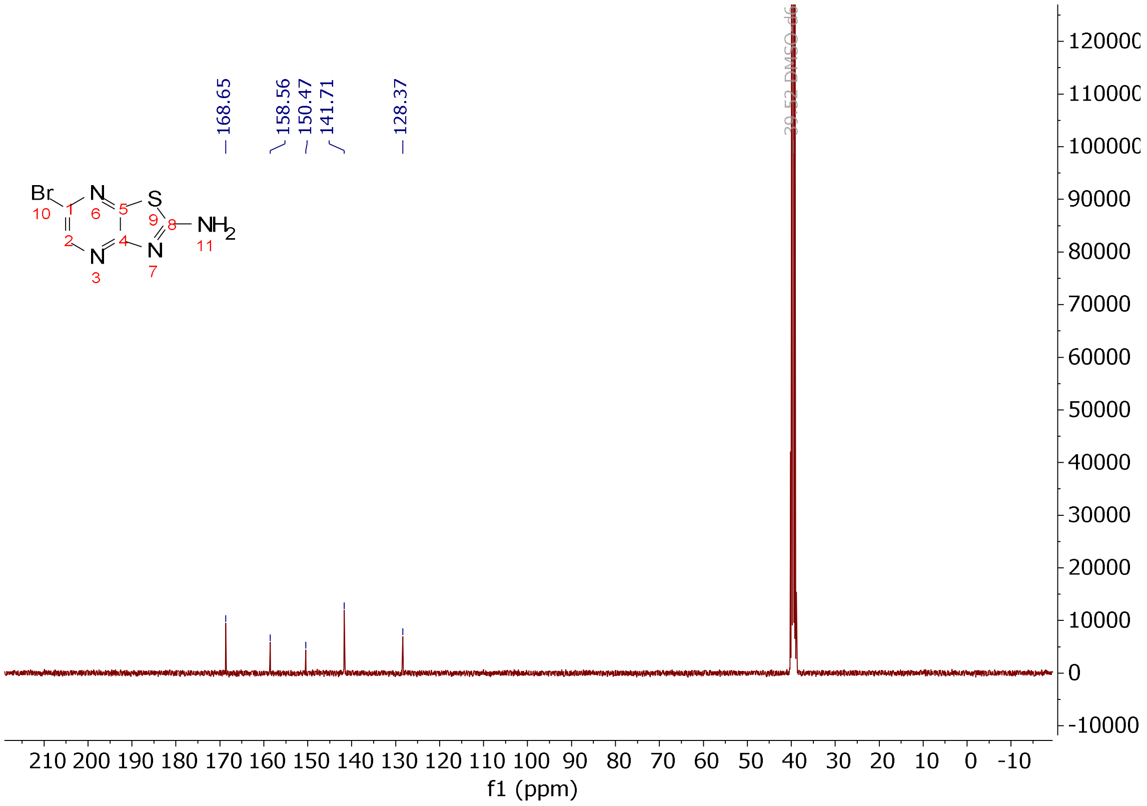

***Compound 27** – 3-(imidazo[1,2-a]pyrimidin-2-yl)propionic acid*

NMR data:

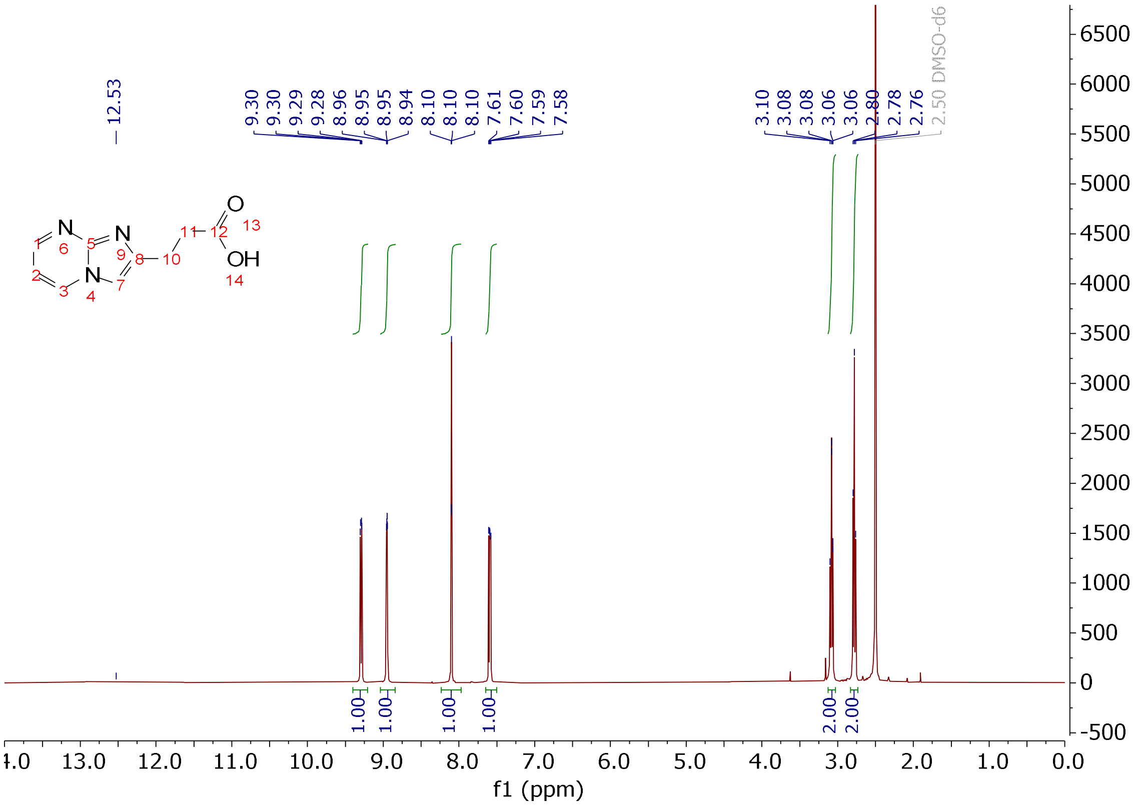

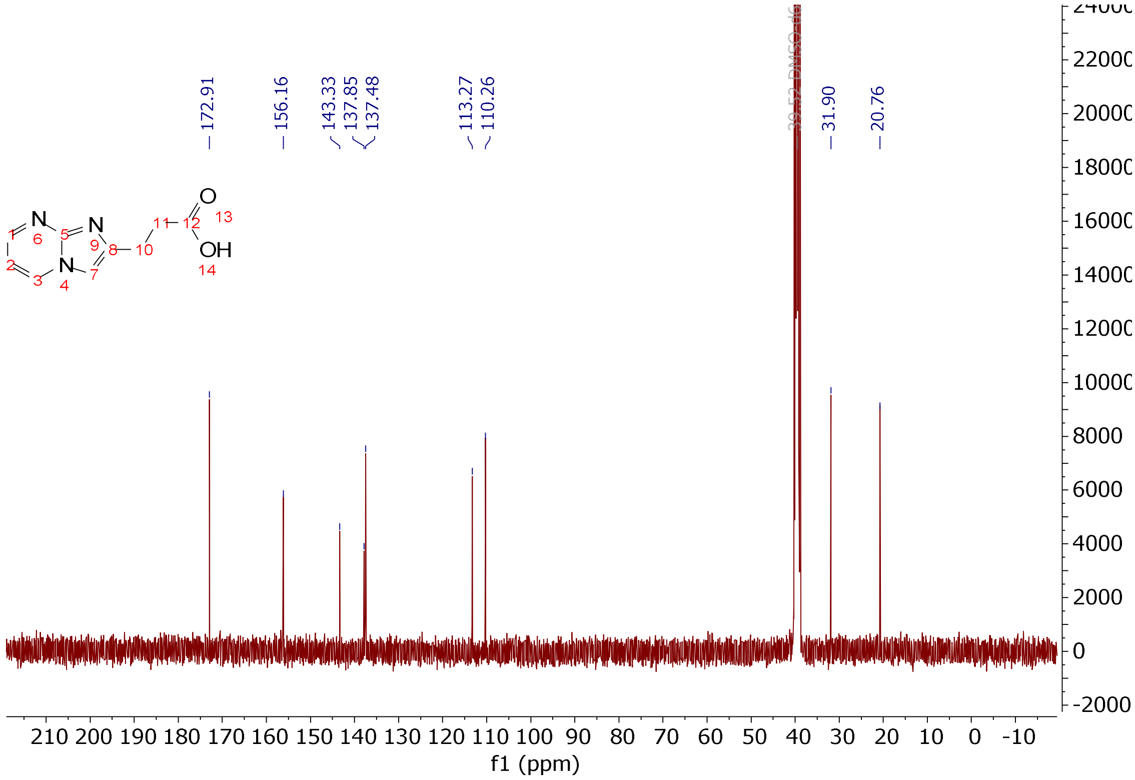

References

1. Sterling T, Irwin JJ. ZINC 15 - Ligand Discovery for Everyone. J Chem Inf Model. 2015;55(11):2324-2337.
2. Irwin JJ, Duan D, Torosyan H, Doak AK, Ziebart KT, Sterling T, Tumanian G, Shoichet BK. An Aggregation Advisor for Ligand Discovery. J Med Chem. 2015;58(17):7076-7087.
3. Berggren G, Sahlin M, Crona M, Tholander F, Sjöberg BM. Compounds with capacity to quench the tyrosyl radical in Pseudomonas aeruginosa ribonucleotide reductase. Journal of Biological Inorganic Chemistry. 2019;24(6):841-848.
4. Pichler A, Knipscheer P, Oberhofer E, van Dijk WJ, Körner R, Olsen JV, Jentsch S, Melchior F, Sixma TK. SUMO modification of the ubiquitin-conjugating enzyme E2-25K. Nat Struct Mol Biol. 2005;12(3):264-269.
5. Tong YC. lH-Irnidazo[4,5-b]pyrazines.1 11. 2-Thiols and Derivatives (1,2). J Heterocycl Chem. 1981;18(4):751-753
6. Pu X, Li Q, Lu Z, Yang X. N-Chloro-N-methoxybenzenesulfonamide: A Chlorinating Reagent. European J Org Chem. 2016;36:5937-5940.
7. Surase YB, Samby K, Amale SR, Sood R, Purnapatre KP, Pareek PK, Das B, Nanda K, Kumar S, Verma AK. Identification and synthesis of novel inhibitors of mycobacterium ATP synthase. Bioorg Med Chem Lett. 2017;27(15):3454-3459.
8. Lizano E, Grima J, Pujol MD. Efficient Halogenation of 2-Aminopyrazine. Synlett. 2019;30(17):2000-2003.
9. Wang M, Zhang Y, Wang T, Wang C, Xue D, Xiao J. Story of an Age-Old Reagent: An Electrophilic Chlorination of Arenes and Heterocycles by 1-Chloro-1,2-benziodoxol-3-one. Org Lett. 2016;18(9):1976-1979.
10. Ladd DL. Synthesis of some substituted guanidinopyrimidines and their structural assignment by 13C and 1H NMR. J Heterocycl Chem. 1982;19(4):917-921.
11. Chang L, Lee SY, Leonczak P, Rozenski J, De Jonghe S, Hanck T, Müller CE, Herdewijn P. Imidazopyridine- and purine-thioacetamide derivatives: Potent inhibitors of nucleotide pyrophosphatase/phosphodiesterase 1 (NPP1). J Med Chem. 2014;57(23):10080-10100.

12. Ibrahim N, Mouawad L, Legraverend M. Novel 8-arylated purines as inhibitors of glycogen synthase kinase. Eur J Med Chem. 2010;45(8):3389-3393.
13. Liu X, Zhang SB, Dong ZB. AlCl3-Promoted Synthesis of 2-Mercapto Benzoheterocycles by Using Sodium Dimethyldithiocarbamate as Thiocarbonyl Surrogate. European J Org Chem. 2018;39:5406-5411.
14. Pokorna A, Bobal P, Oravec M, Rarova L, Bobalova J, Jampilek J. Investigation of permeation of theophylline through skin using selected piperazine-2,5-diones. Molecules. 2019;24(3):566.
15. Fleischhauer J, Zahn S, Beckert R, Grummt UW, Birckner E, Görls H. A way to stable, highly emissive fluorubine dyes: Tuning the electronic properties of azaderivatives of pentacene by introducing substituted pyrazines. Chemistry - A European Journal. 2012;18(15):4549-4557.
16. Roiser L, Waser M. Enantioselective Spirocyclopropanation of para-Quinone Methides Using Ammonium Ylides. Org Lett. 2017;19(9):2338-2341.
17. Kiser PD, Zhang J, Badiee M, Kinoshita J, Peachey NS, Tochtrop GP, Palczewski K. Rational tuning of visual cycle modulator pharmacodynamics. Journal of Pharmacology and Experimental Therapeutics. 2017;362(1):131-145.
18. Meegan JE, Yang X, Rungsirisakun R, Cosgrove SC, Bushby RJ, Sadeghpour A, Rappolt M, Brydson R, Ansell RJ. Synthesis and organogelating behaviour of amino acid-functionalised triphenylenes. Soft Matter. 2017;13(35):5922-5932.
19. Li B, Berliner M, Buzon R, Chiu CK, Colgan ST, Kaneko T, Keene N, Kissel W, Le T, Leeman KR, et al. Aqueous phosphoric acid as a mild reagent for deprotection of tert-butyl carbamates, esters, and ethers. Journal of Organic Chemistry. 2006;71(24):9045-9050.
20. Sablayrolles C, Cros GH, Milhavet JC, Rechenq E, Chapat JP, Boucard M, Serrano JJ, McNeill JH. Synthesis of imidazo[1,2-a]pyrazine derivatives with uterine-relaxing, antibronchospastic, and cardiac-stimulating properties. J Med Chem. 1984;27(2):206–212.
21. Theodorou V, Skobridis K, Tzakos AG, Ragoussis V. A simple method for the alkaline hydrolysis of esters. Tetrahedron Lett. 2007;48(46):8320-8233.
22. Liszkiewicz H. New derivatives of 1H-imidazo[4,5-b]pyridine-2(3H)-thione. Synthesis of thiosemicarbazides and their cyclic analogues. Phosphorus Sulfur Silicon Relat Elem. 2008;183(6):1402-1409.
